# Structure Based Discovery of Inhibitors of CYP125 and CYP142 from *Mycobacterium tuberculosis*

**DOI:** 10.1002/chem.202203868

**Published:** 2023-04-12

**Authors:** Mona M. Katariya, Matthew Snee, Richard B. Tunnicliffe, Madeline E. Kavanagh, Helena I. M. Boshoff, Cecilia N. Amadi, Colin W. Levy, Andrew W. Munro, Chris Abell, David Leys, Anthony G. Coyne, Kirsty J. McLean

**Affiliations:** ^1^ Department of Biological and Geographical Sciences University of Huddersfield School of Applied Sciences Queensgate Huddersfield HD1 3DH UK; ^2^ Yusuf Hamied Department of Chemistry University of Cambridge Lensfield Road Cambridge CB2 1EW UK; ^3^ Department of Chemistry Manchester Institute of Biotechnology University of Manchester 131 Princess Street Manchester M1 7DN UK; ^4^ Department of Chemistry The Skaggs Institute for Chemical Biology The Scripps Research Institute La Jolla CA 92-37 USA; ^5^ Tuberculosis Research Section National Institute of Allergy and Infectious Diseases Laboratory of Clinical Immunology and Microbiology National Institutes of Health Bethesda MD 20892 USA

**Keywords:** cytochrome P450 enzymes, drug design, inhibitors, structure-activity relationships, tuberculosis

## Abstract

*Mycobacterium tuberculosis* (*Mtb*) was responsible for approximately 1.6 million deaths in 2021. With the emergence of extensive drug resistance, novel therapeutic agents are urgently needed, and continued drug discovery efforts required. Host‐derived lipids such as cholesterol not only support *Mtb* growth, but are also suspected to function in immunomodulation, with links to persistence and immune evasion. *Mtb* cytochrome P450 (CYP) enzymes facilitate key steps in lipid catabolism and thus present potential targets for inhibition. Here we present a series of compounds based on an ethyl 5‐(pyridin‐4‐yl)‐1H‐indole‐2‐carboxylate pharmacophore which bind strongly to both *Mtb* cholesterol oxidases CYP125 and CYP142. Using a structure‐guided approach, combined with biophysical characterization, compounds with micromolar range in‐cell activity against clinically relevant drug‐resistant isolates were obtained. These will incite further development of much‐needed additional treatment options and provide routes to probe the role of CYP125 and CYP142 in *Mtb* pathogenesis.

## Graphical abstract

**A structure‐based drug design process** is used to build inhibitors for the *Mycobacterium tuberculosis* (Mtb) cholesterol oxidase enzymes CYP125 and CYP142. Elaboration of initial fragment screen used an iterative approach combining high‐resolution X‐ray structures, biophysical characterization, and structure‐activity relationships (SAR). The resulting compounds yielded potent micromolar cellular activity against clinically relevant drug resistant Mtb strains.

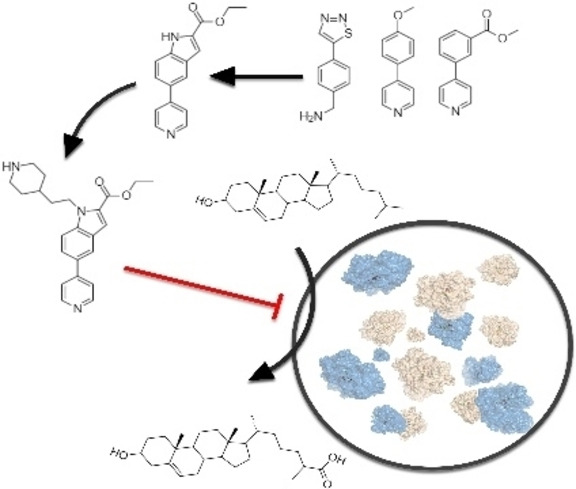

## Introduction

The ongoing battle against tuberculosis has received a significant setback in recent years due to the coronavirus pandemic, with 1.3 million (18 %) fewer diagnoses concomitant with 105,000 additional deaths in 2020 and 2021 versus 2019.^[1]^ Furthermore, drug resistance, which was first studied on the national level in 1955,^[2]^ continues to represent a significant threat, with rates of rifampicin‐resistant (RR‐TB) and multi drug‐resistant (MDR‐TB) tuberculosis ranging from 3–4 % in those who had not previously been treated, to 18 % in those who had undergone treatment in the past.^[1]^ More worryingly, resistance against recently developed antimicrobials such as Bedaquiline,[^3^, ^4^, ^5^, ^6^] and Delamanid[^3^, ^4^, ^7^, ^8^] has been documented in clinical isolates. Analysis of the *Mtb* genome gave the first indications that lipid and sterol degradation^[9]^ had an important function linked to its lifestyle as an obligate pathogen.^[10]^ It has been demonstrated that *Mtb* can grow with cholesterol as the sole carbon source,[^9^, ^11^] and its utilisation was found to be required for persistence of the bacterium in mice via a mechanism which is thought to involve subverting the IFN‐γ‐stimulated depletion of more typical carbon sources.^[12]^ Genes involved in sterol catabolism were also identified as virulence determinants in primates,^[13]^ and it has even been proposed that *Mtb* possesses a specialist sensor for cholesterol which mediates the interaction between the bacterium and the host cell membrane.^[14]^ Cholesterol is transported into *Mtb* via a large transmembrane complex encoded by the MCE4 operon.[^12^, ^15^, ^16^, ^17^] Breakdown is then initiated with the conversion of the C‐3 hydroxyl group to a ketone (cholest‐4‐en‐3‐one, cholestenone) by the oxidase ChoD,^[18]^ and further oxidation at the C‐26 “tail” region via a three‐step process involving oxidation to the hydroxyl, aldehyde, and carboxylic acid species. These sidechain‐modifying reactions are catalysed by the cytochromes P450 CYP125A1 and CYP142A1 (CYP125 and CYP142 hereafter).[^19^, ^20^, ^21^] It has been demonstrated that the CYP125 gene is essential for growth on cholesterol in the BCG strain of *Mycobacterium bovis*.^[19]^ Comparison of the effect of complementing a CYP125 knockout in the CDC1551 strain of *Mtb* (which does not express the CYP142 protein), with CYP125 or CYP142 reveals that the two enzymes are functionally redundant with respect to growth on cholesterol.^[22]^ Knockouts in the cholesterol transport complex result in a loss of persistence in infected animal lungs, and a defect in growth within IFN‐γ‐activated macrophages,^[12]^ and disruption of the CYP125 gene, as well as other genes in the intracellular growth (igr) operon results in a greatly reduced ability to kill THP‐1 cells.^[23]^ Similar transposon mutagenesis experiments have also indicated that CYP125 is a key determinant of Mtb survival in vivo.^[24]^

Cytochromes P450 were first recognized as potential therapeutic targets in *Mtb* as a result of work on *Mtb* CYP51, which revealed striking similarity to the eukaryotic sterol demethylase targeted by the azole class of antifungals.^[25]^ It is now known that the genome of *Mtb* encodes 20 cytochromes P450^[26]^ many of which bind strongly to azole drugs,[^20^, ^26^, ^27^, ^28^, ^29^, ^30^, ^31^] but despite the fact that azoles are indeed potent inhibitors of mycobacterial growth,[^30^, ^31^] their application for treatment of tuberculosis is limited by their inhibition of human sterol metabolism^[32]^ as well as hepatotoxicity.[^33^, ^34^, ^35^] The discovery of potent dual inhibitors of CYP125 and CYP142 might provide a valuable route to new chemotherapeutic agents to complement the existing range of antitubercular treatments.

## Results and Discussion

**Figure 1:**
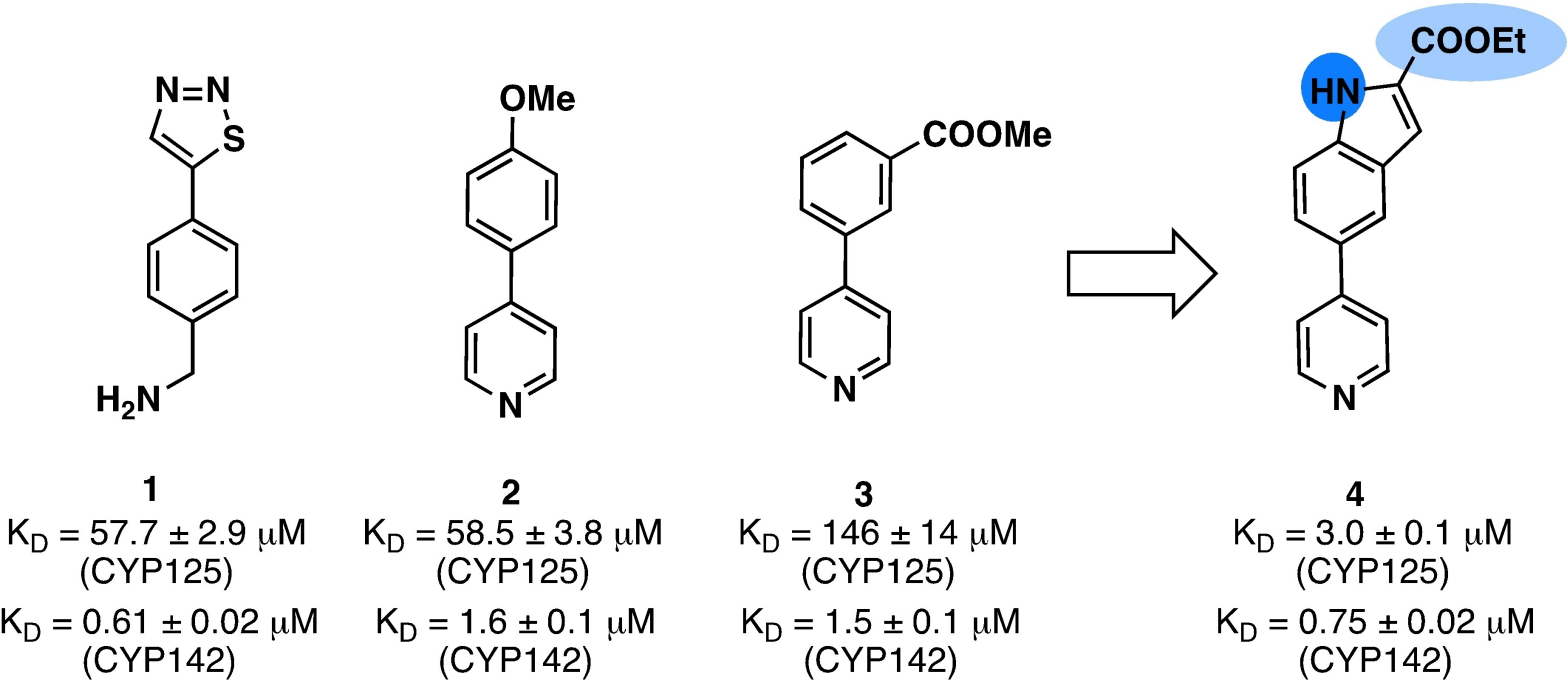
Fragment hits 1,2 and 3 identified for fragment elaboration. Initial optimisation led to the development of compound 4 and shows two vectors (indole NH and ethoxycarbonyl) for possible further elaboration. The *K*_D_ values were measured using UV‐vis spectroscopy.

**Figure 2:**
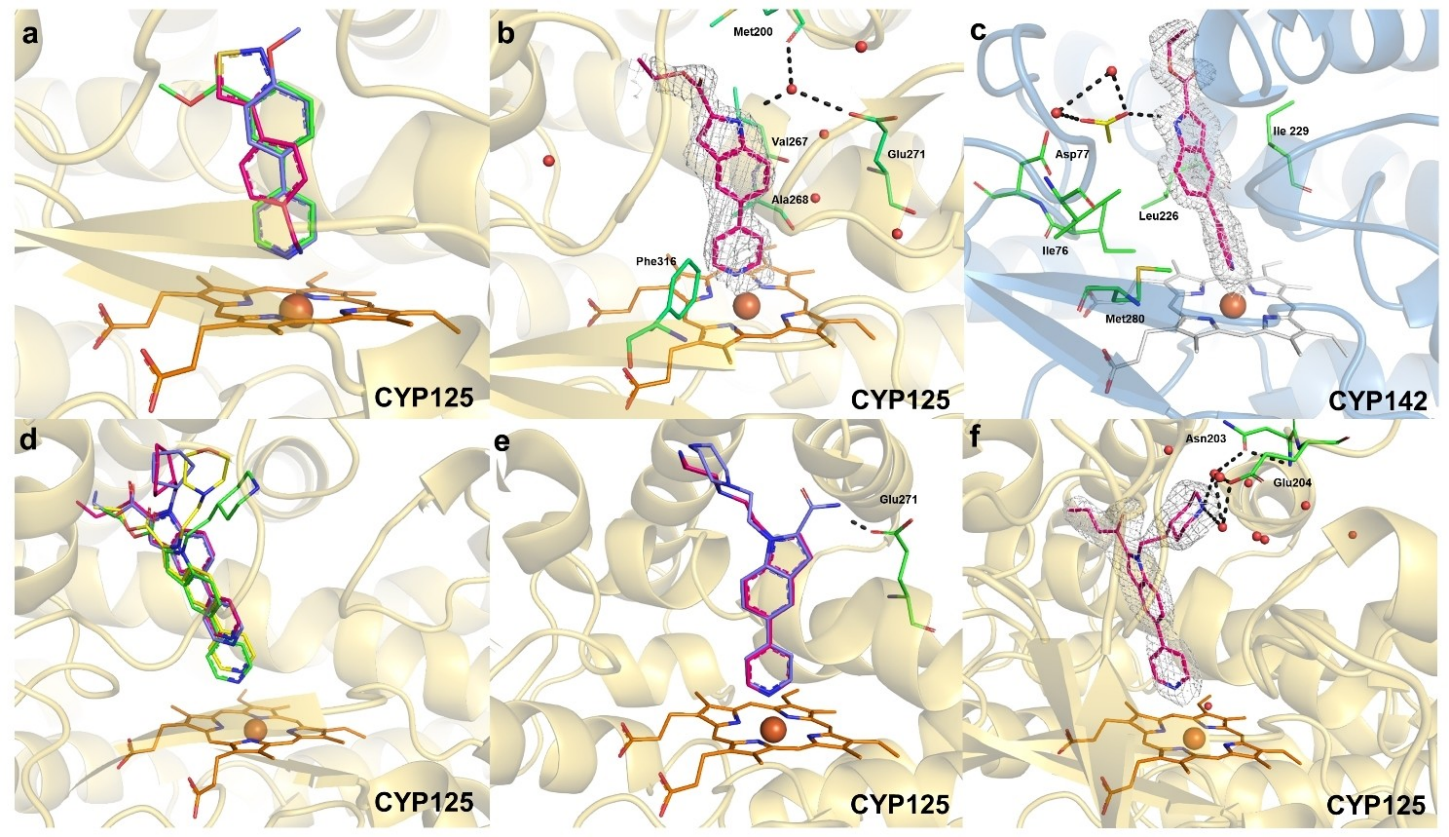
X‐ray co‐crystal structures illustrating the rational structure‐guided design of inhibitors targeting Mycobacterium tuberculosis CYP125 (heme shown in orange) and CYP142 (heme shown in grey). a) superimposing the observed binding modes of initial hits **1** (red), **2** (blue), and **3** (green) in CYP125 suggested a merging strategy resulting in compound **4**. b) compound **4** (red) adopts a pose in CYP125 that is consistent with those of its progenitor fragments c) The binding mode of compound **4** (red) in CYP142 is markedly different to that observed in CYP125 d) elaboration of compound **4** from the R^1^ position of the indole produces higher affinity compounds. In CYP125, compounds **7** (red) and **8** (blue), which feature hydrophobic ring structures are observed in a higher position above the heme iron relative to compound **10** (green) and **12** (yellow), which feature more hydrophobic ring structures on extended linkers. e) compounds **14** (red), and **15** (blue) exhibit dramatically different binding modes in CYP125 and are observed in direct coordination with the heme iron. This is likely due to the reduced bulk of the group found at the R^2^ position. f) compound **19** (red) exhibits a similar binding pose to its parent, compound **10**. Density shown is Fo−Fc density generated with the ligand omitted and is displayed at 3 sigma.

A focused library of eighty potential heme‐binding fragments was screened against CYP142 using UV‐vis spectrophotometry, leading to the identification of several fragment hits which bind directly to the heme iron (putative inhibitor‐like Type II or reverse Type‐1 ligations). The 4‐phenylpyridine scaffolds such as those identified in the fragment screen (Figure S**1**) were used as the start point for elaboration, with subsequent synthesis of derivatives expected to be more straightforward than other fragment hits (M. Kavanagh et al. unpublished work). To further explore the SAR on these fragment hits, compounds **2** and **3** were synthesized as possible start points for fragment elaboration. The OMe group in compound **2** and the COOMe group in compound **3** offer possible vectors for elaboration. In both cases, fragments **2** and **3** were shown by UV‐Vis spectrophotometry to bind to both CYP125 and CYP142 with good affinities (Figure 1). The affinity of these fragments for CYP125 is lower than that observed for CYP142. Compounds **2** and **3** were soaked into crystals of CYP125 and, upon structure solution, a direct interaction of the pyridyl nitrogen was observed with the heme iron (Figure 2). An alternate fragment **1** was identified from the fragment screen, the co‐crystal structure was obtained, and it was observed that the methanamine group directly interacts with the heme iron of CYP125. Fragment **1** was shown to be more selective for CYP142 (dissociation constant, *K*_D_=0.61 μM) in comparison to CYP125 (*K*_D_=57.7 μM). Examination of the overlap of the co‐crystal structures for compounds **1**–**3** in CYP125 (Figure 2) show that the incorporation of a five membered ring onto the phenyl ring offers a vector for further elaboration. This led to the development of compound **4**, where the pyridyl ring is fused onto the 5‐position of an indole ring. The indole ring has an ethoxycarbonyl group in the 2‐position and, along with the NH of the indole, provide vectors for further elaboration. The affinity (K_D_) of compound **4** was shown to be 3.0 μM (CYP125) and 0.75 μM (CYP142), an improvement in affinity in comparison to the original fragment hits. Interestingly the difference in affinity is not as significant between CYP125 and CYP142 in comparison to those observed with the fragment hits.

Structures of the compounds **1**–**3** in complex with CYP125 revealed hydrophobic interactions with the ligands are made with Leu117, Val267, Ala268, Phe316, Trp414, Leu415 (Figure 2a). Gratifyingly, the CYP125 binding mode is preserved when these fragments are merged and modified to develop compound **4** (Figure 2b). In CYP142, the hydrophobic central region of compound 4 is surrounded by Ile65, Ile76, Leu226, Ile229, Met280, and Phe380 (Figure 2c). Although compound **4** is similarly effective in bridging the hydrophobic active site regions in both CYP125 and CYP142, the observed pose is notably different. In CYP125, compound **4** extends perpendicular above the heme and the indole occupies a small pocket formed by Val267, Glu271, Trp414, and Leu415 which is not utilized by cholestenone (PDB code: 2X5 W)^[36]^ (Figure 2b). In CYP142, compound **4** binds at an altered angle relative to the plane of the heme, and the indole is observed perpendicular to the I helix, exploiting the space between Leu226 and Ile229 (Figure 2c). This bears some resemblance to the binding mode of cholestenone seen in *Mycobacterium smegmatis* CYP142 A2 (PDB code: 2YOO), the indole C3 occupying a similar region to C21 of cholestenone.^[37]^ In both CYP125 and CYP142 the indole nitrogen in compound **4** forms solvent‐mediated polar interactions. In CYP125 this involves a water molecule coordinated between the carboxyl oxygen of Met200, and the sidechain of Glu271 (Figure 2b), and for CYP142, the interaction occurs with an acetate ion derived from the crystallisation solution. Although this specific interaction would not be expected to occur in vivo, it is likely that a similar network of water‐based polar interactions does exist in the vicinity of Asp77 (Figure 2c). Combining information from the binding position of compound **4** in both CYP125 and CYP142 suggested that elaboration of the compound from the indole nitrogen offered opportunities to exploit the space above Trp414 and Leu415 (CYP125), or the pocket surrounded by Ile65, Asp77, and Leu72 (CYP142) to further improve affinity.

The NH vector on the indole ring was explored and a benzyl group was introduced to give compound **5** (Table 1). This led to an increase in affinity for both CYP125 (K_D_=0.65 μM) and CYP142 (K_D_=0.04 μM). Introduction of a carbonyl group on the methylene position, compound **6**, increases the CYP125 affinity further, but the affinity for CYP142 dropped (K_D_=0.42 μM). The introduction of a methylenecyclohexyl group on the indole NH, compound **7**, maintained affinity with both CYP125 and CYP142. Changing of the cyclohexyl moiety to a smaller cyclopentyl **8** or cyclobutyl ring **9** was explored. While compound **8** maintained the affinity with both CYP125 and CYP142, the smaller cyclobutyl ring in compound **9** was shown to have poorer affinity for CYP125. The development of compounds **5**–**9** led to high affinity ligands, that unfortunately suffered from poor solubility under assay conditions. To overcome these issues, the cyclohexyl group was substituted with piperazine and morpholine rings, compounds **10**–**12**, leading to enhanced solubility (Table 1).

**Table 1:** K_D_ values (determined using UV‐Vis spectroscopy) for compounds exploring SAR on the indole nitrogen.

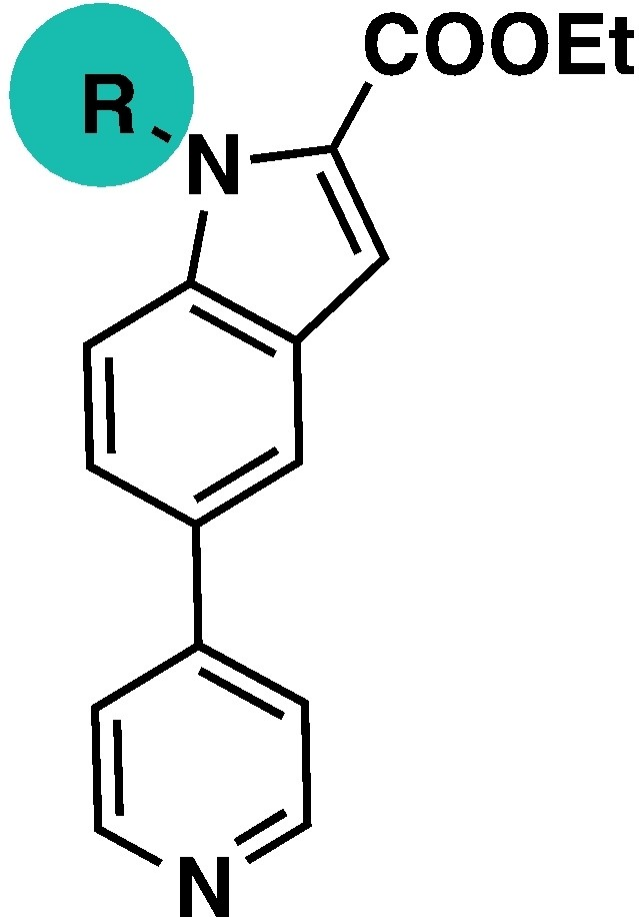			
Compound No.	R	K_D_ μM (CYP125)	K_D_ μM (CYP142)
**5**	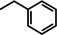	0.65±0.06	0.04±0.01
**6**	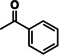	0.12±0.04	0.42±0.08
**7**	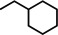	0.31±0.03	0.05±0.01
**8**	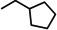	0.30±0.06	0.06±0.01
**9**	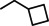	1.1±0.2	0.09±0.01
**10**	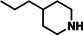	5.0±0.2	0.39±0.06
**11**	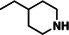	14.8±0.9	0.71±0.09
**12**	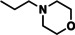	2.8±0.1	1.1±0.1

**Figure 3:**
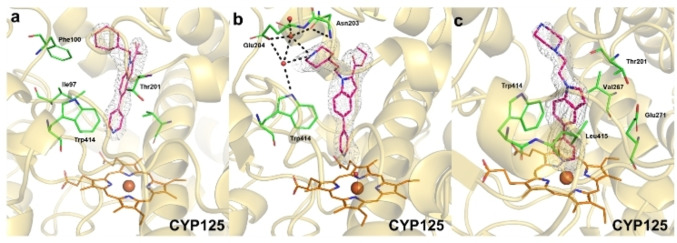
Active site exploitation in CYP125 a) Compound **7** is observed higher in the active site of CYP125 than expected, and it is possible that the conformational flexibility in the regions around Phe100 and Thr201, allow additional binding poses in solution. b) compound **10** is observed in a reverse Type‐I binding pose in CYP125. Changes to the composition of the ring structure and linker relative to **7** appear to promote more favourable interactions with the hydrogen bonding network associated with Asn203 and Glu204. c) In CYP125, compound **14** is rotated relative to other compounds with elaborations at the R1 position (**7**, **8**, **10**, **12**, **19**). This is likely due to a reduction in bulk of the group at the R2 position. This change allows the compound to occupy a previously unexploited pocket between Trp414, Leu415, Glu271, Val267, and thr201, which is unoccupied in the cholestenone‐bound structure of CYP125 (2X5 W). Density shown is Fo−Fc density generated with the ligand omitted and is displayed at 3 sigma.

Upon inspection of the corresponding CYP125 ligand complex X‐ray crystals structures, it was noted that elaboration from the R^1^ position (N of the indole) dramatically changes the binding pose of this series. In the cases of compounds **7**, **8**, **10**, and **12** the ligand is observed at a greater distance from the heme, typically with the pyridine coordination mediated via a water molecule, to produce a reverse‐type I interaction similar to what was observed for the inhibitor LP10^[38]^ (Figures 2d and 3). In the case of compounds **7** and **8**, which feature an aliphatic ring on a methylene linker, it appears that movement of the compound deeper into the active site is blocked by Phe100, leading to a distance of 3.9 Å and 3.3 Å, respectively between the pyridine nitrogen and the heme distal water molecule (Figure 3a). Later compounds such as **10** and **12** which feature longer linkers at the

R^1^ position are observed significantly closer to the heme iron, with compound **10** observed at 2.4 Å from the distal water (Figures 2e and 3b). This is likely attributable to the ability of the additional carbon to facilitate positioning of the ring closer to the plane of the indole, as opposed to compounds **7** and **8** which extend at an angle of 67° from this plane because of the geometric requirements of the single carbon linker. It is notable that compounds **5**–**9** (Table 1) and **13** (Table 2), which contain hydrophobic ring structures at the R^1^ position and a single carbon linker, have the highest affinities for CYP125 and CYP142. In the crystal structures of CYP125 in complex with **7** and **8**, Ile97 and Phe100, which form part of the loop preceding the B’ helix,^[29]^ are positioned below the R^1^ ring structures seemingly blocking further ingress of the compounds. In the same structures, Thr201, which is found in the loop preceding the F α‐helix^[29]^ is observed in a different conformation relative to that which is observed for compound **4**, forming a hydrogen bond with the sidechain of Glu271 and further restricting any downward movement of the compound. Both areas are crucial for substrate recognition, and are known to be capable of rearrangement,^[39]^ so future optimisation efforts may wish to consider that conformations may occur in solution that do allow direct type‐II interaction, allowing the R^1^ ring to access the hydrophobic pocket between Leu410, Trp414, and Leu415 at least a certain proportion of the time. Although all the structures presented here were produced via the soaking approach, attempts were made to obtain CYP125 and CYP142 co‐crystal structures via co‐crystallisation. Where this was successful, no change in the binding mode, or crystal form was observed. Later compounds such as **10**, **12**, and **19** have been optimized towards the observed pose of **7** and **8** in CYP125, with changes that gave improved solubility also allowing more favourable interactions with the more polar environment around Asn203, Glu204, the carboxyl oxygen of Arg99, and the N−H of Trp414 (Figures 2, 3).

**Table 2:** Affinity measurements (*K*_D_ using UV‐Vis spectroscopy) for compounds exploring the SAR on the 2‐position of the indole ring and the indole NH.

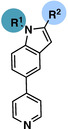				
Compound No.	R^1^	R^2^	K_D_ μM (CYP125)	K_D_ μM (CYP142)
**13**	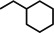	CONH_2_	0.45±0.04	0.03±0.007
**14**	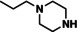	H	271±13	11.1±0.5
**15**	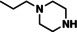	CONH_2_	48.8±2.3	2.5±0.2
**16**	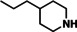	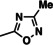	>100	0.39±0.05
**17**	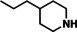	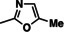	5.3±0.2	0.79±0.05
**18**	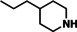	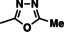	10.2±0.1	0.99±0.10
**19**	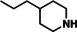	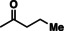	3.6±0.2	0.23±0.05

The vector off the 2‐position of the indole was next explored (Table 2), and the carboxyethyl group was replaced with an amide, compound **13**, and it was shown that the affinity was maintained. The replacement of the cyclohexyl group with a piperazine ring, **14**, showed a significant drop in affinity. The introduction of the amide group on the 2‐position of the indole **15** rescued some affinity however with both CYP125 and CYP142 these were both greater than 1 μM. In the X‐ray co‐crystal structures of compounds **14** (Figures 3c) and **15** in complex with CYP125, the pyridine coordinates directly with the heme iron, but in an orientation that is rotated almost exactly 180° around the heme vertical axis relative to compound **4**.

It is notable that compounds **14** and **15** have much lower affinity for both CYP125 and CYP142 when compared with compound **4**, **10**, and **12**, and it appears that a hydrogen bond between the amide of compound **15** and the carboxyl sidechain of Glu271 is responsible for the partial rescue of its affinity in CYP125 (Figure 2e). X‐ray structures for compounds **11** and **13** were not obtained, and thus the effect of the length of the linker or the hydrophilicity of the ring at the R^1^ position is unclear. It is likely however, that compound **13** shares its in‐solution binding characteristics with compounds **7** and **8** given that their affinities are very similar. The well‐resolved binding positions of compounds **14** and **15**, suggest additional future possibilities for exploiting the active site features of CYP125 beyond the regions that are occupied by cholestenone in the substrate bound structure 2X5W.^[36]^

Compounds **5**–**14** all contain either an ester or an amide in the 2‐position on the indole. Both groups are metabolically labile and replacement of these with isosteric heterocyclic groups could provide greater stability. Compounds **16**–**18** were designed where the ester functionality was substituted with 5‐membered heterocyclic rings. In almost all cases these maintain their affinity, except for compound **16** where the isosteric replacement of the ester with a 1,2,4‐oxadiazole caused a significant decrease in affinity, displaying a *K*_D_ >100 μM, although affinity to CYP142 was maintained. Compound **19** was developed where the ester was substituted with an alkyl ketone, and this gave similar affinity with CYP125 (*K*_D_=3.6±0.2 μM) and CYP142 (*K*_D_=0.23±0.05 μM) as the parent compound **10**. A selection of binding titration data is shown in the Supporting Information. Compound **19** binds in an almost identical manner to its parents (Figure 3), providing further proof that, although the size of the R^2^ group is an important determinant of binding pose and affinity, there is some degree of flexibility with respect to its composition. This would suggest that the R^2^ group (except for compound **15**) generally contributes towards affinity via an entropic mechanism involving the exclusion of ordered water from the binding cavity.

A selection of compounds, **16**–**19**, were screened against the Mtb H37Rv reference strain using different media conditions that supply different carbon and sugar sources for *Mtb* growth, including cholesterol for which CYP125 and CYP142 are essential to enable *Mtb* growth (Table S**1**). These compounds, **16**–**19** were also screened against a panel of susceptible and drug resistant Mtb strains. These data are summarised in Table 3.

**Table 3:** MIC Data (μM) against drug susceptible (DS), multi‐drug resistant (MDR) and extensively drug resistant (XDR) *Mtb* variants using a variety of growth media.

			MIC [μM]			
Mtb Strain		Growth Media	16	17	19	18^[a]^
H37Rv	Reference stain	1‐week MIC 7H9/glucose/casitone/Tx	4.7	4.7	6.25	19
		1‐week MIC 7H9/glucose/casitone/Tx	6.25	6.25	9.4	37
		1‐week MIC 7H9/glucose/glycerol/BSA/Tween	9.4	12.5	9.4	>50
		2‐week MIC 7H9/glucose/glycerol/BSA/Tween	12.5	19	12.5	>50
KB019	MDR+	1‐week MIC 7H9/glucose/glycerol/BSA/Tween	9.4	12.5	12.5	>50
		2‐week MIC 7H9/glucose/glycerol/BSA/Tween	12.5	19	19	50
K18b01MR	MDR	1‐week MIC 7H9/glucose/glycerol/BSA/Tween	6.25	6.25	6.25	50
		2‐week MIC 7H9/glucose/glycerol/BSA/Tween	6.25	6.25	6.25	50
NIH_G269DR	MDR	1‐week MIC 7H9/glucose/glycerol/BSA/Tween	6.25	6.25	6.25	>50
		2‐week MIC 7H9/glucose/glycerol/BSA/Tween	9.4	6.25	6.25	>50
K03b00DS	DS	1‐week MIC 7H9/glucose/glycerol/BSA/Tween	6.25	4.7	4.7	50
		2‐week MIC 7H9/glucose/glycerol/BSA/Tween	6.25	4.7	4.7	50
CDC1551	Clinical strain	1‐week MIC 7H9/glucose/glycerol/BSA/Tween	6.25	6.25	6.25	50
		2‐week MIC 7H9/glucose/glycerol/BSA/Tween	6.25	6.25	6.25	50
53 K113	XDR	1‐week MIC 7H9/glucose/glycerol/BSA/Tween	6.25	6.25	6.25	50
		2‐week MIC 7H9/glucose/glycerol/BSA/Tween	6.25	6.25	6.25	>50
028 K111	XDR	1‐week MIC 7H9/glucose/glycerol/BSA/Tween	6.25	6.25	6.25	50
		2‐week MIC 7H9/glucose/glycerol/BSA/Tween	6.25	6.25	6.25	>50

Gratifyingly compounds **16**, **17** and **19** showed good MIC's (minimal inhibitory concentrations) across all four media examined, which contained various carbon and sugar sources including cholesterol‐containing media (Table S1) and against several drug susceptible (DS), multi drug resistant (MDR), and extensively drug resistant (XDR) strains.^[40]^ CYP125 and CYP142 are involved in the initial oxidative breakdown of cholesterol. The finding that these compounds also inhibit mycobacterial growth on carbon sources other than cholesterol suggested that these likely inhibit other CYP targets. The MIC of compound **18** was shown to be poorer than the other three compounds. From a structural perspective there is a change in the position of the nitrogen on the oxadiazole ring in the structure of **19** in comparison to both **16** and **17** and this has a significant effect on the MIC. This highlights the challenges in generating compounds that can cross the cell wall and membrane structures of *Mtb* to access their cellular targets, where minor modifications can have a significant effect. The MIC for the compounds was measured at both 1 and 2 weeks, and the MIC remained relatively unchanged. This suggests that the compounds were stable during exposure to the cells in the respective growth media and that the bacilli were unable to overcome inhibition of the target by, for example, upregulation of alternative metabolic pathways. The compounds were screened against a panel of DS, XDR and MDR strains of *Mtb* and the MIC values were comparable to those in the H37Rv strain (Table 3). In‐cell activity was maintained for the CDC1551 strain of *Mtb* which lacks detectable expression of CYP142.^[22]^

## Conclusion

In this study, previously identified fragment hits were optimized against the targets CYP125 and CYP142 from *Mtb*. These are important targets and offer the possibility for the development of new antitubercular drugs with novel modes of action. The initial fragment hits were identified through the screening of focused heme library and were shown to bind to the heme iron in an inhibitor‐like Type II binding mode. The 3‐substituted biaryl pyridine **3** fragment was shown to have an affinity of 146 μM against CYP125 and 1.5 μM against CYP142, providing an excellent starting point for elaboration against both.

Subsequent efforts focused on the SAR on the phenyl ring and the pyridine ring was maintained to bind to the heme iron. The fusion and modification of fragments **1**–**3**, based upon information provided by the X‐ray structures, led to the development of a series of compounds (Table 1), the best of which had an affinity of 30 nM against CYP142. Subsequent further optimisation (Table 2) led to the development of more drug like molecules, where low nanomolar affinity compounds were developed. An isosteric replacement of the carboxy ester in the initial series with five membered ring heterocycles minimised future problems with this functionality. The availability of high‐resolution X‐ray crystal structures against both CYP125 and CYP142 were crucial in the development of these novel compounds, and the wealth of structural data produced may prove valuable in developing new chemical scaffolds for inhibitors of these enzymes. A series of these compounds showed good MIC against the H37Rv strain of *Mtb* as well as across a series of MDR and XDR strains. These are a first in class compounds which offer start points for the development of novel antitubercular drugs.

## Experimental Section

**Protein production**: CYP125 and CYP142 were expressed in C41(DE3) in 2 L shake flaks in 2xYT media. Cells were grown at 37 °C until OD_600_ of 0.8. 250 μM 5‐aminolevulinic acid and 200 μM Isopropyl ß‐D‐1‐thiogalactopyranoside (IPTG) were added cultures were incubated at 20 °C for 18 h. Cells were harvested centrifugation at 6000xg for 10 min. Harvested cells were lysed by sonication in 50 mM KPi pH 8.0, 200 mM KCl, 10 % v/v glycerol (buffer A) with the addition of 10 μg/mL of DNase and lysozyme. The lysate was clarified by centrifugation at 42,000×g for 1 h and the supernatant was applied to a steptactin XT high‐capacity resin (IBA) in a gravity column. The column was then washed with 20+ column volumes of buffer A and eluted in buffer A with 10 % IBA buffer BXT (biotin‐containing 10X concentrate). Protein concentration was estimated using Soret ϵ_395_ of 91 mM^−1^cm^−1^ for CYP125, and ϵ_418_ of 95 mM^−1^cm^−1^ for CYP142. Tobacco etch virus protease (TEV) was then applied with ratio of 1 : 20 (TEV:CYP) to enable tag removal with overnight incubation. Then incubated with Nickel‐NTA (Qiagen) for one hour to allow removal of TEV, uncleaved protein, and free tag. The resin was collected in a gravity‐flow column and the flow‐through was concentrated to 1 mL for gel filtration, typically using a HiLoad 16/600 Superdex 200 pg, in 20 mM HEPES pH 7.5, 200 mM KCl, 1 mM TCEP. Fractions were pooled and concentrated to 15 mg/mL and aliquots were either used directly for crystallisation experiments, or flash frozen in liquid nitrogen for storage at −80 °C.

**Protein crystallisation**: The sitting drop method was used with 15 mg/mL protein with a ratio of 1 : 1 at 4 °C. CYP125 crystallised in 0.1 M MES pH 6–6.5 with 1.7–2.0 M ammonium sulphate, and CYP142 crystallised in 0.1 M sodium acetate pH 4.5, 0.2 M KBr, 8 % PEG550 MME, 8 % PEG20,000.

**X‐ray data collection and structure solution**: X‐ray diffraction data was collected at various beamlines at the Diamond Light Source in Oxfordshire UK, and was indexed and integrated using the DIALS pipeline.^[41]^ Scaling and merging of intensities was performed using AIMLESS.^[42]^ Phasing of X‐ray data was performed using PHASER^[43]^ using 2XN8 and 2XKR as the search models for CYP125 and CYP142 respectively. Manual rebuilding of models was performed using Coot,^[44]^ and refinement was performed using PHENIX.refine.^[45]^

**Synthetic procedures**: All non‐aqueous reactions were performed under nitrogen atmosphere unless otherwise stated. Water‐sensitive reactions were performed in anhydrous solvents in oven‐dried glassware cooled under nitrogen before use. Petroleum ether refers to the fraction with a b.p. 40–60 °C, THF refers to tetrahydrofuran and DCM refers to dichloromethane. A rotary evaporator was used to remove the solvents *in vacuo*.

Thin layer chromatography was performed using Merck glass‐backed silica (Kieselgel 60 F_254_ 0.25 mm plates). Ultraviolet lamp (λ_max_=254 nm) and KMnO_4_ were used for visualization. Flash column chromatography was performed using an automated Isolera Spektra One/Four purification systems and an appropriately sized Biotage SNAP column containing KP‐silica gel (50 μm). Perkin–Elmer One FTIR spectrometer was used to analyse the infrared spectra. Absorptions are reported in wavenumbers (cm^−1^).

A SQD2 mass spectrometer detector (Waters) utilising electrospray ionization (ESI) was used for low‐resolution mass spectrometry (MS). High‐resolution mass spectrometry (HRMS) was recorded using a Waters LCT Premier Time of Flight (TOF) mass spectrometer or a Micromass Quadrapole‐Time of Flight (Q‐TOF) spectrometer.

The purity of compounds was determined by HPLC and was carried out using an Ultra Performance Liquid Chromatographic system (UPLC) Waters Acquity H‐class. All final compounds had purity greater than 95 % unless otherwise stated. Samples were detected using a Waters Acquity TUV detector at 2 wavelengths (254 and 280 nm). Samples were run using an Acquity UPLC HSS column and a flow rate of 0.8 mL/min. The eluent consisted of 0.1 % formic acid in water (A) and acetonitrile (B); gradient, from 95 % A to 5 % A over a period of 4 min.

Proton (^1^H), carbon (^13^C) and fluorine (^19^F) NMR data were collected on either a Bruker 400 MHz or 500 Mhz spectrometer. Data were collected at 300 K. Chemical shifts (δ) are given in parts per million (ppm) and they are referenced to the residual solvent peak. Coupling constants (*J*) are reported in Hertz (Hz) and splitting patterns are reported in an abbreviated manner: app. (apparent), s (singlet), d (doublet), t (triplet), q (quartet), m (multiplet), br (broad). Characterisatoin of individual compounds can be found in the Supporting Information.

## Conflict of interest

The authors declare no conflict of interest.

1

## Supporting information

As a service to our authors and readers, this journal provides supporting information supplied by the authors. Such materials are peer reviewed and may be re‐organized for online delivery, but are not copy‐edited or typeset. Technical support issues arising from supporting information (other than missing files) should be addressed to the authors.

Supporting Information

## Data Availability

The data that support the findings of this study are available in the supplementary material of this article.
