# Supplementary material for: Structure Based Discovery of Inhibitors of CYP125 and CYP142 from Mycobacterium tuberculosis
Source: Chemistry. 2023 Apr 12;29(29):e202203868. doi: 10.1002/chem.202203868 (PMC10205683; doi:10.1002/chem.202203868)
Supplement: Supplementary file 1 — Supporting Information [file CHEM-29-0-s001.pdf]

Chemistry–A European Journal

Supporting Information

Structure Based Discovery of Inhibitors of CYP125 and CYP142 from *Mycobacterium tuberculosis*

Mona M. Katariya, Matthew Snee, Richard B. Tunnicliffe, Madeline E. Kavanagh, Helena I. M. Boshoff, Cecilia N. Amadi, Colin W. Levy, Andrew W. Munro, Chris Abell, David Leys, Anthony G. Coyne,* and Kirsty J. McLean*

Expression of *Mtb* CYP125 and CYP142

The cholesterol oxidase enzymes were expressed in C41 (DE3) cells with a cleavable N-terminal twin-strep hexahistidine tag to enable convenient purification. The CYP125 construct was truncated to remove a structurally disordered N-terminal region, encoding residues 18-433, whereas the CYP142 construct encoded the full sequence minus the N terminal methionine (2-398). Sequences were generated as codon-optimised synthetic fragments (ThermoFisher) and were cloned into pET21a. The CYP125 surface-entropy reduction mutant utilised in this study was based upon the same CYP125 construct, with three lysine residues (K152, K332, and K333) identified by the UCLA (SER) server mutated to alanine via quick-change mutagenesis.

Cells transformed with these constructs were cultured in 2 L shake flasks containing 500 mL 2xYT medium (Formedium) and grown at 37°C with shaking until OD_{600} of 0.8. At this point, 5-aminolevulinic acid was added to a concentration of 250 µM to stimulate better heme incorporation. Protein expression was then initiated using 200 µM Isopropyl ß-D-1-thiogalactopyranoside (IPTG) and cultures were incubated at 20°C for a further 18 hours prior to harvesting. Cells expressing the recombinant enzymes were harvested by centrifugation at 6000xg for 10 minutes and flash frozen in liquid nitrogen for storage at -80°C

Purification

To isolate the recombinant proteins, cells were thawed and lysed by sonication in 50 mM Kpi pH 8.0, 200 mM KCl, 10% v/v glycerol (buffer A) with the addition of 10 µg mL^{-1} of DNase and lysozyme (Sigma). The lysate was clarified by centrifugation at 42,000xg for 1 hour and the supernatant was applied to a steptactin XT high-capacity resin (IBA) in a gravity column. The column was then washed with 20+ column volumes of buffer A to remove unbound proteins and then eluted with an elution buffer consisting of buffer A with 10% IBA buffer BXT (biotin-containing 10X concentrate). The concentration of enzyme in the elution fraction was estimated spectroscopically using a Soret extinction coefficient of 91 mM^{-1} cm^{-1} at 395 nm (abs of 1.952 at 1 mg/mL) for CYP125 and a value of 95 mM^{-1} cm^{-1} at 418 nm (abs of 2.076 at 1 mg/ml) for CYP142 (unpublished data). Tobacco etch virus protease (TEV) was then applied to an approximate ratio of 1:20 (TEV:CYP) to enable tag removal. Cleavage was allowed to proceed overnight, and the cleaved sample was then incubated with Nickel-NTA (Qiagen) for one hour to allow removal of TEV, uncleaved protein, and free tag. The resin was collected in a gravity-flow column and the flow-through was concentrated to 1 mL for gel filtration, typically using a HiLoad 16/600 Superdex 200 pg, in 20 mM HEPES pH 7.5, 200 mM KCl, 1 mM TCEP. Size-exclusion fractions from the central region of the main peak where pooled and concentrated to 15 mg mL^{-1} and aliquots were either used directly for crystallisation experiments, or flash frozen in liquid nitrogen for storage at -80°C.

Crystallisation of CYP125 and CYP142

Crystallisation was performed using the sitting drop method at 15 mg mL^{-1} with a mixing ratio of 1:1 at 4°C. CYP125 crystallised in 0.1 M MES pH 6-6.5 with 1.7-2.0 M ammonium sulphate, and CYP142 crystallised in 0.1 M sodium acetate pH 4.5, 0.2 M KBr, 8% PEG550 MME, 8% PEG20,000. The CYP125 surface-entropy reduction mutant crystallised in 0.1 M Sodium cacodylate pH 5.0, 0.2 M Sodium acetate trihydrate, 22% V/V PEG8000.

X-ray data collection and structure solution

X-ray diffraction data was collected at various beamlines at the Diamond Light Source in Oxfordshire UK, and was indexed and integrated using the DIALS pipeline.[1] Scaling and merging of intensities was performed using AIMLESS.[2] Phasing of X-ray data was performed using PHASER[3] using 2XN8 and 2XKR as the search models for CYP125 and CYP142 respectively. Manual rebuilding of models was performed using Coot,[4] and refinement was performed using PHENIX.refine[5].

Steady-state binding affinity measurement (UV Visible spectrophotometry).

Optical Assay: Optical titrations were performed using a CARY400 (BMG Labtech) in the F-bottom well plate (Greiner Bio-one, UK). Assays were performed in reduced volume (1 mL). Fragments (0.1 mM−100 mM) and ligands (0.0001 mM − 100 mM) were prepared as stock solutions in DMSO-d_6 and proteins (4−6 μM) were prepared in HEPES (20 mM), KCl (150 mM), pH 7.5. Aliquots (0.2 μL) of fragment and ligand stock solutions were added directly to cuvette containing either protein solutions, or buffer and the final DMSO-d_6 concentration of 4% v/v of the assay solution. Spectra were recorded continuously between 800−250 nm at 25 °C. To account for any inherent absorbance of added ligands/solvents, buffer control spectra were subtracted from protein spectra. Final difference spectra were obtained by subtracting the initial ligand-free protein spectrum from successive titration spectrum. The maximum change in absorbance for each difference spectrum was then plotted against ligand concentration.
Final binding curves were fitted using non-linear regression a one-site equilibrium binding model hyperbolic/Michaelis-Menten equation $A_{obs} = (A_{max} \times L)/ (K_D + L)$. For tight binding inhibitors a modified version of the Morrison equation $A_{obs} = (A_{max}/2Et) \times ((L + Et + K_D) – (((L + Et + K_D) 2) – (4 \times L \times Et))0.5)$ was used to fit experimental data to the spectra. A_{obs} is the observed change in absorbance, A_{max} is the maximum absorbance change at saturation, L is the concentration of ligand Et is the enzyme concentration and K_D is the dissociation constant for the P450- fragment and P450-ligand complex. Data were processed using Microsoft Excel (Microsoft Office, 2010) and data fitting and analysis were performed using GraphPad Prism 5.01 (GraphPad Software, San Diego, USA). (Figures S2 and S3)

Minimum inhibitory concentration (MIC) determination against *M. tuberculosis*.

M. tuberculosis strains including H37Rv (ATCC 27294) and a panel of drug-resistant clinical isolates[6] were grown in the respective medium of choice with a variety of carbon and sugar sources to an OD_{650nm} of 0.2. Media were either (1) Middlebrook 7H9 medium supplemented with 0.5% BSA fraction V, 0.08% NaCl, 0.2% glucose, 0.2% glycerol and 0.05% Tween 80 (7H9/glucose/glycerol/BSA/Tw), (2) Middlebrook 7H9 medium supplemented with 0.5% BSA fraction V, 0.08% NaCl, 0.4% glucose, and 0.05% Tyloxapol (7H9/glucose/BSA/Tx), (3) Middlebrook 7H9 medium supplemented with 0.03% Bacto casitone, 0.08% NaCl,

0.4% glucose, and 0.05% Tyloxapol (7H9/glucose/casitone/Tx), (4) Middlebrook 7H9 medium supplemented with 0.5% BSA fraction V, 0.08% NaCl, 5 mg/mL dipalmitoyl phosphatidylcholine, 24 mg/mL cholesterol and 0.05% Tyloxapol (7H9/DPPC/cholesterol/BSA/Tx), or (5) Middlebrook 7H9 medium supplemented with 0.03% Bacto casitone, 0.08% NaCl, 14 mg/mL dipalmitoyl phosphatidylcholine, and 0.05% Tyloxapol (7H9/DPPC/casitone/Tx). The cultures were diluted 1000-fold in BSA-containing or 500-fold in BSA-free fresh medium and 50 mL dispensed per well in round-bottom clear 96-well plate (Nunclon) containing 50 mL of the respective medium per well, with or without 2-fold serial dilutions of compound. Plates were sealed in ziplock bags and incubated at 37 ℃ for 2 weeks. Growth was recorded after 1- and 2- weeks of growth by monitoring growth with an inverted enlarging mirror. The MIC was determined as the lowest concentration that completely inhibited growth. Each compound was tested in duplicate. DMSO was used as negative control which resulted in no growth inhibition up to 2%. Isoniazid served as positive control drug.

	CYP125	CYP125	CYP125	CYP125	CYP125 (SE)	CYP125 (SE)	CYP125	CYP125	CYP125	CYP125	CYP125	CYP142
	Compound 1	Compound 2	Compound 3	Compound 4	Compound 7	Compound 8	Compound 10	Compound 12	Compound 14	Compound 15	Compound 19	Compound 4
	(PDB 7R3U)	(PDB 7ZQR)	(PDB 7ZSU)	(PDB 7QWN)	(PDB 7QKE)	(PDB 7QNN)	(PDB 7R1I)	(PDB 7ZLZ)	(PDB 7YXF)	(PDB 7ZT0)	(PDB 7ZXD)	4(PDB 7QJL)
Data collection												
Space group	C 1 2 1	C 1 2 1	C 1 2 1	C 1 2 1	$C\ 2\ 2\ 2_1$	$C\ 2\ 2\ 2_1$	C 1 2 1	C 1 2 1	C 1 2 1	$P\ 2_1 2_1 2_1$	C 1 2 1	$P\ 2_1\ 2_1\ 2_1$
**Cell dimensions**												
a, b, c (Å)	137.40, 68.70, 144.15	136.78, 69.11, 144.79	137.20, 68.90, 144.10	137.05, 69.20, 144.54	54.01, 119.52, 145.75	54.36, 120.02, 145.62	137.94, 69.47, 144.90	137.44, 69.00, 144.65	136.44, 69.38, 144.35	67.81, 90.10, 152.87	137.24, 69.22, 144.14	55.52, 66.17, 130.01
α, β, γ (°)	90, 93.9, 90	90, 94.0, 90	90, 94.0, 90	90, 94.4, 90	90, 90, 90	90, 90, 90	90, 94.1, 90	90, 94.6, 90	90, 94.3, 90	90, 90, 90	90, 94.35, 90	90, 90, 90
Resolution (Å)	1.86	1.79	2.2	1.93	2.3	2.47	2.24	1.89	1.85	1.99	2.09	1.38
No. reflections (total)	378610 (19068)	419208 (19495)	230178 (14951)	338029 (15498)	279194 (25219)	225020 (25484)	223516 (15120)	366311 (18365)	337245 (17178)	428105 (30884)	548327 (28803)	681767 (31069)
No. reflections (unique)	112646 (5512)	126949 (6281)	68265 (4587)	101498 (5012)	21451 (2058)	17538 (1939)	66030 (4386)	108204 (5366)	114696 (5642)	65089 (4531)	79538 (4496)	99205 (4848)
R_{merge}	0.051(0.901)	0.056 (0.675)	0.072 (0.480)	0.087 (0.903)	0.159 (1.202)	0.220 (1.597)	0.079 (0.597)	0.043 (1.002)	0.063 (0.930)	0.071 (1.474)	0.054 (0.470)	0.087 (1.509)
I / σI	10.5 (1.3)	8.9 (1.4)	9.1 (2.6)	5.9 (1.1)	11.1 (1.4)	7.8 (1.0)	8.5 (1.7)	12.6 (1.1)	9.4 (1.1)	11.4 (1.1)	19.5 (3.1)	8.2 (1.1)
CC 1/2	0.998 (0.545)	0.997 (0.513)	0.996 (0.662)	0.995 (0.505)	0.994 (0.924)	0.991 (0.849)	0.997 (0.717)	0.997 (0.521)	0.006 (0.503)	0.998 (0.627)	0.999 (0.904)	0.996 (0.527)
Completeness (%)	99.9 (99.8)	99.9 (99.7)	99.8 (99.5)	99.9 (100.0)	100.0 (100.0)	100.0 (100.0)	99.9 (100.0)	100.0 (99.8)	99.8 (99.6)	100.0 (100.0)	99.3 (99.2)	100.0 (100.0)
Multiplicity	3.4 (3.5)	3.3 (3.1)	3.4 (3.3)	3.3 (3.1)	13 (12.3)	12.8 (13.1)	3.4 (3.4)	3.4 (3.4)	3.3 (3.0)	6.6 (6.8)	6.9 (6.4)	6.9 (6.4)
Refinement												
Rwork / Rfree	19.5 / 22.3	18.0 / 20.8	0.21 / 0.23	0.18 / 0.22	0.19 / 0.23	0.20 / 0.25	0.20 / 0.24	0.19 / 0.22	0.18 / 0.22	0.20 / 0.24	0.20 / 0.22	0.15 / 0.17
**R.m.s. deviations**												
Bond lengths (Å)	0.004	0.008	0.003	0.005	0.003	0.004	0.003	0.008	0.012	0.003	0.003	0.013
Bond angles (°)	0.678	0.903	0.581	0.742	0.57	0.713	0.611	0.762	1.072	0.632	0.572	1.382
Outliers (%)												
Ramachandran	0	0	0.1	0	0	0	0	0	0.1	0	0	0
RSRZ	2.9	4.4	6	3.7	2.4	0.7	4.3	1.4	1.2	4.4	7	2.8
MolProbity	1.12	1.28	1.33	1.19	0.95	1.68	1.34	1.23	1.24	1.4	1.32	1.22

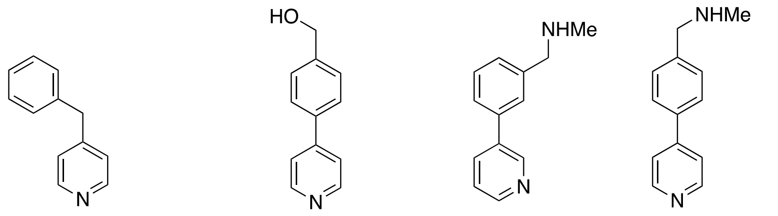

NMR 170
Δλ = +4 nm (1 mM, CYP142)
K_d = 150 μM (CYP125)
K_d = 1.9 μM (CYP142)

NMR084
Δλ = +2 nm
(1 mM, CYP142)

NMR195
Δλ = +3 nm
(1 mM, CYP142)

NMR425
Δλ = +3 nm
(1 mM, CYP142)

Figure S1: Fragment hits identified from heme-focused fragment library.

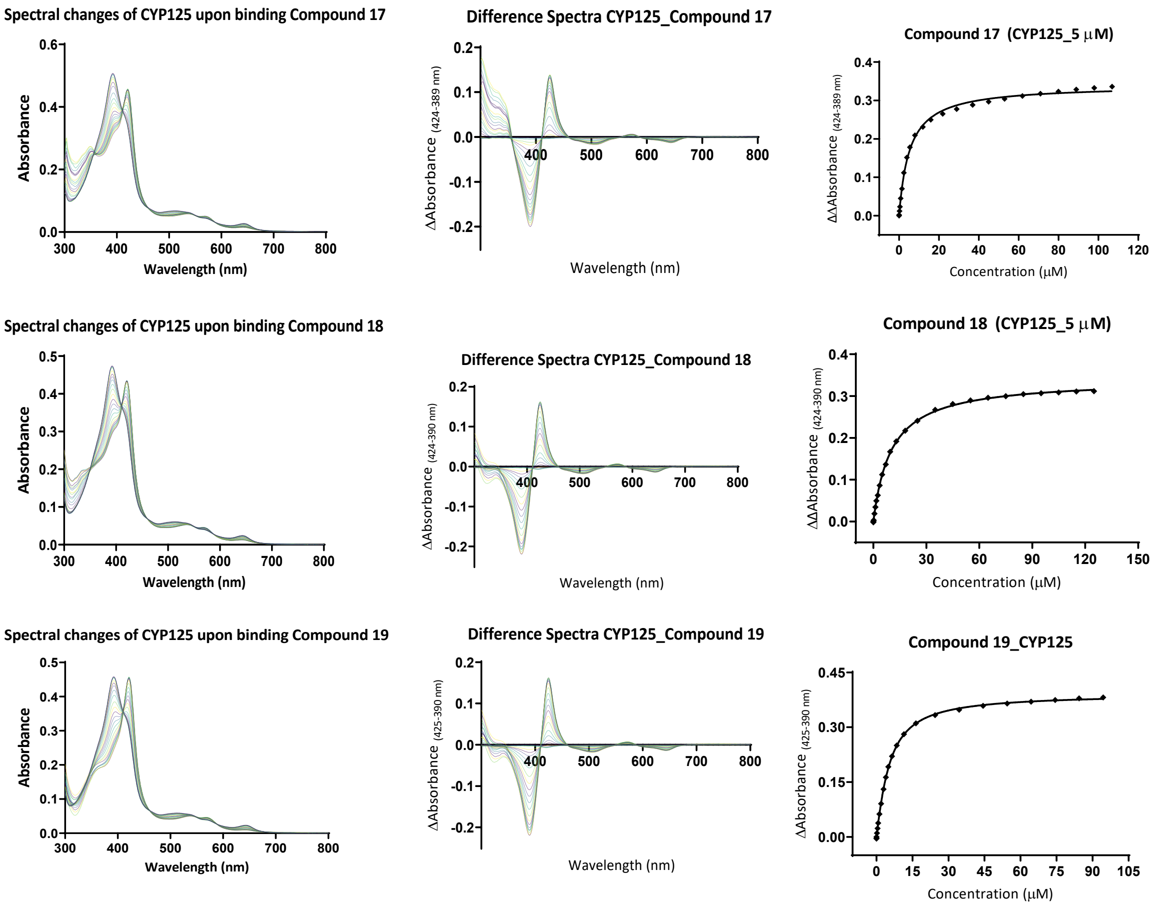

Figure S2: UV-vis binding titrations for CYP125 with compounds 17, 18,19.

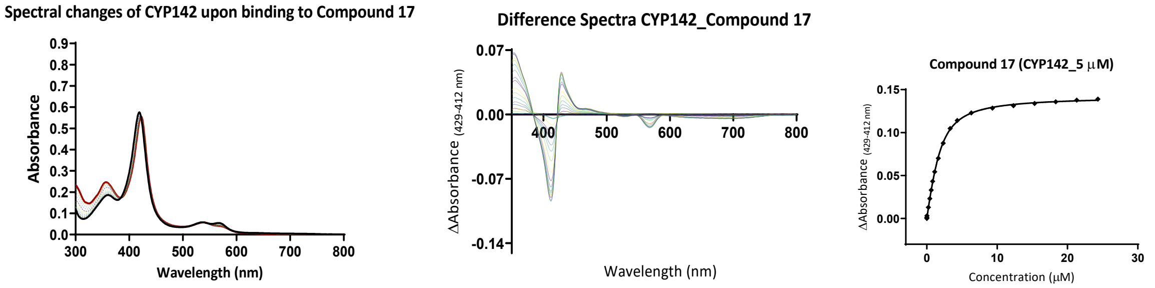

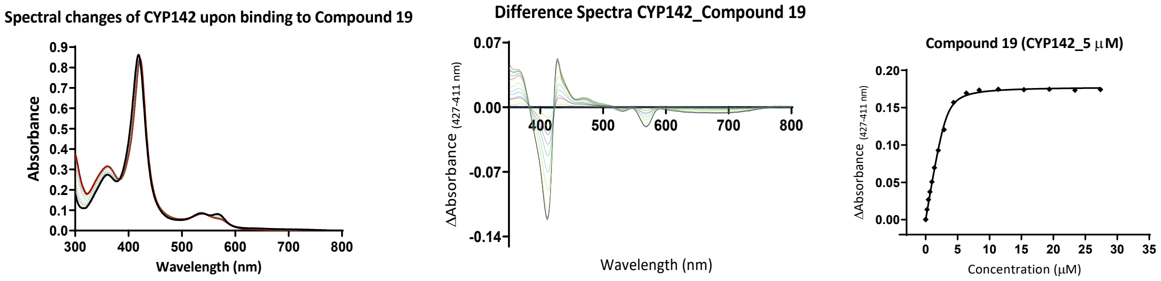

Figure S3: UV-vis binding titrations for CYP125 with compound 17.

Table S1: MIC Data (μM) against *Mtb* (H37Rv) in media containing different carbon sources. CYP125 and CYP142 are required for *Mtb* growth in the presence of cholesterol.

	7H9/glucose/casitone/Tx	7H9/glucose/casitone/Tx	7H9/glucose/BSA/Tx	7H9/glucose/BSA/Tx	7H9/DPPC/casitone/Tx	7H9/DPPC/casitone/Tx	7H9/DPPC/cholesterol/BSA/Tx	7H9/DPPC/cholesterol/BSA/Tx
	**1 Week**	**2 Week**	**1 Week**	**2 Week**	**1 Week**	**2 Week**	**1 Week**	**2 Week**
Isoniazid (+ve control)	0.15	0.2	0.2	0.3	0.1	0.1	0.15	0.2
**16**	6.25	9.4	25	50	6.25	6.25	9.4	9.4
**17**	6.25	9.4	25	50	6.25	6.25	9.4	9.4
**19**	9.4	9.4	12.5	37	12.5	12.5	9.4	12.5
**18**	25	50	>50	>50	25	25	25	50

Table S2: MDR and XDR Strains

	**Clinical Resistance**	**Drug susceptibility testing (DST)**
Kb019	MDR+	HREPKOTh
K18b01MR.NIH 79	MDR	HRERb
NIH_G269DR	MDR	HRERb
K03b00DS 57	DS	-
CDC1551	DS	-
053K113	XDR	HRSKOP, Cap, Amk, Pth, Mfx, Lev, Rbu, PZA, LZD
028K111	XDR	HRESKP, Cap, Amk, Pth, Cs, Ofx, Mfx, Lev, Rbu, PZA, LZD

Drugs: H = isoniazid, R = Rifampicin (Rifampin), E = Ethambutol, P = para-aminosalicylic acid, K = Kanamycin, O = Ofloxacin, Th = Thiacetazone, Rb = Rifabutin, S = Streptomycin, Ofx = Ofloxacin, Pth = Prothionamide, Cap = Capreomycin, Rbu = Rifabutin, PZA = Pyrazinamide, Z = Pyrazinamide, LZD = Linezolid, Cs = Cycloserine, Amk = Amikacin, Lev = Levofloxacin

Synthetic Chemistry

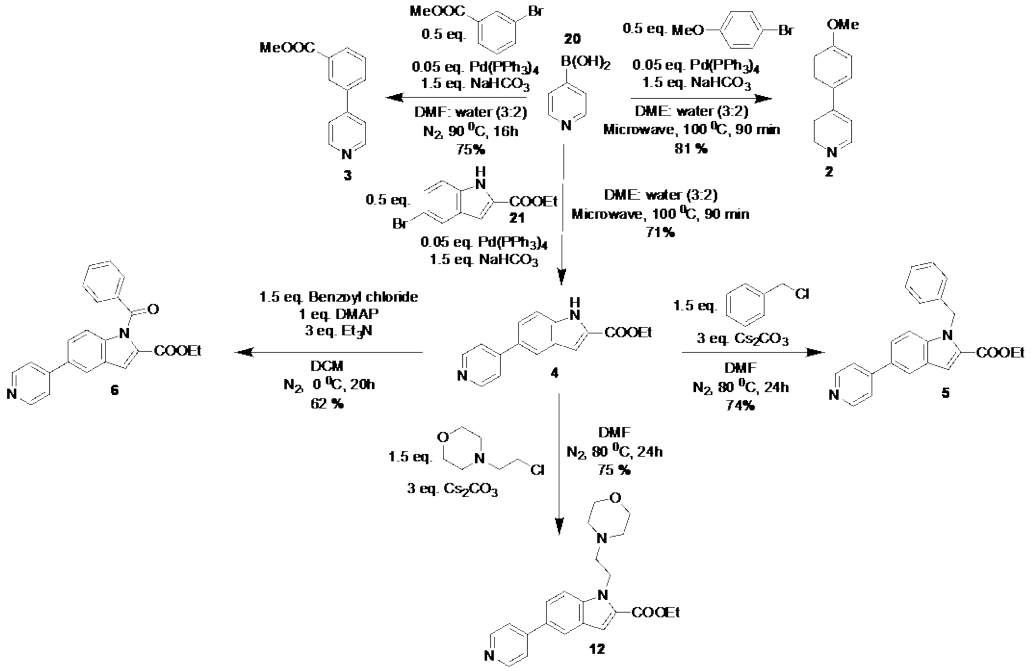

Scheme S1

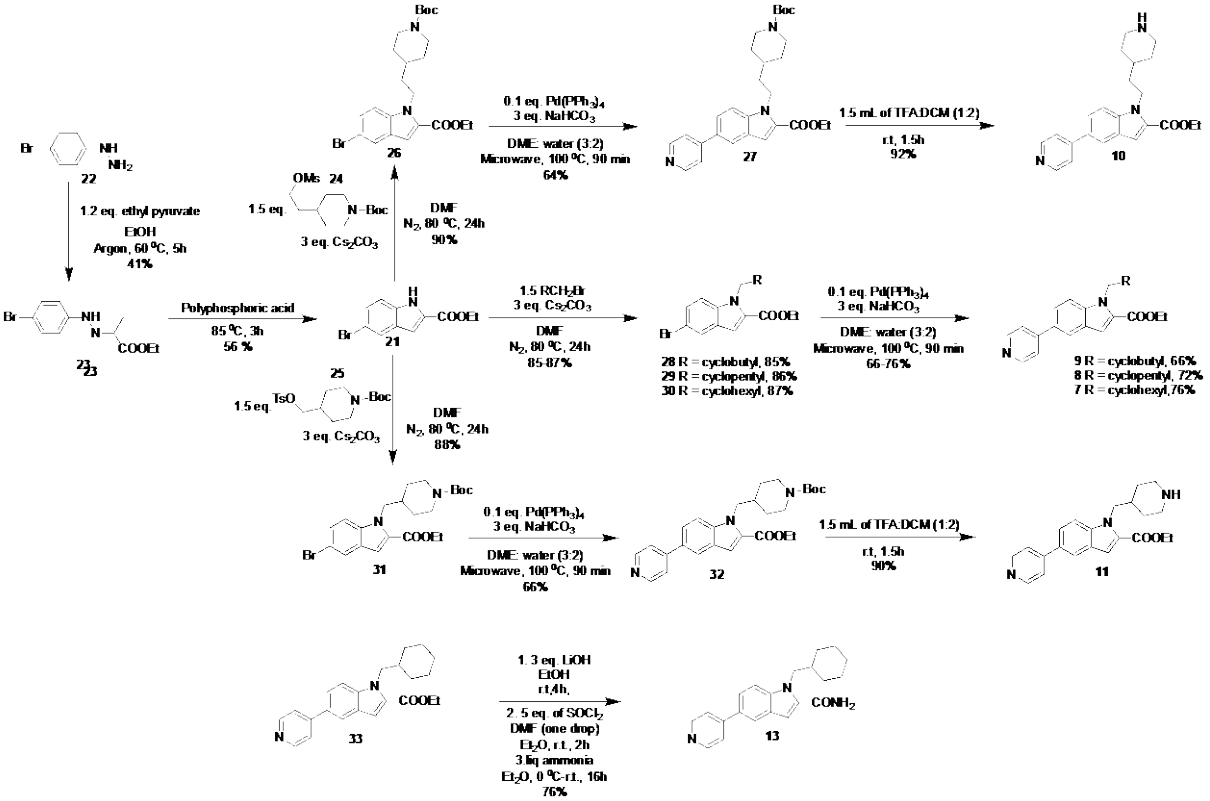

Scheme S2

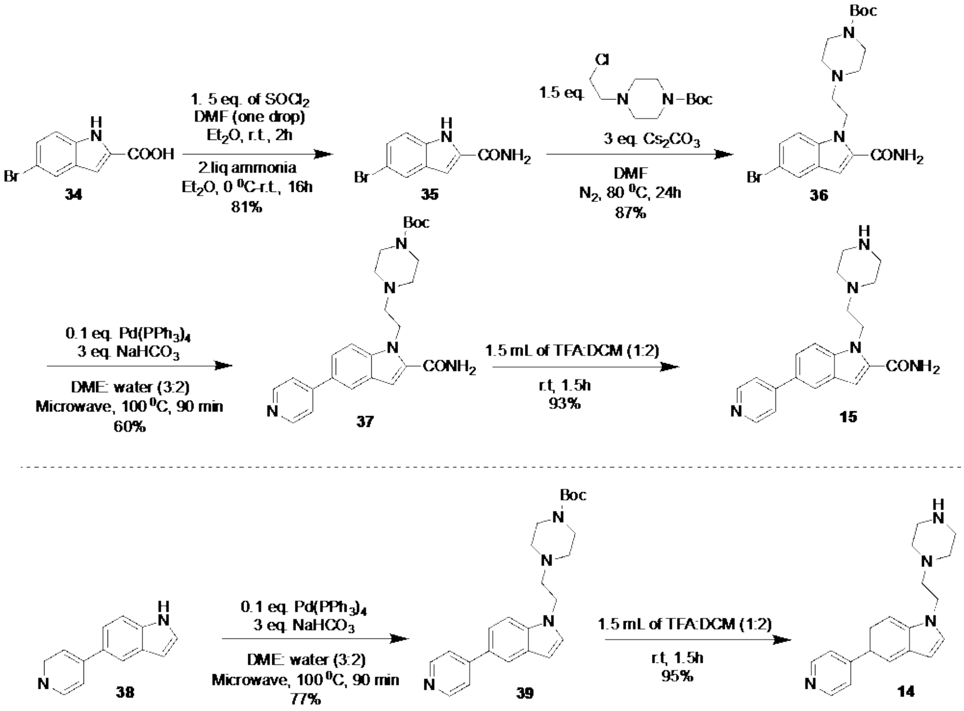

Scheme S3

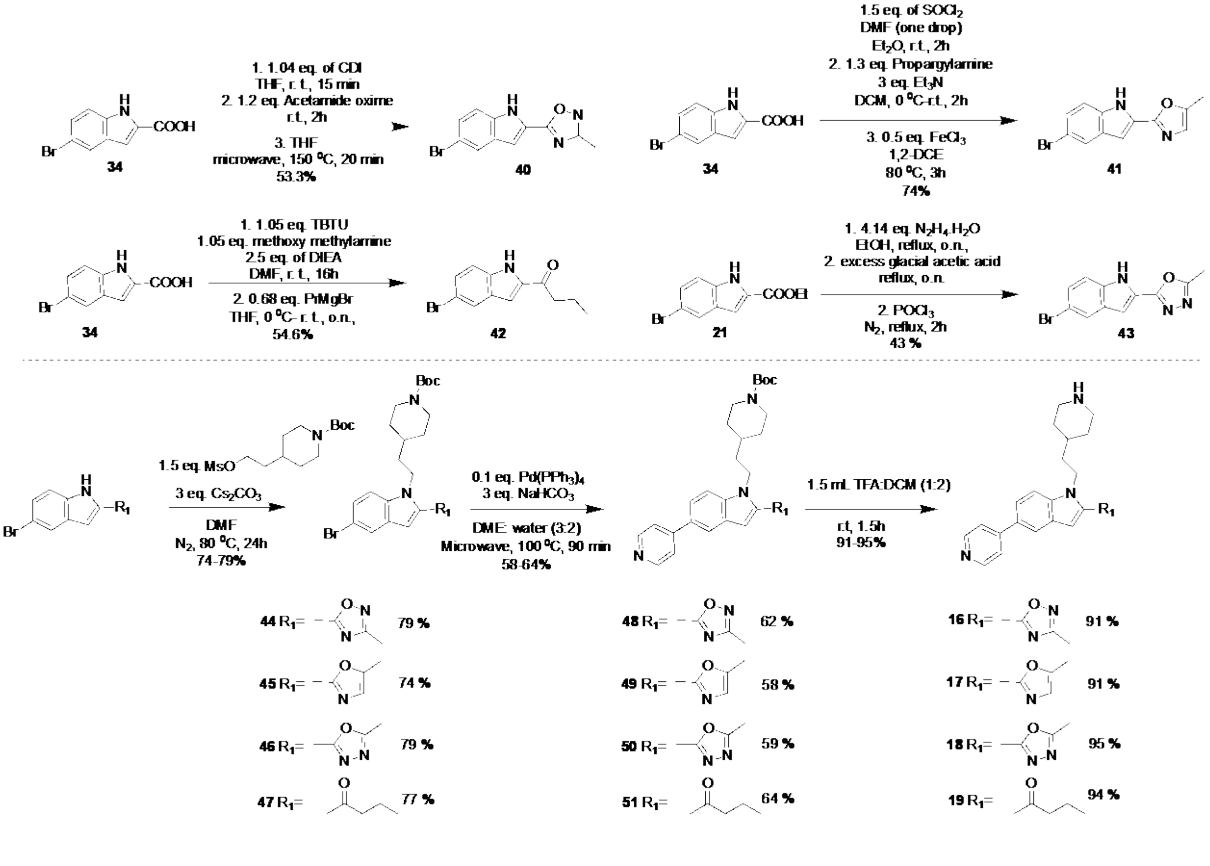

Scheme S4

General Procedures

All non-aqueous reactions were performed under nitrogen atmosphere unless otherwise stated. Water-sensitive reactions were performed in anhydrous solvents in oven-dried glassware cooled under nitrogen before use. Petroleum ether refers to the fraction with a b.p. 40–60 °C, THF refers to tetrahydrofuran and DCM refers to dichloromethane. A rotary evaporator was used to remove the solvents *in vacuo*.

Thin layer chromatography was performed using Merck glass-backed silica (Kieselgel 60 F_{254} 0.25 mm plates). Ultraviolet lamp (λ_{max} = 254 nm) and $KMnO_4$ were used for visualization. Flash column chromatography was performed using an automated Isolera Spektra One/Four purification systems and an appropriately sized Biotage SNAP column containing KP-silica gel (50 μm). Perkin-Elmer One FT-IR spectrometer was used to analyse the infrared spectra. Absorptions are reported in wavenumbers (cm^{-1}).

A SQD2 mass spectrometer detector (Waters) utilising electrospray ionization (ESI) was used for low-resolution mass spectrometry (MS). High-resolution mass spectrometry (HRMS) was recorded using a Waters LCT Premier Time of Flight (TOF) mass spectrometer or a Micromass Quadrapole-Time of Flight (Q-TOF) spectrometer.

The purity of compounds was determined by HPLC and was carried out using an Ultra Performance Liquid Chromatographic system (UPLC) Waters Acquity H-class. All final compounds had purity greater than 95% unless otherwise stated. Samples were detected using a Waters Acquity TUV detector at 2 wavelengths (254 and 280 nm). Samples were run using an Acquity UPLC HSS column and a flow rate of 0.8 mL/min. The eluent consisted of 0.1% formic acid in water (A) and acetonitrile (B); gradient, from 95% A to 5% A over a period of 4 min.

Proton (^{1}H), carbon (^{13}C) and fluorine (^{19}F) NMR data were collected on either a Bruker 400 MHz or 500 Mhz spectrometer. Data were collected at 300 K. Chemical shifts (δ) are given in parts per million (ppm) and they are referenced to the residual solvent peak. Coupling constants (*J*) are reported in Hertz (Hz) and splitting patterns are reported in an abbreviated manner: app. (apparent), s (singlet), d (doublet), t (triplet), q (quartet), m (multiplet), br (broad).

General Procedure 1A: Modified Suzuki-Miyaura Cross Coupling

4-pyridinylboronic acid (5 mmol, 2 equiv.) aryl bromide (2.5 mmol, 1 equiv.) and $Pd(PPh_3)_4$ (0.25 mmol, 0.1 equiv.) and $NaHCO_3$ (7.5 mmol, 3 equiv.) were combined in 50 mL round bottom flask and flushed with N_2 for 5 minutes. A mixture of DMF (6 mL) and water (4 mL) in 3:2 ratio was added and the reaction flask was flushed with N_2 for further 5 minutes. The reaction was heated at 90^{0}C for 16h. The reaction mixture was partitioned between 50mL of EtOAc and 20 mL water. The organic layer was extracted twice with water (10 mL) and brine (10 mL), dried over anhydrous Na_2SO_4 and organic phase was concentrated under reduced pressure. The crude product was purified by silica gel chromatography eluting with 0%-50% v/v EtOAc in hexane.

General Procedure 1B: Modified Suzuki-Miyaura Cross Coupling

4-pyridinylboronic acid (1 mmol, 2 equiv.) aryl bromide (0.5 mmol, 1 equiv.) and $Pd(PPh_3)_4$ (0.05 mmol, 0.1 equiv.) and $NaHCO_3$ (1.5 mmol, 3 equiv.) were combined in a microwave vial and flushed with N_2 for 5 minutes. A mixture of DME (3 mL) and water (2 mL) in 3:2 ratio was added, and the reaction flask was flushed with N_2 for further 5 minutes. The reaction was heated at 100 ^{0}C for 90 minutes. The reaction mixture was diluted with EtOAc (50 mL). The organic layer was extracted thrice with water (10 mL), brine solution (10 mL), and dried over anhydrous Na_2SO_4 and organic phase was concentrated under reduced pressure. The crude product was purified by silica gel chromatography eluting with 0%-80% v/v EtOAc in hexane.

General Procedure 2

An appropriate indole compound (1 mmol, 1 equiv.) and caesium carbonate/potassium carbonate (3 mmol, 3 equiv.) were dissolved in 2 mL of DMF. The appropriate halide/OTs/OMs (1.5 eq) were added. The reaction was placed under nitrogen and heated at 80-90 °C for 24h. The reaction was cooled to room temperature and diluted with water (10 mL) and extracted twice with EtOAc (30 mL). The organic layers were combined, washed with brine solution (30 mL), dried over $MgSO_4$, and concentrated in vacuo to afford a solid. The crude product was purified by silica gel chromatography eluting with 0%-80% v/v EtOAc in hexane to give yield between 73-90 %.

General Procedure 3: Boc deprotection

TFA (0.5 mL) was added to the Boc-protected compound (0.2 mmol) in DCM (1 mL), the mixture was stirred at room temperature for 1.5 h. Reaction progress was monitored by LC-MS. After completion, the volatiles were evaporated *in vacuo*, yellow oil formed was dissolved in DCM (30 mL). The pH of solution (pH =8) was adjusted using saturated $NaHCO_3$ (1N) and then extracted twice in DCM (30 mL). The combined organic layer was washed with brine (40 mL) and dried over anhydrous Na_2SO_4. Solvent was concentrated in vacuo to afford crude compound, which was purified by reverse phase chromatography eluting with 5%-35% v/v ACN in water to give yield between 90-95 %.

Ethyl (E)-2-(2-(4-bromophenyl)hydrazineylidene)propanoate (**23**)[6]

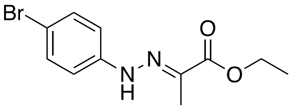

A mixture of 4-bromophenylhydrazine (3 g, 16.03 mmol), ethyl pyruvate (2.23 g, 19.25 mmol) in EtOH was boiled under argon for 5h. The reaction mixture was then filtered, and the precipitate was washed with water. The crude product was triturated with cyclohexane and filtered to give a yellow solid. The crude product was purified by silica gel chromatography eluting with 0%-80% v/v EtOAc in hexane.

Yield (1.88 g, 41%), ^{1}H NMR (400 MHz, $CDCl_3$): δ 7.72 (s, 1H), 7.38 (d, *J* = 8.9 Hz, 2H), 7.08 (d, *J* = 8.9 Hz, 2H), 4.31 (q, *J* = 7.1 Hz, 2H), 2.09 (s, 3H), 1.37 (t, *J* = 7.1 Hz, 3H). ^{13}C NMR (101 MHz, $CDCl_3$): δ 165.1, 142.4, 133.4, 132.2, 115.6, 114.2, 61.4, 14.4, 10.4. LCMS (+ESI): *m/z 285.0* $[M+H]^+$, retention time 2.11 min, (>99%).

Ethyl 5-bromo-1H-indole-2-carboxylate (21)[6]

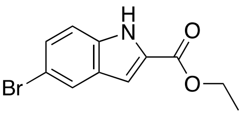

A mixture of ethyl (E)-2-(2-(4-bromophenyl)hydrazineylidene)propanoate **23** (1.88 g, 6.60 mmol) and polyphosphoric acid (18.6 g) was heated to 85 ^{0}C for 3h. The reaction was then cooled, poured into ice-cold water, and neutralised with saturated aq. $NaHCO_3$. The crude product was extracted with and neutralised with EtOAc, dried over Na_2SO_4, filtered,

and concentrated under reduced pressure to give a pale-yellow solid. The crude product was purified by silica gel chromatography eluting with 0%-80% v/v EtOAc in hexane. Yield (0.99 g, 56%) ^{1}H NMR (400 MHz, DMSO-d$_6$): δ 12.05 (s, 1H), 7.87 (d, *J* = 1.7 Hz, 1H), 7.42 (d, *J* = 8.8 Hz, 1H), 7.36 (dd, *J* = 8.8, 1.9 Hz, 1H), 7.12 (s, 1H), 4.34 (q, *J* = 7.1 Hz, 2H), 1.33 (t, *J* = 7.1 Hz, 3H). ^{13}C NMR (101 MHz, DMSO-d$_6$): δ 161.0, 135.9, 128.6, 128.5, 127.2, 124.2, 114.6, 112.6, 107.1, 60.7, 14.3. IR (thin film, v_{max}): 3314, 3008, 2975, 2930, 2897 (m), 1693 (s), 1570, 1521 (s). LCMS (+ESI): *m/z 268.0* [M+H]$^+$, retention time 2.17 min, (97%).

Ethyl 5-bromo-1-(cyclohexylmethyl)-1H-indole-2-carboxylate (**30**)

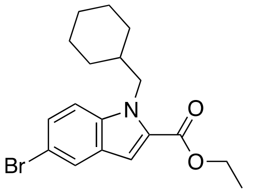

Yield (0.31 g, 87 %), ^{1}H NMR (400 MHz, CDCl$_3$): δ 7.69 (d, *J* = 1.8 Hz, 1H), 7.29 (dd, *J* = 8.9, 1.9 Hz, 1H), 7.17 (d, *J* = 9.0 Hz, 1H), 7.14 (s, 1H), 4.29 (d, *J* = 7.2 Hz, 2H), 4.28 (q, *J* = 7.1 Hz, 2H), 1.75 (dqt, *J* = 14.3, 7.1, 3.4 Hz, 1H), 1.66 – 1.54 (m, 3H), 1.42 (d, *J* = 13.0 Hz, 2H), 1.32 (t, *J* = 7.1 Hz, 3H), 1.06 (d, *J* = 6.5 Hz, 2H), 1.04 – 1.01 (m, 1H), 1.00 – 0.86 (m, 2H). ^{13}C NMR (101 MHz, CDCl$_3$): δ 161.7, 138.1, 128.8, 127.6, 127.3, 124.7, 113.5, 112.6, 109.6, 60.7, 50.8, 39.4, 30.9, 26.3, 25.8, 14.3. IR (thin film, υ_{max}): 2981, 2924, 2852 (m), 1706, 1513 (s). HRMS (+ESI): *m/z* (Calcd. $C_{18}H_{23}BrNO_2$ [M+H]$^+$ = 364.0912), *Obs.* 364.0922 (δ ppm = 2.7) LCMS (+ESI): *m/z 364.2* [M+H]$^+$, retention time 2.90 min, (>99 %).

Ethyl 5-bromo-1-(cyclobutylmethyl)-1H-indole-2-carboxylate (**28**)

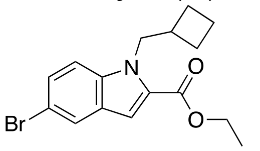

Synthesised according to General Procedure 2, Yield (0.28 g, 85 %), ^{1}H NMR (400 MHz, CDCl$_3$): δ 7.70 (d, *J* = 1.7 Hz, 1H), 7.30 (dd, *J* = 8.9, 1.9 Hz, 1H), 7.22 (d, *J* = 8.9 Hz, 1H), 7.13 (d, *J* = 0.5 Hz, 1H), 4.52 (d, *J* = 7.1 Hz, 2H), 4.29 (q, *J* = 7.1 Hz, 2H), 2.78 – 2.65 (m, 1H), 1.90 – 1.80 (m, 2H), 1.80 – 1.63 (m, 4H), 1.33 (t, *J* = 7.1 Hz, 3H). ^{13}C NMR (101 MHz, CDCl$_3$): δ 161.8, 137.9, 128.6, 127.6, 127.4, 124.8, 113.5, 112.4, 109.6, 60.7, 49.2, 36.7, 26.2, 18.4, 14.4. IR (thin film υ_{max}): 2977, 2936, 2865 (m), 1709, 1513 (s). HRMS (+ESI): *m/z* (Calcd. $C_{16}H_{19}BrNO_2$ [M+H]$^+$ = 336.0594), *Obs.* 336.0593 (δ ppm = -0.2); LCMS (+ESI): *m/z 336.1* [M+H]$^+$, retention time 2.73 min, (>99%).

Ethyl 5-bromo-1-(cyclopentylmethyl)-1H-indole-2-carboxylate (29)

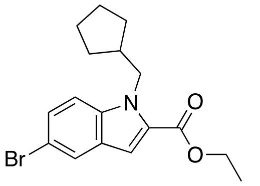

Synthesised according to General Procedure 2, Yield (0.30 g, 86 %), ^{1}H NMR (400 MHz, CDCl$_3$): δ 7.71 (d, *J* = 1.8 Hz, 1H), 7.30 (dd, *J* = 8.9, 1.9 Hz, 1H), 7.21 (d, *J* = 8.9 Hz, 1H), 7.15 (s, 1H), 4.43 (d, *J* = 7.6 Hz, 2H), 4.29 (q, *J* = 7.1 Hz, 2H), 2.37 – 2.24 (m, 1H), 1.65 – 1.53 (m, 2H), 1.53 – 1.46 (m, 2H), 1.46 – 1.37 (m, 2H), 1.33 (t, *J* = 7.1 Hz, 3H), 1.28 – 1.12 (m, 2H). ^{13}C NMR (101 MHz, CDCl$_3$): δ 161.9, 137.9, 128.7, 127.6, 127.4, 124.8, 113.5, 112.5, 109.7, 60.7, 49.1, 41.6, 30.3, 24.8, 14.4. IR (thin film, υ_{max}): 2952, 2868, 1707, 1512 (s). HRMS (+ESI): *m/z* (Calcd. $C_{17}H_{21}BrNO_2$ [M+H]$^+$ = 350.0756), *Obs.* 350.0769 (δ ppm = 3.7) LCMS (+ESI): *m/z 350.1* [M+H]$^+$, retention time 2.81 min, (>99%).

tert-butyl 4-((tosyloxy)methyl)piperidine-1-carboxylate (25)

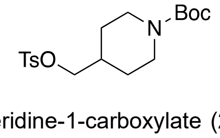

To a mixture of tert-butyl 4-(hydroxymethyl)piperidine-1-carboxylate (2.15 g, 10 mmol), DMAP (60 mg, 0.49 mmol) in pyridine (6 mL, 74.4 mmol) cooled to 0-5 ^{0}C, tosyl chloride (1.99 g, 1.05 mmol) was added in portion over a period of 15 mins. After addition the reaction mixture was stirred at room temperature for 16h and then poured into ice water (150 mL). The resulting mixture was extracted twice with EtOAc (120 mL), combined organic extracts was washed thrice with dilute hydrochloride acid (1N, 100 mL), saturated $NaHCO_3$ (80 mL) and dried over anhydrous Na_2SO_4, filtered, and evaporated. 20 mL pet ether was added and solid formed, the mixture was stirred vigorously for 20 mins, the forming precipitation was filtered under reduced pressure to give a white solid, which was employed in the subsequent step without further purification, Yield (3.37 g, 91%),

^{1}H NMR (400 MHz, $CDCl_3$): δ 7.79 – 7.69 (m, 2H), 7.38 – 7.28 (m, 2H), 4.05 (br s, 2H), 3.89 – 3.74 (m, 2H), 2.82 – 2.50 (m, 2H), 2.48 – 2.34 (m, 3H), 1.91 – 1.70 (m, 1H), 1.61 (d, *J* = 12.6 Hz, 2H), 1.47 – 1.34 (m, 9H), 1.07 (q, *J* = 12.3 Hz, 2H). ^{13}C NMR (101 MHz, $CDCl_3$): δ 154.6, 144.9, 132.8, 129.8, 127.8, 79.4, 73.9, 43.2 (br), 35.7, 28.4, 28.1, 21.6. IR (thin film, υ_{max}): 2979, 2952, 2911, 2854 (m), 1692, 1598 (s). LCMS (+ESI): *m/z 370.2* $[M+H]^+$, retention time 2.26 min, (>99%).

Ethyl-5-bromo-1-((1-(tert-butoxycarbonyl)piperidin-4-yl)methyl)-1H-indole-2-carboxylate (**31**)

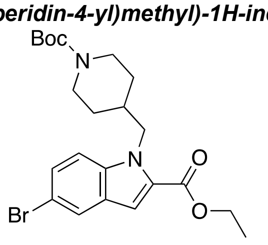

Synthesised according to General Procedure 2, Yield (0.41 g, 88%), ^{1}H NMR (400 MHz, $CDCl_3$) δ 7.72 (d, *J* = 1.7 Hz, 1H), 7.32 (dd, *J* = 9.0, 1.8 Hz, 1H), 7.17 (s, 1H), 7.17 (d, *J* = 8.8 Hz, 1H), 4.35 (d, *J* = 7.2 Hz, 2H), 4.30 (q, *J* = 7.1 Hz, 2H), 4.01 (s, 2H), 2.50 (t, *J* = 11.8 Hz, 2H), 2.01 – 1.87 (m, 1H), 1.44 – 1.34 (m, 2H, overlap Boc peak), 1.37 (s, 9H), 1.34 (t, *J* = 7.1 Hz, 3H), 1.17 (qd, *J* = 12.3, 3.7 Hz, 2H).. ^{13}C NMR (101 MHz, $CDCl_3$) δ 161.8, 154.7, 138.1, 128.6, 127.9, 127.3, 124.9, 113.7, 112.3, 109.9, 79.4, 60.8, 50.0, 43.5, 38.0, 29.9, 28.5, 14.4 (br). IR (thin film, υ_{max}): 2979, 2934, 2856 (m), 1717, 1673, 1515 (s). HRMS (+ESI): *m/z* (Calcd. $C_{22}H_{29}BrN_2O_4Na$ $[M+Na]^+$ = *487.1203*), *Obs.* 487.1188 (δ ppm = -3.1) LCMS (+ESI): *m/z 464.2* $[M+H]^+$, retention time 3.72 min, (>99%).

Ethyl-1-((1-(tert-butoxycarbonyl)piperidin-4-yl)methyl)-5-(pyridin-4-yl)-1H-indole-2-carboxylate (32)

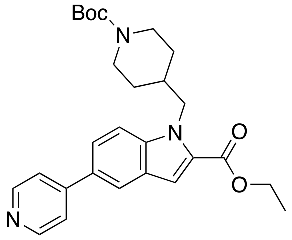

Synthesised according to General Procedure 1B, Yield (0.15 g, 66 %), ^{1}H NMR (500 MHz, $CDCl_3$) δ 8.58 (d, *J* = 6.1 Hz, 2H), 7.89 (d, *J* = 1.5 Hz, 1H), 7.56 (dd, *J* = 8.8, 1.8 Hz, 1H), 7.49 (d, *J* = 6.1 Hz, 2H), 7.41 (d, *J* = 8.8 Hz, 1H), 7.34 (s, 1H), 4.43 (d, *J* = 7.1 Hz, 2H), 4.33 (q, *J* = 7.1 Hz, 2H), 4.14 – 3.92 (m, 2H), 2.68 – 2.40 (m, 2H), 2.07 – 1.92 (m, 1H), 1.45 (d, *J* = 13.4 Hz, 2H), 1.38 (s, 9H), 1.36 (t, *J* = 7.1 Hz, 3H), 1.28 – 1.20 (m, 2H). ^{13}C NMR (126 MHz, $CDCl_3$) δ 161.9, 154.8, 150.2, 148.9, 139.8, 130.9, 128.8, 126.4, 124.1, 121.7, 121.3, 111.6, 111.4, 79.5, 60.9, 50.1, 43.5, 38.1, 30.0, 28.5, 14.4. IR (thin film, υ_{max}): 3000-2927, 2855 (m), 1687, 1596, 1525 (s). HRMS (+ESI): *m/z* (Calcd. $C_{27}H_{34}N_3O_4$ $[M+H]^+$ = 464.2543), *Obs.* 464.2551 (δ ppm = 1.6) LCMS (+ESI): *m/z 464.3* $[M+H]^+$, retention time 2.06 min, (>95%).

tert-butyl 4-(2-((methylsulfonyl)oxy)ethyl)piperidine-1-carboxylate (**24**)

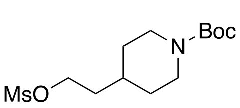

To a stirred solution of tert-butyl-4-(2-hydroxyethyl)piperazine-1-carboxylate (0.50 g, 2.15 mmol) and triethylamine (0.45 mL, 3.23 mmol) in DCM (7 mL) was added methanosulfonyl chloride (0.20 mL , 2.58 mmol) dropwise at 0 ^{0}C. The mixture was allowed to warm to room temperature and stirred for 3 h. The organic phase was diluted with DCM (40 mL), washed with water (10 mL) and brine (10 mL) and dried over anhydrous Na_2SO_4. Solvent was evaporated using rotatory evaporated below 35 ^{0}C and the crude product was precipitated using pet ether (20 mL), filtered to give a yellowish solid product, which was employed in the subsequent step without further purification. Yield (0.64 g, 97 %), ^{1}H NMR (500 MHz, $CDCl_3$): δ 4.22 (t, *J* = 6.4 Hz, 2H), 4.13 – 3.92 (m, 2H), 2.95 (s, 3H), 2.71 – 2.54 (m, 2H), 1.65 – 1.59 (m, 4H), 1.58 – 1.52 (m, 1H), 1.38 (s, 9H), 1.07 (qd, *J* = 12.6, 4.4 Hz, 2H). ^{13}C NMR (126 MHz, $CDCl_3$): δ 154.8, 79.4, 67.5, 46.1, 37.4, 35.6, 32.4, 31.8, 28.5. IR (thin film, υ_{max}): 3009, 2976, 2932, 2849 (m), 1666, 1534 (s).

Ethyl-5-bromo-1-(2-(1-(tert-butoxycarbonyl)piperidin-4-yl)ethyl)-1H-indole-2-carboxylate (**26**)

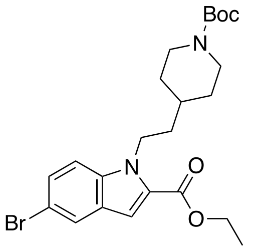

Synthesised according to General Procedure 2, Yield (0.43 g, 90 %), ^1H NMR (400 MHz, $CDCl_3$): δ 7.73 (d, *J* = 1.8 Hz, 1H), 7.33 (dd, *J* = 8.8, 1.9 Hz, 1H), 7.18 (d, *J* = 8.1 Hz, 1H), 7.14 (s, 1H), 4.60 – 4.41 (m, 2H), 4.30 (q, *J* = 7.1 Hz, 2H), 4.16 – 3.88 (m, 2H), 2.62 (t, *J* = 11.9 Hz, 2H), 1.74 – 1.59 (m, 4H), 1.52 – 1.41 (m, 1H), 1.40 (s, 9H), 1.34 (t, *J* = 7.1 Hz, 3H), 1.13 (qd, *J* = 12.5, 4.0 Hz, 2H). ^{13}C NMR (101 MHz, $CDCl_3$): δ 161.7, 154.8, 137.3, 128.3, 127.9, 127.5, 125.0, 113.7, 111.7, 109.6, 79.3, 60.8, 42.7, 37.0, 34.1, 32.1, 32.1, 28.5, 14.4. IR (thin film, υ_{max}): 2974, 2930, 2852 (m), 1704, 1682, 1511 (s). HRMS (+ESI): *m/z* (Calcd. $C_{23}H_{31}BrN_2O_4Na$ $[M+H]^+$ = 501.1360), *Obs.* 501.1362 (δ ppm = 0.4) LCMS (+ESI): *m/z 479.2* $[M+H]^+$, retention time 2.82 min, (>99%).

Ethyl-1-(2-(1-(tert-butoxycarbonyl)piperidin-4-yl)ethyl)-5-(pyridin-4-yl)-1H-indole-2-carboxylate (**27**)

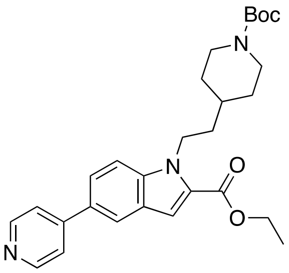

Synthesised according to General Procedure 1B, Yield (0.15 g, 64 %), ^1H NMR (500 MHz, $CDCl_3$): δ 8.58 (d, *J* = 6.1 Hz, 2H), 7.89 (d, *J* = 1.3 Hz, 1H), 7.56 (dd, *J* = 8.8, 1.8 Hz, 1H), 7.49 (d, *J* = 6.2 Hz, 2H), 7.39 (d, *J* = 8.8 Hz, 1H), 7.30 (d, *J* = 0.6 Hz, 1H), 4.63 – 4.51 (m, 2H), 4.32 (q, *J* = 7.1 Hz, 2H), 4.15 – 3.92 (m, 2H), 2.64 (t, *J* = 10.6 Hz, 2H), 1.71 (d, *J* = 6.8 Hz, 2H), 1.68 (d, *J* = 6.9 Hz, 2H), 1.52 – 1.44 (m, 1H), 1.39 (s, 9H), 1.36 (t, *J* = 7.1 Hz, 3H), 1.16 (qd, *J* = 12.2, 3.9 Hz, 2H). ^{13}C NMR (126 MHz, $CDCl_3$): δ 161.8, 154.9, 150.2, 148.9, 139.1, 130.8, 128.5, 126.5, 124.1, 121.7, 121.4, 111.0, 110.9, 79.4, 60.8, 42.8, 37.1, 34.2, 32.1, 32.1, 28.5, 14.4. IR (thin film, υ_{max}): 2977, 2926, 2854 (m), 1704, 1685, 1617, 1595, 1524 (s). HRMS (+ESI): *m/z* (Calcd. $C_{28}H_{36}N_3O_4$ $[M+H]^+$ = 478.2701), *Obs.* 478.2702 (δ ppm = 0.3) LCMS (+ESI): *m/z 479.3* $[M+H]^+$, retention time 2.21 min, (>99 %).

tert-butyl 4-(2-(5-(pyridin-4-yl)-1H-indol-1-yl)ethyl)piperazine-1-carboxylate (39)

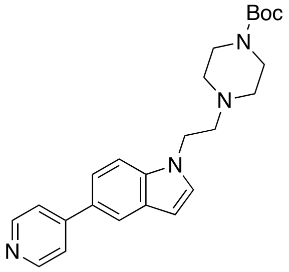

Synthesised according to General Procedure 2, Yield (0.31 g, 77 %), ^1H NMR (500 MHz, $CDCl_3$): δ 8.55 (d, *J* = 6.2 Hz, 2H), 7.86 (d, *J* = 1.5 Hz, 1H), 7.51 (d, *J* = 6.2 Hz, 2H), 7.44 (dd, *J* = 8.6, 1.7 Hz, 1H), 7.37 (d, *J* = 8.6 Hz, 1H), 7.14 (d, *J* = 3.1 Hz, 1H), 6.51 (d, *J* = 3.1 Hz, 1H), 4.21 (t, *J* = 6.8 Hz, 2H), 3.35 (t, *J* = 3.9 Hz, 4H), 2.72 (t, *J* = 6.8 Hz, 2H), 2.37 (t, *J* = 3.9 Hz, 4H), 1.39 (s, 9H).^{13}C NMR (126 MHz, $CDCl_3$): δ 154.7, 149.9, 149.8, 136.4, 129.5, 129.3, 129.2, 121.8, 120.7, 119.9, 109.9, 102.2, 79.8, 57.9, 53.3, 44.5, 28.5. IR (thin film, υ_{max}): 2970, 2921 (m), 1686, 1594,1545, 1515 (s). HRMS (+ESI): *m/z* (Calcd. $C_{24}H_{31}N_4O_2$ $[M+H]^+$ = 407.2441), *Obs.* 407.2441 (δ ppm = 0.0) LCMS (+ESI): *m/z 407.3* $[M+H]^+$, retention time 1.81 min (Purity >92% by NMR).

5-Bromo-1H-indole-2-carboxamide (**35**)

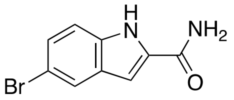

DMF (one drop) and thionyl chloride (1.55 mL, 20.84 mmol) were added to a solution of 5-bromo-1H-indole-2-carboxylic acid **34** (1.00 g, 4.16 mmol) in diethyl ether (40 mL) and reaction was stirred at room temperature for 2 h. The volatiles were removed, and the crude acyl chloride was dissolved in diethyl ether (40 mL) and added to liquid ammonia at -60 ^{0}C. The mixture was slowly warmed to room temperature and was stirred for 12 h. The mixture was diluted with EtOAc (50 mL) and washed with water (20 mL), saturated $NaHCO_3$ (20 mL), water (20 mL) and brine (20 mL), dried over anhydrous Na_2SO_4 and concentrated to give a crude white solid. Yield (0.81 g, 81 %),
^{1}H NMR (400 MHz, DMSO-d$_6$) δ 11.68 (s, 1H), 8.02 (s, 1H), 7.59 (s, *J* = 8.8 Hz, 1H), 7.57 (s, 1H), 7.42 (s, 1H), 7.15 (dd, *J* = 8.8, 1.6 Hz, 1H), 7.13 (d, *J* = 1.2 Hz, 1H). ^{13}C NMR (126 MHz, $CDCl_3$): ^{13}C NMR (101 MHz, DMSO-d$_6$) δ 162.4, 137.2, 132.7, 126.2, 123.4, 122.6, 116.0, 114.7, 103.1. IR (thin film, υ_{max}): 3427, 3381, 3165, 1646, 1611, 1528 (s). LCMS (+ESI): *m/z 407.3* [M+H]$^+$, retention time 1.81 min. (Purity >91% by NMR).

tert-Butyl-4-(2-(5-bromo-2-carbamoyl-1H-indol-1-yl)ethyl)piperazine-1-carboxylate (**36**)

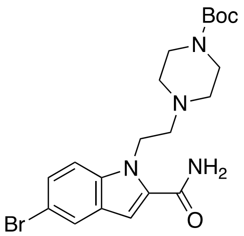

Synthesised according to General Procedure 2, Yield (0.39 g, 87%),^{1}H NMR (400 MHz, DMSO-d$_6$): δ 8.02 (s, 1H), 7.83 (d, *J* = 1.7 Hz, 1H), 7.54 (d, *J* = 8.9 Hz, 1H), 7.41 (s, 1H), 7.35 (dd, *J* = 8.8, 1.8 Hz, 1H), 7.04 (s, 1H), 4.66 (t, *J* = 6.3 Hz, 2H), 3.23 (t, *J* = 4.2 Hz, 4H), 2.54 (t, *J* = 6.5 Hz, 2H), 2.31 (t, *J* = 4.2 Hz, 4H), 1.38 (s, 9H). ^{13}C NMR (101 MHz, DMSO-d$_6$): δ 163.4, 153.8, 136.5, 133.3, 127.4, 125.8, 123.64, 112.9, 112.4, 104.5, 78.7, 57.7, 52.8, 41.4, 28.0. IR (thin film, υ_{max}): 3374, 3165 (b), 2968, 2918, 2851, 2813 (m), 1664, 1620, 1519 (s). HRMS (+ESI): *m/z* (Calcd. $C_{20}H_{28}BrN_4O_3$ [M+H]$^+$ = 451.1339), *Obs.* 451.1341 (δ ppm = 0.4) LCMS (+ESI): *m/z 451.2* [M+H]$^+$, retention time 1.2 min, (98%).

tert-Butyl-4-(2-(2-carbamoyl-5-(pyridin-4-yl)-1H-indol-1-yl)ethyl)piperazine-1-carboxylate (**37**)

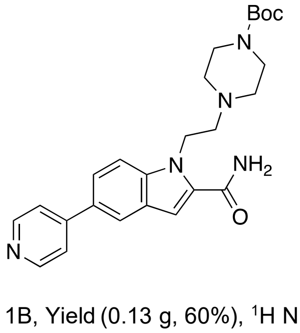

Synthesised according to General Procedure 1B, Yield (0.13 g, 60%), ^{1}H NMR (500 MHz, $CDCl_3$): δ 8.58 (d, *J* = 6.1 Hz, 2H), 7.87 (d, *J* = 1.3 Hz, 1H), 7.55 (dd, *J* = 8.7, 1.8 Hz, 1H), 7.49 (d, *J* = 6.2 Hz, 2H), 7.45 (d, *J* = 8.7 Hz, 1H), 6.95 (s, 1H), 4.66 (t, *J* = 6.7 Hz, 2H), 3.32 (t, *J* = 3.6 Hz, 4H), 2.71 (t, *J* = 6.6 Hz, 2H), 2.39 (t, *J* = 3.6 Hz, 4H), 1.38 (s, 9H). ^{13}C NMR (126 MHz, $CDCl_3$): δ 163.7, 154.7, 150.2, 148.9, 138.9, 132.1, 131.0, 126.8, 123.7, 121.7, 120.7, 111.1, 106.3, 79.8, 58.0, 42.7, 28.5. IR (thin film, υ_{max}): 3329, 3171 (b), 2917, 2849 (s), 1739, 1669, 1597 (s). HRMS (+ESI): *m/z* (Calcd. $C_{25}H_{32}N_5O_3$ [M+H]$^+$ = 450.2500), *Obs.* 450.2517 (δ ppm = 3.8) LCMS (+ESI): *m/z 450.3* [M+H]$^+$, retention time 0.93 min, (98%).

5-(5-Bromo-1H-indol-2-yl)-3-methyl-1,2,4-oxadiazole (**40**)

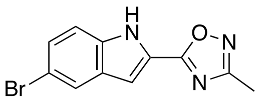

To a solution of 5-bromo-2-indolecarboxylic acid **34** (3.00 g, 12.6 mmol) in THF (20 mL) was added carbonyldiimidazole (CDI) (2.16 g, 13.2 mmol) and the mixture was stirred for 15 minutes at room temperature. Acetamide Oxime (1.14 g, 15 mmol) was then added to the mixture and the mixture was stirred for another 2 h. The THF was evaporated *in vacuo* and crude was extracted twice with EtOAc (80 mL), washed with aq. HCl (1N, 30 mL). The combined organic layer was washed with brine solution (50 mL), dried over anhydrous Na_2SO_4 and concentrated to give a crude yellowish solid, which was employed in the subsequent step without further purification. (LC/MS ESI m/z $(M+H)^+$=296.2 (>99%))

The crude (N-((5-bromo-1H-indole-2-carbonyl)oxy)acetimidamide) was suspended in THF and was heated at 150 ^{0}C in microwave reactor for 20 minutes. The solvent was evaporated and residue was triturated in EtOAc (20 mL) and filtered *in vacuo* to give 5-(5-bromo-1H-indol-2-yl)-3-methyl-1,2,4-oxadiazole. The crude product was purified by silica gel chromatography eluting with 0-50% v/v EtOAc in hexane.

Yield (1.87g, 53%), ^{1}H NMR (500 MHz, $CDCl_3$): δ 8.99 (s, 1H), 7.83 – 7.78 (m, 1H), 7.37 (dd, *J* = 8.7, 1.9 Hz, 1H), 7.27 (dt, *J* = 8.8, 0.9 Hz, 1H), 7.24 (dd, *J* = 2.1, 0.9 Hz, 1H), 2.41 (s, 3H). ^{13}C NMR (126 MHz, $CDCl_3$): δ 168.2, 166.5, 134.8, 128.4, 127.7, 123.7, 121.5, 113.5, 112.2, 106.2, 10.5. IR (thin film, υ_{max}): 3200-3100 (b), 2931, 2885 (m), 1601, 1565, 1545 (s). HRMS (+ESI): *m/z* (Calcd. $C_{11}H_9BrN_3O$ $[M+H]^+$ = 277.9923), *Obs.* 277.9928 (δ ppm = 1.7) LCMS (+ESI): *m/z 278.0* $[M+H]^+$, retention time 2.08 min, (>99%).

tert-Butyl-4-(2-(5-bromo-2-(3-methyl-1,2,4-oxadiazol-5-yl)-1H-indol-1-yl)ethyl)piperidine-1-carboxylate (**44**)

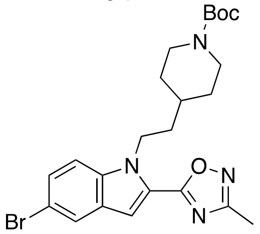

Synthesised according to General Procedure 2, Yield (0.38g, 79%), ^{1}H NMR (400 MHz, $CDCl_3$): δ 7.76 (d, *J* = 1.6 Hz, 1H), 7.35 (dd, *J* = 8.9, 1.8 Hz, 1H), 7.26 (s, 1H), 7.19 (d, *J* = 8.9 Hz, 1H), 4.60 (t, *J* = 8.0 Hz, 2H), 4.17 – 3.82 (m, 2H), 2.63 (t, *J* = 12.0 Hz, 2H), 2.41 (s, 3H), 1.65 (dd, *J* = 15.4, 7.0 Hz, 4H), 1.47 (dddd, *J* = 14.6, 10.9, 7.0, 3.6 Hz, 1H), 1.38 (s, 9H), 1.14 (qd, *J* = 12.4, 3.8 Hz, 2H). ^{13}C NMR (101 MHz, $CDCl_3$): δ 169.3, 167.5, 154.8, 137.4, 128.4, 128.1, 124.8, 123.6, 114.2, 111.7, 108.3, 79.4, 43.7, 43.0, 36.6, 34.0, 32.0, 28.5, 11.8. IR (thin film, υ_{max}): 2932, 2847 (m), 1686, 1606, 1540 (s). HRMS (+ESI): *m/z* (Calcd. $C_{23}H_{29}BrN_4O_3Na$ $[M+Na]^+$ = 511.1315), *Obs.* 511.1313 (δ ppm = -0.4), LCMS (+ESI): *m/z 489.1* $[M+H]^+$, retention time 2.75 min, (>99%).

tert-Butyl-4-(2-(2-(3-methyl-1,2,4-oxadiazol-5-yl)-5-(pyridin-4-yl)-1H-indol-1-yl)ethyl)piperidine-1-carboxylate (**48**)

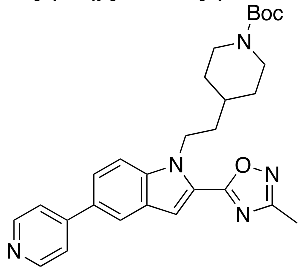

Synthesised according to General Procedure 1B, Yield (0.15g, 62%), ^{1}H NMR (500 MHz, $CDCl_3$): δ 8.61 (d, *J* = 5.2 Hz, 2H), 7.94 (d, *J* = 1.4 Hz, 1H), 7.59 (dd, *J* = 8.9, 1.7 Hz, 1H), 7.52 (d, *J* = 6.0 Hz, 2H), 7.43 (d, *J* = 8.9 Hz, 1H), 7.43 (s, 1H), 4.76 – 4.63 (m, 2H), 4.12 – 3.93 (m, 2H), 2.65 (t, *J* = 10.7 Hz, 2H), 2.44 (s, 3H), 1.71 (dd, *J* = 15.6, 6.8 Hz, 4H), 1.39 (s, 9H), 1.24 – 1.11 (m, 2H). ^{13}C NMR (126 MHz, $CDCl_3$): δ 168.3, 166.5, 153.8, 149.1, 138.1, 130.1, 126.4, 123.2, 122.7, 120.7, 120.2, 110.0, 108.6, 78.4, 43.1, 42.0, 35.7, 33.0, 30.0, 27.4, 10.8. One of the aromatic carbon also overlap with other aromatic carbon. IR (thin film, υ_{max}): 2973,2924,2851 (m), 1686, 1607, 1596 (s). HRMS (+ESI): *m/z* (Calcd. $C_{28}H_{34}N_5O_3$ $[M+H]^+$ = 488.2656), *Obs.* 488.2664 (δ ppm = 1.6) LCMS (+ESI): *m/z 488.2* $[M+H]^+$, retention time 2.15 min, (>99%).

2-(5-Bromo-1H-indol-2-yl)-5-methyloxazole (41)

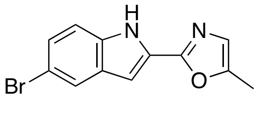

DMF (one drop) and thionyl chloride (1.55 mL, 20.8 mmol) were added to a solution of 5-bromo-1H-indole-2-carboxylic acid **34** (1.00 g, 4.16 mmol) in diethyl ether (40 mL) and reaction was stirred at room temperature for 2 h. Volatiles were removed *in vacuo*, resulting acid chloride was dissolved in DCM (3 mL) and was then added to a cooled solution of

propargyl amine (0.34 mL, 5.42 mmol) in DCM (3 mL) and triethylamine (2.1 mL, 20.8 mmol) and the resulting mixture was allowed to reach room temperature. The completion of the reaction was monitored by the disappearance of acid chloride by TLC and appearance of 5-bromo-N-(prop-2-yn-1-yl)-1H-indole-2-carboxamide. (LC/MS ESI m/z $(M+H)^+$=277.2 (87%)).

The mixture was diluted with water (50 mL). The aqueous layer was extracted three times with DCM (30mL), and the combined organic layers were washed saturated $NaHCO_3$ (30 mL) followed by water (30 mL) and brine (30 mL), dried over Na_2SO_4 and concentrated *in vacuo* to obtain the crude 5-bromo-N-(prop-2-yn-1-yl)-1H-indole-2-carboxamide. The crude product was dissolved in 1,2-DCE (5 mL) followed by the addition of $FeCl_3$ (0.43 g, 2.71 mmol) and stirred at 80^0 C for 3h till the completion of the reaction by LC-MS. The reaction mixture was diluted with water (30 mL). The aqueous layer was extracted thrice with DCM (30 mL), and the combined organic layers were washed with water (20 mL) and brine (20 mL), dried over Na_2SO_4 and concentrated *in vacuo*. The crude product was purified by silica gel chromatography eluting with 0%-80% v/v EtOAc in hexane.

Yield (1.11g, 74%), ^1H NMR (400 MHz, $CDCl_3$): δ 9.27 (s, 1H), 7.72 (s, 1H), 7.26 (dd, *J* = 8.7, 1.7 Hz, 1H), 7.21 (d, *J* = 8.7 Hz, 1H), 6.92 (d, *J* = 1.2 Hz, 1H), 6.78 (s, 1H), 2.35 (s, 3H). ^{13}C NMR (101 MHz, $CDCl_3$): δ 149.3, 135.3, 130.0, 126.8, 126.8, 123.9, 123.9, 113.7, 112.8, 102.3, 11.1. one of the quaternary carbons overlap other peaks. IR (thin film, υ_{max}): 3114, 3062, 3010, 2919, 2871 (m), 1639, 1608, 1561, 1506 (s). HRMS (+ESI): *m/z* (Calcd. $C_{12}H_9BrN_2O$ $[M+H]^+$ = 276.9971), *Obs.* 276.9973 (δ ppm = 0.9) LCMS (+ESI): *m/z 277.0* $[M+H]^+$, retention time 2.13 min, (>99%).

tert-Butyl-4-(2-(5-bromo-2-(5-methyloxazol-2-yl)-1H-indol-1-yl)ethyl)piperidine-1-carboxylate (45)

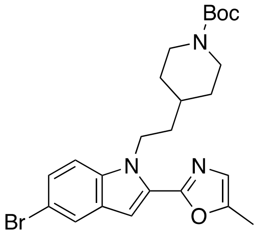

Synthesised according to General Procedure 2, Yield (0.36g, 74%), ^1H NMR (400 MHz, $CDCl_3$): 7.79 (d, *J* = 1.8 Hz, 1H), 7.37 (dd, *J* = 8.8, 1.9 Hz, 1H), 7.25 (d, *J* = 8.8 Hz, 1H, overlap with $CDCl_3$), 7.05 (s, 1H), 6.88 (q, *J* = 1.2 Hz, 1H), 4.74 (t, *J* = 7.9 Hz, 2H), 2.70 (s, 1H), 2.44 (d, *J* = 1.2 Hz, 3H), 1.74 (q, *J* = 7.1 Hz, 4H), 1.47 (s, overlap with H_2O), 1.21 (d, *J* = 12.9 Hz, 1H). ^{13}C NMR (126 MHz, $CDCl_3$) δ 154.9, 154.8, 148.6, 136.6, 128.9, 127.7, 126.1, 124.1, 123.9, 113.5, 111.3, 103.5, 79.4, 43.7 (br), 42.7, 36.5, 34.0, 32.1, 28.5, 11.0. IR (thin film, υ_{max}): 2923, 2847 (m), 1684, 1608 (s). HRMS (+ESI): *m/z* (Calcd. $C_{24}H_{30}BrN_3O_3Na$ $[M+Na]^+$ = 510.1362), *Obs.* 510.1363 (δ ppm = 0.1) LCMS (+ESI): *m/z 488.1* $[M+H]^+$, retention time 2.8 min, (>95%).

tert-butyl-4-(2-(2-(5-methyloxazol-2-yl)-5-(pyridin-4-yl)-1H-indol-1-yl)ethyl)piperidine-1-carboxylate (49)

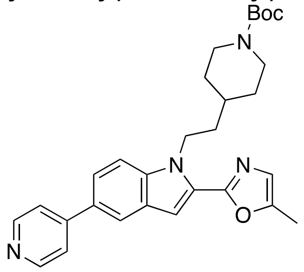

Synthesised according to General Procedure 1B, Yield (0.14g, 58%), ^1H NMR (500 MHz, DMSO-d_6): δ 8.54 (d, *J* = 6.1 Hz, 2H), 8.06 – 8.03 (m, 1H), 7.68 (d, *J* = 6.2 Hz, 2H), 7.66 – 7.62 (m, 2H), 7.11 (s, 1H), 7.04 (d, *J* = 1.2 Hz, 1H), 4.77 – 4.66 (m, 2H), 3.83 (d, *J* = 9.9 Hz, 2H), 2.73 – 2.50 (m, 2H), 2.35 (d, *J* = 1.2 Hz, 3H), 1.64 (d, *J* = 12.3 Hz, 2H), 1.57 (dd, *J* = 15.1, 6.9 Hz, 2H), 1.51 – 1.40 (m, 1H), 1.32 (s, 9H), 0.99 (qd, *J* = 12.5, 4.2 Hz, 2H). ^{13}C NMR (126 MHz, DMSO-d_6): δ 154.1, 153.8, 150.1, 148.9, 147.9, 138.1, 129.4, 127.2, 127.0, 124.2, 122.3, 121.1, 119.8, 111.3, 104.5, 78.4, 43.2 (broad baseline peak), 42.2, 36.3, 33.2, 31.6, 28.1, 10.6. IR (thin film, υ_{max}): 2974, 2922, 2852 (m), 1682, 1595, 1545 (s). HRMS (+ESI): *m/z* (Calcd. $C_{29}H_{35}N_4O_3$ $[M+H]^+$ = 487.2704), *Obs.* 487.2711 (δ ppm = 1.5) LCMS (+ESI): *m/z 487.2* $[M+H]^+$, retention time 2.01 min, (>95% by NMR).

1-(5-Bromo-1H-indol-2-yl)butan-1-one (42)[7]

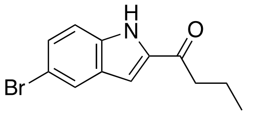

A solution of 5-bromo-1H-indole-2-carboxylic acid **34** (2.00 g, 8.4 mmol), N,N-diisopropylethylamine (DIEA) (3.84 mL, 20.8 mmol), and O-(benzotriazol-1-yl)-N,N,N′,N′-tetramethyluronium tetrafluoroborate (TBTU) (2.96 g, 8.8 mmol) was charged with methoxymethylamine hydrochloride (0.88 g, 8.8 mmol), and the reaction was diluted with dimethylformamide (DMF) (10 mL) and stirred for 16h at room temperature. The reaction was quenched with water (25 mL), and the product was extracted three times with EtOAc (50 mL). The organic layer was dried over Na_2SO_4 and reduced to minimum volume *in vacuo* to give colourless liquid, which was employed in the subsequent step without further purification. The crude colourless compound was dissolved in THF (10 mL) and cooled to 0 °C. Propylmagnesium bromide (2.80 mL 5.7 mmol) was added, and the reaction was warmed to room temperature overnight. The reaction was quenched with HCl (0.5M), and the product was extracted three times with into EtOAc (50 mL). The organic layer was dried over Na_2SO_4 and reduced to minimum volume by rotary evaporation. The crude product was purified by silica gel chromatography eluting with 0%-60% v/v EtOAc in hexane.

Yield (0.12 g, 55%), ^{1}H NMR (500 MHz, $CDCl_3$): δ 9.02 (s, 1H), 7.78 (d, *J* = 1.8 Hz, 1H), 7.35 (dd, *J* = 8.8, 1.9 Hz, 1H), 7.24 (d, *J* = 8.8 Hz, 1H), 7.05 (dd, *J* = 2.0, 0.8 Hz, 1H), 2.85 (t, *J* = 7.4 Hz, 2H), 1.80 – 1.69 (m, 2H), 0.96 (t, *J* = 7.4 Hz, 3H). ^{13}C NMR (126 MHz, $CDCl_3$): δ 193.3, 136.1, 135.6, 129.3, 129.2, 125.4, 114.0, 113.6, 108.0, 40.3, 18.4, 13.9. IR (thin film, υ_{max}): 3306 (s), 2963, 2933, 2898, 2875 (m), 1653, 1614, 1564, 1518 (s). HRMS (+ESI): *m/z* (Calcd. $C_{12}H_{13}BrNO$ $[M+H]^+$ = 266.0175), *Obs.* 266.0178 (δ ppm = 1.0), LCMS (+ESI): *m/z 266.0* $[M+H]^+$, retention time 2.15 min, (>99%).

tert-Butyl 4-(2-(5-bromo-2-butyryl-1H-indol-1-yl)ethyl)piperidine-1-carboxylate (**47**)

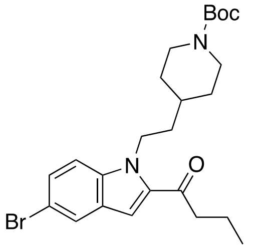

Synthesised according to General Procedure 2, Yield (0.36g, 77%), ^{1}H NMR (500 MHz, $CDCl_3$): δ 7.75 (d, *J* = 1.7 Hz, 1H), 7.36 (dd, *J* = 8.9, 1.9 Hz, 1H), 7.17 (d, *J* = 9.0 Hz, 1H), 7.15 (s, 1H), 4.49 (s, 2H), 4.03 (s, 2H), 2.87 (t, *J* = 7.4 Hz, 2H), 2.69 – 2.53 (m, 2H), 1.72 (dt, *J* = 14.8, 7.4 Hz, 2H), 1.66 (d, *J* = 14.1 Hz, 2H), 1.60 (dd, *J* = 15.2, 7.3 Hz, 2H), 1.47 – 1.41 (m, 1H), 1.38 (s, 9H), 1.14 (qd, *J* = 14.0, 4.6 Hz, 2H), 0.95 (t, *J* = 7.4 Hz, 3H). ^{13}C NMR (126 MHz, $CDCl_3$): δ 194.3, 154.9, 137.7, 135.0, 128.7, 127.5, 125.3, 113.8, 111.9, 110.6, 79.4, 43.5 (br), 43.1, 42.1, 37.0, 34.1, 32.1, 28.5, 18.6, 14.0. IR (thin film, υ_{max}): 2953, 2926, 2869 (m), 1721, 1667, 1508 (s). HRMS (+ESI): *m/z* (Calcd. $C_{24}H_{33}BrN_2O_3Na$ $[M+Na]^+$ = 499.1567), *Obs.* 499.1567 (δ ppm = 0.0). LCMS (+ESI): *m/z 476.2* $[M+Na]^+$, retention time 2.79 min, (>99%).

tert-Butyl-4-(2-(2-butyryl-5-(pyridin-4-yl)-1H-indol-1-yl)ethyl)piperidine-1-carboxylate (**51**)

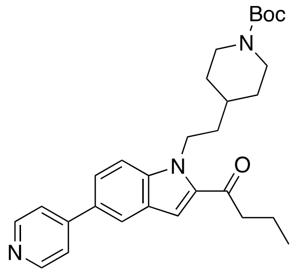

Synthesised according to General Procedure 1B, Yield (0.15g, 64%), ^{1}H NMR (400 MHz, DMSO-d_6): δ 8.61 (d, *J* = 5.4 Hz, 2H), 8.16 (s, 1H), 7.79 (d, *J* = 8.8 Hz, 1H), 7.74 (d, *J* = 5.5 Hz, 2H), 7.70 (d, *J* = 8.8 Hz, 1H), 7.60 (s, 1H), 4.69 – 4.51 (m, 2H), 3.90 (d, *J* = 11.6 Hz, 2H), 2.99 (t, *J* = 7.2 Hz, 2H), 2.79 – 2.56 (m, 2H), 1.67 (dd, *J* = 14.5, 7.2 Hz, 4H), 1.56 (dd, *J* = 14.4, 6.6 Hz, 2H), 1.50 – 1.42 (m, 1H), 1.38 (s, 9H), 1.04 (qd, *J* = 12.9, 3.9 Hz, 2H), 0.95 (t, *J* = 7.4 Hz, 3H). ^{13}C NMR (101 MHz, DMSO-d_6): δ 194.0, 153.8, 150.1, 147.6, 139.2, 135.0, 129.7, 126.0, 124.6, 121.3, 121.1, 112.5, 111.8, 78.4, 43.5, 42.4, 41.3, 36.7, 33.2, 31.6, 28.1, 18.1, 13.7. IR (thin film, υ_{max}): 2967, 2927, 2851 (m), 1737, 1686, 1663, 1617, 1595, 1522 (s). HRMS (+ESI): *m/z* (Calcd. $C_{29}H_{38}N_3O_3$ $[M+H]^+$ = 477.2866), *Obs.* 477.2853 (δ ppm = -2.7) LCMS (+ESI): *m/z 477.2* $[M+H]^+$, retention time 2.26 min, (>99%).

2-(5-Bromo-1H-indol-2-yl)-5-methyl-1,3,4-oxadiazole (43)

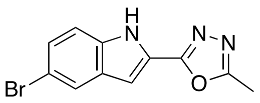

The hydrazine monohydrate (0.77 g, 15.47 mmol) was added to ethyl 5-bromo-1H-indole-2-carboxylate (1.00 g, 3.73 mmol) in EtOH (8 mL). The solution was refluxed overnight and then cooled to room temperature. The precipitate obtained was filtered and washed with ice-cold EtOH and hexane, dried *in vacuo* to give a yellow solid, which was then refluxed with glacial acetic acid (20 mL). Excess glacial acetic acid was removed *in vacuo* to yield crude product (N'-acetyl-5-bromo-1H-indole-2-carbohydrazide), which was recrystallised in EtOH. The product was filtered, dried, and added to clean 100 mL round bottom flask. $POCl_3$ (15 mL) was added, and the solution was refluxed for 2h under nitrogen. Excess $POCl_3$ was removed *in vacuo* and crushed ice was added to reaction flask. The mixture was poured into the beaker and saturated $NaHCO_3$ was added until pH was slightly basic (pH = 8). The product was filtered, dried and was purified by silica gel chromatography eluting with 0%-60% v/v EtOAc in hexane.
Yield (0.37g, 43%), ^{1}H NMR (500 MHz, $CDCl_3$): δ 9.51 (s, 1H), 7.78 – 7.75 (m, 1H), 7.35 (d, *J* = 0.6 Hz, 1H), 7.34 (d, *J* = 1.7 Hz, 1H), 7.01 (dd, *J* = 2.1, 0.6 Hz, 1H), 2.59 (s, 3H). ^{13}C NMR (126 MHz, $CDCl_3$): δ 163.6, 159.5, 135.9, 129.4, 128.0, 124.3, 122.4, 114.2, 113.4, 105.0, 11.1. IR (thin film, υ_{max}): 3196 (b), 1624, 1576 (s). HRMS (+ESI): *m/z* (Calcd. $[M+H]^+$ = 277.9924), *Obs.* 277.9929 (δ ppm = 1.9) LCMS (+ESI): *m/z 278.0* $[M+H]^+$, retention time 1.90 min, (>95%).

tert-butyl-4-(2-(5-bromo-2-(5-methyl-1,3,4-oxadiazol-2-yl)-1H-indol-1-yl)ethyl)piperidine-1-carboxylate (46)

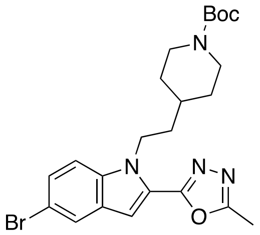

Synthesised according to General Procedure 2. This compound was isolated however contained an impurity which could not be removed. This was carried forward to the next step without purification. Crude yield (0.38g, 79%), LCMS (+ESI): *m/z 388.3* $[M+H]^+$, retention time 2.55 min, (>75%).

tert-butyl-4-(2-(2-(5-methyl-1,3,4-oxadiazol-2-yl)-5-(pyridin-4-yl)-1H-indol-1-yl)ethyl)piperidine-1-carboxylate (50)

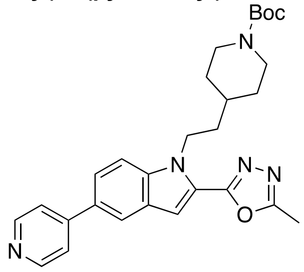

Synthesised according to General Procedure 1B. This compound was isolated however contained an impurity which could not be removed. This was carried forward to the next step without purification. Crude yield (0.14g, 59%), LCMS (+ESI): *m/z 488.3* $[M+H]^+$, retention time 1.94 min, (>85%).

Methyl 3-(pyridin-4-yl)benzoate (3)

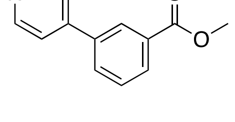

Synthesised according to general procedure 1B,Yield (0.08 g, 75%), ^{1}H NMR (500 MHz, $CDCl_3$): δ 8.63 (d, *J* = 5.8 Hz, 2H), 8.25 (s, 1H), 8.07 – 8.02 (m, 1H), 7.76 (d, *J* = 7.7 Hz, 1H), 7.50 (t, *J* = 7.7 Hz, 1H), 7.47 (d, *J* = 6.0 Hz, 2H), 3.89 (s, 3H). ^{13}C NMR (126 MHz, $CDCl_3$): δ 165.6, 149.4, 146.2, 137.5, 130.3, 130.1, 129.0, 128.3, 127.1, 120.6, 51.4. LCMS (+ESI): *m/z 214.2* $[M+H]^+$, retention time 1.25 min, (>99%)

4-(4-methoxyphenyl)pyridine (**2**)

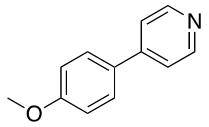

Synthesised according to general procedure 1A ,Yield (0.08 g, 81%), ^{1}H NMR (400 MHz, DMSO-d_6): δ 8.58 (d, *J* = 6.2 Hz, 2H), 7.77 (d, *J* = 8.9 Hz, 2H), 7.66 (d, *J* = 6.2 Hz, 2H), 7.07 (d, *J* = 8.9 Hz, 2H), 3.81 (s, 3H). ^{13}C NMR (101 MHz, DMSO-d_6): δ 160.3, 150.2, 146.5, 129.2, 128.1, 120.6, 114.6, 55.3. LCMS (+ESI): *m/z 186.1* $[M+H]^+$, retention time 1.08 min, (>99%).

Ethyl 5-(pyridin-4-yl)-1H-indole-2-carboxylate (**4**)

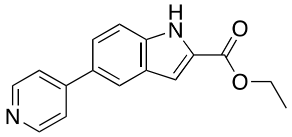

Synthesised according to general procedure 1B,Yield (0.09 g, 71%),^{1}H NMR (500 MHz, $CDCl_3$): δ 9.00 (s, 1H), 8.59 (d, *J* = 6.1 Hz, 2H), 7.93 – 7.90 (m, 1H), 7.55 (dd, *J* = 8.6, 1.8 Hz, 1H), 7.49 (d, *J* = 6.1 Hz, 2H), 7.46 (d, *J* = 8.6 Hz, 1H), 7.25 – 7.21 (m, 1H), 4.37 (q, *J* = 7.1 Hz, 2H), 1.37 (t, *J* = 7.1 Hz, 3H). ^{13}C NMR (126 MHz, $CDCl_3$): δ 161.7, 150.2, 149.0, 137.0, 131.1, 128.8, 128.1, 124.6, 121.8, 121.3, 112.6, 109.1, 61.3, 14.5. IR (thin film, υ_{max}): 3384 (s), 2991, 2162 (w), 1684, 1619, 1593, 1576, 1538, 1502, 1470, 1452, 1438, 1414, 1383, 1358, 1337, 1319, 1295, 1257, 1188, 1149, 1123, 1066, 1016, 991, 974, 885, 869, 830, 799, 768, 741, 731, 699 (s). LCMS (+ESI): *m/z 267.2* $[M+H]^+$, retention time 1.34 min, (>99%).

Ethyl-1-benzyl-5-(pyridin-4-yl)-1H-indole-2-carboxylate ***(5)***

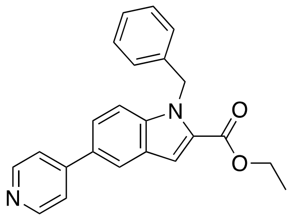

Ethyl 5-(pyridin-4-yl)-1H-indole-2-carboxylate (100 mg, 0.36 mmol) and caesium carbonate (351 mg, 1.08 mmol) were dissolved in 2 mL of DMF. 1.5 eq. of (chloromethyl)benzene (61 μL, 0.54 mmol) was added. The reaction was placed under nitrogen and heated at 80-90 °C for 24h. The reaction was cooled to room temperature and diluted with water (10 mL) and extracted twice with EtOAc (30 mL). The organic layers were combined, washed with brine solution (30 mL), dried over magnesium sulfate, and concentrated *in vacuo* to afford a solid. The crude product was purified by silica gel chromatography eluting with 0%-80% v/v EtOAc in hexane.

Yield (0.10 g, 80%), ^{1}H NMR (500 MHz, $CDCl_3$): δ 8.57 (d, *J* = 5.9 Hz, 2H), 7.92 (d, *J* = 1.3 Hz, 1H), 7.52 (dd, *J* = 8.8, 1.8 Hz, 1H), 7.48 (d, *J* = 6.1 Hz, 2H), 7.40 - 7.38 (m, 2H), 7.22 - 7.13 (m, 3H), 7.00 (d, *J* = 7.0 Hz, 2H), 5.82 (s, 2H), 4.28 (q, *J* = 7.1 Hz, 2H), 1.31 (t, *J* = 7.1 Hz, 3H). ^{13}C NMR (126 MHz, $CDCl_3$): δ 160.7, 149.1, 147.9, 138.7, 136.9, 130.0, 127.9, 127.6, 126.7, 125.6, 125.2, 123.3, 120.6, 120.2, 110.6, 110.4, 59.8, 47.1, 13.3. IR (thin film, υ_{max}): 3030, 2982, 2908, 2162 (w), 1967 (w), 1706, 1617, 1596, 1546, 1522, 1497, 1467, 1451, 1433, 1412, 1379, 1349, 1327, 1294, 1253, 1242, 1183, 1157, 1098, 1022, 991, 940, 893, 861, 823, 789, 765, 746, 731, 708, 695, 668 (s). HRMS (+ESI): *m/z* (Calc. $C_{23}H_{21}N_2O_2$ $[M+H]^+$ = 357.1598), *Obs.* 357.1590 (δ ppm = - 2.0) LCMS (+ESI): *m/z 357.2* $[M+H]^+$, retention time 1.85 min (>99%).

Ethyl 1-benzoyl-5-(pyridin-4-yl)-1H-indole-2-carboxylate (**6**)

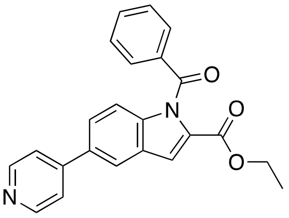

Ethyl 5-(pyridin-4-yl)-1H-indole-2-carboxylate (100 mg, 0.36 mmol), benzoyl chloride (62 μL, 0.54 mmol) and DMAP (44 mg, 0.36 mmol) were dissolved in 2 mL of DCM at 0^0C. 3 eq. of triethylamine (151 μL, 1.08 mmol) was added and the reaction was placed under nitrogen for 20 h. The reaction mixture was diluted with water (10 mL) and extracted twice with DCM (30 mL). The organic layers were combined, washed with brine solution (30 mL), dried over magnesium sulfate, and concentrated in vacuo to afford a solid. The crude product was purified by silica gel chromatography eluting with 0%-80% v/v EtOAc in hexane.

Yield (0.08 g, 62%), ^{1}H NMR (500 MHz, $CDCl_3$) δ 8.61 (d, *J* = 6.1 Hz, 2H), 7.91 (d, *J* = 1.3 Hz, 1H), 7.83 (d, *J* = 8.8 Hz, 1H), 7.66 (dd, *J* = 8.3, 1.2 Hz, 2H), 7.62 (dd, *J* = 8.8, 1.8 Hz, 1H), 7.54 (t, *J* = 7.5 Hz, 1H), 7.50 (d, *J* = 6.1 Hz, 2H), 7.42 (t, *J* = 7.8 Hz, 2H), 7.37 (d, *J* = 0.6 Hz, 1H), 3.93 (q, *J* = 7.1 Hz, 2H), 1.04 (t, *J* = 7.1 Hz, 3H). ^{13}C NMR (126 MHz, $CDCl_3$) δ 167.9, 159.7, 149.3, 147.2, 137.9, 134.3, 132.7, 132.4, 131.0, 128.4, 127.8, 126.8, 125.2, 120.7, 120.0, 114.3, 113.7, 60.5, 12.8. IR (thin film, υ_{max}): 3317 – 3000 (w), 1722, 1691, 1593, 1545, 1505, 1490, 1460, 1445, 1415, 1391, 1374, 1356, 1327, 1294, 1250, 1232, 1221, 1178, 1159, 1094, 1026, 992, 959, 918, 905, 875, 846, 811, 786, 760, 746, 714, 701, 670, 660 (s). HRMS (+ESI): *m/z* (Calc. $C_{23}H_{19}N_2O_3$ $[M+H]^+$ = 371.1390), *Obs.* 371.1390 (δ ppm = 0), LCMS (+ESI): *m/z 371.1* $[M+H]^+$, retention time 1.79 min, (98%).

Ethyl 1-(cyclohexylmethyl)-5-(pyridin-4-yl)-1H-indole-2-carboxylate (**7**)

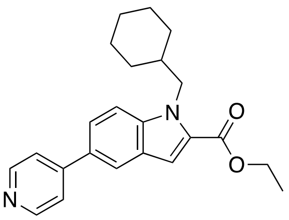

Synthesised according to general procedure 1B, Yield (0.13 g, 76 %), ^{1}H NMR (500 MHz, $CDCl_3$): δ 8.57 (d, *J* = 6.1 Hz, 2H), 7.88 (d, *J* = 1.4 Hz, 1H), 7.54 (dd, *J* = 8.8, 1.8 Hz, 1H), 7.49 (d, *J* = 6.2 Hz, 2H), 7.43 (d, *J* = 8.8 Hz, 1H), 7.31 (d, *J* = 0.6 Hz, 1H), 4.38 (d, *J* = 7.4 Hz, 2H), 4.32 (q, *J* = 7.1 Hz, 2H), 1.87 – 1.77 (m, 1H), 1.68 – 1.55 (m, 3H), 1.48 (m, 2H, overlap with H_2O), 1.36 (t, *J* = 7.1 Hz, 3H), 1.13 – 0.96 (m, 5H). ^{13}C NMR (126 MHz, $CDCl_3$): δ 161.9, 150.2, 149.0, 139.9, 130.6, 129.1, 126.3, 123.8, 121.7, 121.2, 111.9, 111.0, 60.7, 50.9, 39.5, 31.0, 26.4, 25.9, 14.4. IR (thin film, υ_{max}): 2981, 2925, 2851 (m), 1707, 1617, 1595, 1554, 1524, 1500, 1467, 1449, 1410, 1381, 1351, 1327,1295, 1233, 1220, 1190, 1178, 1160, 1149, 1094, 1019, 992, 961, 891, 863, 831, 797, 765, 746, 708, 670 (s). HRMS (+ESI): *m/z* (Calc. $C_{23}H_{27}N_2O_2$ $[M+H]^+$ = 363.2067), *Obs.* 363.2073 (δ ppm = 1.5) LCMS (+ESI): *m/z 363.2* $[M+H]^+$, retention time 2.29 min, (>99%).

Ethyl 1-(cyclobutylmethyl)-5-(pyridin-4-yl)-1H-indole-2-carboxylate (**9**)

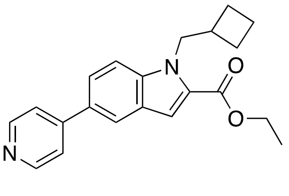

Synthesised according to general procedure 1B, Yield (0.11 g, 66 %), ^{1}H NMR (400 MHz, $CDCl_3$): δ 8.56 (d, *J* = 6.1 Hz, 2H), 7.87 (d, *J* = 1.2 Hz, 1H), 7.53 (dd, *J* = 8.8, 1.7 Hz, 1H), 7.48 (d, *J* = 6.2 Hz, 2H), 7.45 (d, *J* = 8.9 Hz, 1H), 7.29 (s, 1H), 4.59 (d, *J* = 7.1 Hz, 2H), 4.32 (q, *J* = 7.1 Hz, 2H), 2.85- 2.71 (m, 1H), 1.95-1.83 (m, 2H), 1.82-1.69 (m, 4H), 1.35 (t, *J* = 7.1 Hz, 3H). ^{13}C NMR (101 MHz, $CDCl_3$): δ 161.9, 150.2 149.0, 139.6, 130.6, 128.9, 126.4, 123.8, 121.6, 121.2, 111.6, 111.0, 60.7, 49.3, 36.8, 26.2, 18.4, 14.4. IR (thin film, υ_{max}): 2977, 2938 (m), 1705, 1613, 1594, 1544, 1522, 1498, 1463, 1405, 1390, 1352, 1324, 1296, 1265, 1239, 1219, 1185, 1163, 1149, 1126, 1092, 1012, 992, 890, 862, 829, 797, 768, 744, 702 (s). HRMS (+ESI): *m/z* (Calcd. $C_{21}H_{23}N_2O_2$ $[M+H]^+$ = 335.1754), *Obs.* 335.1765 (δ ppm = 3.4) LCMS (+ESI): *m/z 335.2* $[M+H]^+$, retention time 1.96 min, (>99%).

Ethyl 1-(cyclopentylmethyl)-5-(pyridin-4-yl)-1H-indole-2-carboxylate (**8**)

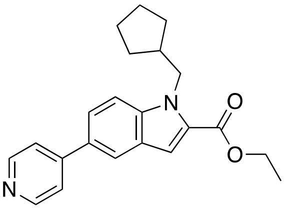

Synthesised according to general procedure 1B, Yield (0.12 g, 72 %), ^{1}H NMR (400 MHz, $CDCl_3$): δ 8.57 (d, *J* = 6.1 Hz, 2H), 7.88 (d, *J* = 1.3 Hz, 1H), 7.53 (dd, *J* = 8.8, 1.7 Hz, 1H), 7.48 (d, *J* = 6.1 Hz, 2H), 7.44 (d, *J* = 8.8 Hz, 1H), 7.31 (s, 1H), 4.50 (d, *J* = 7.5 Hz, 2H), 4.32 (q, *J* = 7.1 Hz, 2H), 2.44- 2.29 (m, 1H), 1.77-1.50 (m, 4H), 1.50-1.39 (m, 2H), 1.35 (t, *J* = 7.1 Hz, 3H), 1.31-1.17 (m, 2H).^{13}C NMR (101 MHz, $CDCl_3$): δ 161.9, 150.2, 149.0, 139.6, 130.6, 128.9, 126.4, 123.8, 121.7, 121.2, 111.7, 111.1, 60.7, 49.1, 41.6, 30.3, 24.8, 14.4. IR (thin film, υ_{max}): 2953, 2865 (m), 1708, 1614, 1594, 1545, 1523, 1499, 1466, 1411, 1382, 1340, 1323, 1295, 1238, 1197, 1172, 1154, 1135, 1091, 1021, 992, 890, 864, 826, 791, 764, 744, 706 (s). HRMS (+ESI): *m/z* (Calcd. $C_{22}H_{25}N_2O_2$ $[M+H]^+$ =349.1911), *Obs. 349.1901* (δ ppm = -2.6) LCMS (+ESI): *m/z 349.2* $[M+H]^+$, retention time 1.52 min, (98%).

1-(cyclohexylmethyl)-5-(pyridin-4-yl)-1H-indole-2-carboxamide (**13**)

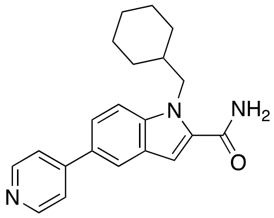

A mixture of ethyl 1-(cyclohexylmethyl)-5-(pyridin-4-yl)-1H-indole-2-carboxylate (0.362 g,1 mmol) and LiOH (71 mg, 3 mmol) in EtOH (4 mL) was refluxed and monitored by TLC until no starting material was observed. After completion, the mixture was concentrated, and crude was dissolved in diethyl ester (40 mL). DMF (one drop) and thionyl chloride (1.55 mL, 20.8 mmol) were added to the mixture and reaction was stirred at room temperature for 2h. The volatiles were removed, and the crude acyl chloride was dissolved in diethyl ether (40 mL) and added to liquid ammonia at -60 ^{0}C. The mixture was slowly warmed to room temperature and was stirred for 12 hr. The mixture was diluted with EtOAc (50 mL) and washed with water (20 mL), saturated $NaHCO_3$ (20 mL), water (20 mL) and brine (20 mL), dried over anhydrous Na_2SO_4 and concentrated to give a crude white solid.

Yield (0.25 g, 76 %), ^{1}H NMR (400 MHz, $CDCl_3$): δ 8.57 (d, *J* = 5.9 Hz, 2H), 7.86 (d, *J* = 1.1 Hz, 1H), 7.53 (dd, *J* = 8.7, 1.6 Hz, 1H), 7.49 (d, *J* = 6.1 Hz, 2H), 7.43 (d, *J* = 8.8 Hz, 1H), 6.92 (s, 1H), 4.42 (d, *J* = 7.3 Hz, 2H), 1.81 (m, 1H), 1.49 – 1.79 (m, 3H), 1.47 (d, *J* = 12.3 Hz, 2H), 1.14-0.90 (m, 5H, masked by water peak.) ^{13}C NMR (101 MHz, $CDCl_3$): δ 164.0, 150.2, 149.1, 139.5, 131.7, 130.7, 126.4, 123.4, 121.7, 120.6, 111.8, 105.9, 50.7, 39.4, 30.9, 26.4, 25.9. IR (thin film, υ_{max}): 3425, 3344, 3289, 3186, 2927, 2854 (m), 1644, 1604, 1591, 1545, 1524, 1499, 1465, 1451, 1414, 1390, 1352, 1330, 1289, 1274, 1228, 1196, 1184, 1174, 1159, 1119, 1025, 991, 963, 884, 868, 855, 827, 796, 780, 767, 745, 704, 668 (s). HRMS (+ESI): *m/z* (Calcd. $C_{21}H_{24}N_3O$ $[M+H]^+$ = 334.1914), *Obs.* 334.1921 (δ ppm = 2.0) LCMS (+ESI): *m/z 334.2* $[M+H]^+$, retention time 1.51 min, (>99%).

Ethyl 1-(piperidin-4-ylmethyl)-5-(pyridin-4-yl)-1H-indole-2-carboxylate (TFA salt) (**11**)

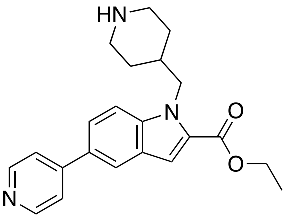

Synthesised according to general procedure 3, Yield (0.06 g, 90 %), ^{1}H NMR (500 MHz, DMSO-d_6): δ 8.62 (d, *J* = 6.1 Hz, 2H), 8.18 (t, *J* = 1.1 Hz, 1H), 7.80 (d, *J* = 1.1 Hz, 2H), 7.74 (d, *J* = 6.2 Hz, 2H), 7.41 (s, 1H), 4.58 (d, *J* = 7.2 Hz, 2H), 4.35 (q, *J* = 7.1 Hz, 2H), 3.17 (d, *J* = 13.1 Hz, 2H), 2.70 (t, *J* = 13.2 Hz, 2H), 2.08 (s, 1H), 1.54 - 1.50 (m, 2H), 1.45 – 1.38 (m, 2H), 1.36 (t, *J* = 7.1 Hz, 3H), 1.16 – 1.11 (m, 1H). ^{13}C NMR (126 MHz, DMSO-d_6): δ 161.17, 150.16, 147.56, 139.52, 129.91, 128.39, 125.82, 123.98, 121.19, 121.01, 112.36, 111.20, 60.65, 48.64, 43.12, 35.12, 26.49, 14.18. ^{19}F NMR (376 MHz, DMSO-d_6): δ -73.4. IR (thin film, υ_{max}): 3200-2500 (w), 2155, 2020 (w), 1710, 1669, 1636, 1605, 1529, 1505, 1471,

1414, 1390, 1368, 1350, 1325, 1272, 1253, 1171, 1156, 1125, 1098, 1022, 987, 967, 889, 832, 800, 767, 746, 721, 701 (s). HRMS (+ESI): *m/z* (Calcd. $C_{22}H_{26}N_3O_2$ $[M+H]^+$ =364.2020), *Obs.* 364.2022 (δ ppm = 0.7) LCMS (+ESI**):** *m/z 364.2* $[M+H]^+$, retention time 1.19 min, (>99%).

ethyl 1-(2-(piperidin-4-yl)ethyl)-5-(pyridin-4-yl)-1H-indole-2-carboxylate (**10**)

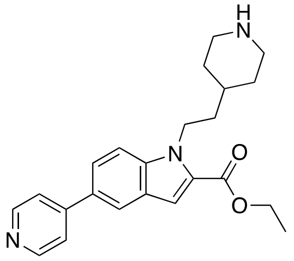

Synthesised according to general procedure 3, Yield (0.07 g, 92 %),^{1}H NMR (400 MHz, $CDCl_3$): δ 8.66 (d, J = 5.8 Hz, 1H), 7.97 (s, 1H), 7.64 (d, J = 8.7 Hz, 1H), 7.60-7.53 (m, 2H), 7.47 (t J = 10.0 Hz, 1H), 7.39 (s, 1H), 4.63-4.60 (m, 2H), 4.40-4.35 (m, 1H), 3.40 (d, J = 12.0 Hz, 1H) 3.09-2.93 (m, 2H), 2.82 (t, J =10.9 Hz, 1H), 2.05-1.89 (m, 2H), 1.88-1.71 (m, 2H), 1.40 (t J = 9.0 Hz, 4H) ^{13}C NMR (101 MHz, $CDCl_3$): δ 161.8, 150.1, 150.0, 148.9, 148.8, 139.1, 138.9, 130.8, 130.6, 128.5, 128.3, 126.5, 126.4, 124.2, 123.9, 121.6, 121.4, 121.3, 111.1, 111.0, 110.9, 110.8, 60.8, 60.7, 52.1, 44.4, 43.0, 42.4, 37.2, 36.5, 34.3, 32.3, 32.1, 29.1, 14.1. IR (thin film, υ_{max}): 3383 (w), 2926, 2850, 2772 (m), 2500 – 2100 (w), 1689, 1594, 1533, 1508, 1468, 1453, 1411, 1378, 1351, 1333, 1304, 1241, 1185, 1157, 1089, 1021, 993, 975, 955, 929, 886, 864, 842, 831, 798, 763, 746, 729, 705 (s). HRMS (+ESI): *m/z* (Calc. $C_{23}H_{28}N_3O_2$ $[M+H]^+$ = 378.2176), *Obs.* 378.2176 (δ ppm = 0.1) LCMS (+ESI): *m/z 378.2* $[M-H]^+$, retention time 1.25 min, (>99%).
In the ^{1}H NMR and ^{13}C NMR there are two conformers present. LCMS shows that there is only one compound present.

Ethyl 1-(2-morpholinoethyl)-5-(pyridin-4-yl)-1H-indole-2-*carboxylate* (**12**)

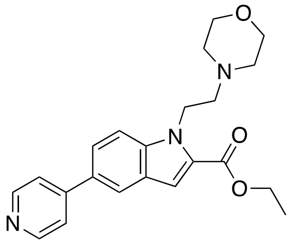

Synthesised according to general procedure 2, Yield (0.28 g, 75 %), ^{1}H NMR (500 MHz, DMSO-d_6): δ 8.68 (d, *J* = 6.2 Hz, 2H), 8.24 (d, *J* = 1.7 Hz, 1H), 7.89-7.85 (m, 3H), 7.81 (d, *J* = 8.7 Hz, 1H), 7.44 (s, 1H), 4.88 (t, *J* = 7.2 Hz, 2H), 4.40 (q, *J* = 7.1 Hz, 2H), 3.82-3.69 (m, 4H), 1.39 (t, *J* = 7.1 Hz, 3H) 6H are masked by water peak in the ^{1}H NMR spectra ^{13}C NMR (126 MHz, DMSO-d_6): δ 160.9, 148.1, 139.1, 129.5, 128.4, 126.1, 124.2, 121.5, 111.7, 111.4, 64.0, 60.7, 55.3, 52.1, 40.1, 14.0. IR (thin film, υ_{max}): 2959, 2854, 2808 (m), 1705, 1595, 1525, 1501, 1466, 1412, 1379, 1351, 1324, 1297, 1264, 1240, 1195, 1156, 1115, 1095, 1071, 1089, 992, 912, 867, 831, 796, 764, 727 (s). HRMS (+ESI): *m/z* (Calc. $C_{22}H_{26}N_3O_3$ $[M+H]^+$ =380.1969), *Obs.* 380.1973 (δ ppm = 1.1) LCMS (+ESI): *m/z 380.2* $[M+H]^+$, retention time 1.15 min, (>95%).

1-(2-(piperazin-1-yl)ethyl)-5-(pyridin-4-yl)-1H-indole *(TFA salt)* (14)

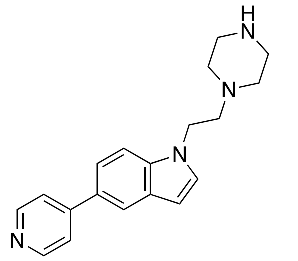

Synthesised according to general procedure 3, Yield (0.06 g, 95 %), ^{1}H NMR (400 MHz, DMSO-d_6): δ 8.75 (d, *J* = 6.0 Hz, 2H), 8.58 (s, 2H), 8.23 (d, *J* = 0.6 Hz, 1H), 8.17 (d, *J* = 5.6 Hz, 2H), 7.75 (dd, *J* = 8.8, 1.4 Hz, 1H), 7.70 (d, *J* = 8.7 Hz, 1H), 7.54 (d, *J* = 3.2 Hz, 1H), 6.59 (d, *J* = 3.1 Hz, 1H), 4.36 (t, *J* = 6.4 Hz, 2H), 3.05 (s, 4H), 2.79 (t, *J* = 6.3 Hz, 2H), 2.72 – 2.61 (m, 4H)· ^{13}C NMR (126 MHz, DMSO-d_6) δ 153.9, 144.7, 137.2, 131.0, 128.8, 126.0, 122.2, 120.6, 120.5, 111.0, 102.1, 56.8, 49.3, 43.0, 42.9. ^{19}F NMR (376 MHz, DMSO-d_6): δ -73.8. IR (thin film, υ_{max}): 3440, 3015, 2643, 2115 (w), 1670, 1636, 1608, 1521, 1479, 1421, 1342, 1323, 1182, 1128, 975, 834, 798, 965, 721 (s). HRMS (+ESI): *m/z* (Calc. $C_{19}H_{23}N_4$ [M+H]$^+$ = 307.1917), *Obs.* 307.1919 (δ ppm = 0.7), LCMS (+ESI): *m/z 307.2* [M+H]$^+$, retention *time 1.0* min, (>95%).

1-(2-(piperazin-1-yl)ethyl)-5-(pyridin-4-yl)-1H-indole-2-carboxamide *(TFA salt)* (15)

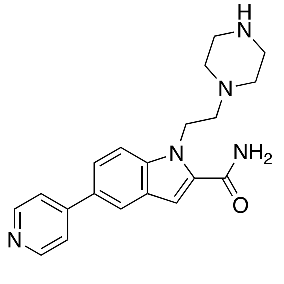

Synthesised according to general procedure 3, Yield (0.06 g, 93 %), ^{1}H NMR (500 MHz, DMSO-d_6): δ 8.81 (d, *J* = 5.2 Hz, 2H), 8.64 (s, 2H), 8.36 (d, *J* = 1.4 Hz, 1H), 8.24 (d, *J* = 5.5 Hz, 2H), 8.15 (s, 1H), 7.89 (dd, *J* = 8.8, 1.8 Hz, 1H), 7.78 (d, *J* = 8.9 Hz, 1H), 7.52 (s, 1H), 7.28 (s, 1H), 4.77 (t, *J* = 6.5 Hz, 2H), 3.09 (s, 4H), 2.87-2.68 (m, 6H). ^{13}C NMR (126 MHz, DMSO-d_6): δ 163.5, 153.7, 144.6, 139.2, 133.5, 127.2, 126.4, 122.9, 122.4, 122.0, 111.9, 106.5, 57.0, 49.5, 42.6, 41.1. ^{19}F NMR (376 MHz, DMSO-d_6): δ -74.05. IR (thin film, υ_{max}): 3341, 3139, 2931, 2819, 2479 (w), 1663, 1617, 1593, 1528, 1501, 1465, 1418, 1387, 1339, 1306, 1289, 1270, 1225, 1201, 1160, 1130, 1112, 1069, 1050, 1004, 937, 884, 859, 829, 795, 765, 745, 697, 666 (s). HRMS (+ESI): *m/z* (Calc. $C_{20}H_{24}N_5O$ [M+H]$^+$ = 350.1975), *Obs. 350.1976* (δ ppm = 0.1). LCMS (+ESI): *350.2* [M+H]$^+$, retention time 0.27min, (>95%).

3-Methyl-5-(1-(2-(piperidin-4-yl)ethyl)-5-(pyridin-4-yl)-1H-indol-2-yl)-1,2,4-oxadiazole (TFA salt) (16)

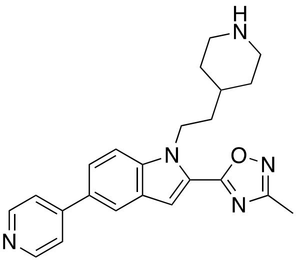

Synthesised according to general procedure 3, Yield (0.06 g, 93 %), ^{1}H NMR (500 MHz, DMSO-d_6): δ 8.77 (d, *J* = 5.7 Hz, 2H), 8.52 (d, *J* = 9.2 Hz, 1H), 8.37 (d, *J* = 1.2 Hz, 1H), 8.22 (d, *J* = 7.7 Hz, 1H), 8.09 (d, *J* = 5.0 Hz, 2H), 7.94 (dd, *J* = 8.9, 1.7 Hz, 1H), 7.88 (d, *J* = 8.9 Hz, 1H), 7.62 (d, *J* = 0.5 Hz, 1H), 4.77 (d, *J* = 9.0 Hz, 2H), 3.27 (d, *J* = 12.7 Hz, 2H), 2.91 – 2.81 (m, 2H), 2.47 (s, 3H), 1.92 (app d, *J* = 13.5 Hz, 2H), 1.76 – 1.65 (m, 3H), 1.44 – 1.32 (m, 2H). ^{13}C NMR (126 MHz, DMSO-d_6): δ 168.7, 167.4, 151.4, 146.5, 139.4, 128.8, 126.9, 124.3, 123.4, 122.2, 121.9, 112.2, 109.7, 43.0, 42.5, 35.7, 30.8, 28.2, 11.3. ^{19}F NMR (376 MHz, DMSO-d_6): δ -73.9. IR (thin film, υ_{max}): 3315 (w), 2917, 2849 (m), 1596, 1544, 1469, 1415, 1391, 1352, 1328, 1287, 1245, 1187, 1158, 1086, 993, 902, 828, 795, 753, 736, 687 (s). HRMS (+ESI): *m/z* (Calc. $C_{23}H_{26}N_5O$ [M+H]$^+$ = 388.2132), *Obs.* 388.2143 (δ ppm = 2.8) LCMS (+ESI): *m/z 388.3* [M+H]$^+$, retention time 1.21 min, (>99%).

5-Methyl-2-(1-(2-(piperidin-4-yl)ethyl)-5-(pyridin-4-yl)-1H-indol-2-yl)oxazole (TFA salt) (17)

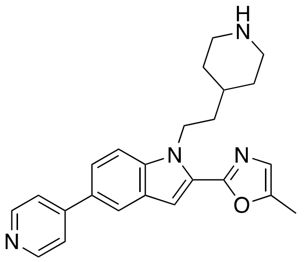

Synthesised according to general procedure 3, Yield (0.07 g, 91 %), ^{1}H NMR (400 MHz, DMSO-d$_6$) δ 8.70 (d, *J* = 6.0 Hz, 2H), 8.55 (d, *J* = 12.2 Hz, 1H), 8.27 (d, *J* = 12.6 Hz, 1H), 8.22 (d, *J* = 1.1 Hz, 1H), 7.94 (d, *J* = 6.1 Hz, 2H), 7.80 (dd, *J* = 8.8, 1.6 Hz, 1H), 7.76 (d, *J* = 8.8 Hz, 1H), 7.22 (s, 1H), 7.11 (d, *J* = 1.2 Hz, 1H), 4.87 – 4.70 (m, 2H), 3.27 (d, *J* = 12.5 Hz, 2H), 2.92 – 2.78 (m, 2H), 2.44 (d, *J* = 1.0 Hz, 3H), 1.91 (app d, *J* = 13.6 Hz, 2H), 1.78 – 1.58 (m, 3H), 1.37 (qd, *J* = 13.6, 3.1 Hz, 2H). ^{13}C NMR (126 MHz, DMSO-d$_6$) δ 154.0, 150.0, 149.0, 148.0, 138.5, 128.7, 127.3, 127.2, 124.2, 122.5, 121.6, 120.5, 111.4, 104.8, 43.1, 42.1, 35.8, 30.9, 28.3, 10.6. ^{19}F NMR (471 MHz, DMSO-d$_6$) δ -73.6. IR (thin film, υ_{max}): 3016, 2922, 2827, 2748, 2522 (m), 1662, 1634, 1604, 1546, 1522, 1466, 1454, 1437, 1422, 1390, 1337, 1345, 1324, 1295, 1272, 1172, 1124, 1076, 1029, 1010, 959, 918, 899, 887, 828, 792, 752, 719, 672 (s). HRMS (+ESI): *m/z* (Calc. $C_{24}H_{27}N_4O$ [M+H]$^+$ = 387.2179), *Obs.* 387.2182 (δ ppm = 0.6) LCMS (+ESI): *m/z 387.2* [M+H]$^+$, retention time 1.22 min, (>99%).

2-Methyl-5-(1-(2-(piperidin-4-yl)ethyl)-5-(pyridin-4-yl)-1H-indol-2-yl)-1,3,4-oxadiazole (TFA salt) (**18**)

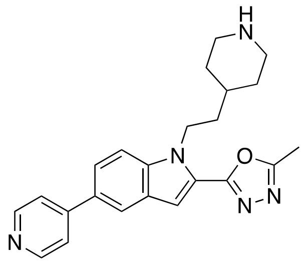

Synthesised according to general procedure 3, Yield (0.07 g, 95 %), ^{1}H NMR (500 MHz, DMSO-d$_6$): δ 8.77 (d, *J* = 5.7 Hz, 2H), 8.47 (d, *J* = 11.5 Hz, 1H), 8.34 (d, *J* = 1.5 Hz, 1H), 8.18 (d, *J* = 10.5 Hz, 1H), 8.08 (d, *J* = 4.9 Hz, 2H), 7.91 (dd, *J* = 8.9, 1.7 Hz, 1H), 7.85 (d, *J* = 8.9 Hz, 1H), 7.37 (s, 1H), 4.78 (dd, *J* = 8.0, 6.8 Hz, 2H), 3.27 (m, 2H), 2.87 (q, *J* = 10.5 Hz, 2H), 2.64 (s, 3H), 1.93 (d, *J* = 13.5 Hz, 2H), 1.76 -1.61 (m, 3H), 1.38 (qd, *J* = 13.5, 3.5 Hz, 2H). ^{13}C NMR (126 MHz, DMSO-d$_6$): δ 163.4, 158.6, 146.5, 139.1, 128.5, 127.1, 123.7, 123.5, 122.1, 121.5, 111.9, 107.1, 43.1, 42.7, 35.7, 30.9, 28.3, 10.6. ^{19}F NMR (471 MHz, DMSO-d$_6$): δ -73.9. IR (thin film, υ_{max}): 3007, 2829, 2757, 2516, 2165 (m), 1659, 1634, 1602, 1575, 1522, 1474, 1456, 1437, 1421, 1344, 1325, 1300, 1272, 1257, 1211, 1173, 1133, 1085, 1042, 1024, 1000, 976, 960, 917, 892, 829, 802, 753, 719, 690, 677 (s). HRMS (+ESI): *m/z* (Calcd. $C_{23}H_{26}N_5O$ [M+H]$^+$ = 388.2132), *Obs. 388.2136* (δ ppm = 1.2) LCMS (+ESI): *m/z 388.3* [M+H]$^+$, retention time 1.21min, (>99%).

1-(1-(2-(piperidin-4-yl)ethyl)-5-(pyridin-4-yl)-1H-indol-2-yl)butan-1-one (TFA salt) (**19**)

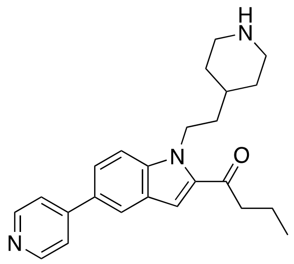

Synthesised according to general procedure 3, Yield (0.07 g, 94 %), ^{1}H NMR (400 MHz, DMSO-d$_6$): δ 8.85 (d, *J* = 6.6 Hz, 2H), 8.71 (d, *J* = 10.0 Hz, 1H), 8.42 (d, *J* = 1.5 Hz, 1H), 8.39 (d, *J* = 10.0 Hz, 1H), 8.27 (d, *J* = 6.7 Hz, 2H), 7.98 (dd, *J* = 8.9, 1.8 Hz, 1H), 7.82 (d, *J* = 8.9 Hz, 1H), 7.69 (s, 1H), 4.72 – 4.51 (m, 2H), 3.28 (d, *J* = 12.4 Hz, 2H), 3.03 (t, *J* = 7.2 Hz, 2H), 2.86 (q, *J* = 11.9 Hz, 2H), 1.90 (d, *J* = 13.3 Hz, 2H), 1.78 – 1.53 (m, 5H), 1.38 (q, *J* = 12.4 Hz, 2H), 0.96 (t, *J* = 7.2 Hz, 3H). ^{13}C NMR (126 MHz, DMSO-d$_6$): δ 194.1, 153.8, 144.2, 140.0, 135.4, 127.5, 126.1, 124.9, 123.2, 122.7, 113.0, 112.2, 43.1, 42.4, 41.3, 36.3, 31.0, 28.3, 18.0, 13.6. ^{19}F NMR (376 MHz, DMSO-d$_6$): δ -74.1. IR (thin film, v_{max}): 3000-2500 (w), 1668, 1635, 1609, 1503, 1469, 1412, 1397, 1349, 1328, 1271, 1175, 1168, 1127, 1015, 984, 893, 834, 796, 721, 705 (s). HRMS (+ESI): *m/z* (Calcd. $C_{24}H_{30}N_3O$ [M+H]$^+$ = 376.2383), *Obs.* 376.2382 (δ ppm = -0.4) LCMS (+ESI): *m/z 376.2* [M+H]$^+$, retention time 1.25 min, (>99%).

References

[1] "DIALS: implementation and evaluation of a new integration package" can be found under https://onlinelibrary.wiley.com/iucr/doi/10.1107/S2059798317017235

[2] P. R. Evans, G. N. Murshudov, *Acta Cryst. D.* **2013**, *69*, 1204–1214.

[3] A. J. McCoy, R. W. Grosse-Kunstleve, P. D. Adams, M. D. Winn, L. C. Storoni, R. J. Read, *J. Appl Cryst,* **2007**, *40*, 658–674.

[4] P. Emsley, B. Lohkamp, W. G. Scott, K. Cowtan, *Acta Cryst D,* **2010**, *66*, 486–501.
[5] P. V. Afonine, R. W. Grosse-Kunstleve, N. Echols, J. J. Headd, N. W. Moriarty, M. Mustyakimov, T. C. Terwilliger, A. Urzhumtsev, P. H. Zwart, P. D. Adams, *Acta Cryst D,* **2012**, *68*, 352–367
[6] A. Bruel, C. Logé, M-L de Tauzia, M. Ravache, R. Le Guevel, C. Guillouzo, J-F Lohier, J. Sopkova-de Oliveira Santos, O. Lozach, L. Meijer, S. Ruchaud, M. Bénédetti, J-M. Roberet, *Eur. J. Med. Chem.*, **2012**, *57*, 225-233.
[7] S. J. Taylor, A. Abeywardane, S. Liang, I. Muegge, A.K. Padyana, Z. Xiong, M. Hill-Drzewi, B. Farmer, X. Li., B. Collins, J. X. Li, A Heim-Riether, J. Proudfoot, Q. Zhang, D. Goldberg, L. Zuvela-Jelaska, H. Zaher, J. Li, N. A. Farrow, *J. Med Chem.*, **2011**, *54*, 8174-8187.

NMR Spectra

Ethyl (E)-2-(2-(4-bromophenyl)hydrazineylidene)propanoate (23)

^{1}H NMR Spectra (400 MHz, ($CDCl_3$)

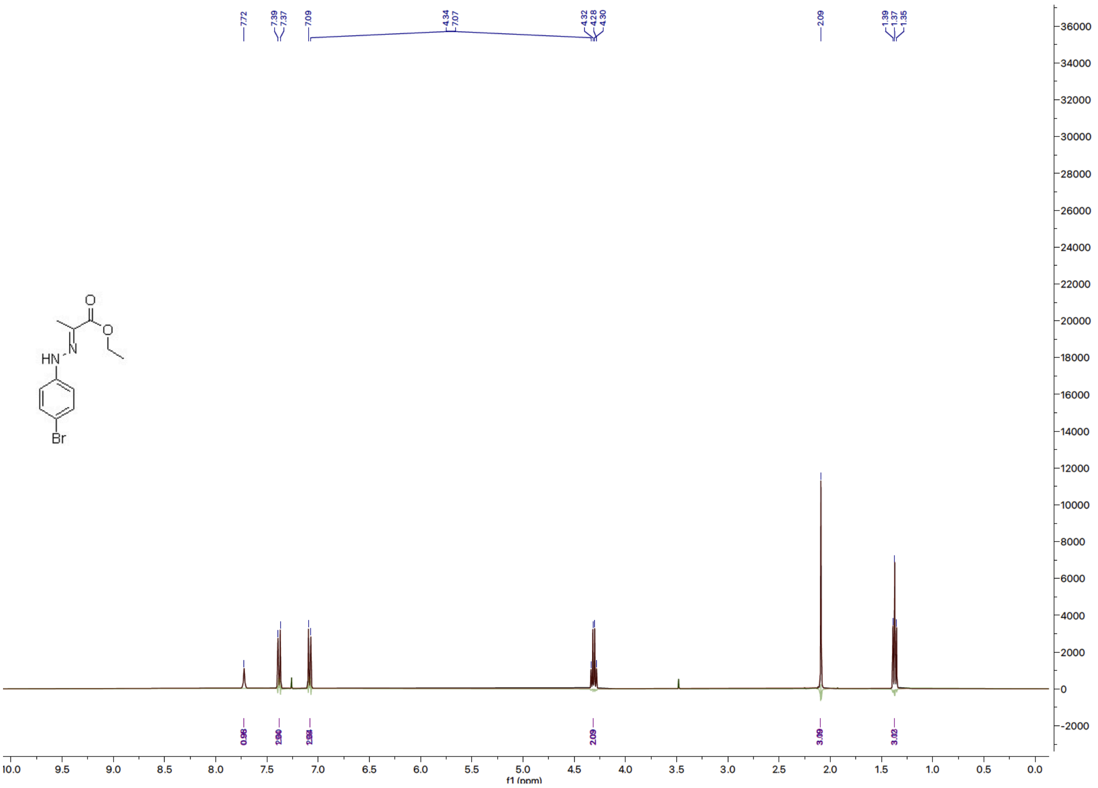

^{13}C NMR Spectra (101 MHz, (CDCl$_3$)

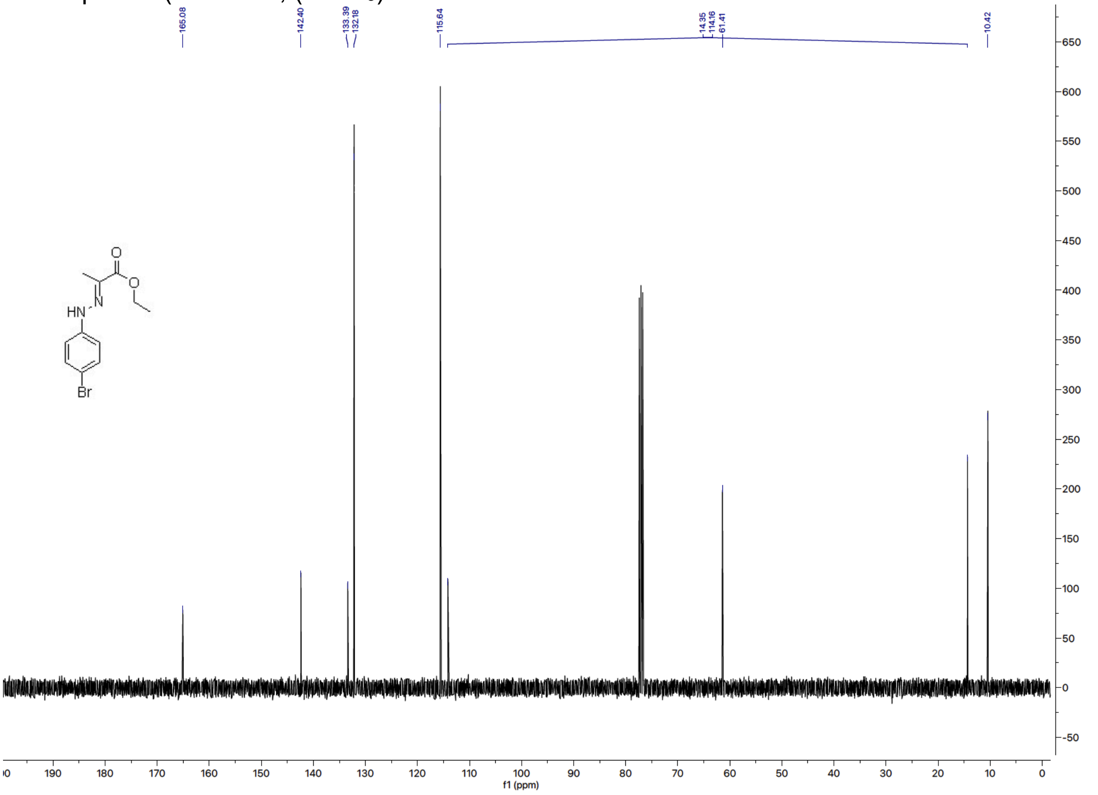

Ethyl 5-bromo-1H-indole-2-carboxylate (21)

^{1}H NMR Spectra (400 MHz, (DMSO-d$_6$)

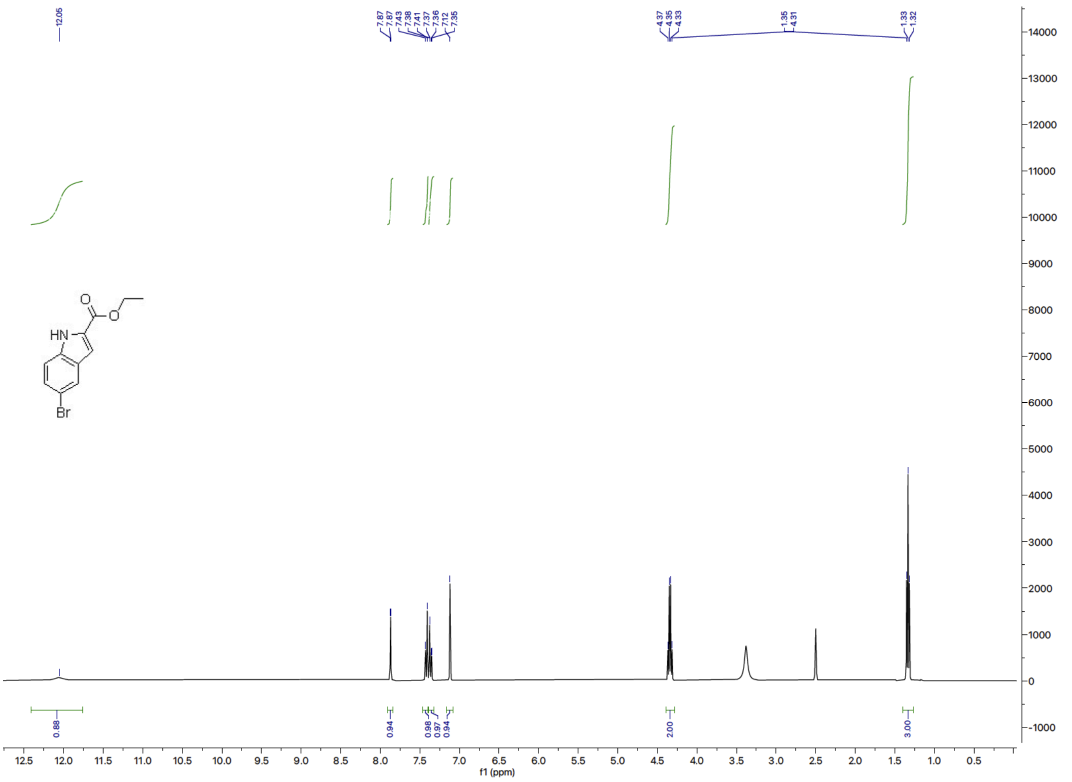

^{13}C NMR Spectra (101 MHz, (DMSO-d_6)

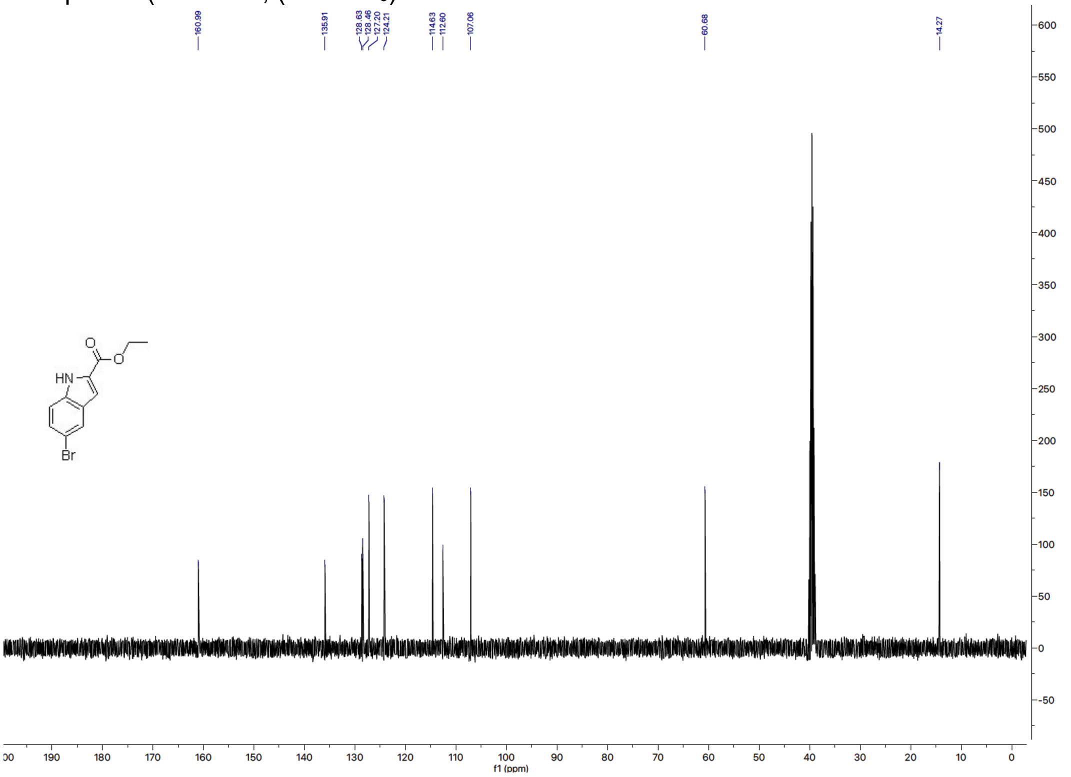

Ethyl 5-bromo-1-(cyclohexylmethyl)-1H-indole-2-carboxylate (30)

^{1}H NMR Spectra (400 MHz, ($CDCl_3$))

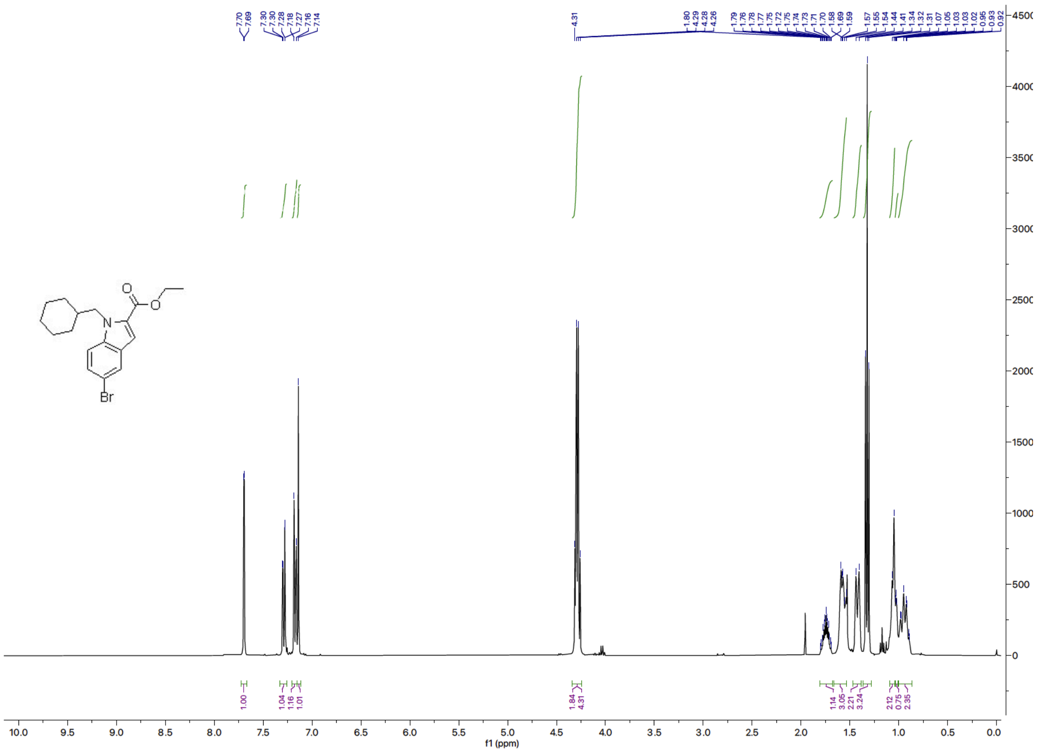

^{13}C NMR Spectra (101 MHz, ($CDCl_3$))

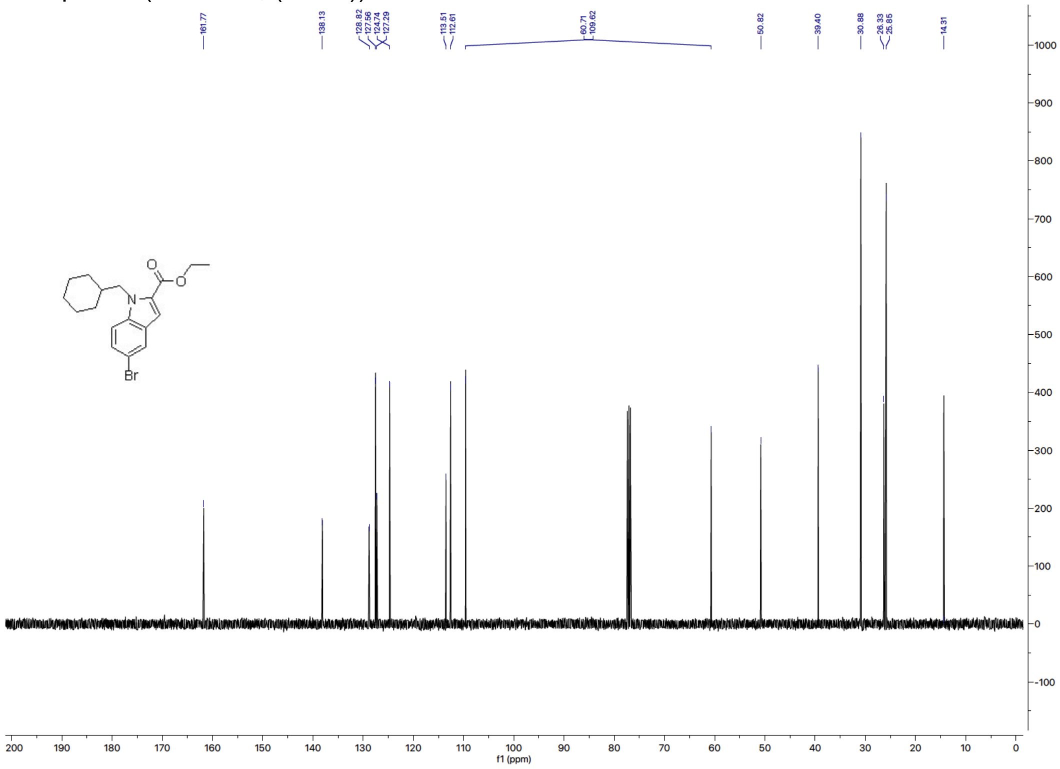

Ethyl 5-bromo-1-(cyclobutylmethyl)-1H-indole-2-carboxylate (**28**)

^{1}H NMR Spectra (400 MHz, ($CDCl_3$))

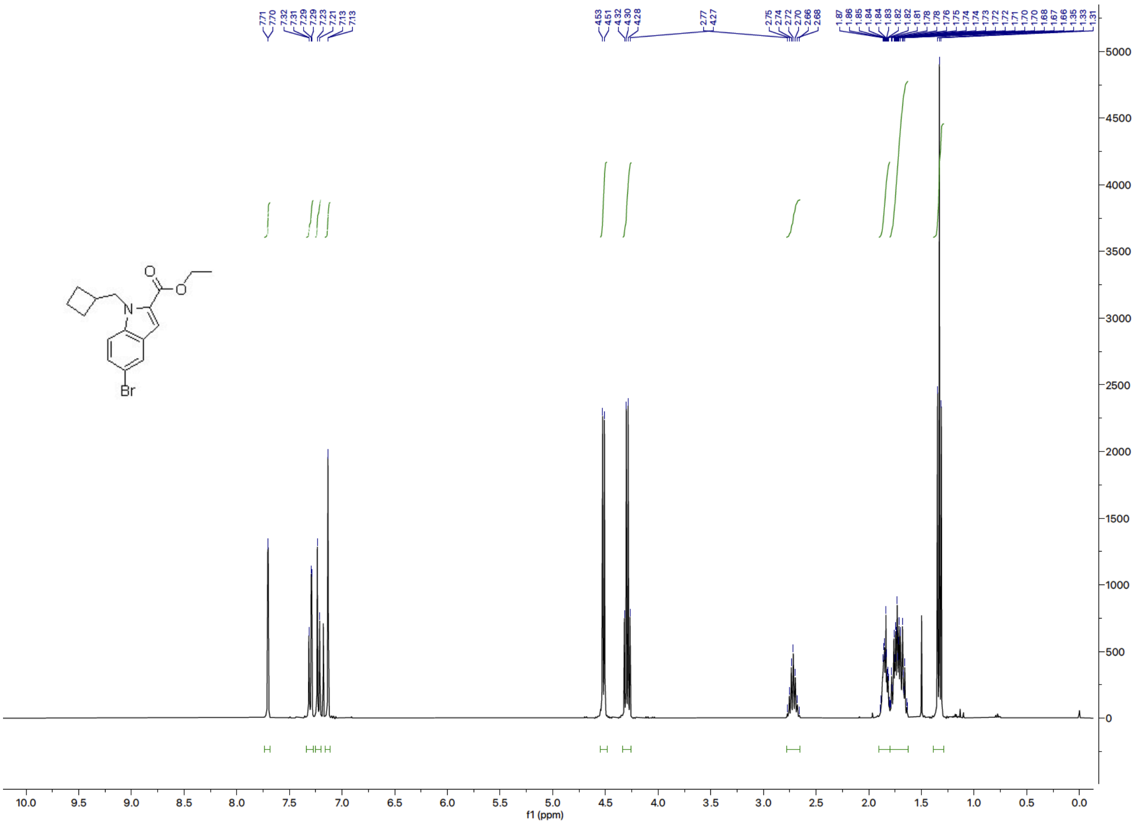

^{13}C NMR Spectra (101 MHz, ($CDCl_3$))

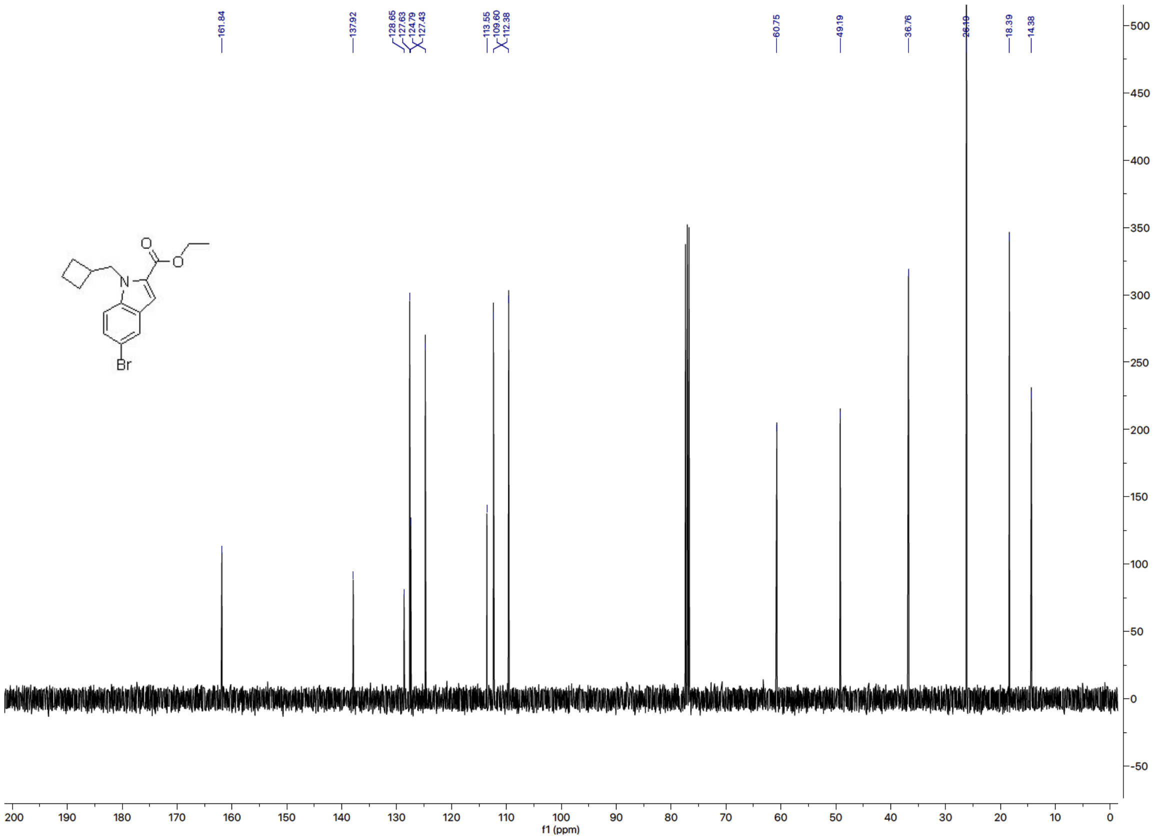

Ethyl 5-bromo-1-(cyclopentylmethyl)-1H-indole-2-carboxylate (29)

^{1}H NMR Spectra (400 MHz, ($CDCl_3$))

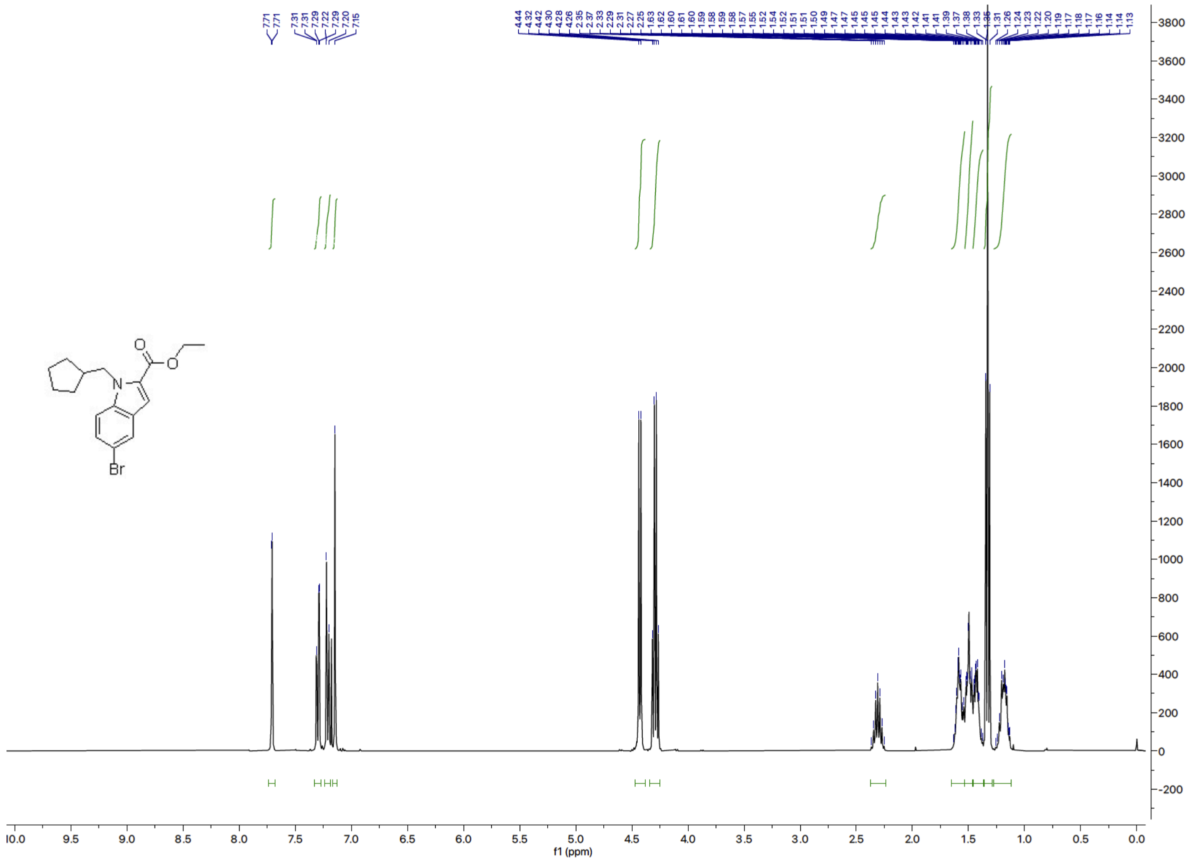

^{13}C NMR Spectra (101 MHz, ($CDCl_3$))

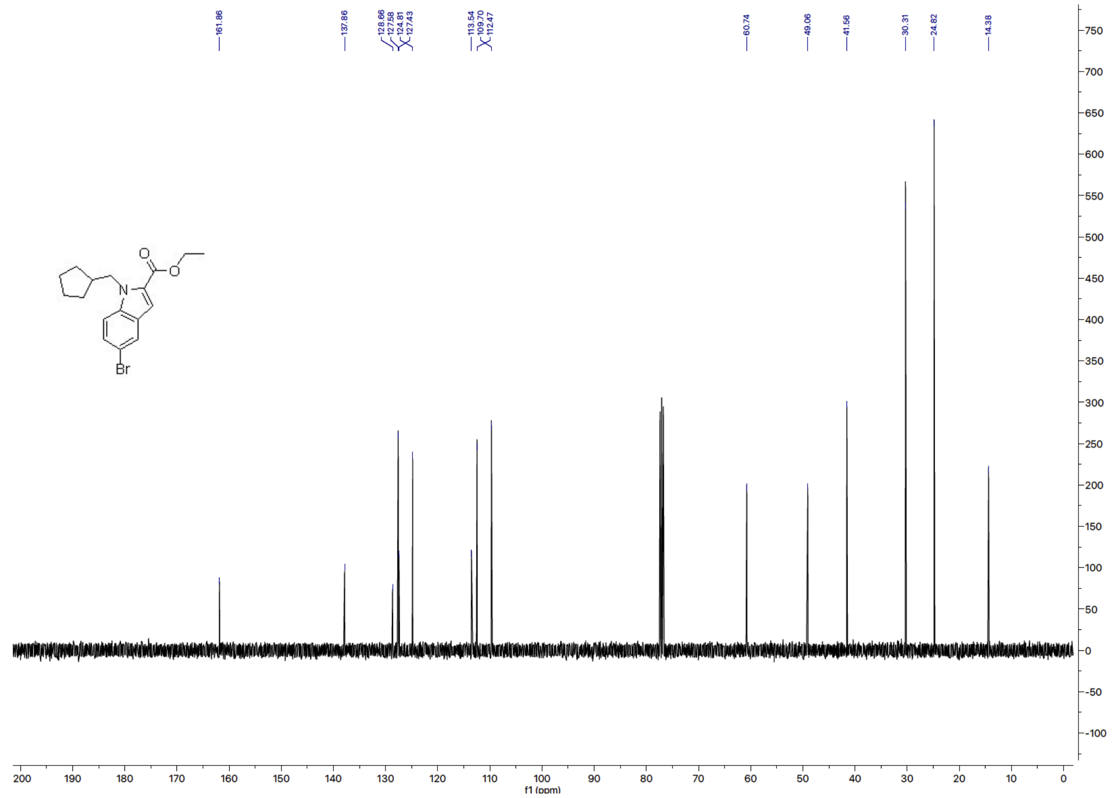

tert-butyl 4-((tosyloxy)methyl)piperidine-1-carboxylate (25)

^{1}H NMR Spectra (400 MHz, ($CDCl_3$))

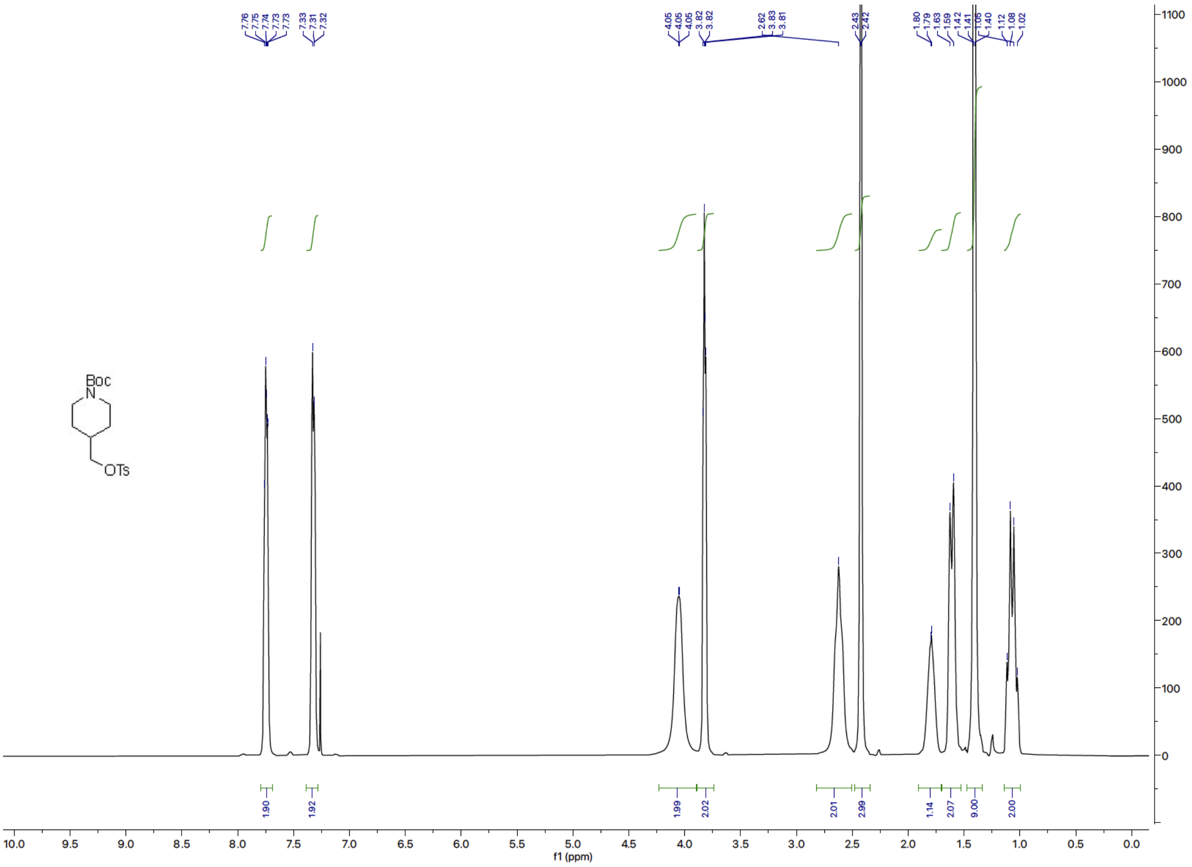

^{13}C NMR Spectra (101 MHz, ($CDCl_3$))

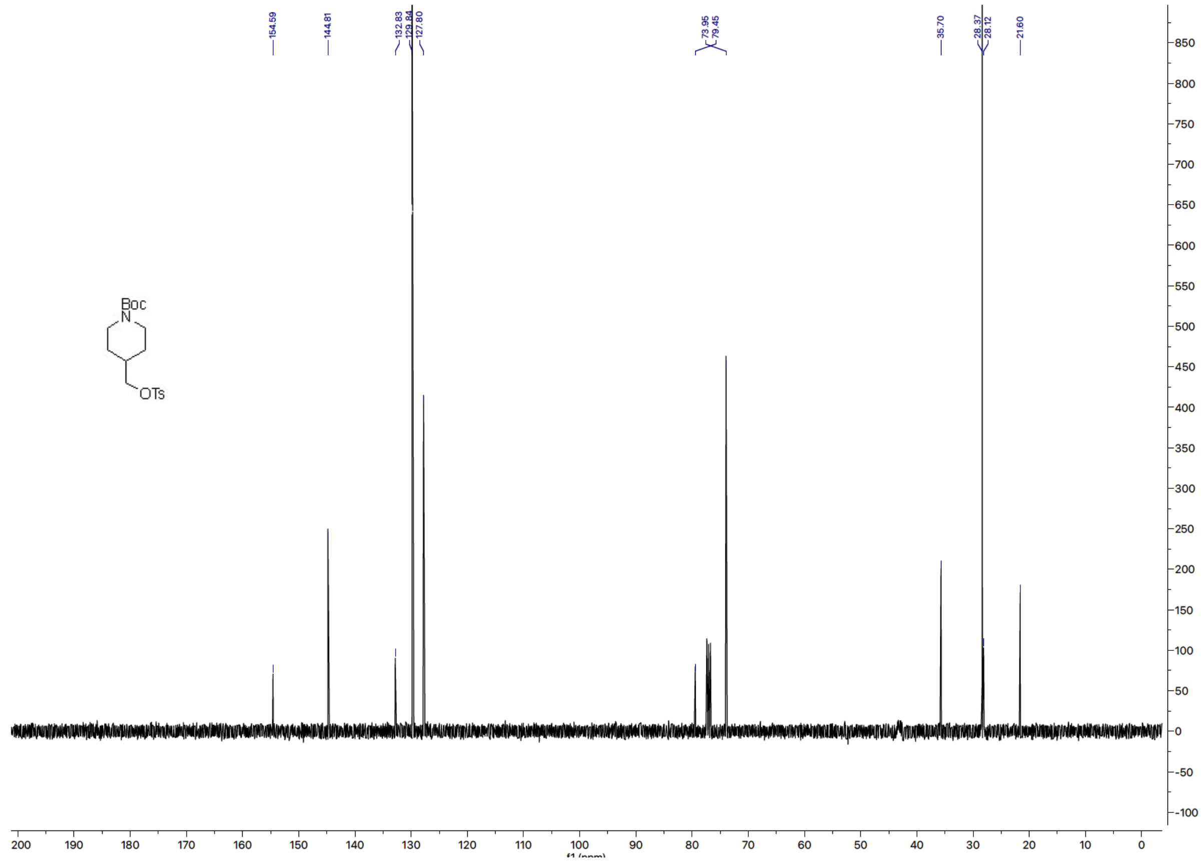

Ethyl-5-bromo-1-((1-(tert-butoxycarbonyl)piperidin-4-yl)methyl)-1H-indole-2-carboxylate (31)

^{1}H NMR Spectra (400 MHz, ($CDCl_3$))

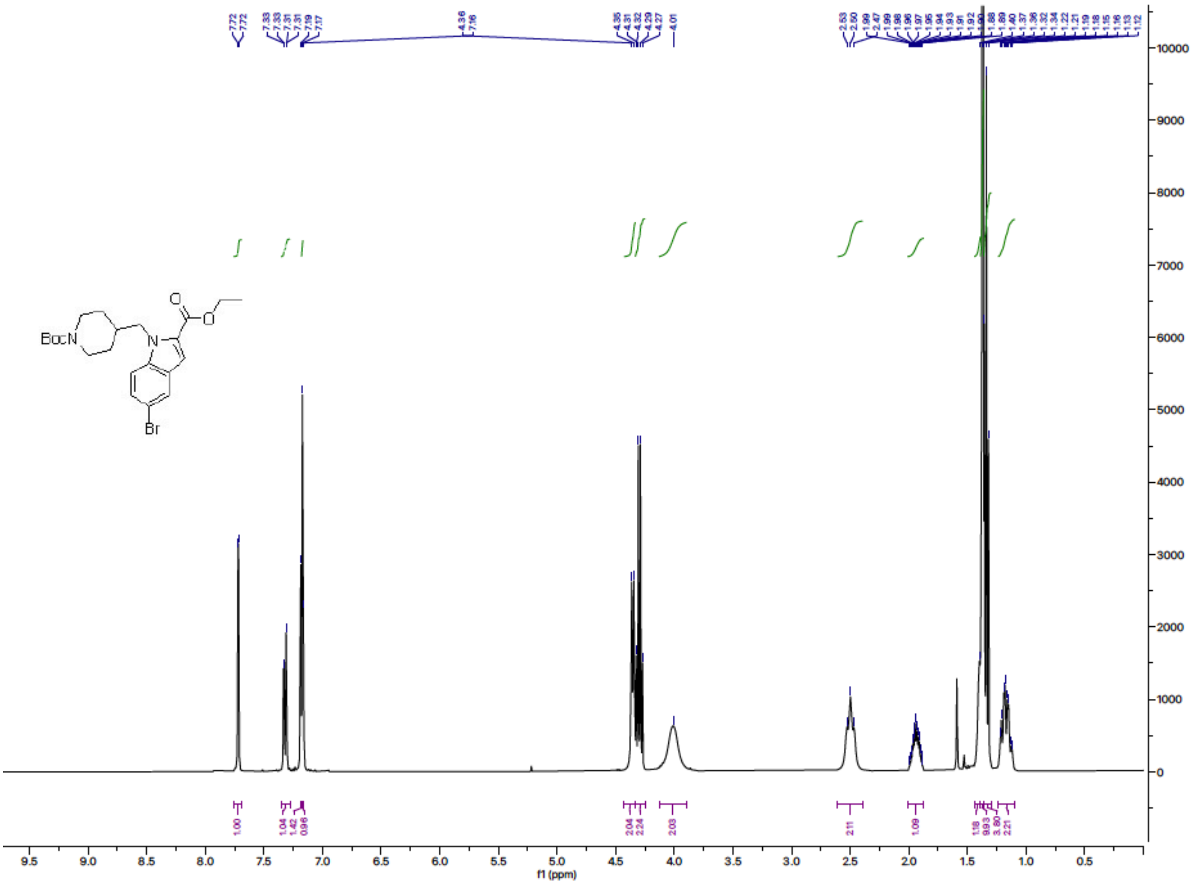

^{13}C NMR Spectra (101 MHz, ($CDCl_3$))

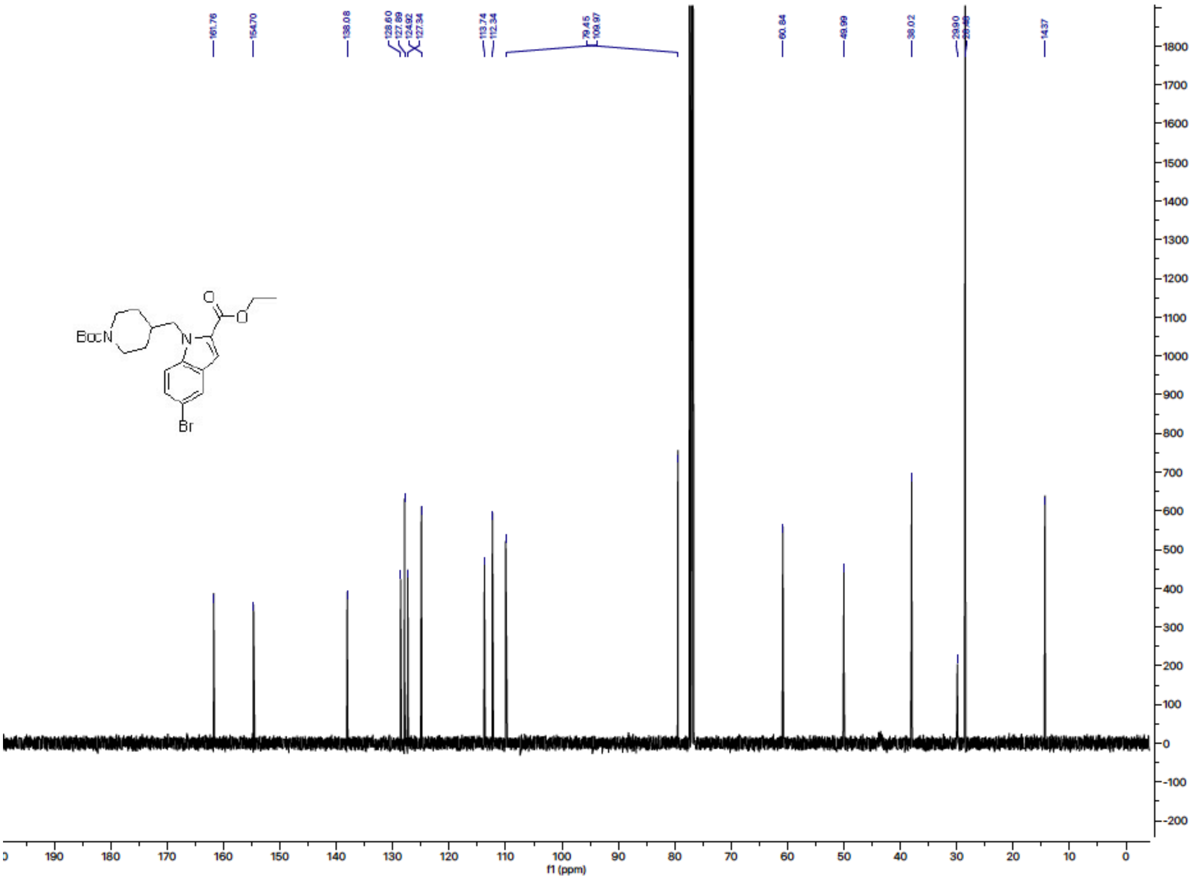

Ethyl-1-((1-(tert-butoxycarbonyl)piperidin-4-yl)methyl)-5-(pyridin-4-yl)-1H-indole-2-carboxylate (32)

^{1}H NMR Spectra (500 MHz, ($CDCl_3$))

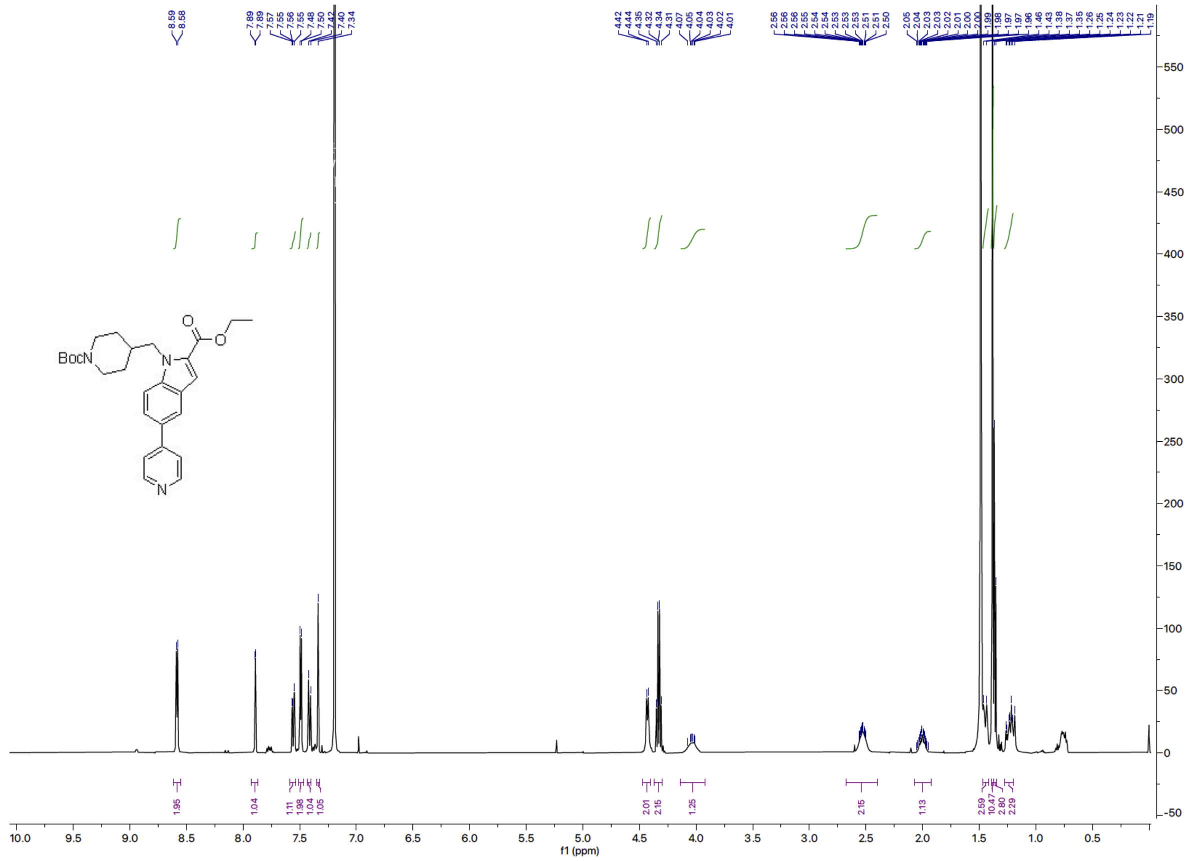

^{13}C NMR Spectra (126 MHz, ($CDCl_3$))

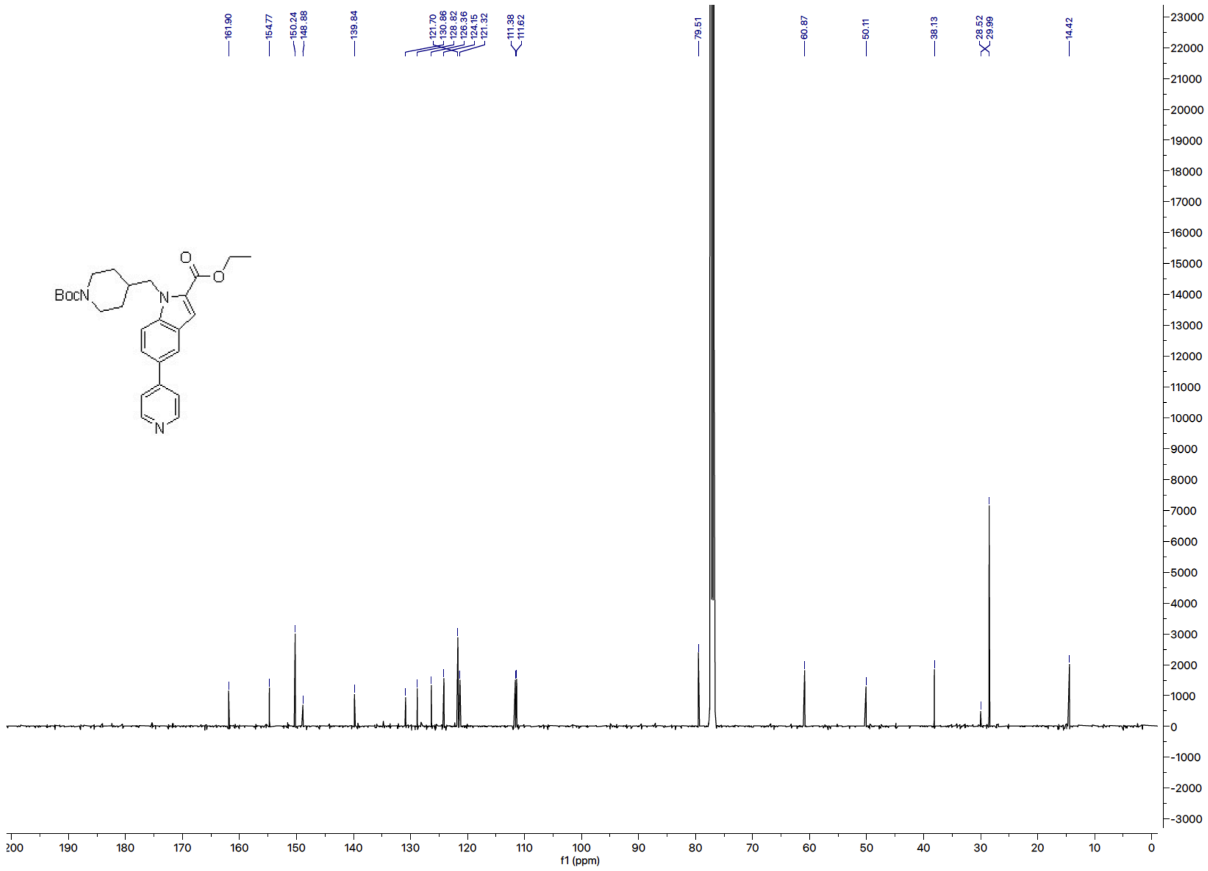

tert-butyl 4-(2-((methylsulfonyl)oxy)ethyl)piperidine-1-carboxylate (24)

^{1}H NMR Spectra (500 MHz, ($CDCl_3$))

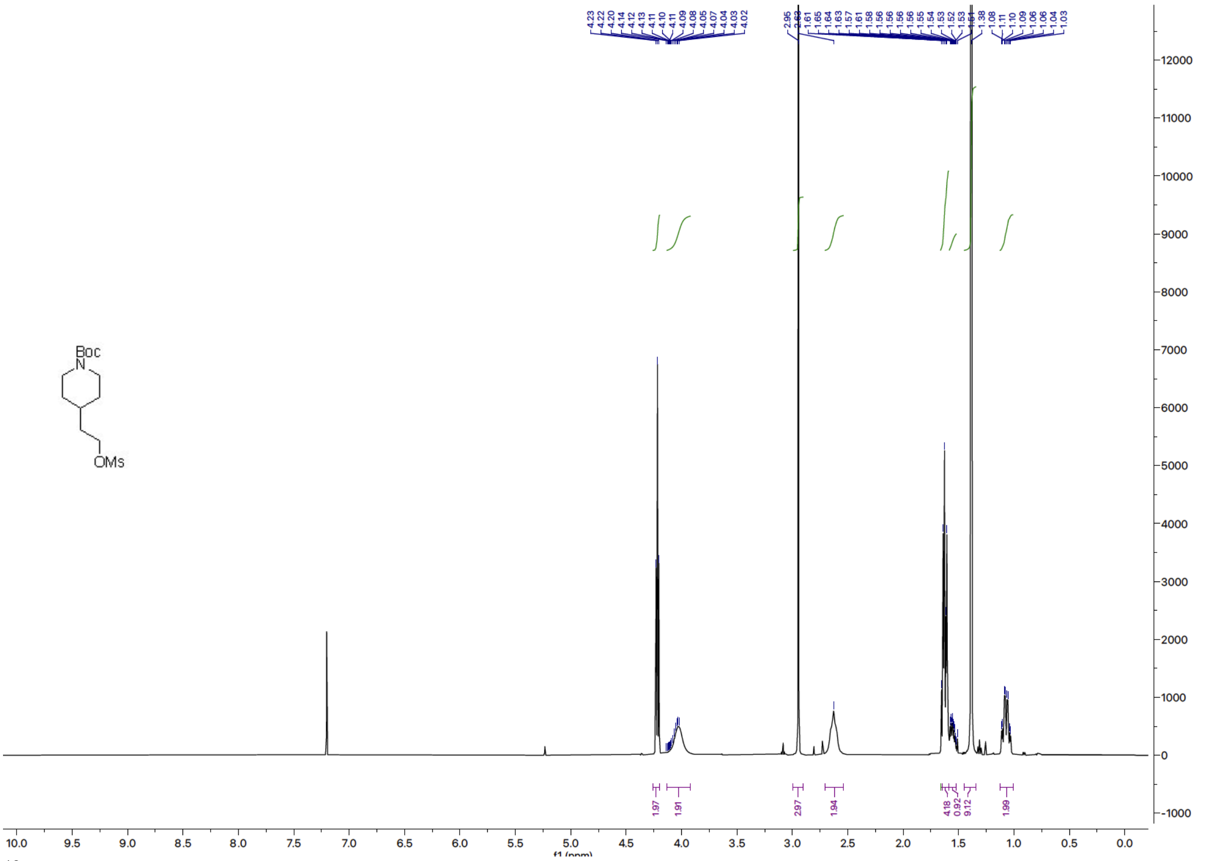

^{13}C NMR Spectra (126 MHz, ($CDCl_3$))

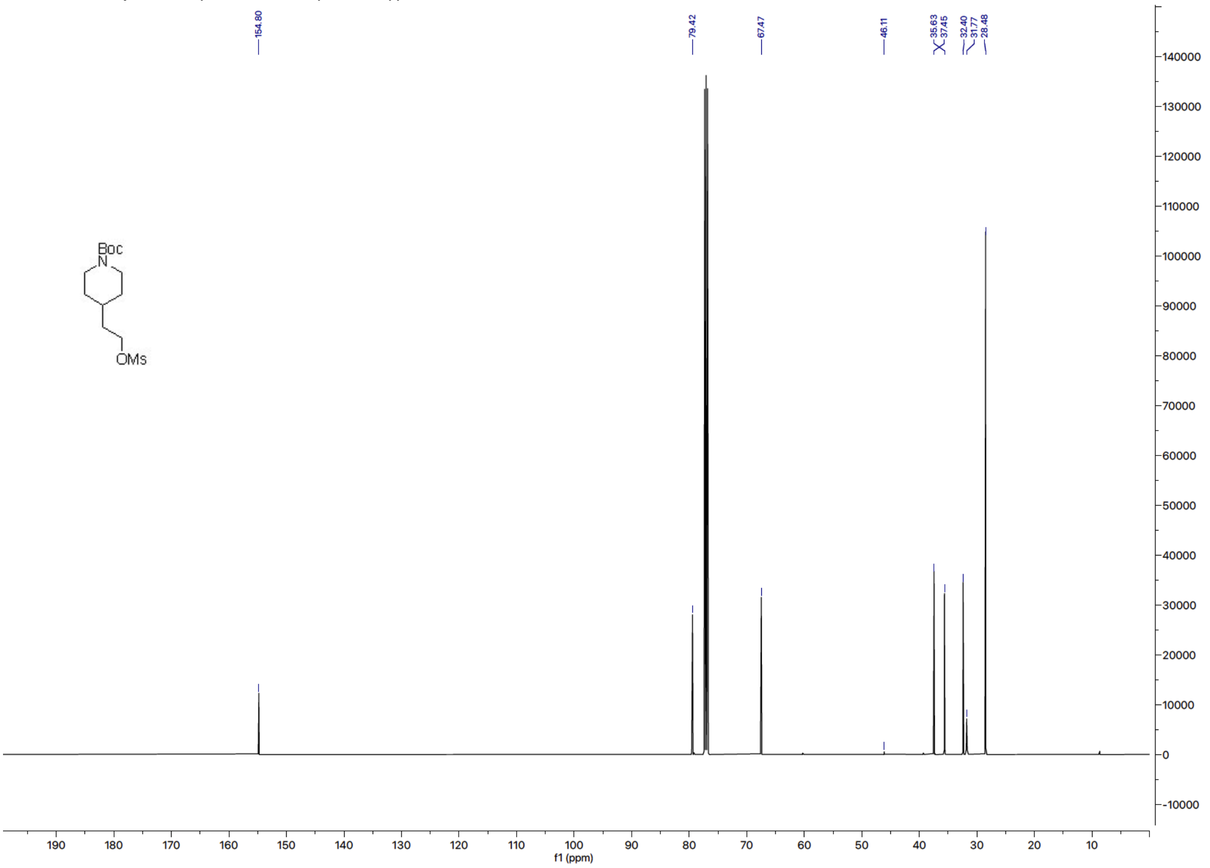

Ethyl-5-bromo-1-(2-(1-(tert-butoxycarbonyl)piperidin-4-yl)ethyl)-1H-indole-2-carboxylate (26)

^{1}H NMR Spectra (400 MHz, ($CDCl_3$))

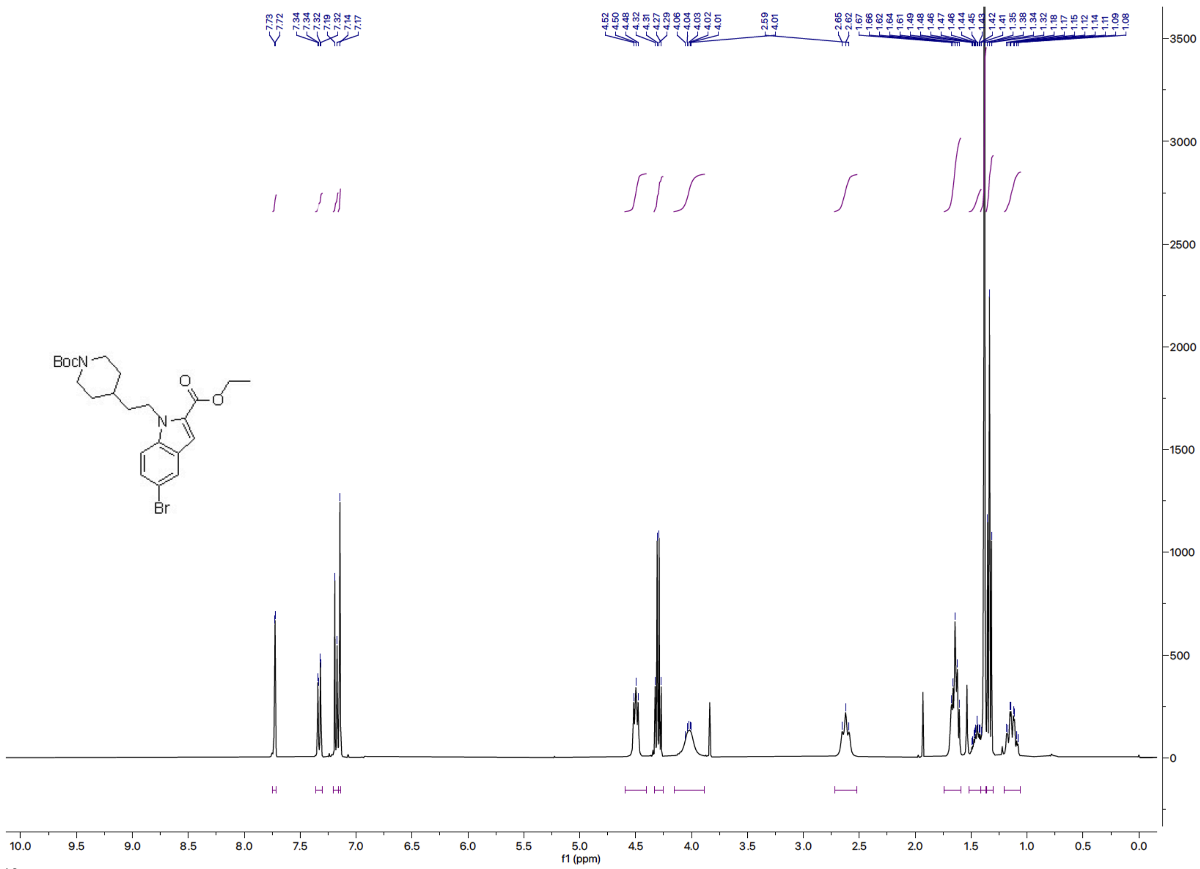

^{13}C NMR Spectra (101 MHz, ($CDCl_3$))

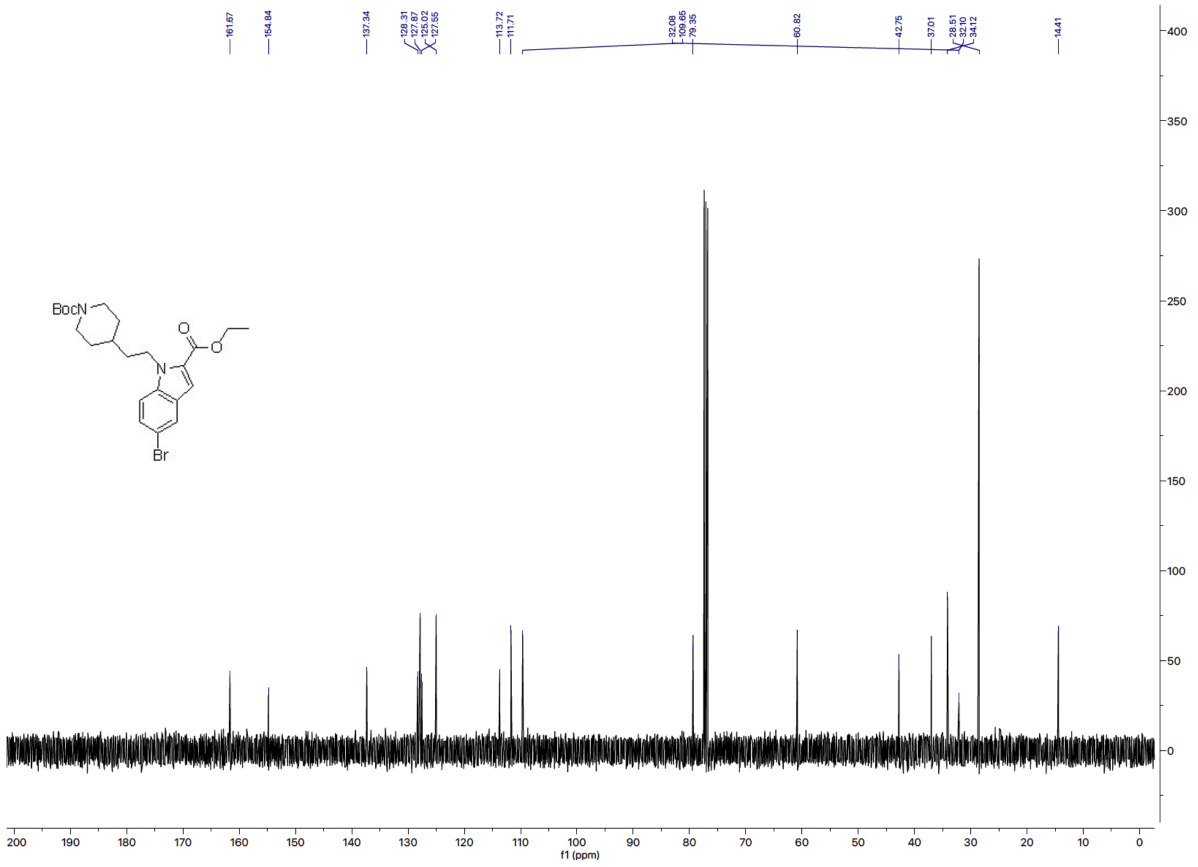

Ethyl-1-(2-(1-(tert-butoxycarbonyl)piperidin-4-yl)ethyl)-5-(pyridin-4-yl)-1H-indole-2-carboxylate (27)

^{1}H NMR Spectra (500 MHz, ($CDCl_3$))

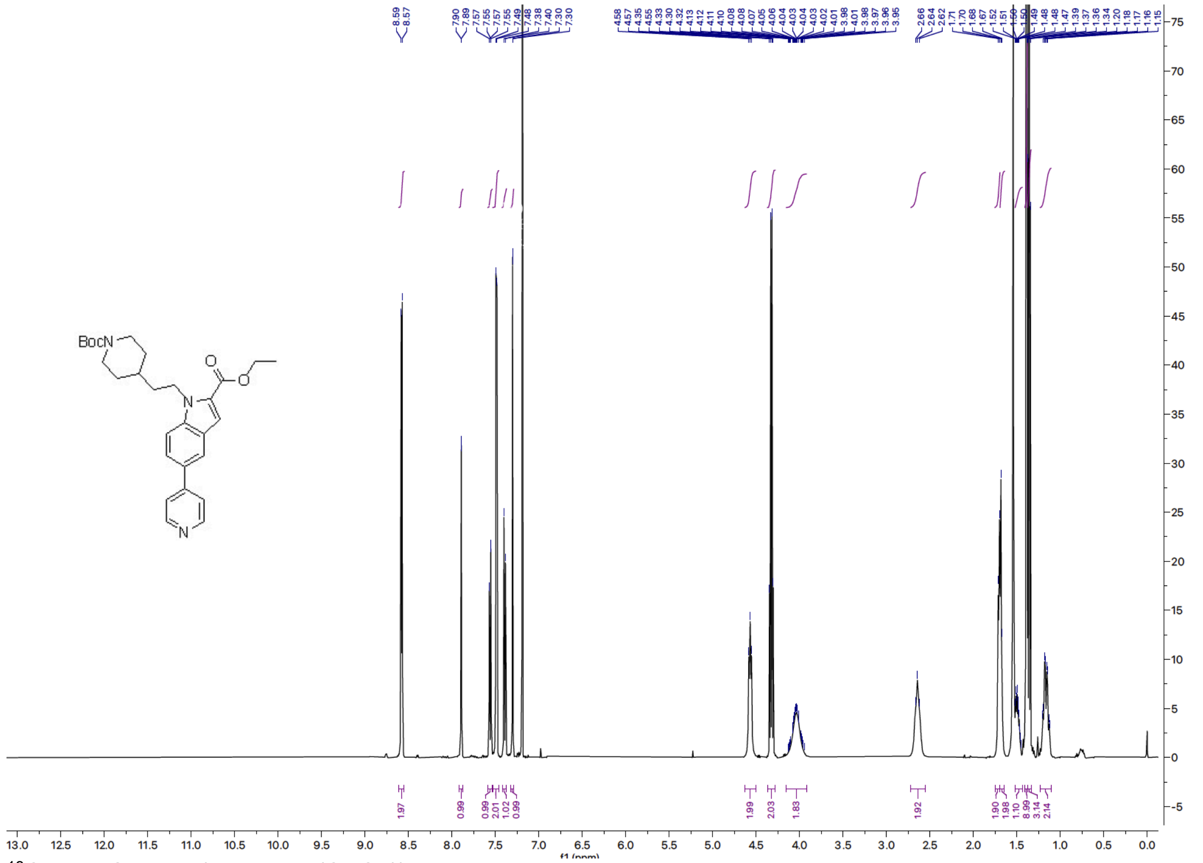

^{13}C NMR Spectra (126 MHz, ($CDCl_3$))

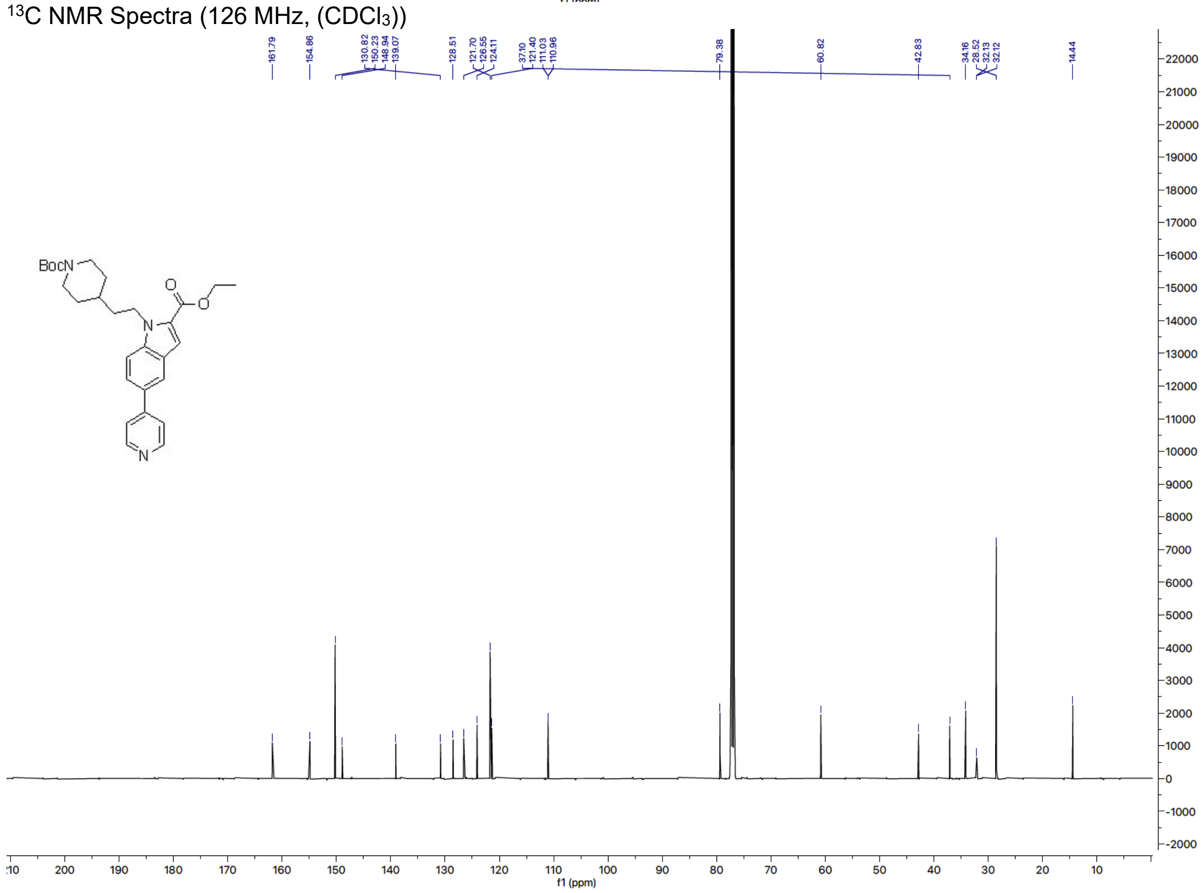

tert-butyl 4-(2-(5-(pyridin-4-yl)-1H-indol-1-yl)ethyl)piperazine-1-carboxylate (39)

^{1}H NMR Spectra (500 MHz, ($CDCl_3$))

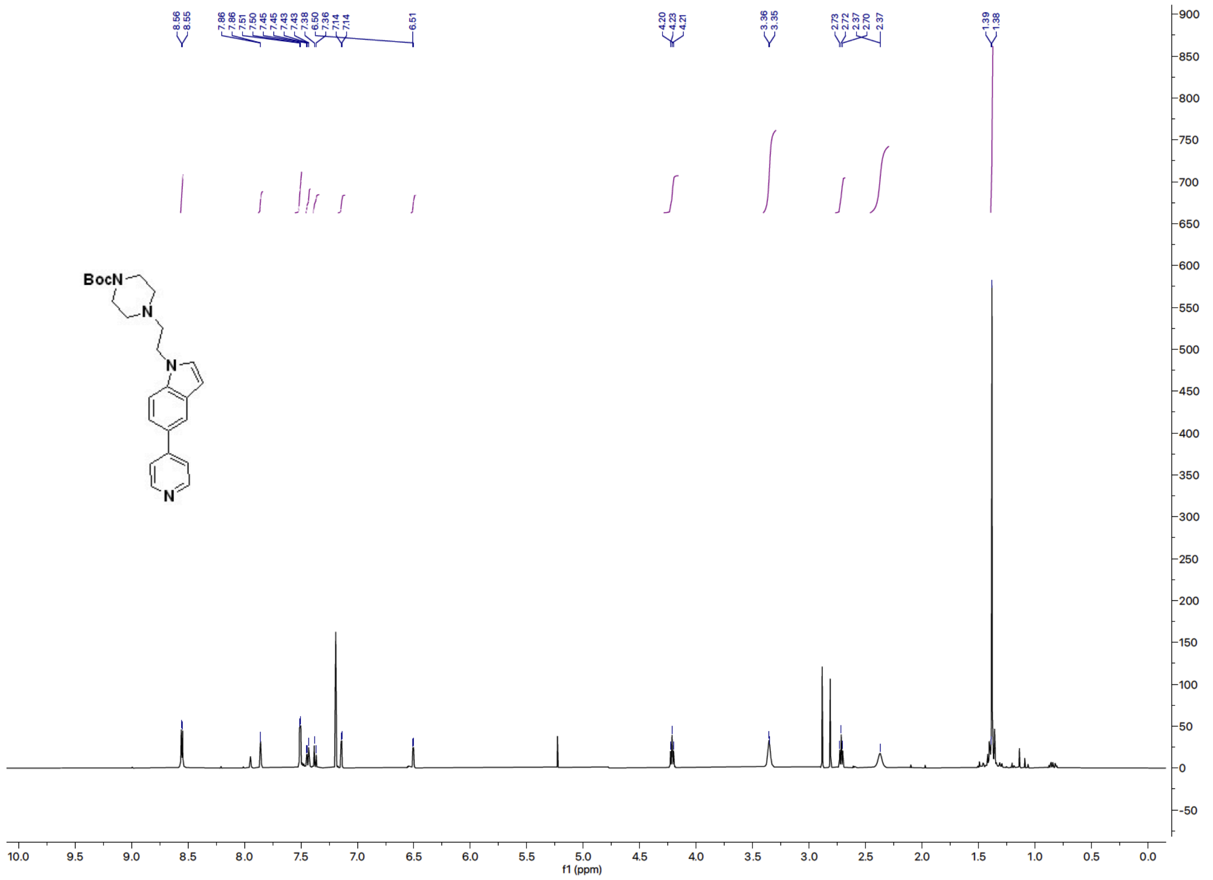

^{13}C NMR Spectra (126 MHz, ($CDCl_3$))

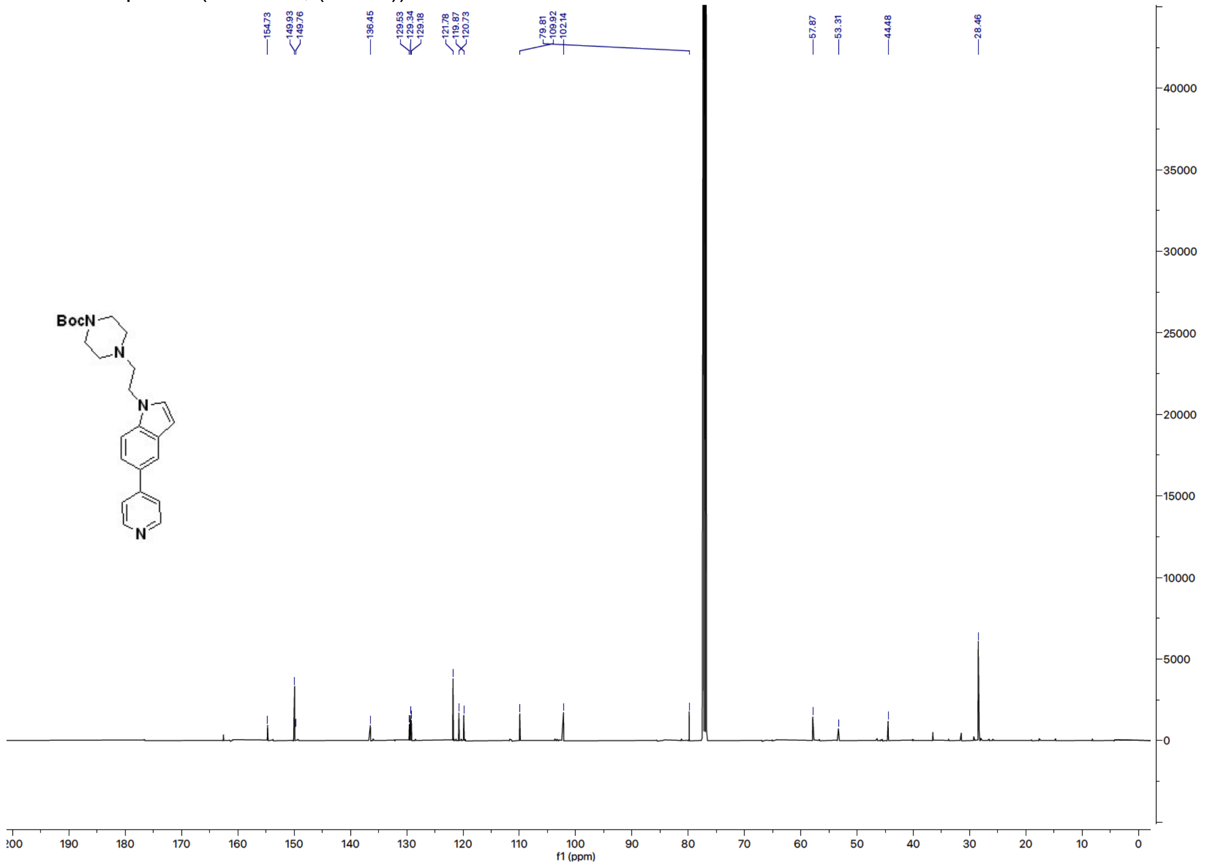

tert-Butyl-4-(2-(5-bromo-2-carbamoyl-1H-indol-1-yl)ethyl)piperazine-1-carboxylate (36)

^{1}H NMR Spectra (400 MHz, (DMSO-d_6))

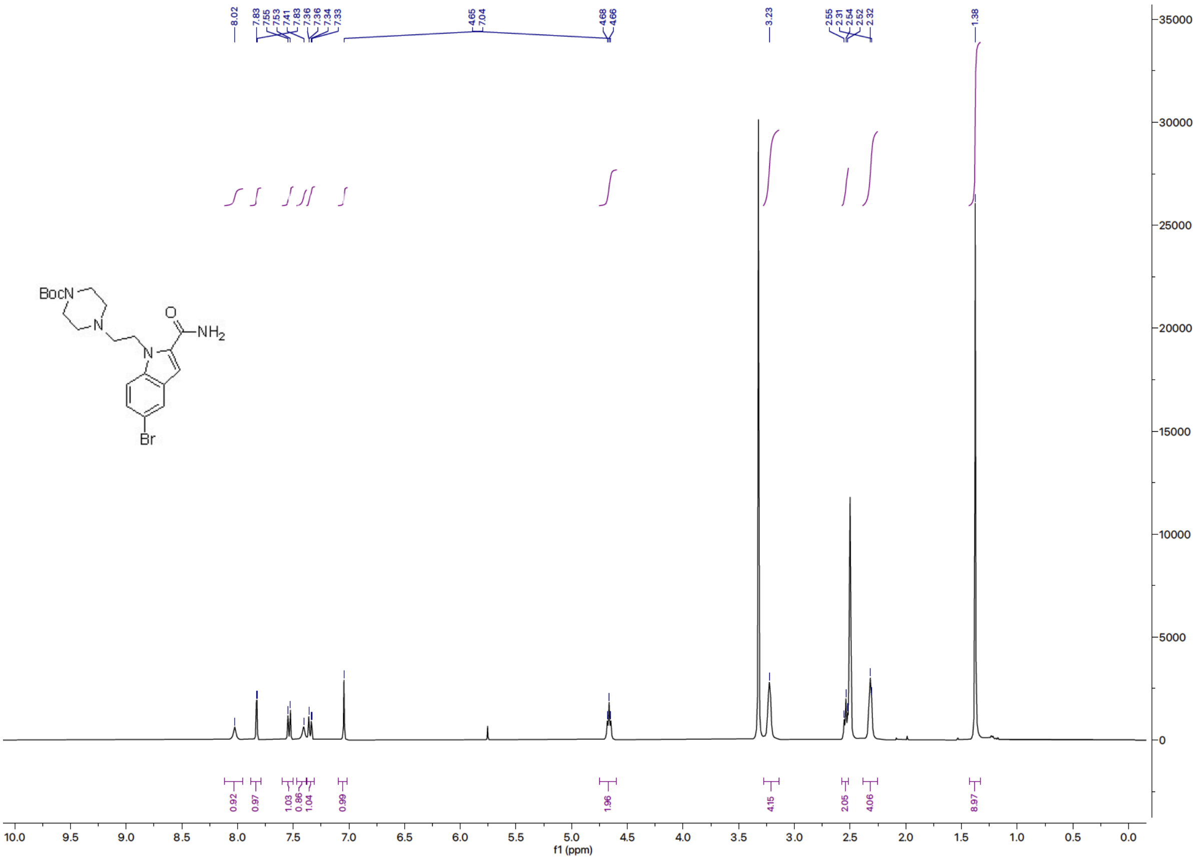

^{13}C NMR Spectra (101 MHz, (DMSO-d_6))

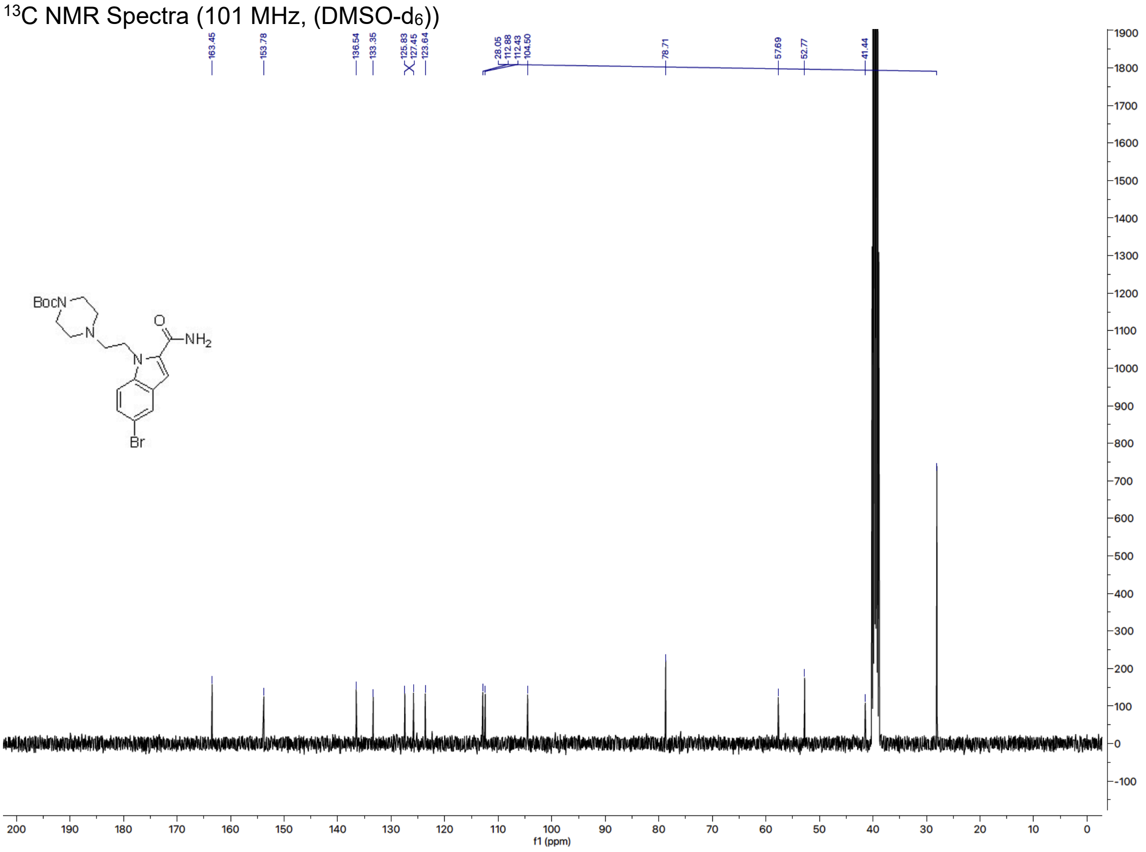

tert-Butyl-4-(2-(2-carbamoyl-5-(pyridin-4-yl)-1H-indol-1-yl)ethyl)piperazine-1-carboxylate (37)
^{1}H NMR Spectra (400 MHz, ($CDCl_3$))

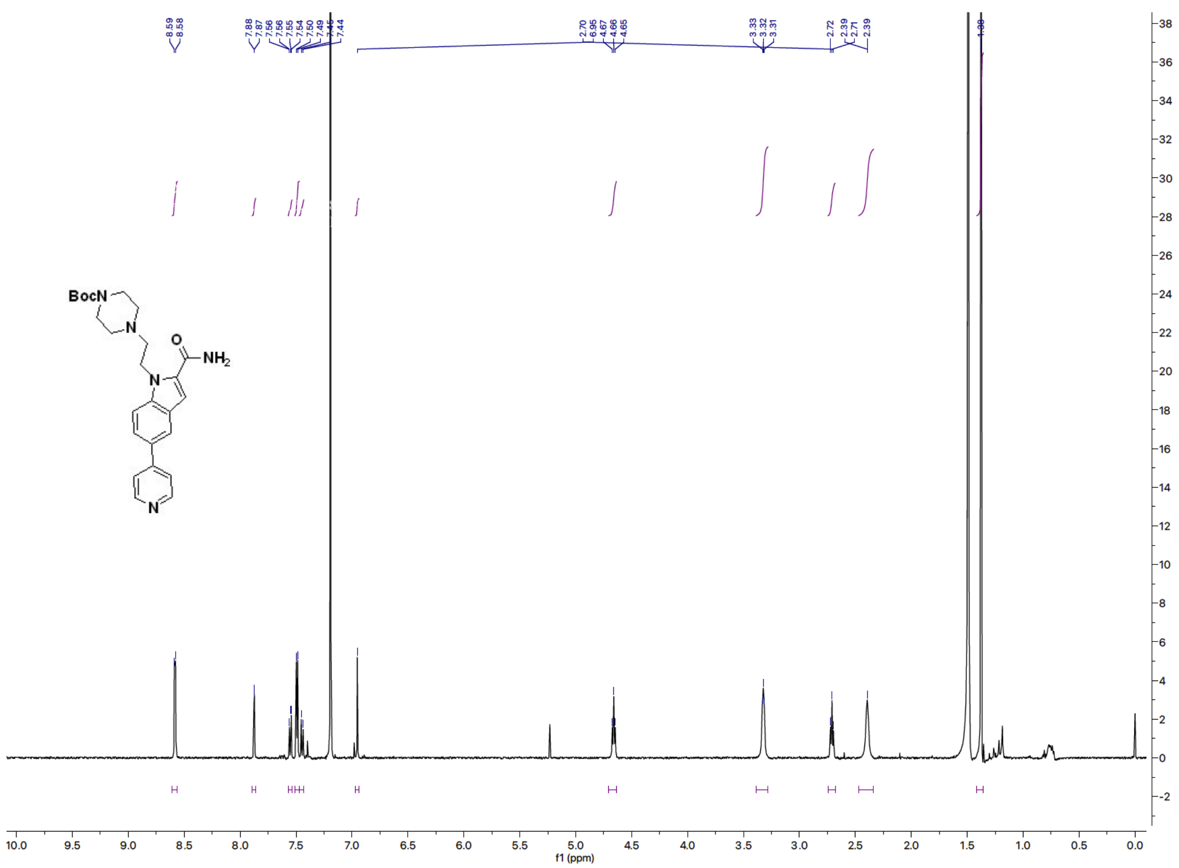

^{13}C NMR Spectra (101 MHz, ($CDCl_3$))

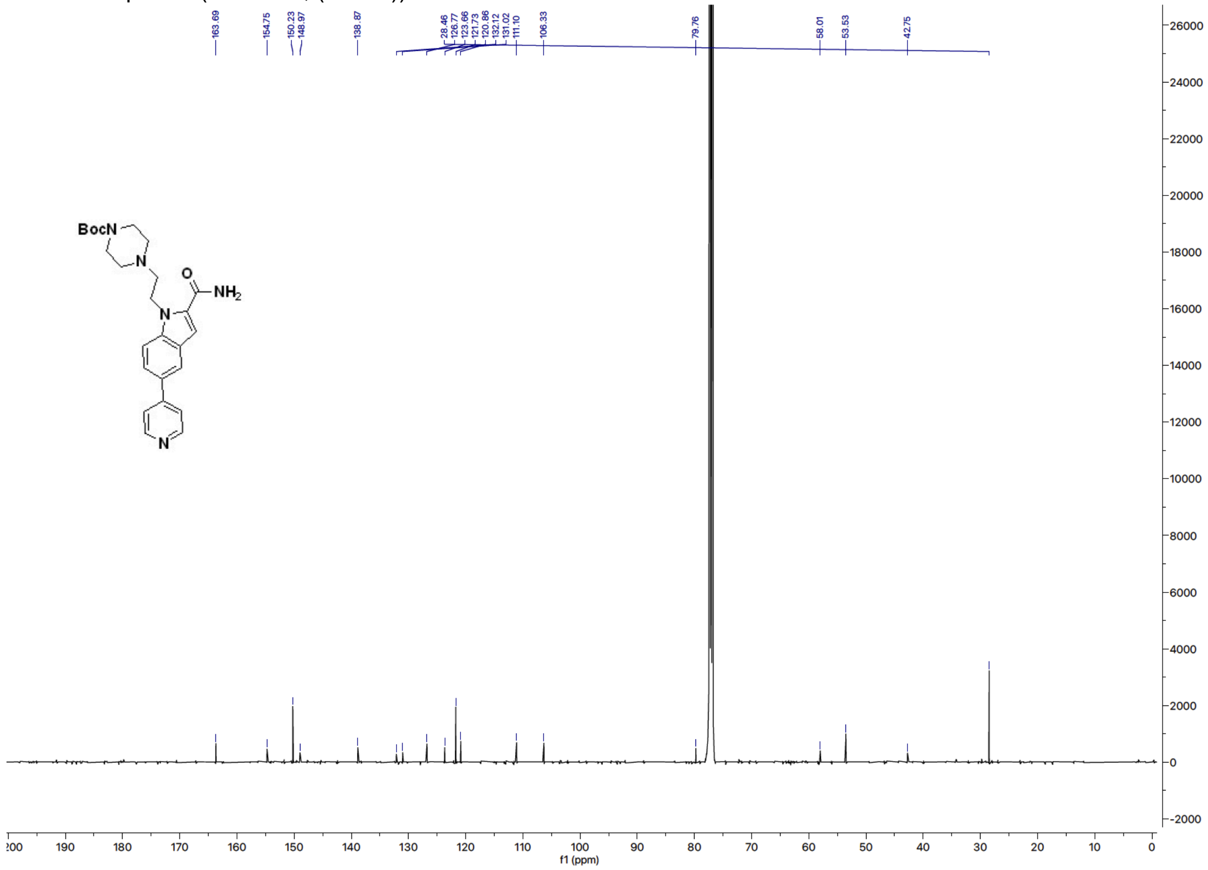

5-(5-Bromo-1H-indol-2-yl)-3-methyl-1,2,4-oxadiazole (40)

^{1}H NMR Spectra (500 MHz, ($CDCl_3$))

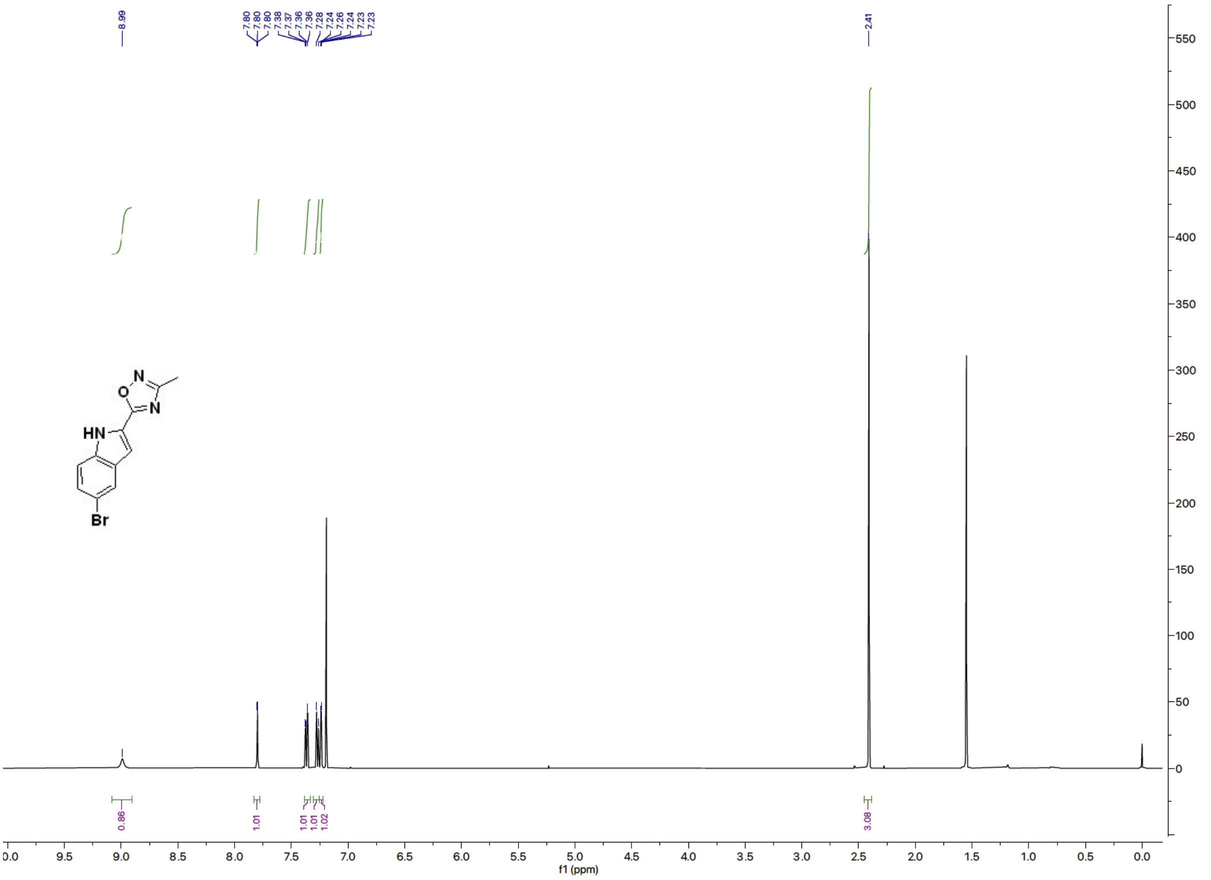

^{13}C NMR Spectra (126 MHz, ($CDCl_3$))

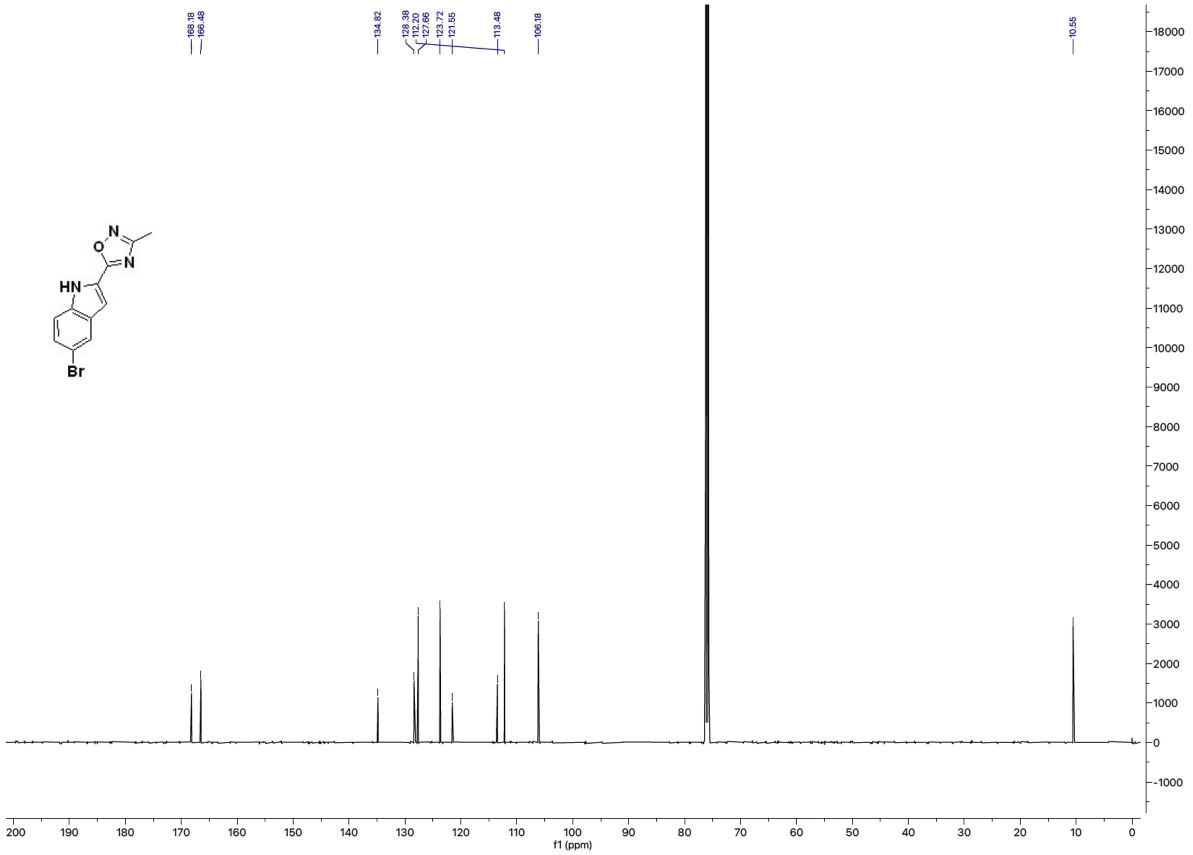

tert-Butyl-4-(2-(5-bromo-2-(3-methyl-1,2,4-oxadiazol-5-yl)-1H-indol-1-yl)ethyl)piperidine-1-carboxylate (44)

^{1}H NMR Spectra (400 MHz, ($CDCl_3$))

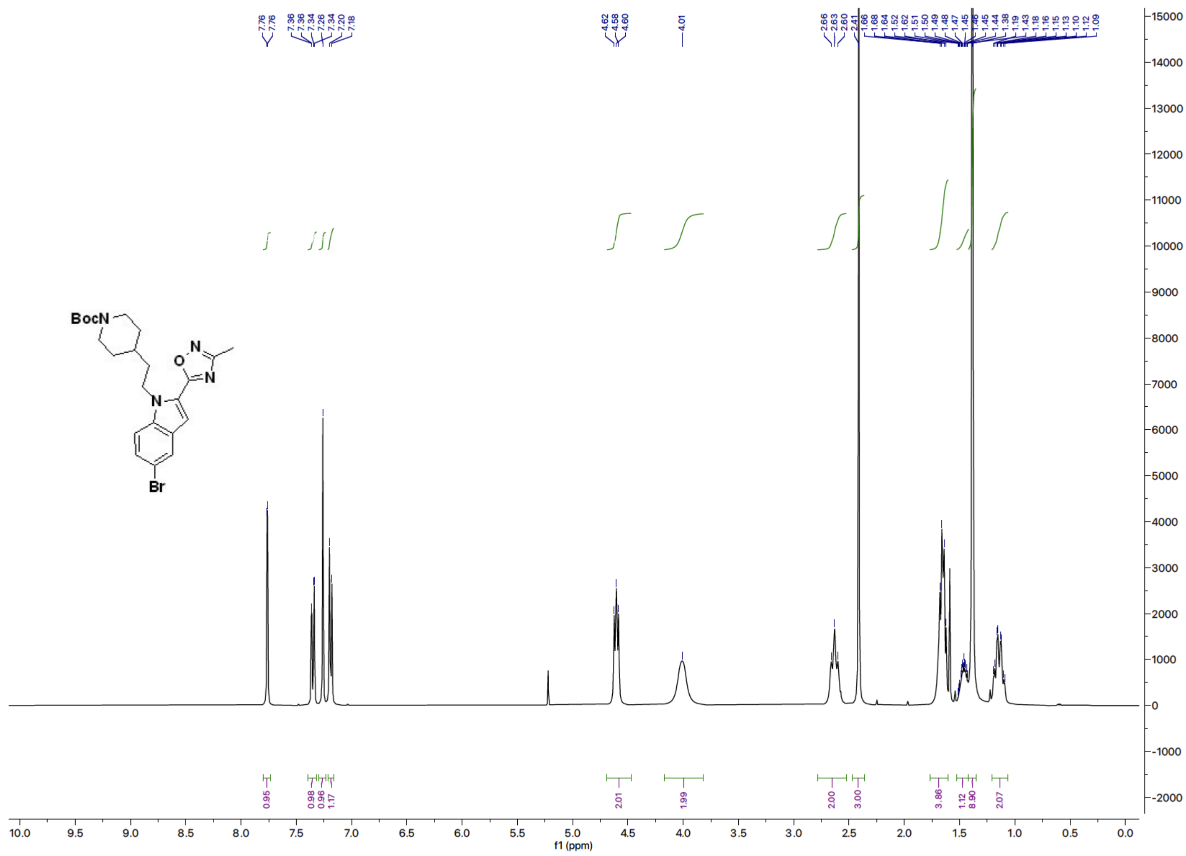

^{13}C NMR Spectra (101 MHz, ($CDCl_3$))

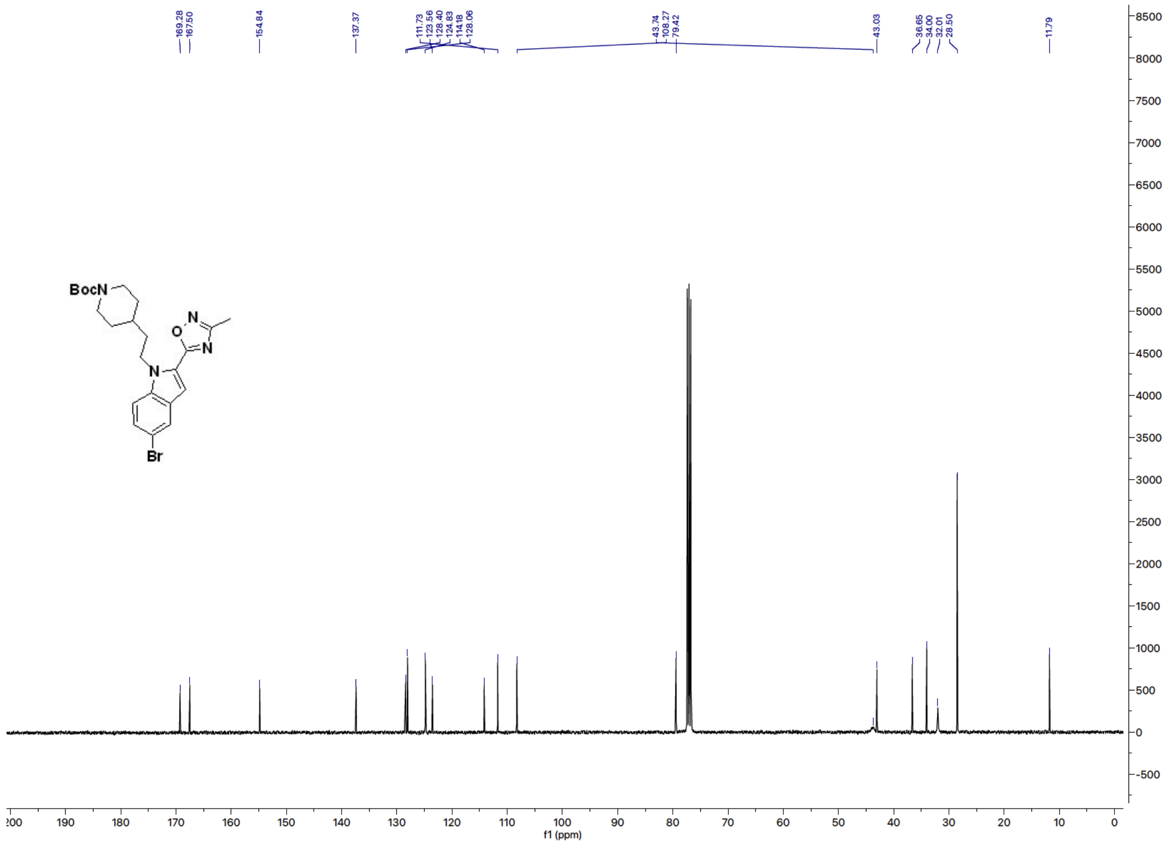

2-(5-Bromo-1H-indol-2-yl)-5-methyloxazole (41)

^{1}H NMR Spectra (400 MHz, ($CDCl_3$))

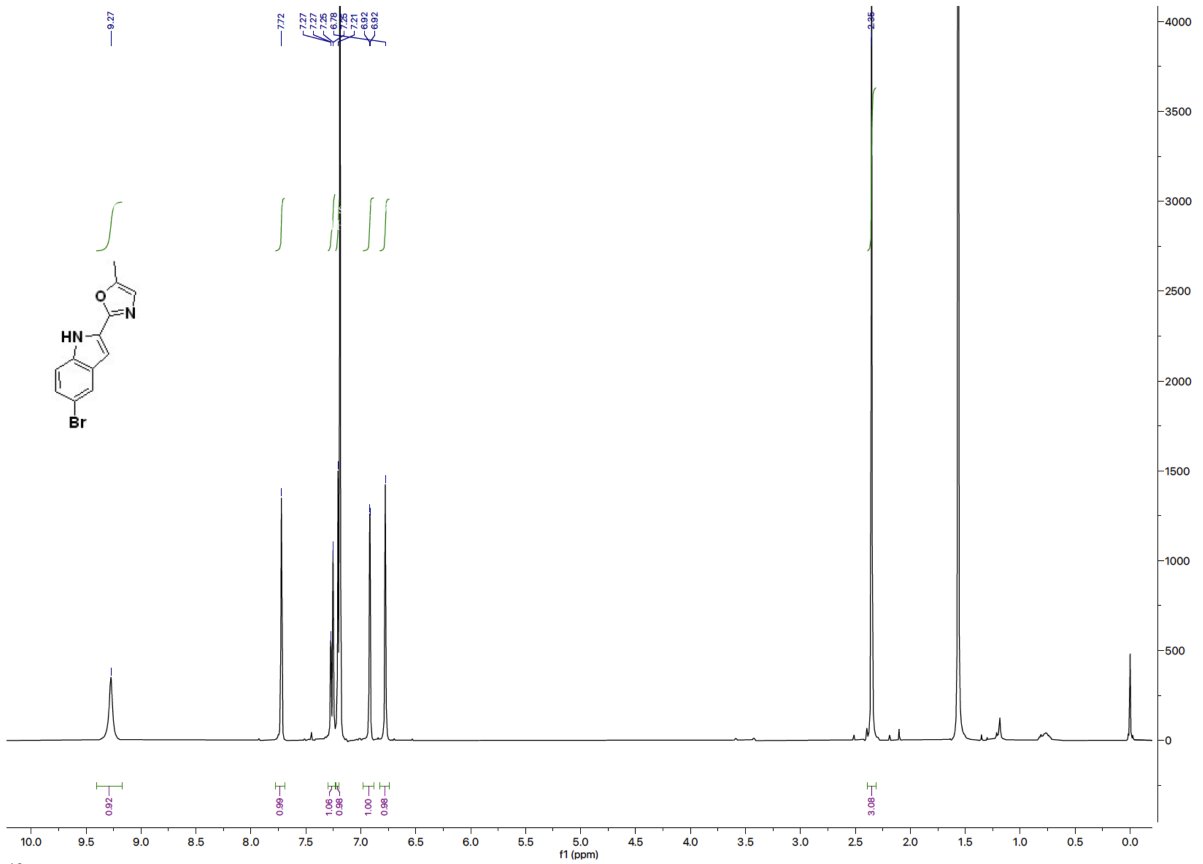

^{13}C NMR Spectra (101 MHz, ($CDCl_3$))

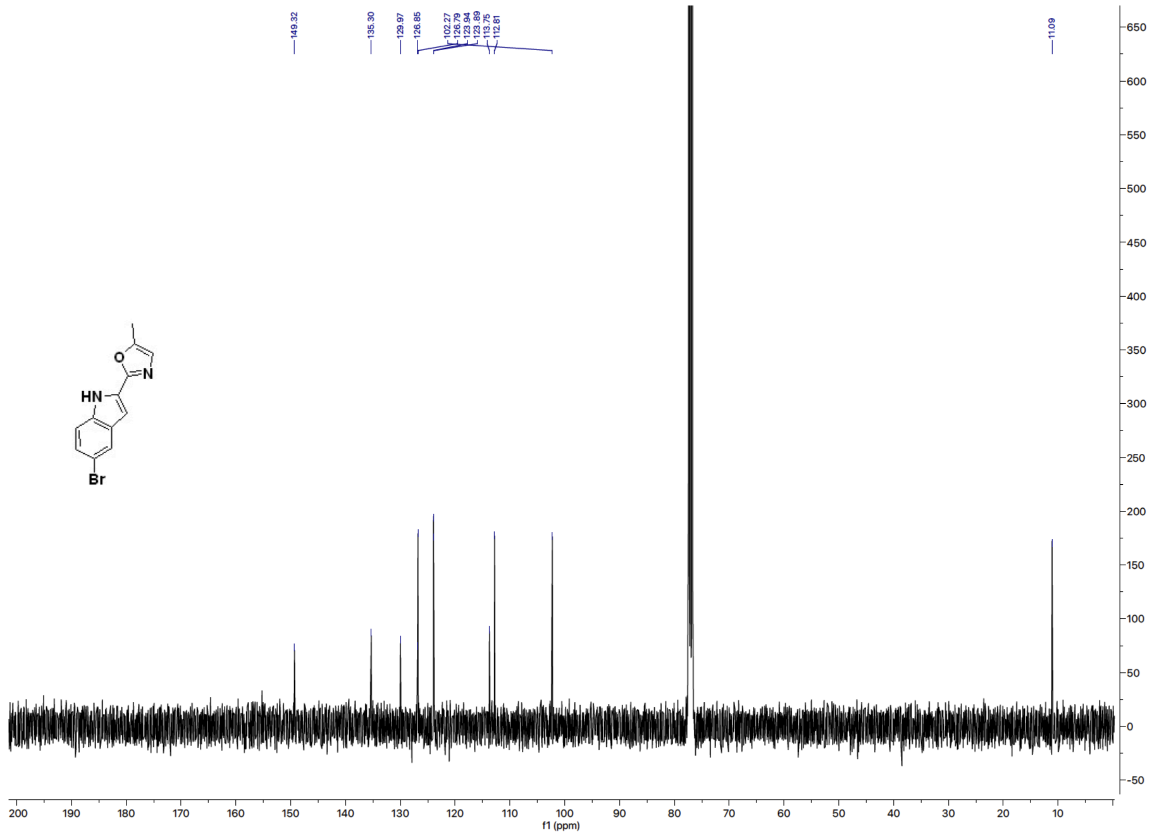

***tert*-Butyl-4-(2-(5-bromo-2-(5-methyloxazol-2-yl)-1*H*-indol-1-yl)ethyl)piperidine-1-carboxylate (45)**

^{1}H NMR Spectra (400 MHz, ($CDCl_3$))

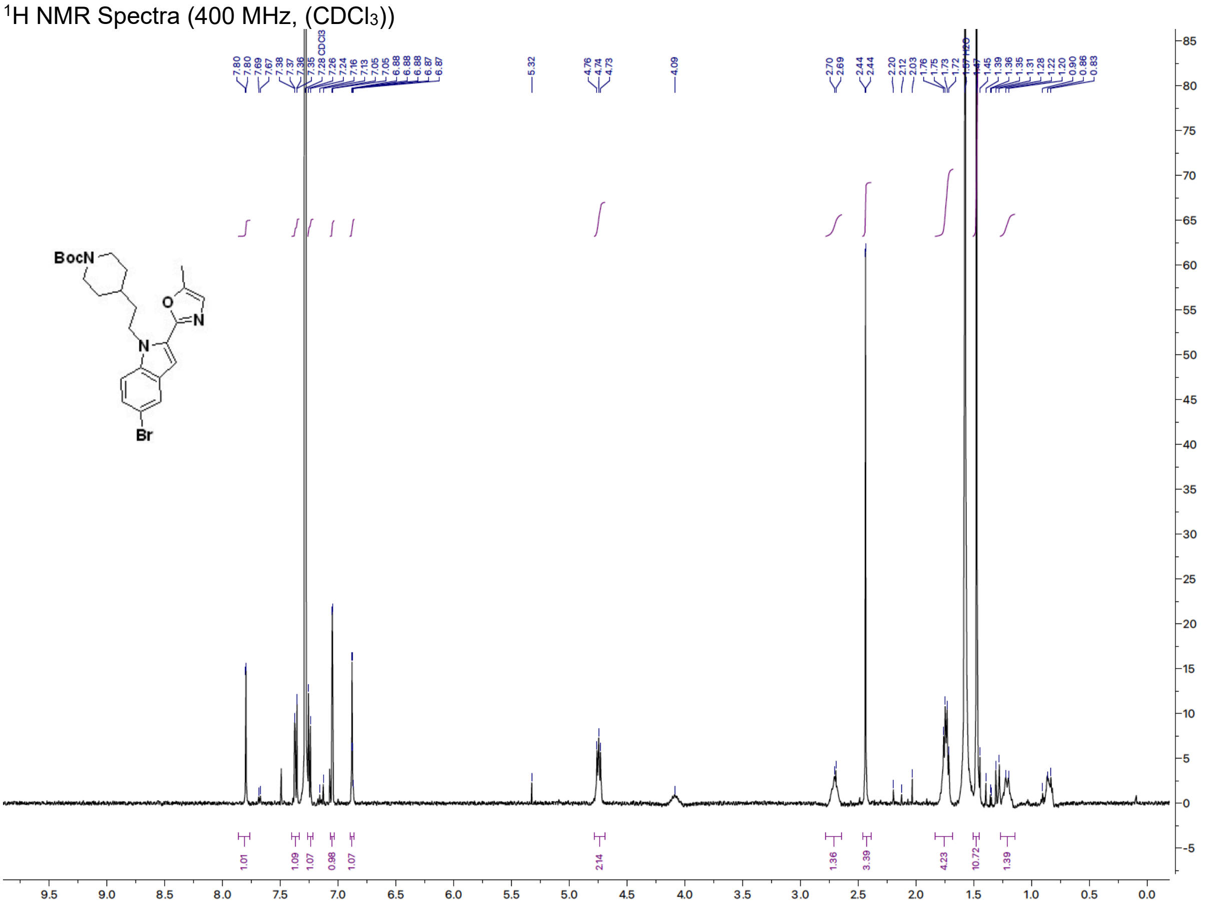

^{13}C NMR Spectra (126 MHz, ($CDCl_3$))

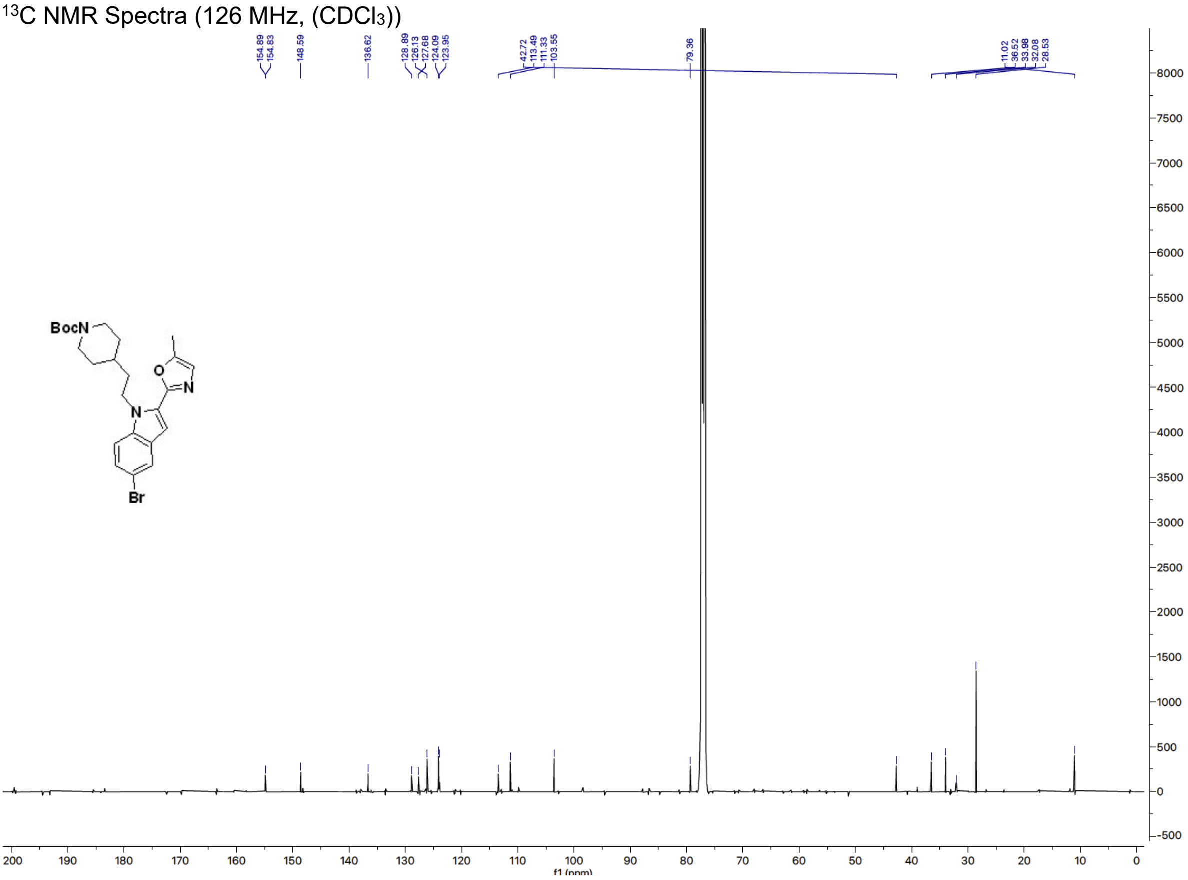

***tert*-butyl-4-(2-(2-(5-methyloxazol-2-yl)-5-(pyridin-4-yl)-1*H*-indol-1-yl)ethyl)piperidine-1-carboxylate** (**49**)

^{1}H NMR Spectra (500 MHz, (DMSO-d_6))

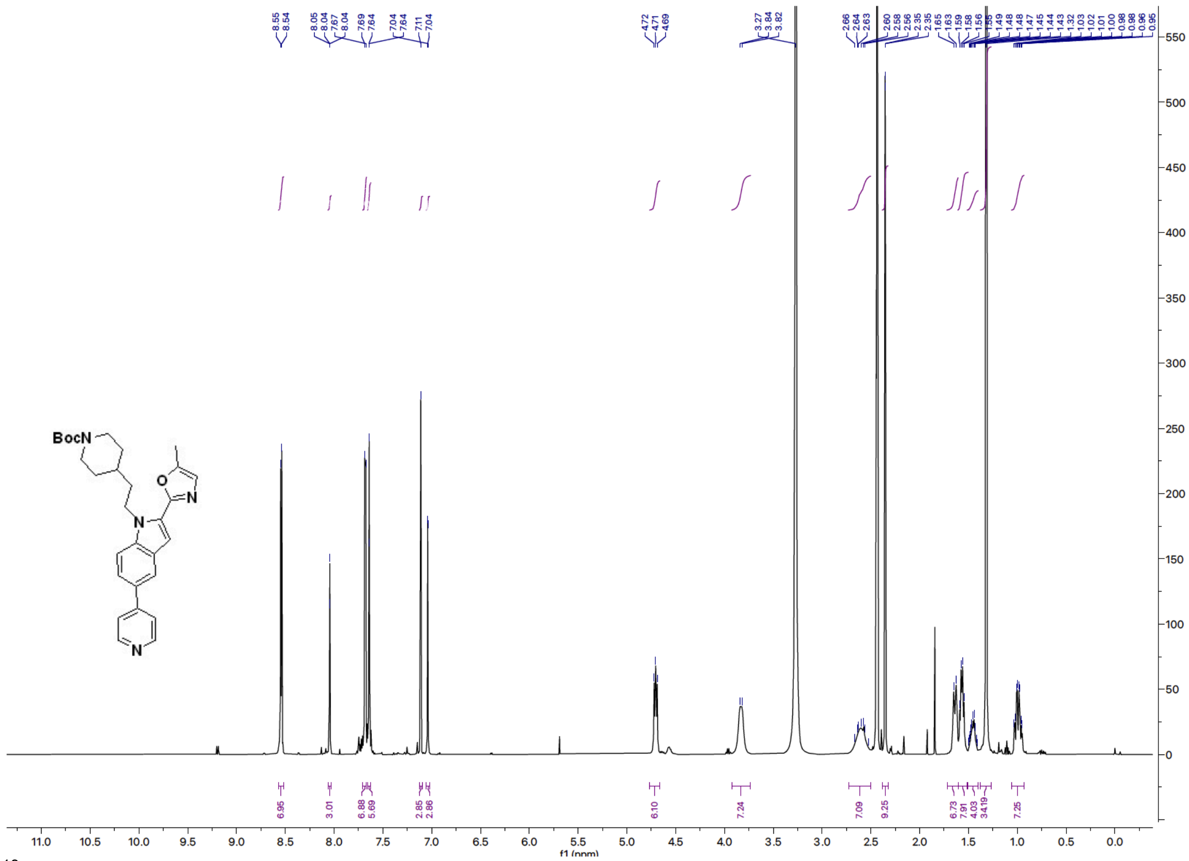

^{13}C NMR Spectra (126 MHz, (DMSO-d$_6$))

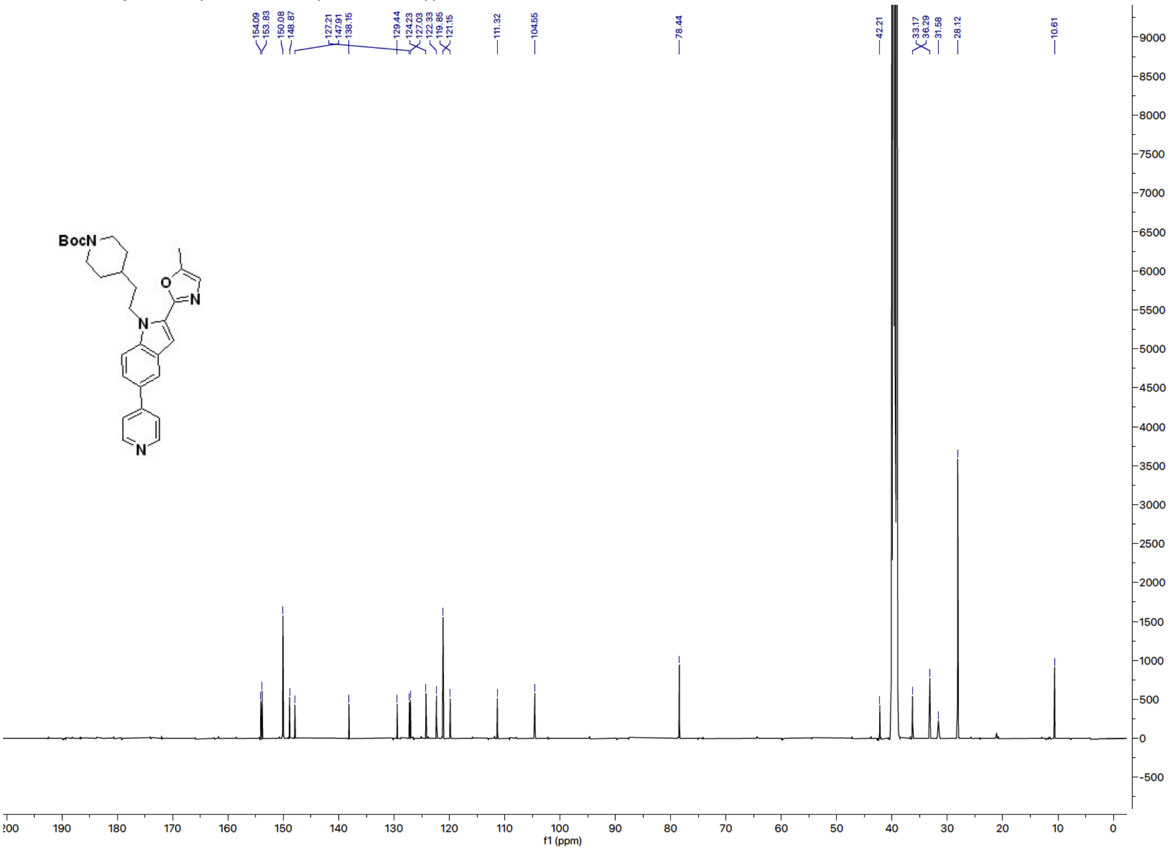

1-(5-Bromo-1H-indol-2-yl)butan-1-one (42)

^{1}H NMR Spectra (500 MHz, (CDCl$_3$))

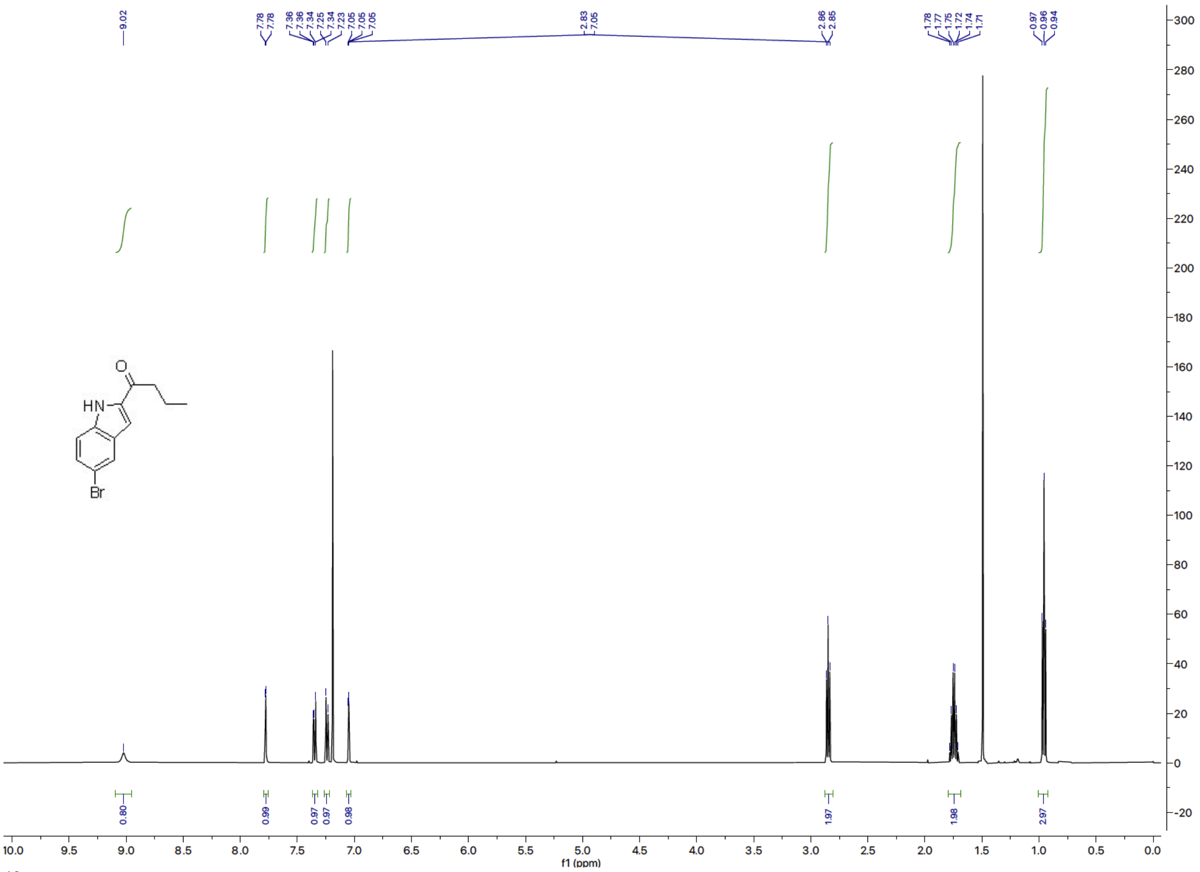

^{13}C NMR Spectra (126 MHz, ($CDCl_3$))

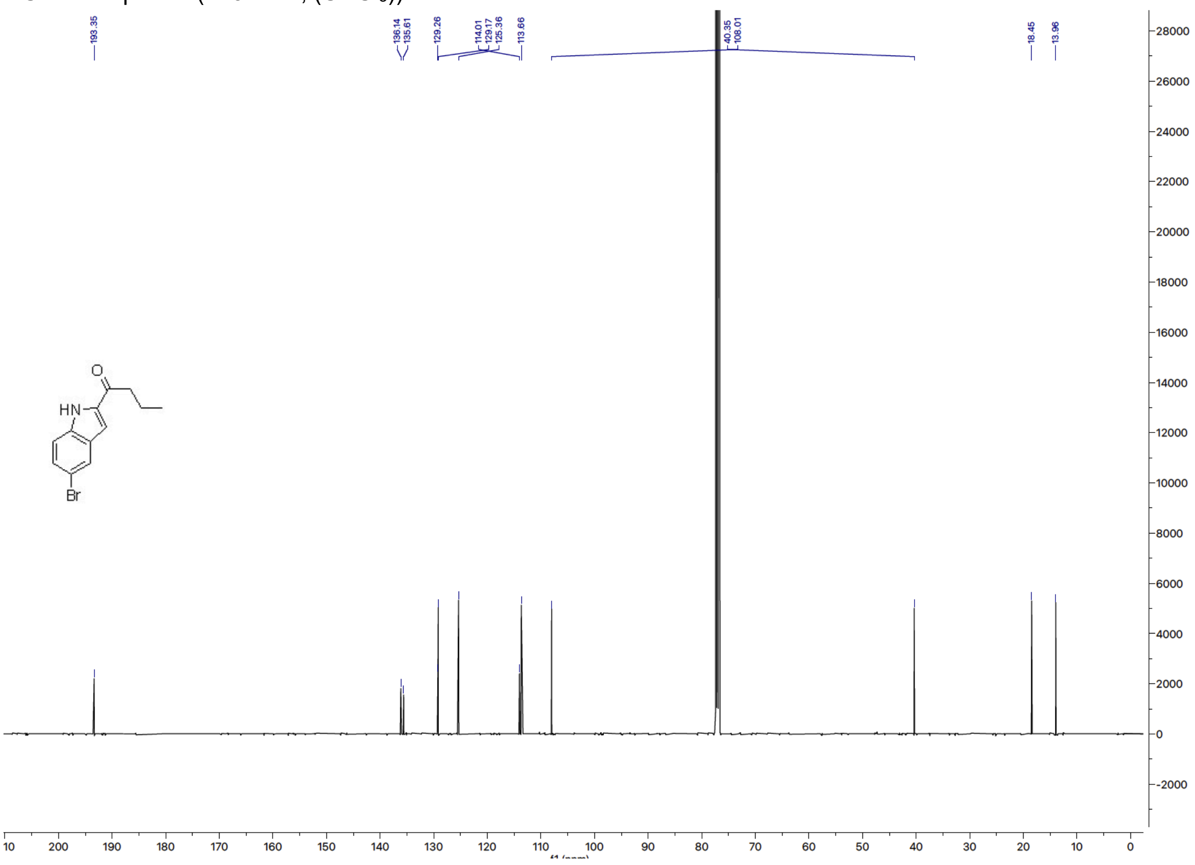

***tert*-Butyl 4-(2-(5-bromo-2-butyryl-1*H*-indol-1-yl)ethyl)piperidine-1-carboxylate** (**47**)
^{1}H NMR Spectra (500 MHz, ($CDCl_3$))

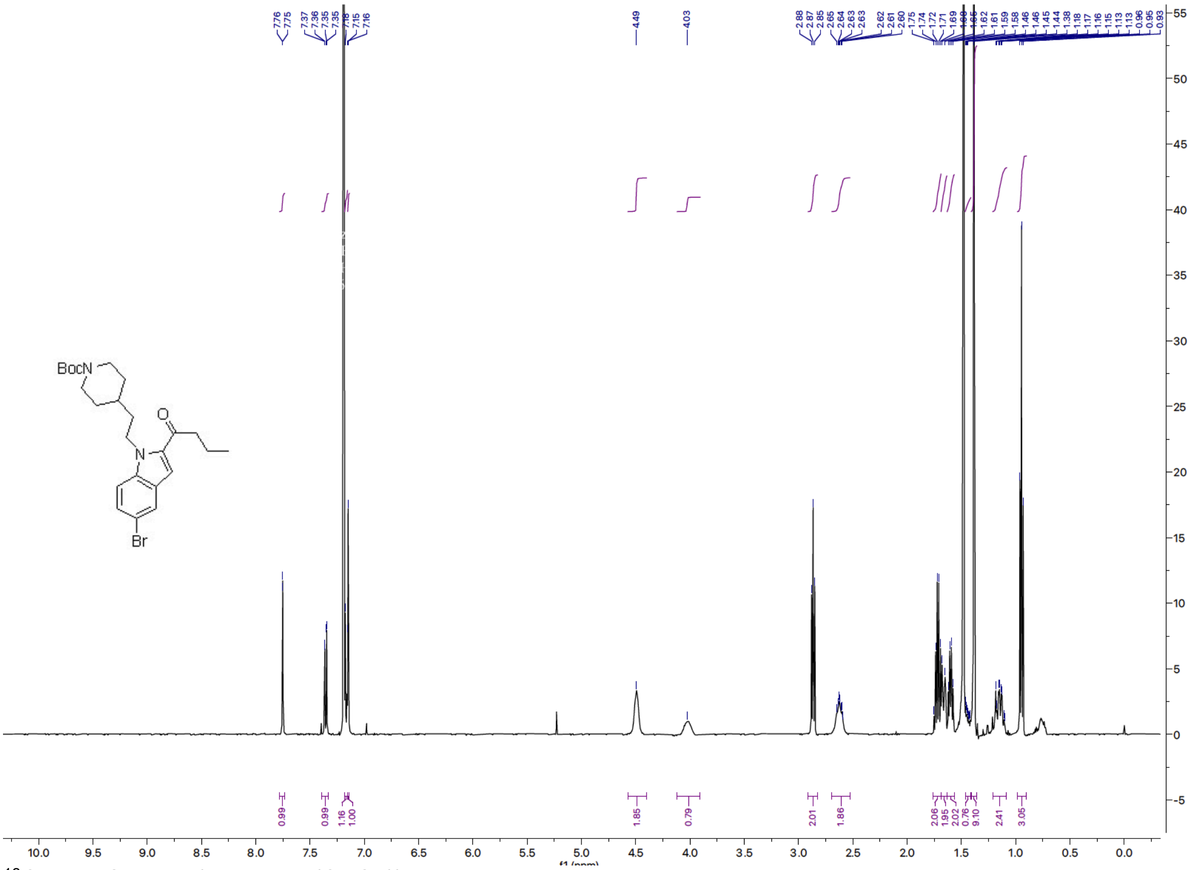

^{13}C NMR Spectra (126 MHz, (CDCl$_3$))

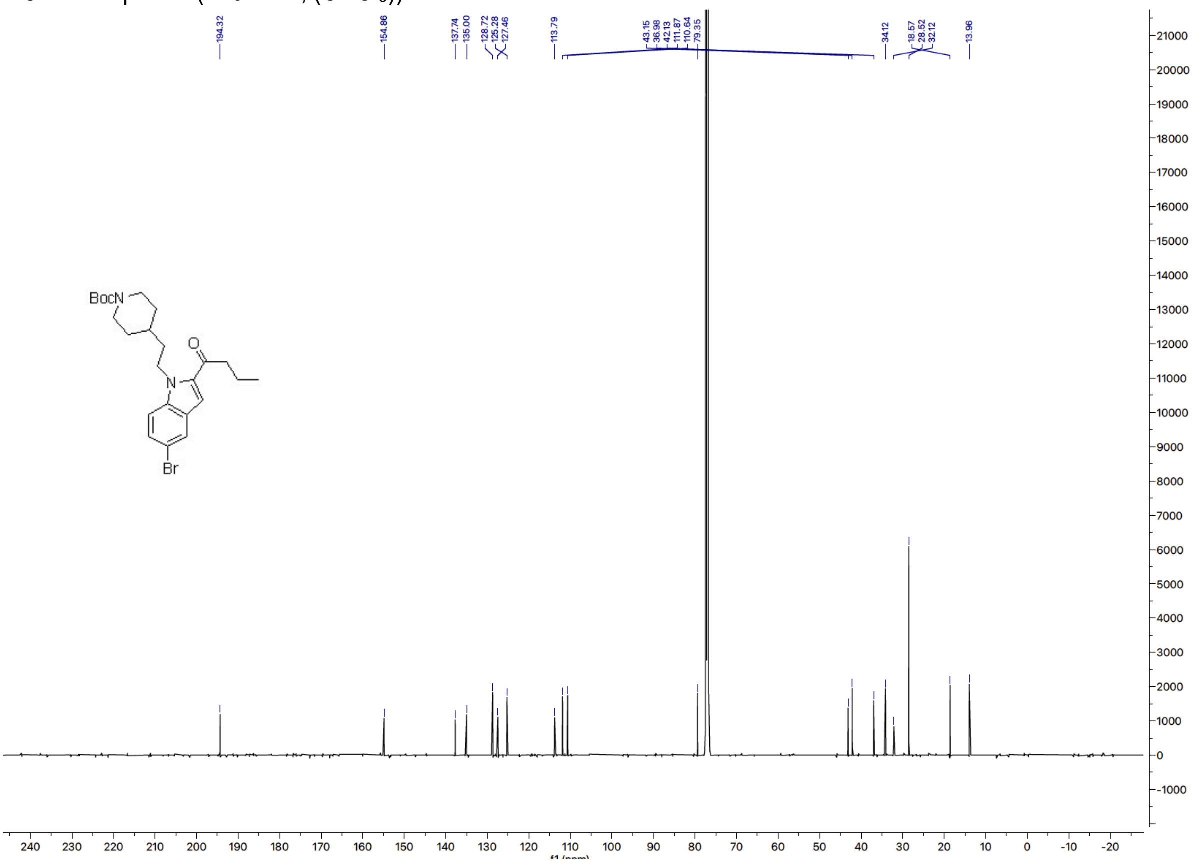

tert-Butyl-4-(2-(2-butyryl-5-(pyridin-4-yl)-1H-indol-1-yl)ethyl)piperidine-1-carboxylate (51)

^{1}H NMR Spectra (500 MHz, (DMSO-d$_6$))

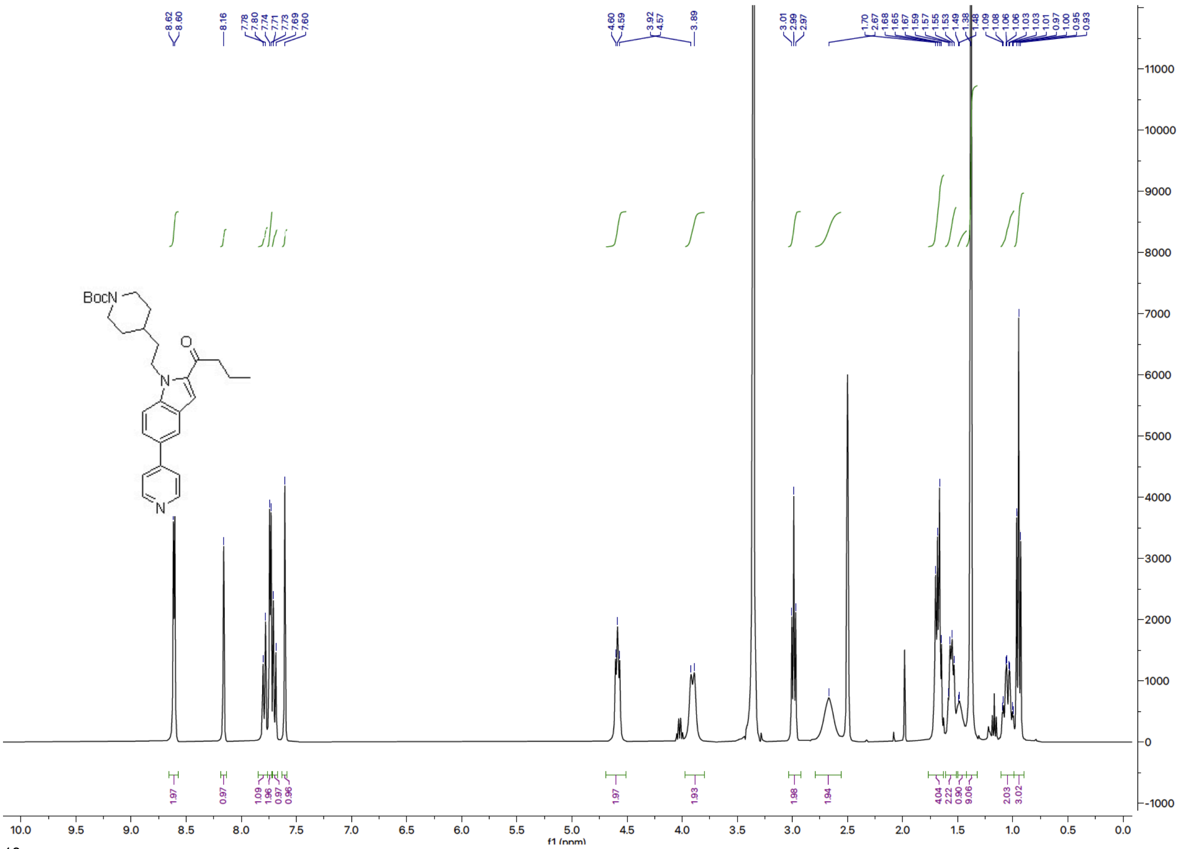

^{13}C NMR Spectra (126 MHz, (DMSO-d$_6$))

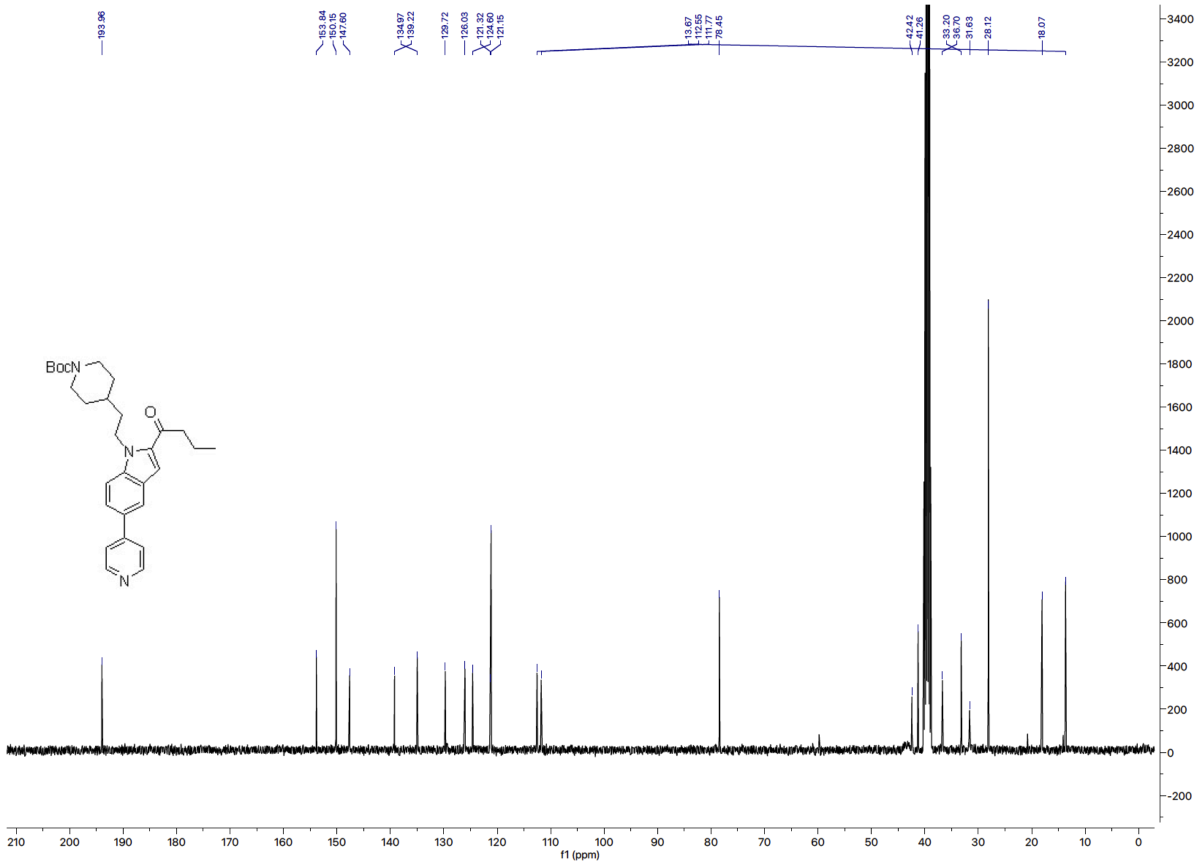

2-(5-Bromo-1H-indol-2-yl)-5-methyl-1,3,4-oxadiazole (43)

^{1}H NMR Spectra (500 MHz, (DMSO-d$_6$))

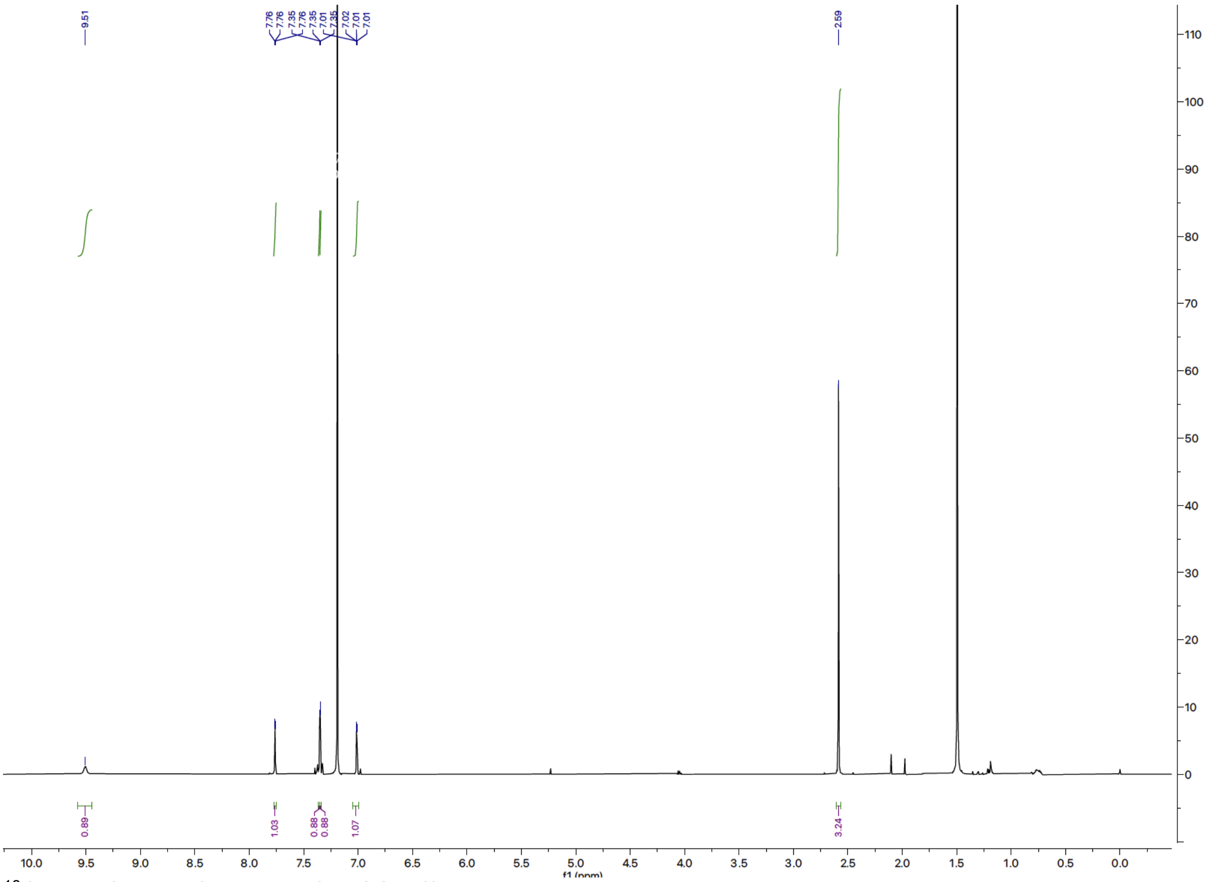

^{13}C NMR Spectra (126 MHz, (DMSO-d_6))

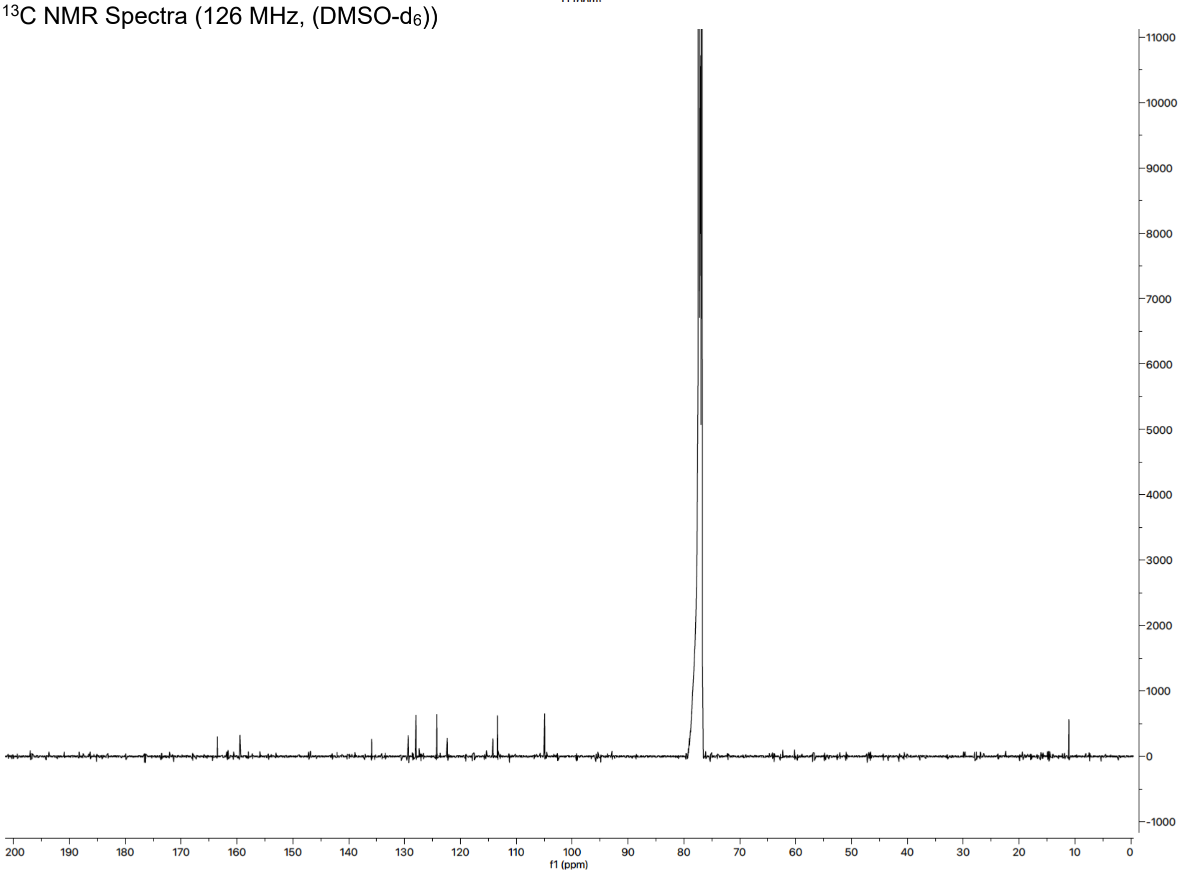

Methyl 3-(pyridin-4-yl)benzoate (3)

^1H NMR Spectra (500 MHz, ($CDCl_3$))

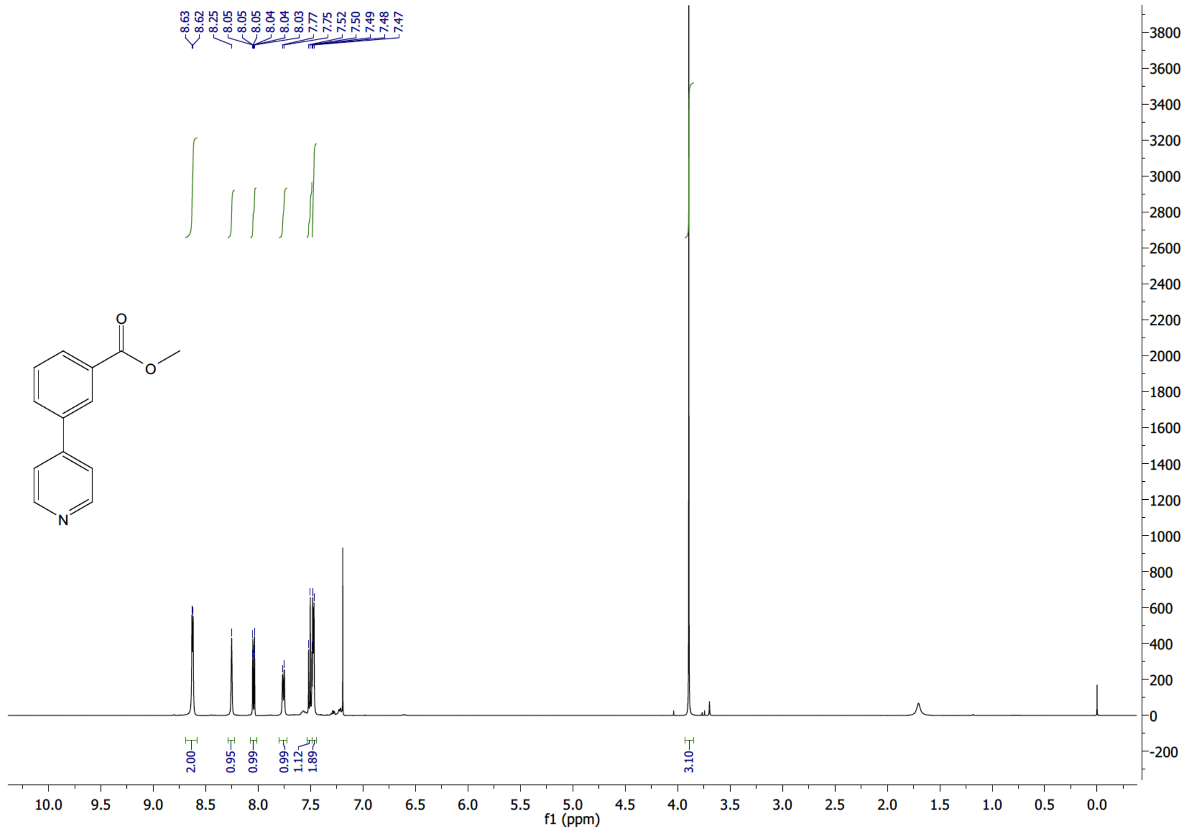

^{13}C NMR Spectra (126 MHz, ($CDCl_3$))

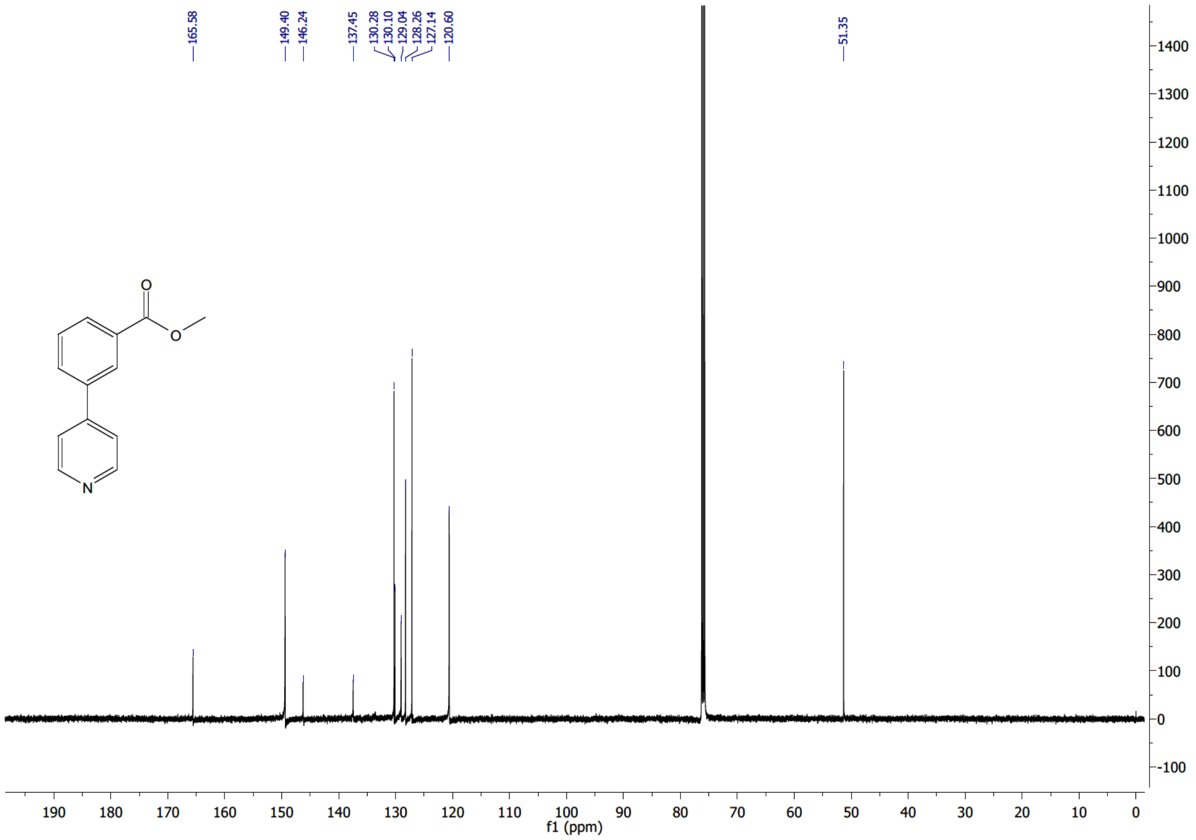

4-(4-methoxyphenyl)pyridine (**2**)

^{1}H NMR Spectra (500 MHz, (DMSO-d_6))

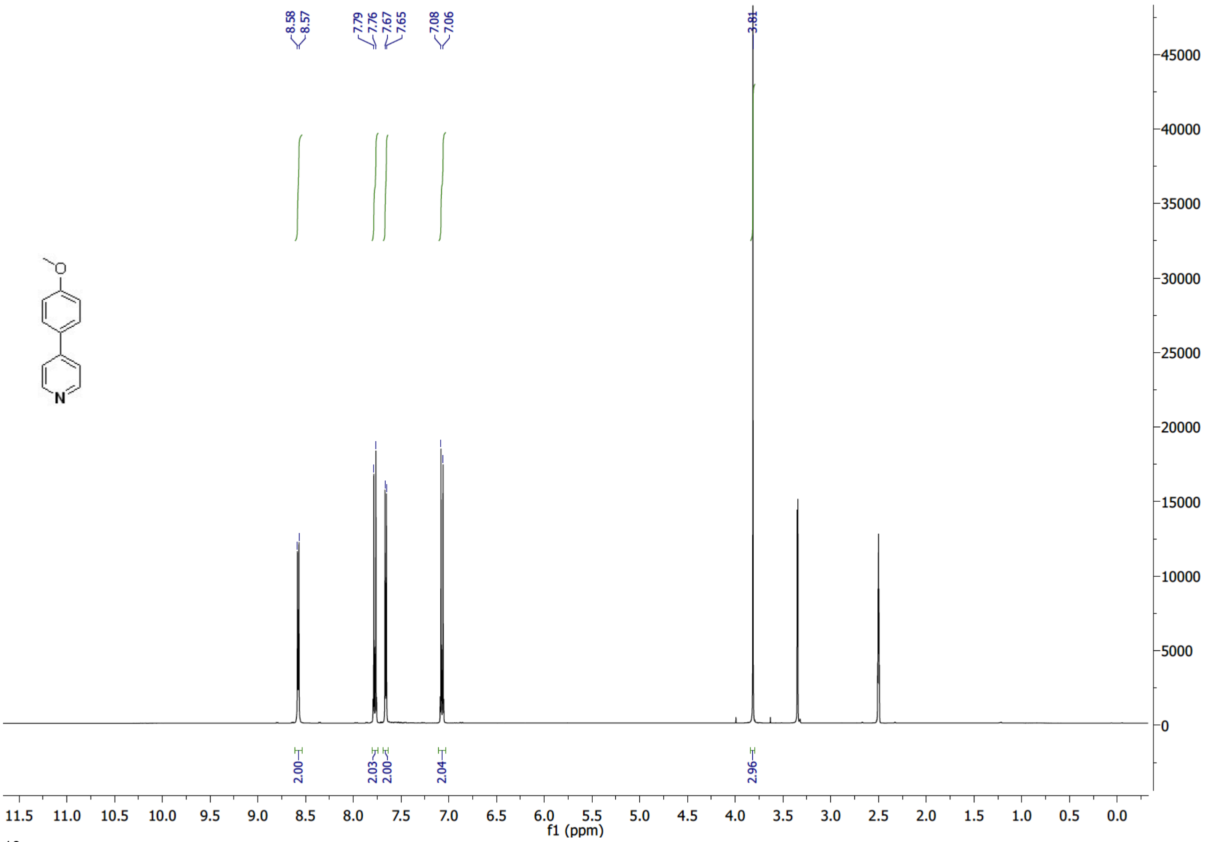

^{13}C NMR Spectra (101 MHz, (DMSO-d_6))

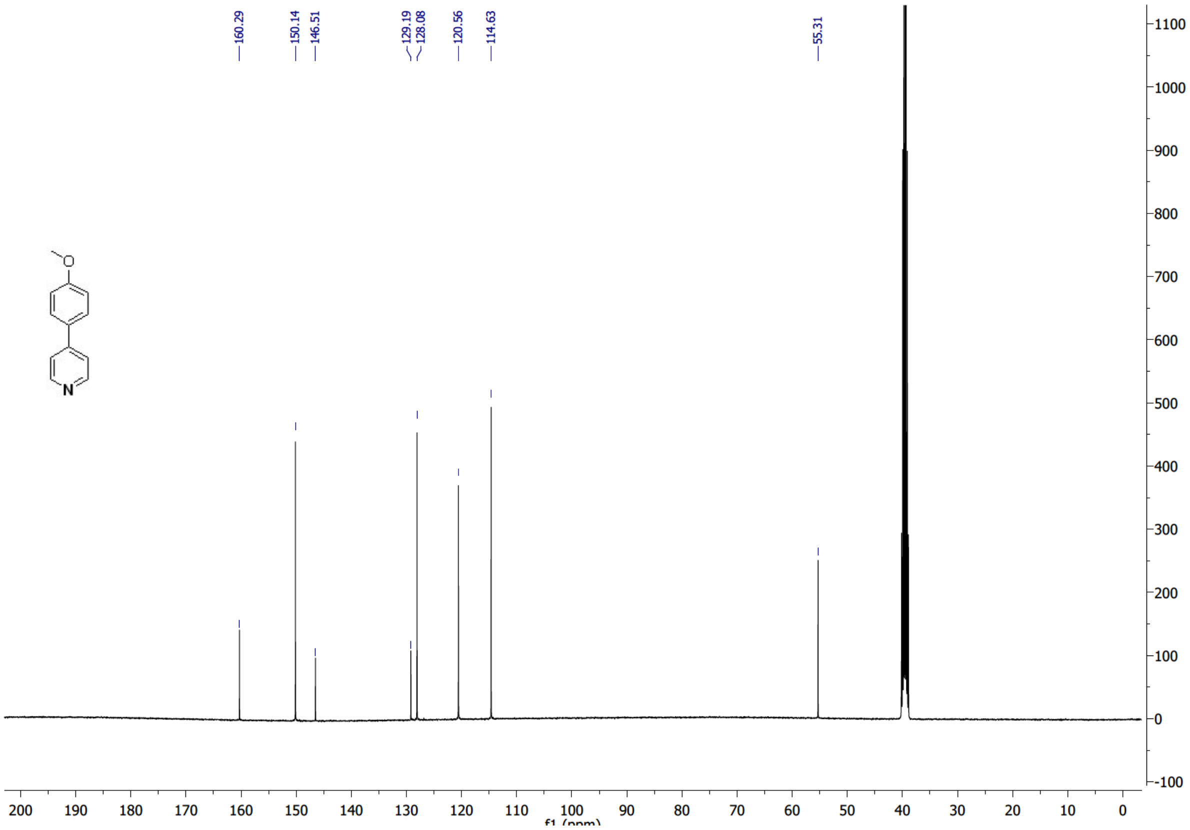

Ethyl 5-(pyridin-4-yl)-1H-indole-2-carboxylate (4)

^{1}H NMR Spectra (500 MHz, ($CDCl_3$))

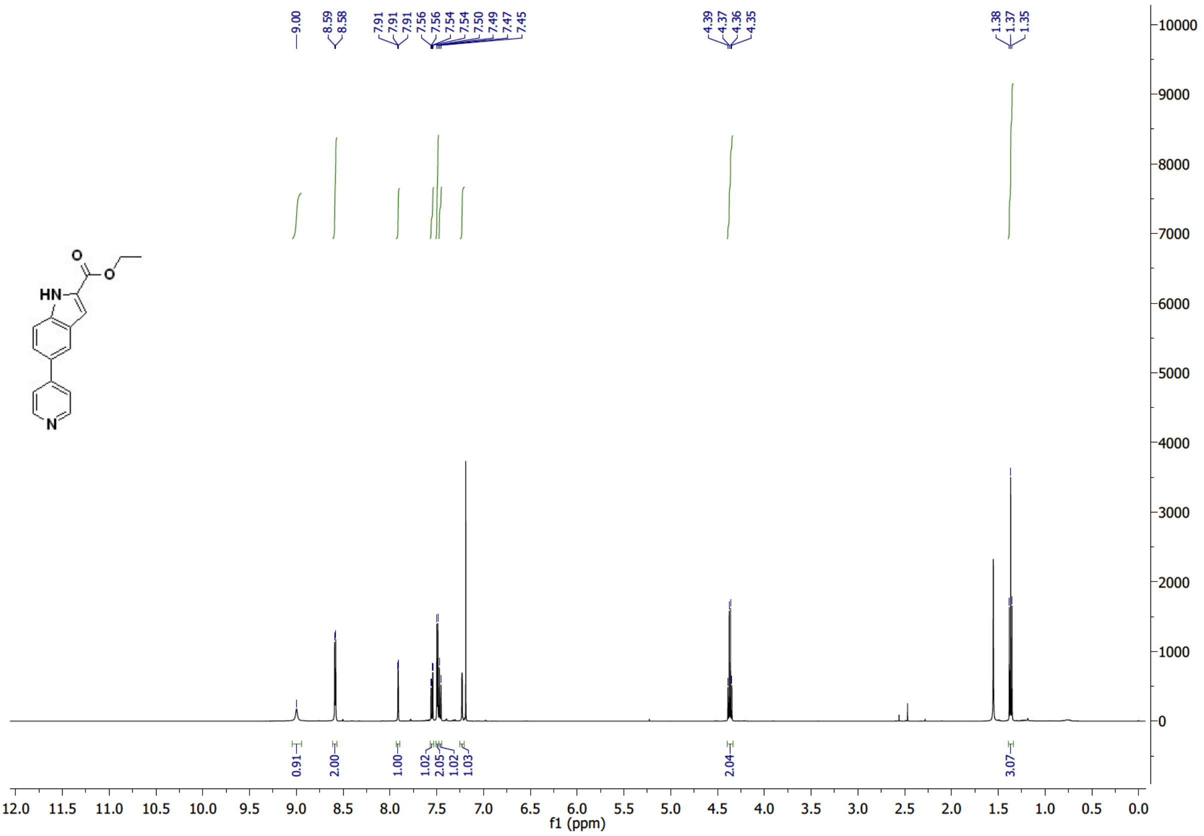

^{13}C NMR Spectra (126 MHz, ($CDCl_3$))

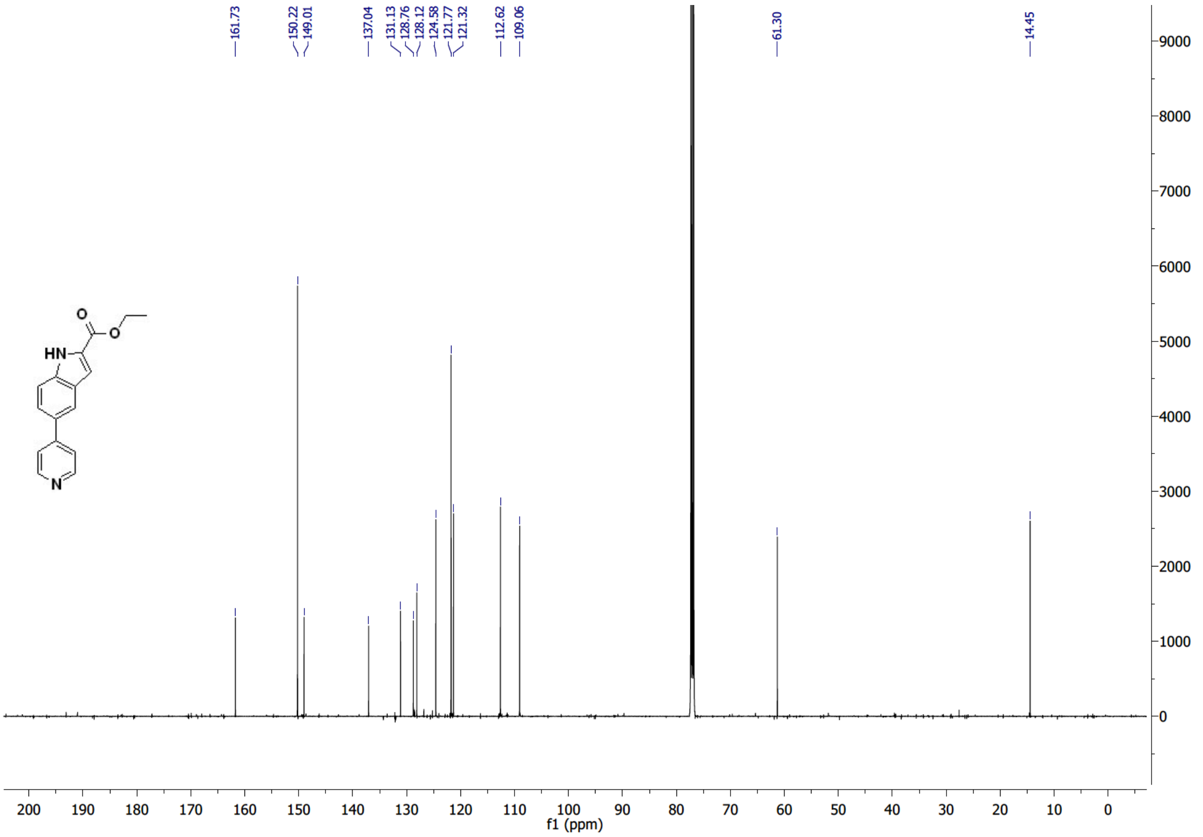

Ethyl-1-benzyl-5-(pyridin-4-yl)-1H-indole-2-carboxylate (5)

^{1}H NMR Spectra (500 MHz, ($CDCl_3$))

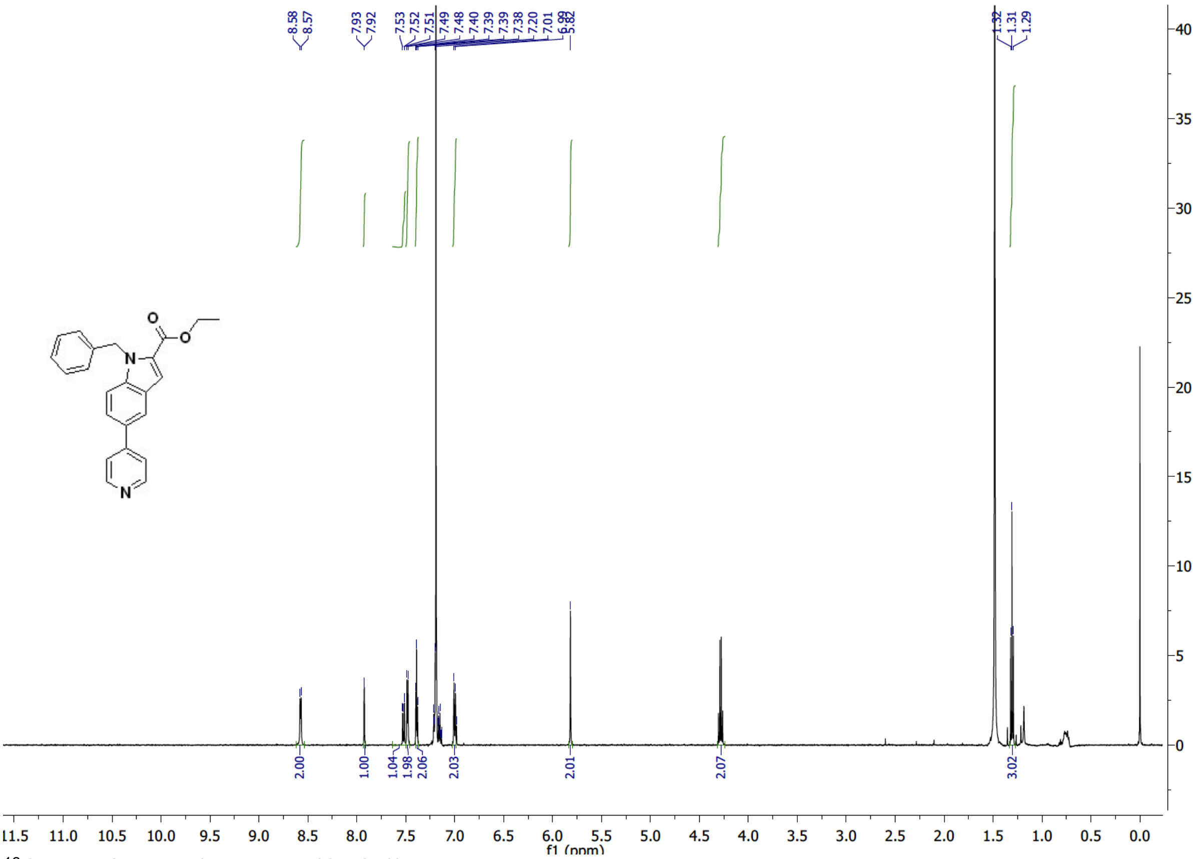

^{13}C NMR Spectra (126 MHz, ($CDCl_3$))

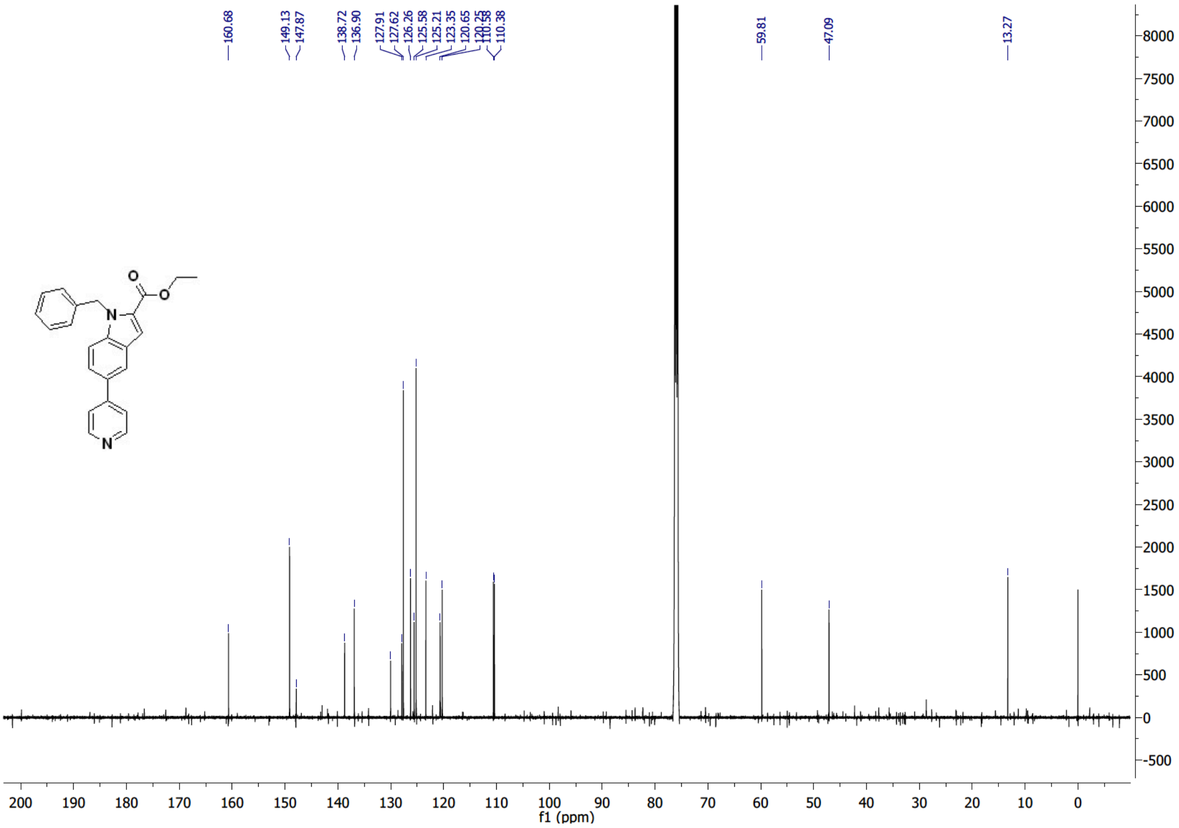

Ethyl 1-benzoyl-5-(pyridin-4-yl)-1H-indole-2-carboxylate (6)

^{1}H NMR Spectra (500 MHz, ($CDCl_3$))

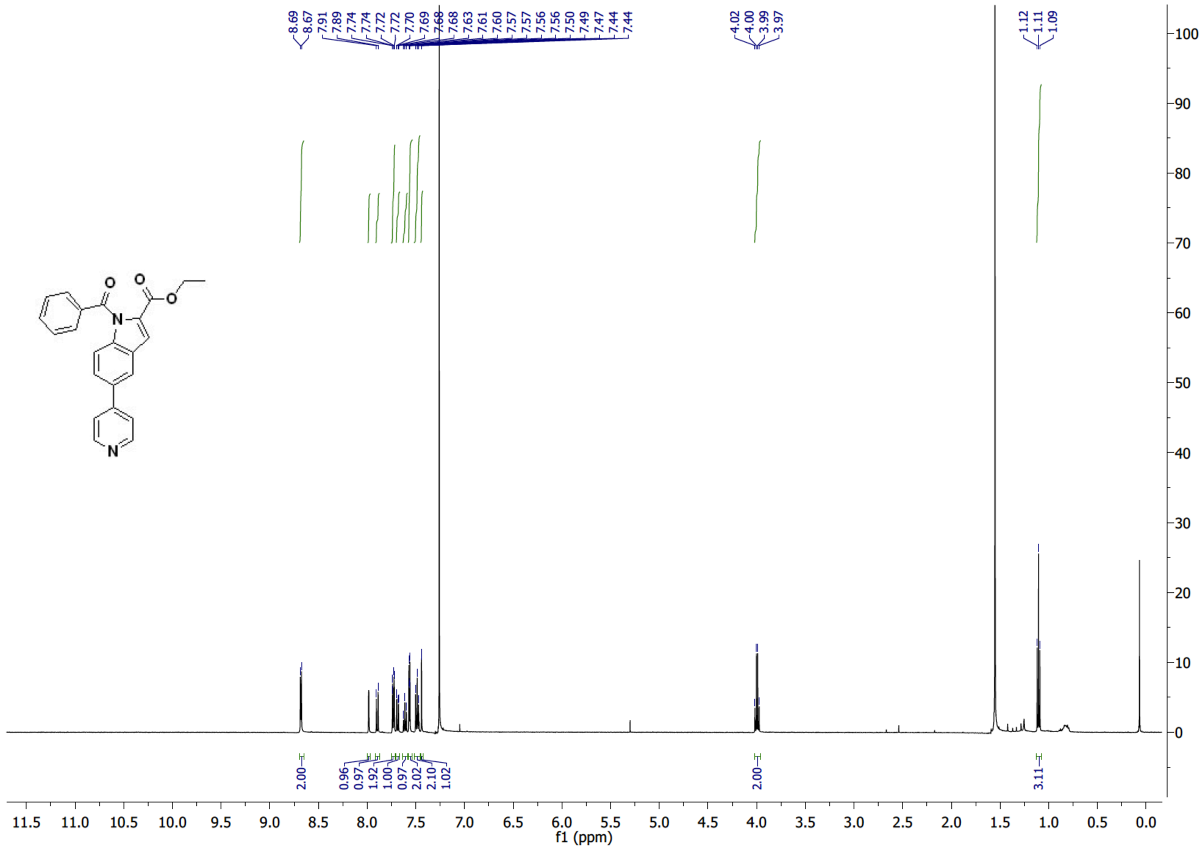

^{13}C NMR Spectra (126 MHz, ($CDCl_3$))

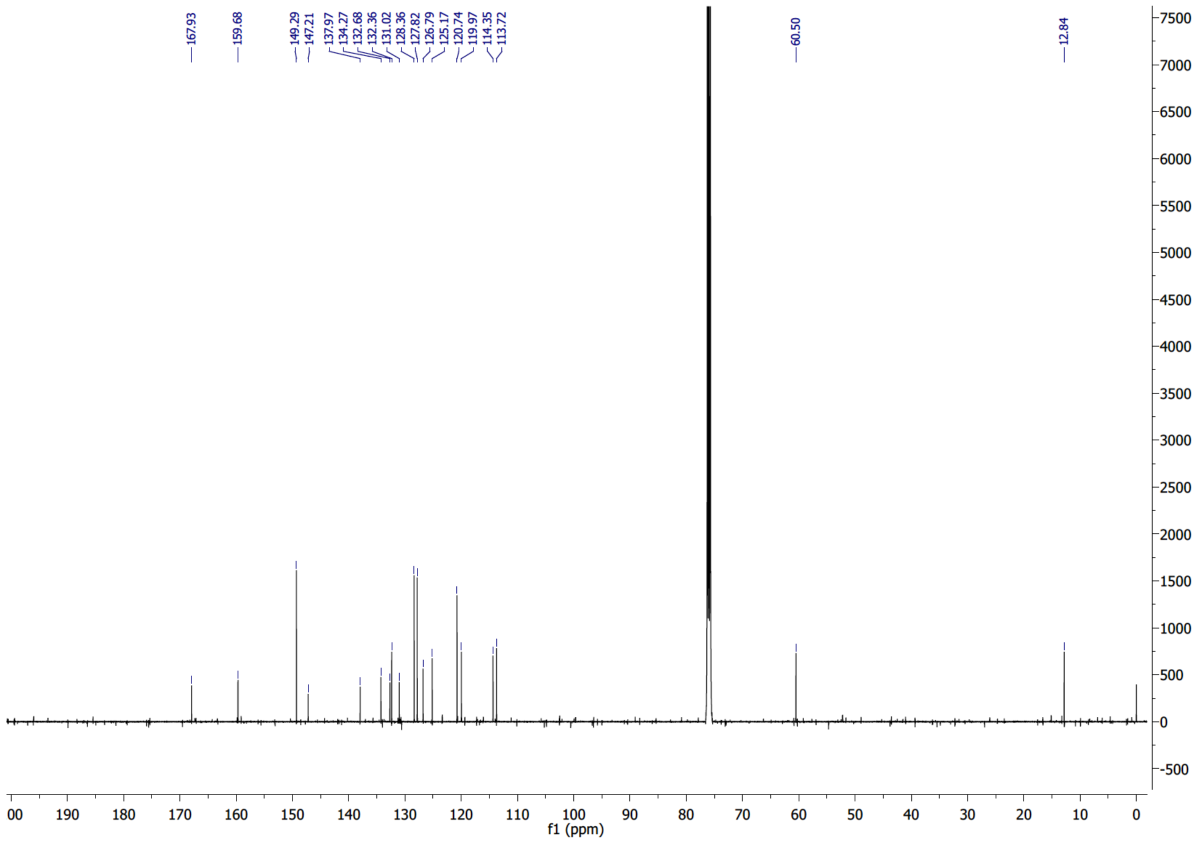

Ethyl 1-(cyclohexylmethyl)-5-(pyridin-4-yl)-1H-indole-2-carboxylate (**7**)

^{1}H NMR Spectra (500 MHz, ($CDCl_3$))

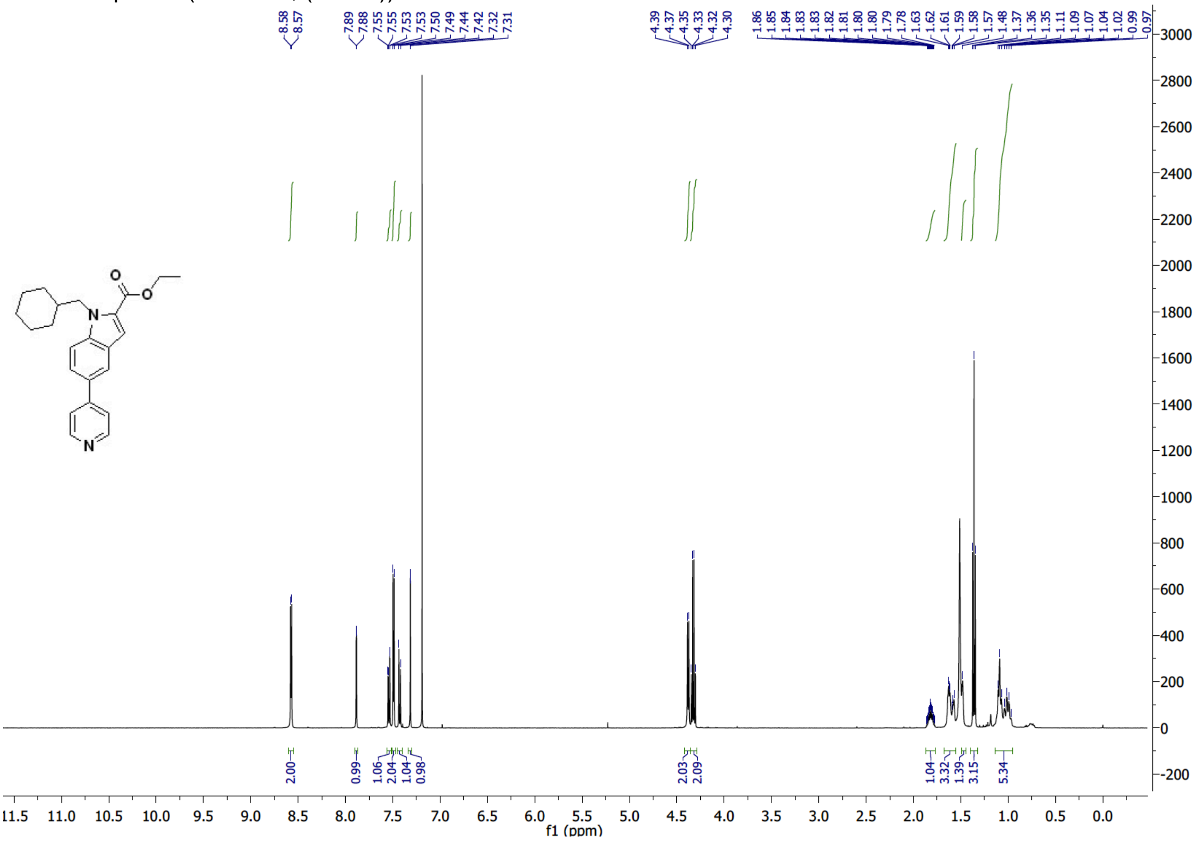

^{13}C NMR Spectra (126 MHz, ($CDCl_3$))

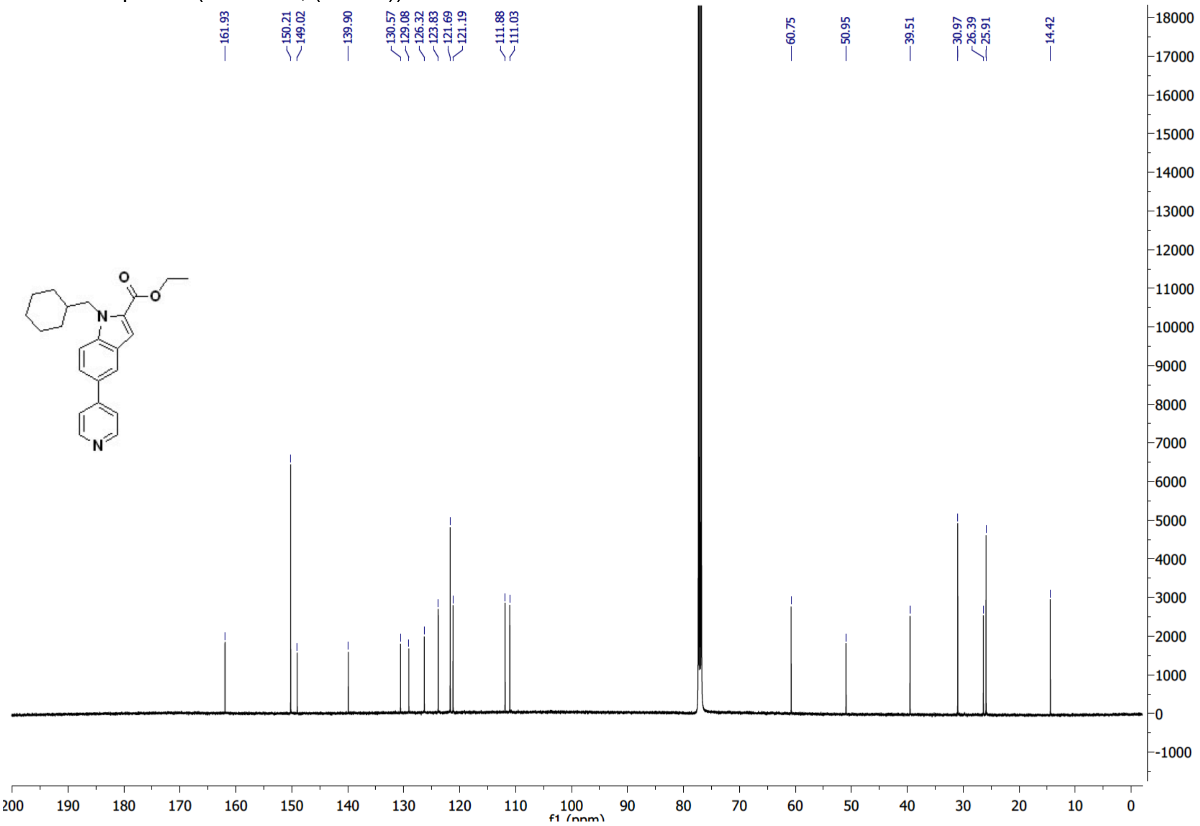

Ethyl 1-(cyclobutylmethyl)-5-(pyridin-4-yl)-1H-indole-2-carboxylate (**9**)

^{1}H NMR Spectra (400 MHz, (CDCl3))

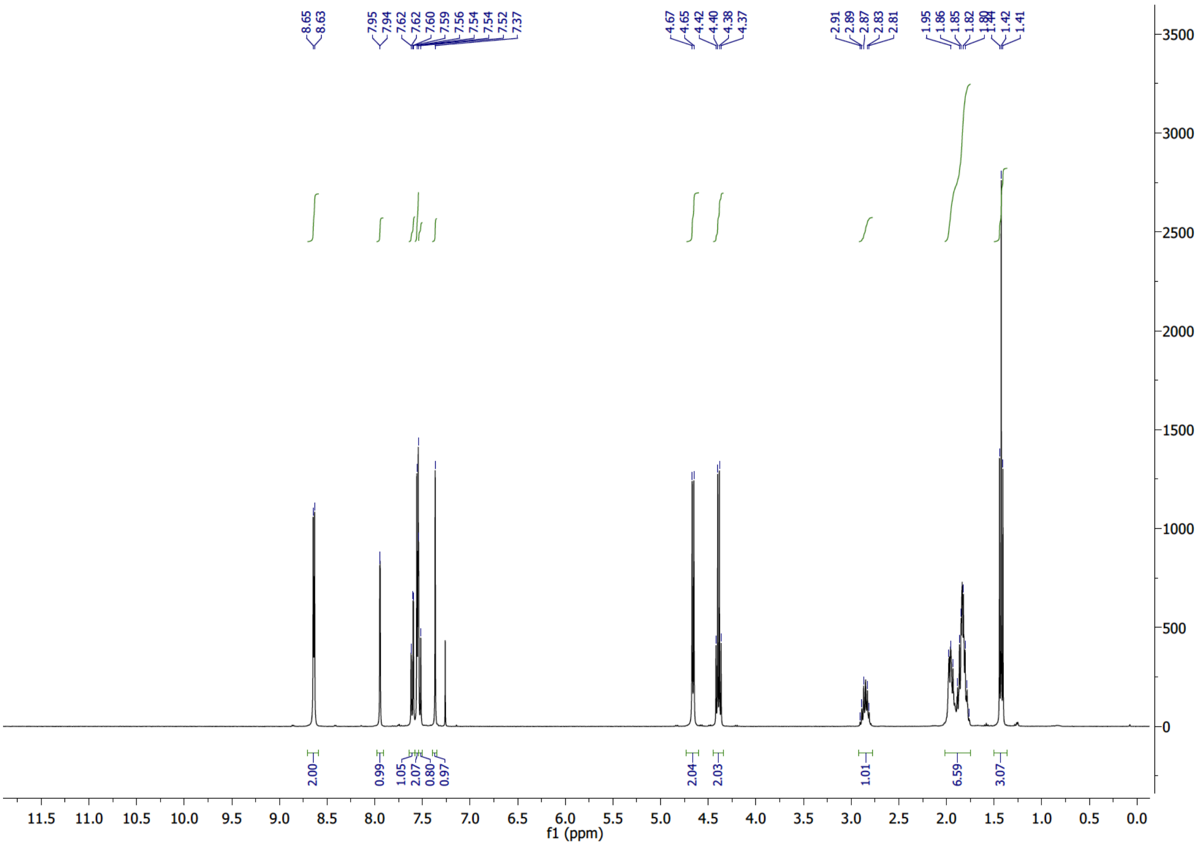

^{13}C NMR Spectra (101 MHz, (CDCl3))

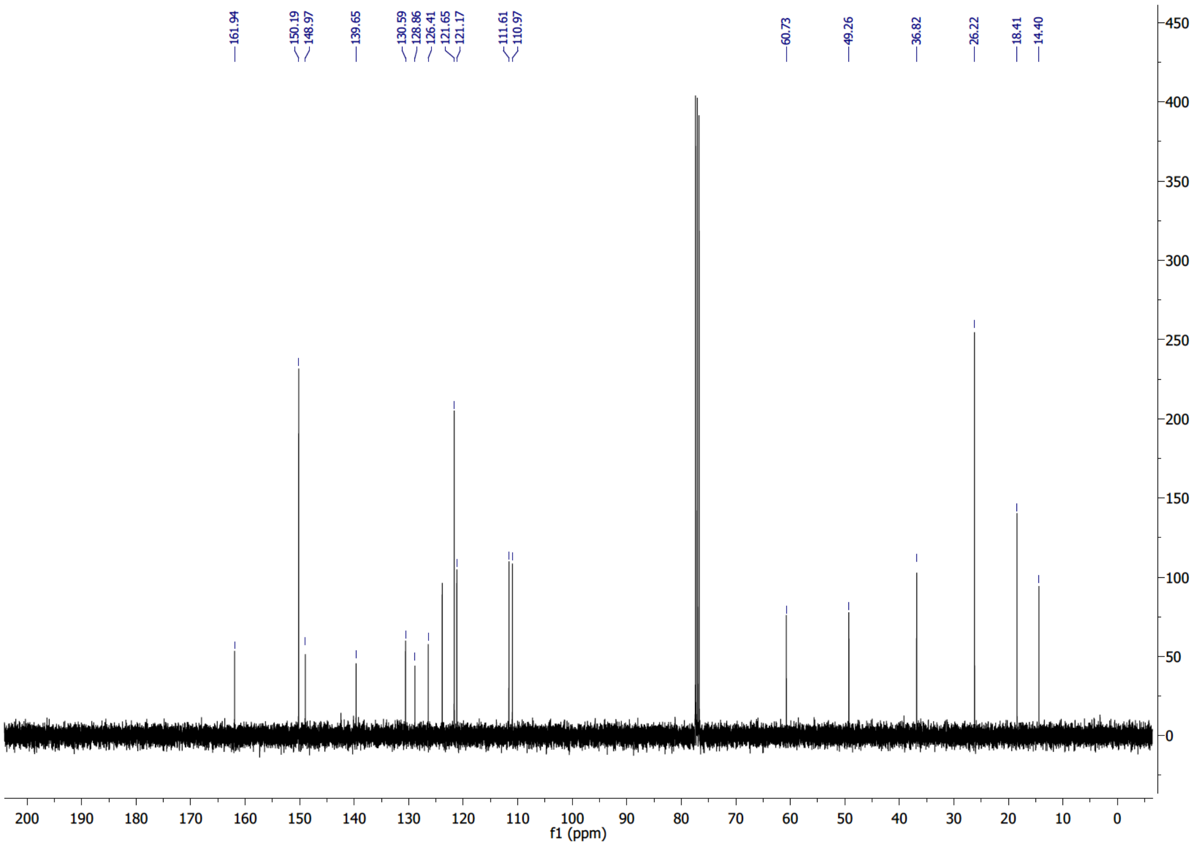

Ethyl 1-(cyclopentylmethyl)-5-(pyridin-4-yl)-1H-indole-2-carboxylate (**8**)

^{1}H NMR Spectra (400 MHz, (CDCl$_3$))

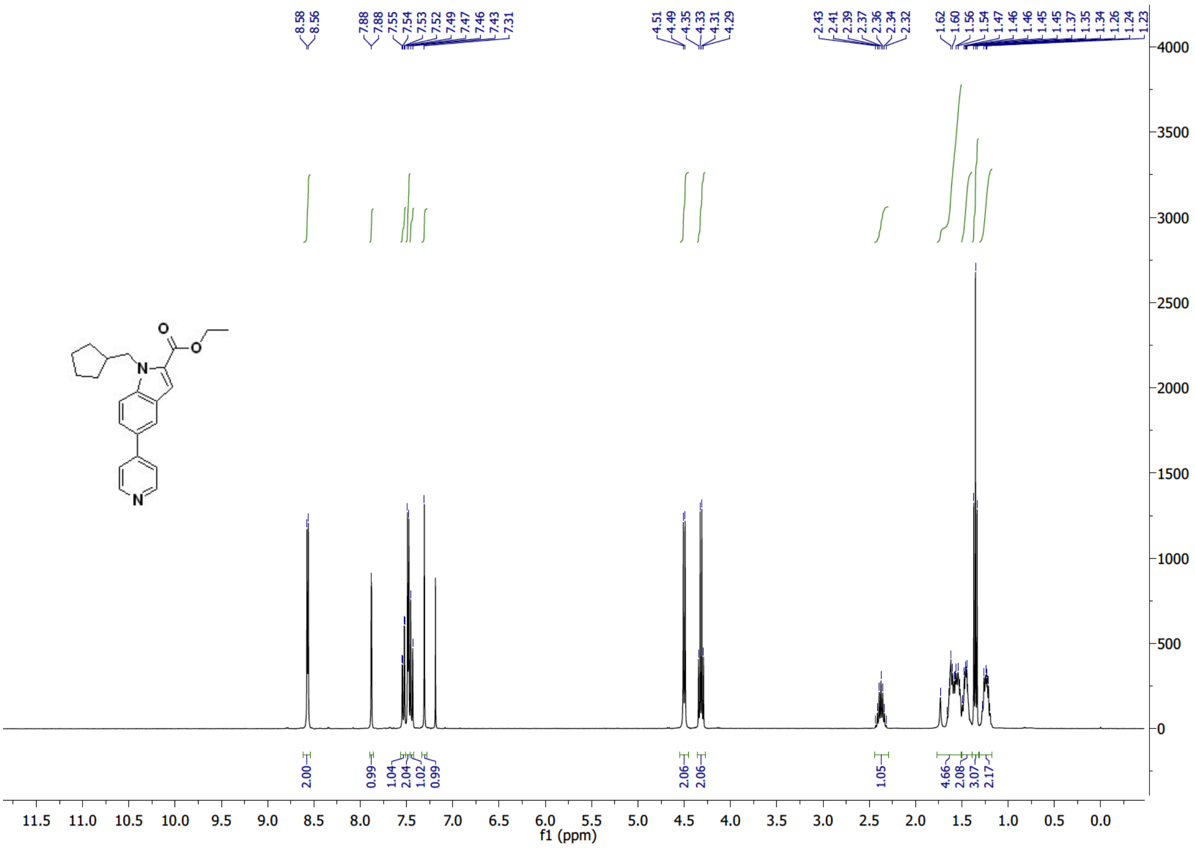

^{13}C NMR Spectra (101 MHz, (CDCl$_3$))

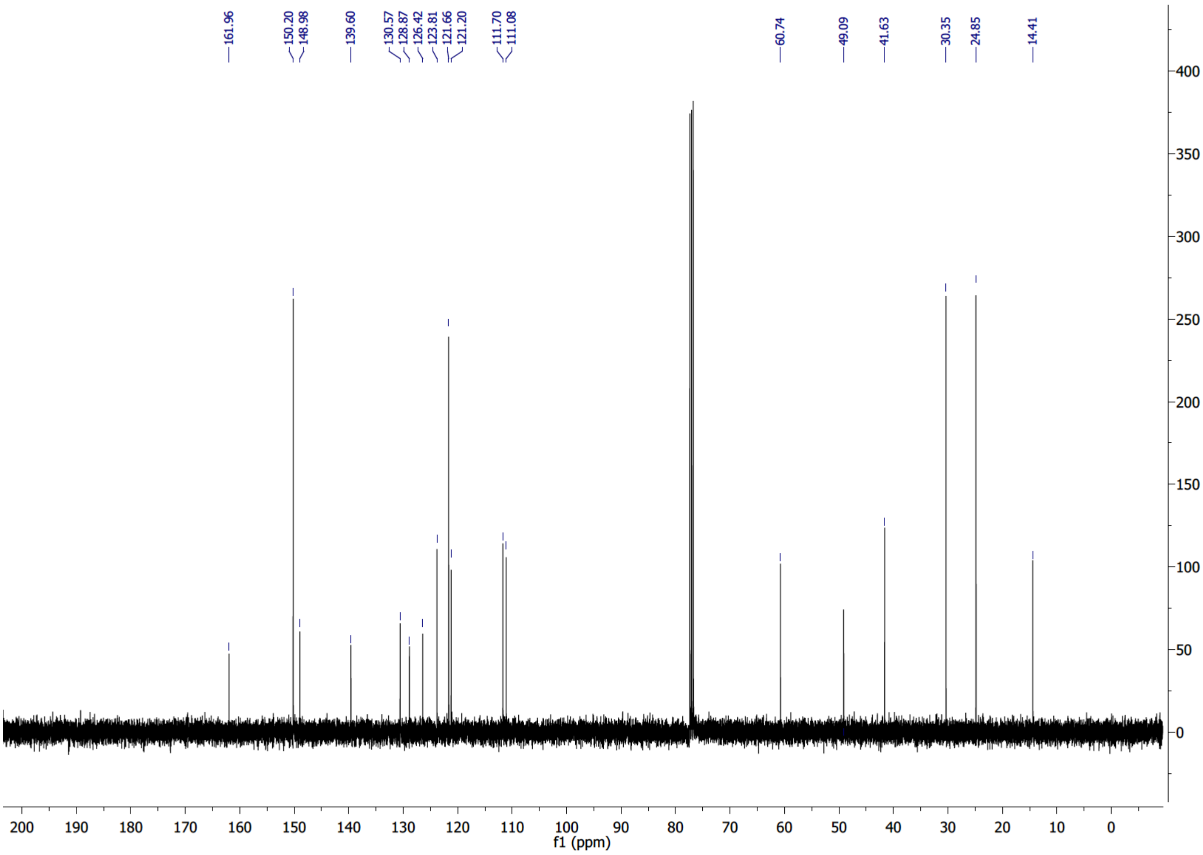

1-(cyclohexylmethyl)-5-(pyridin-4-yl)-1H-indole-2-carboxamide (**13**)

^{1}H NMR Spectra (400 MHz, ($CDCl_3$))

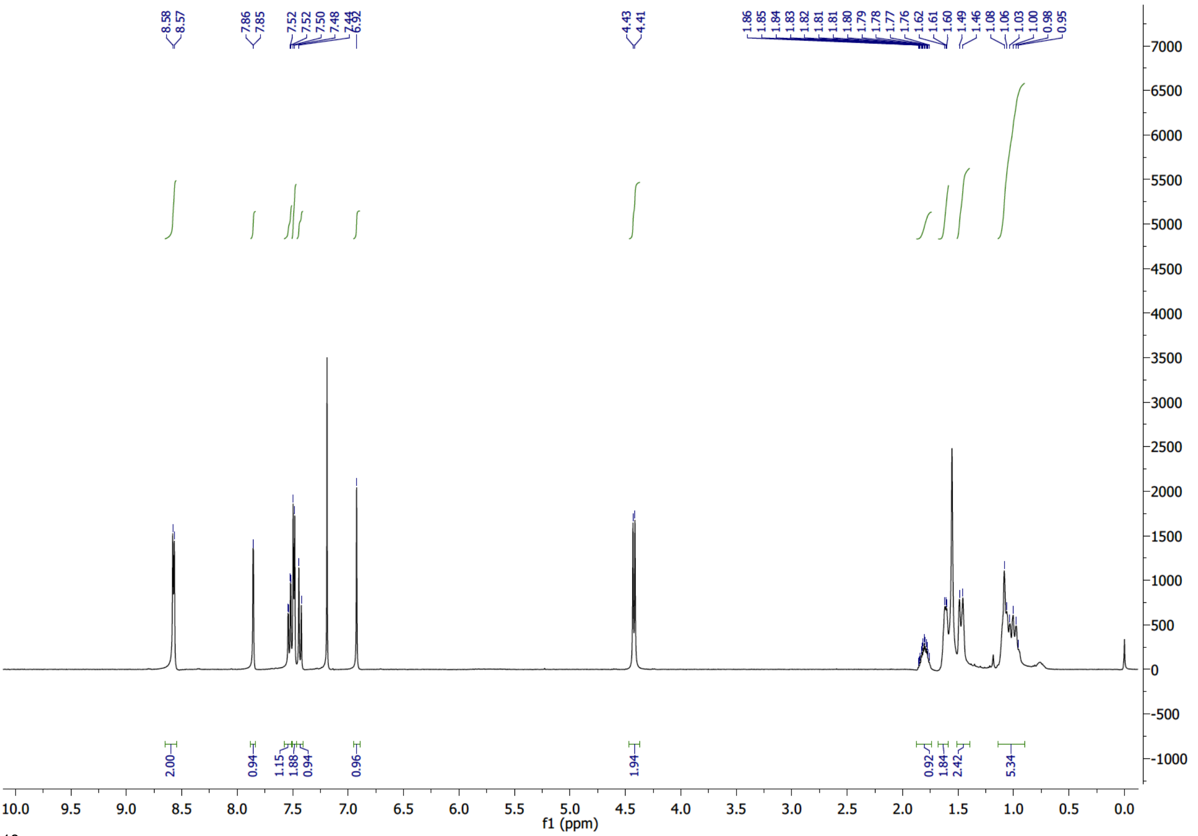

^{13}C NMR Spectra (101 MHz, ($CDCl_3$))

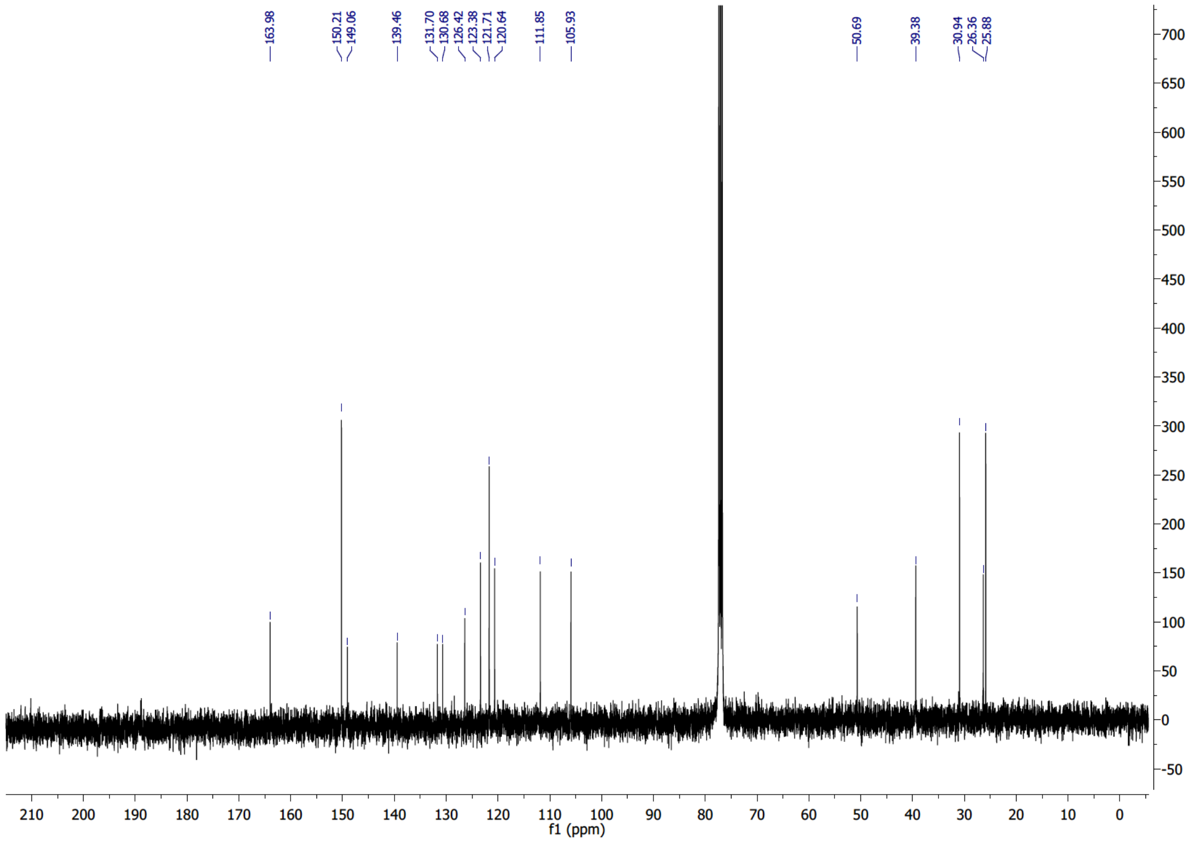

Ethyl 1-(piperidin-4-ylmethyl)-5-(pyridin-4-yl)-1H-indole-2-carboxylate (TFA salt) (**11**)

^{1}H NMR Spectra (400 MHz, ($CDCl_3$))

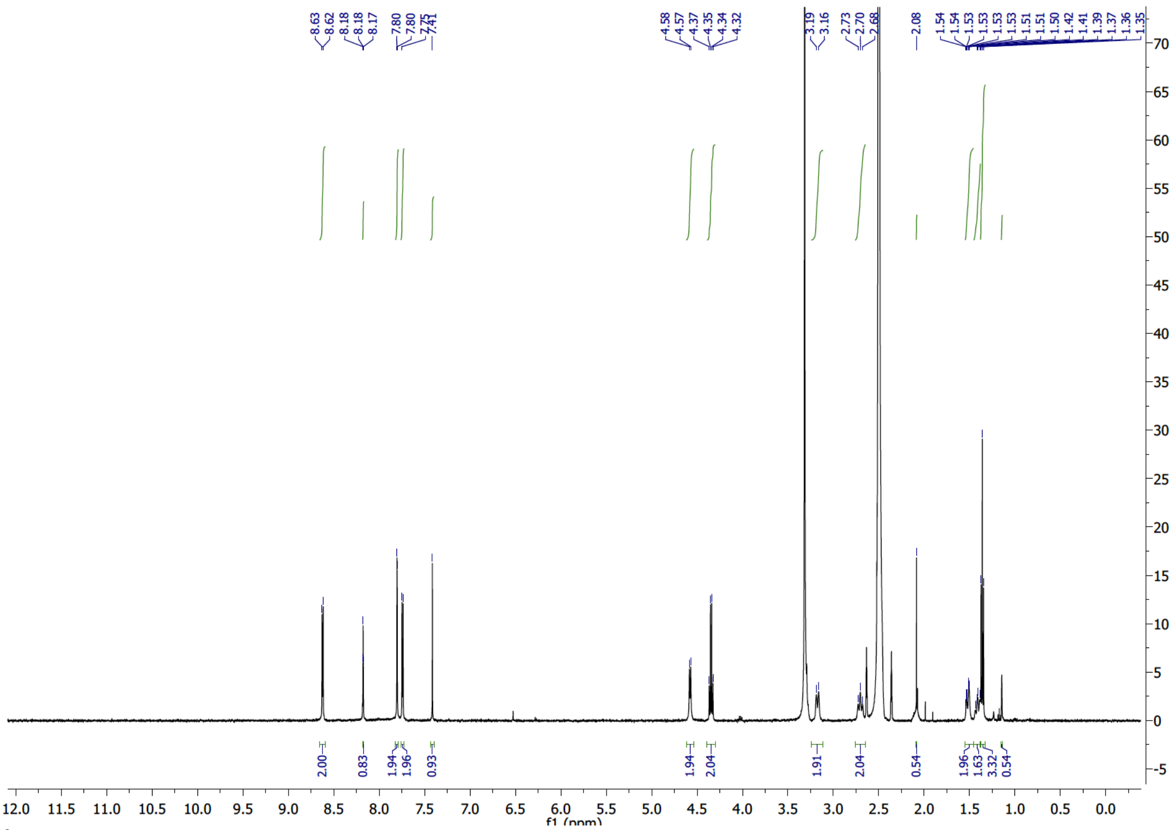

^{13}C NMR Spectra (101 MHz, ($CDCl_3$))

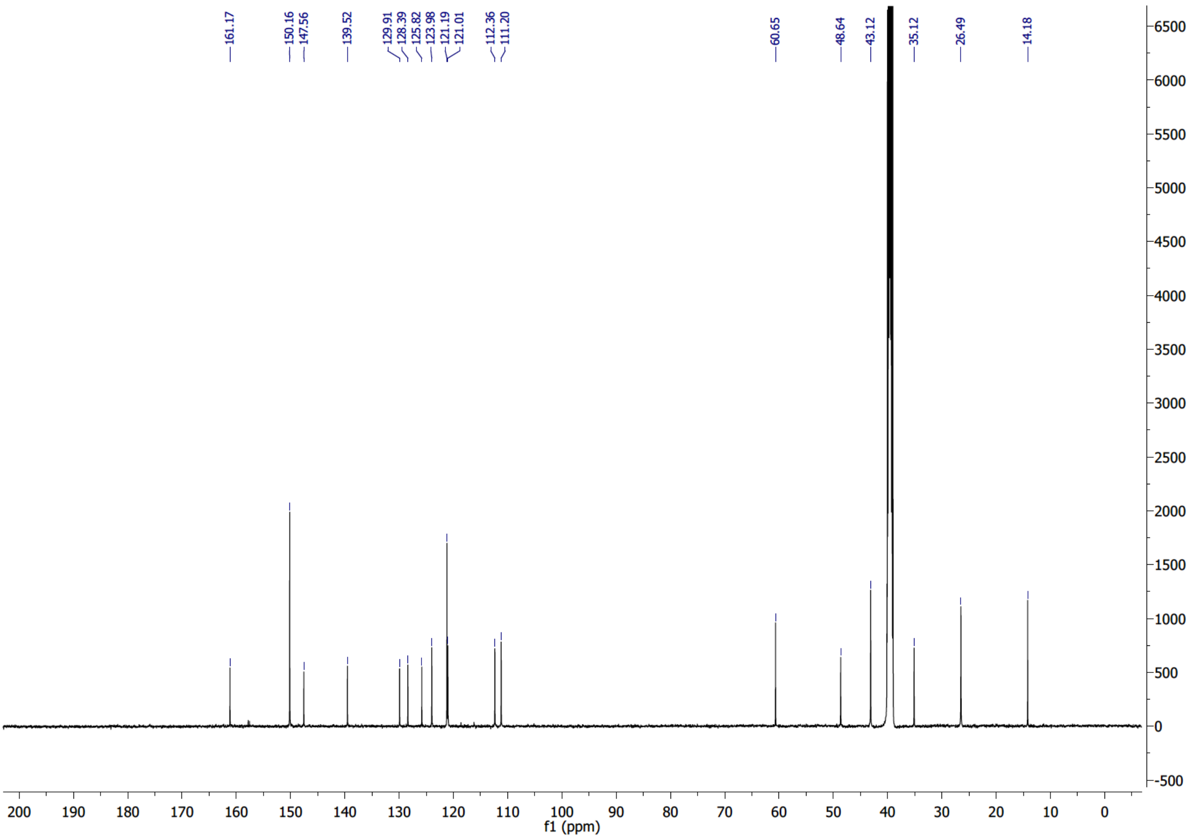

Ethyl 1-(2-(piperidin-4-yl)ethyl)-5-(pyridin-4-yl)-1H-indole-2-carboxylate (10)
^{1}H NMR Spectra (400 MHz, ($CDCl_3$))

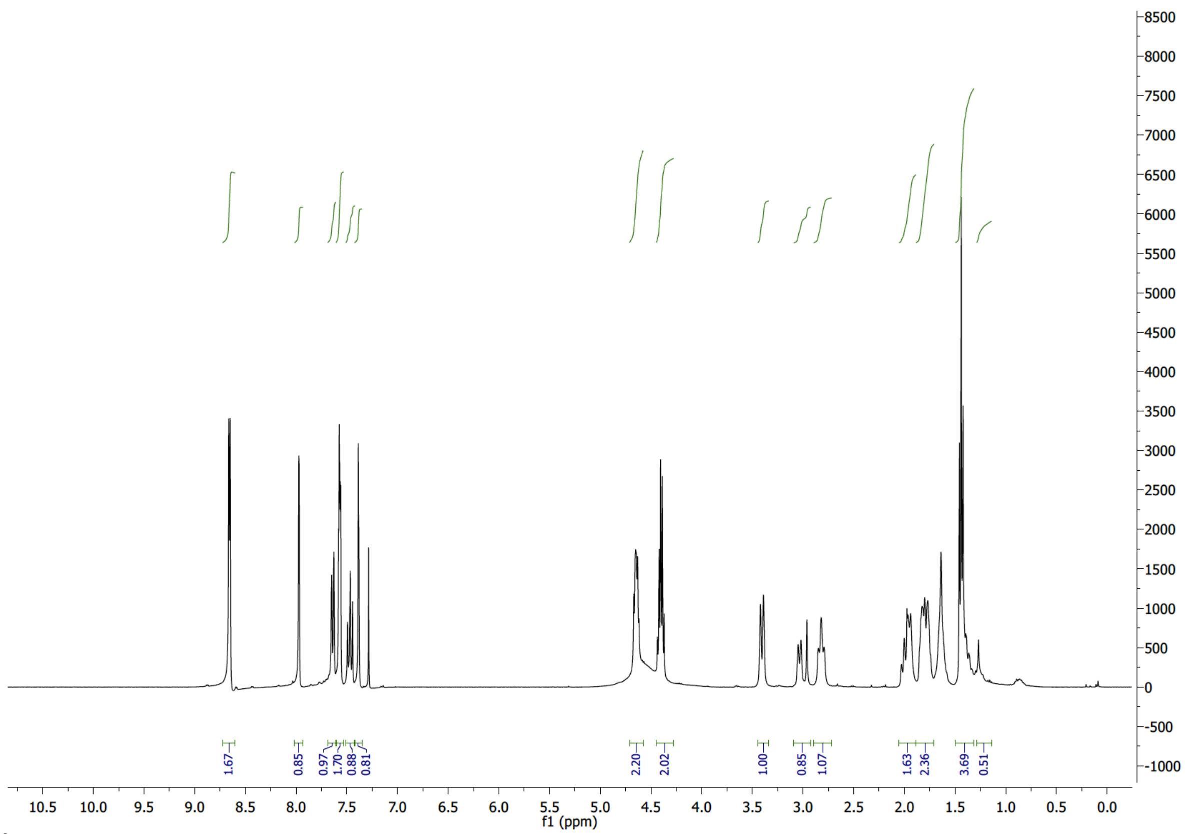

^{13}C NMR Spectra (101 MHz, ($CDCl_3$))

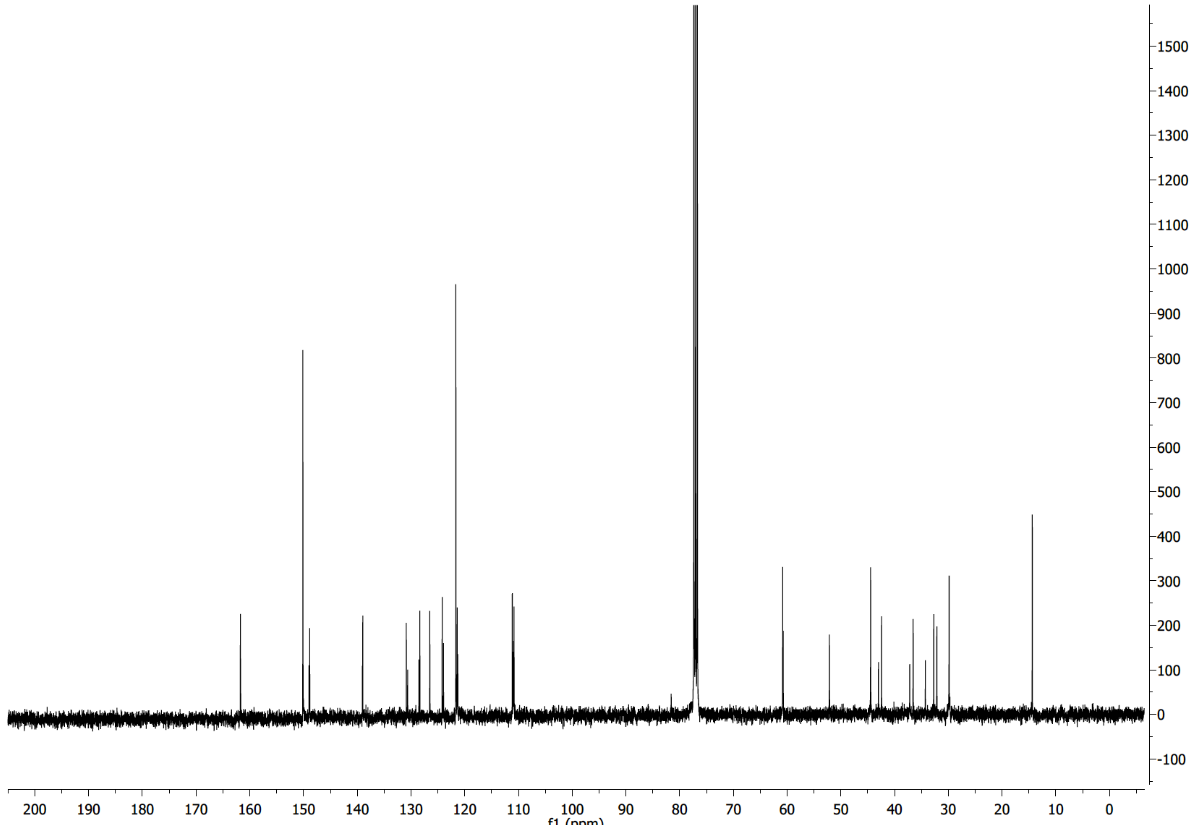

Ethyl 1-(2-morpholinoethyl)-5-(pyridin-4-yl)-1H-indole-2-*carboxylate* (12)

^{1}H NMR (500 MHz, DMSO-d$_6$)

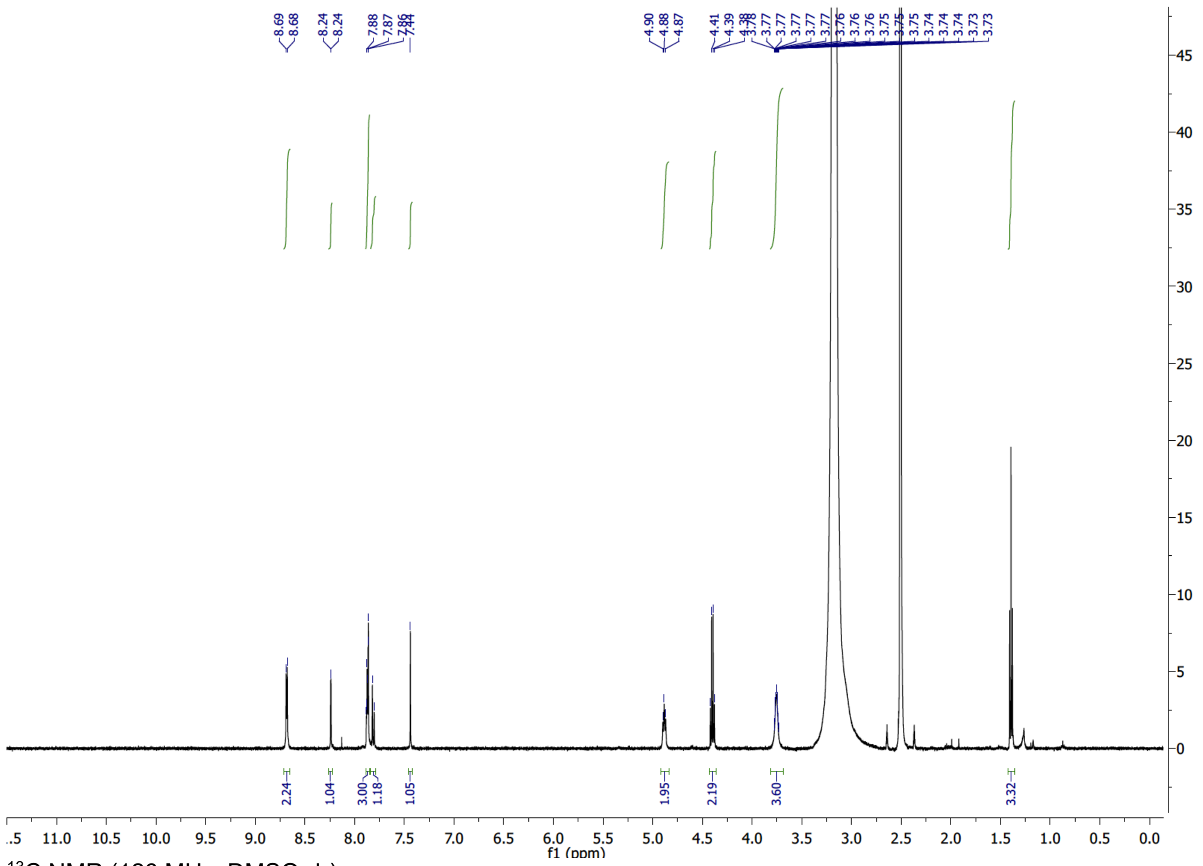

^{13}C NMR (126 MHz, DMSO-d$_6$)

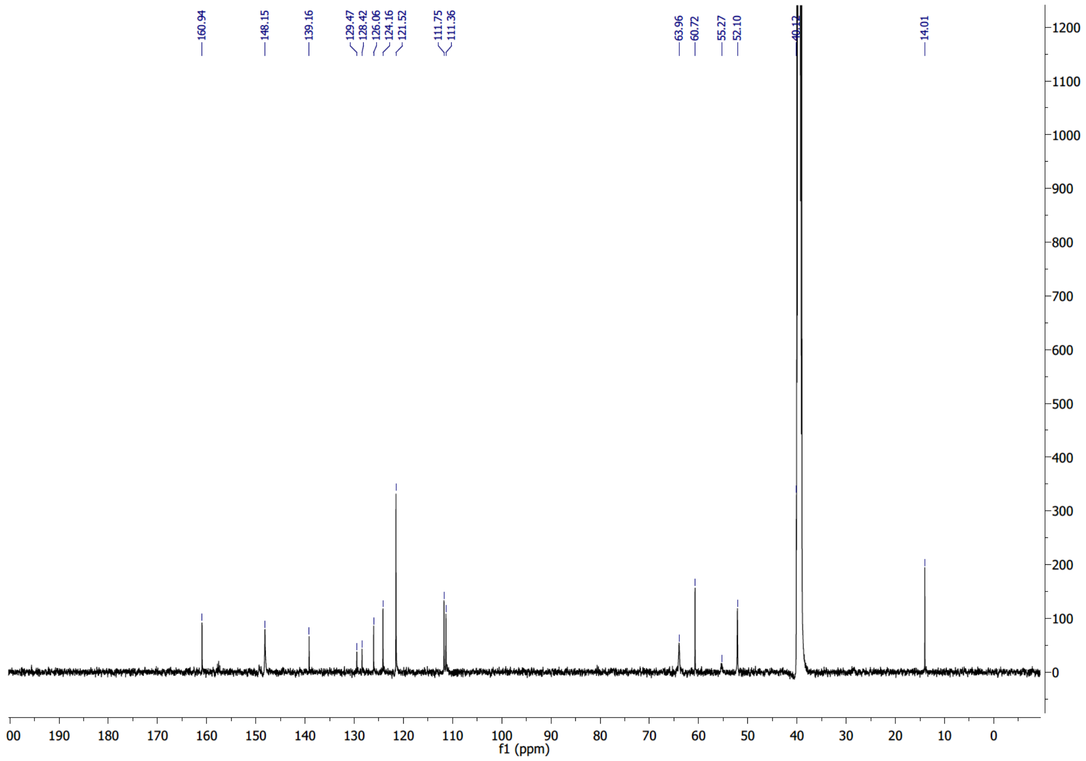

1-(2-(piperazin-1-yl)ethyl)-5-(pyridin-4-yl)-1H-indole (TFA Salt) (14)

^{1}H NMR (400 MHz, DMSO-d$_6$)

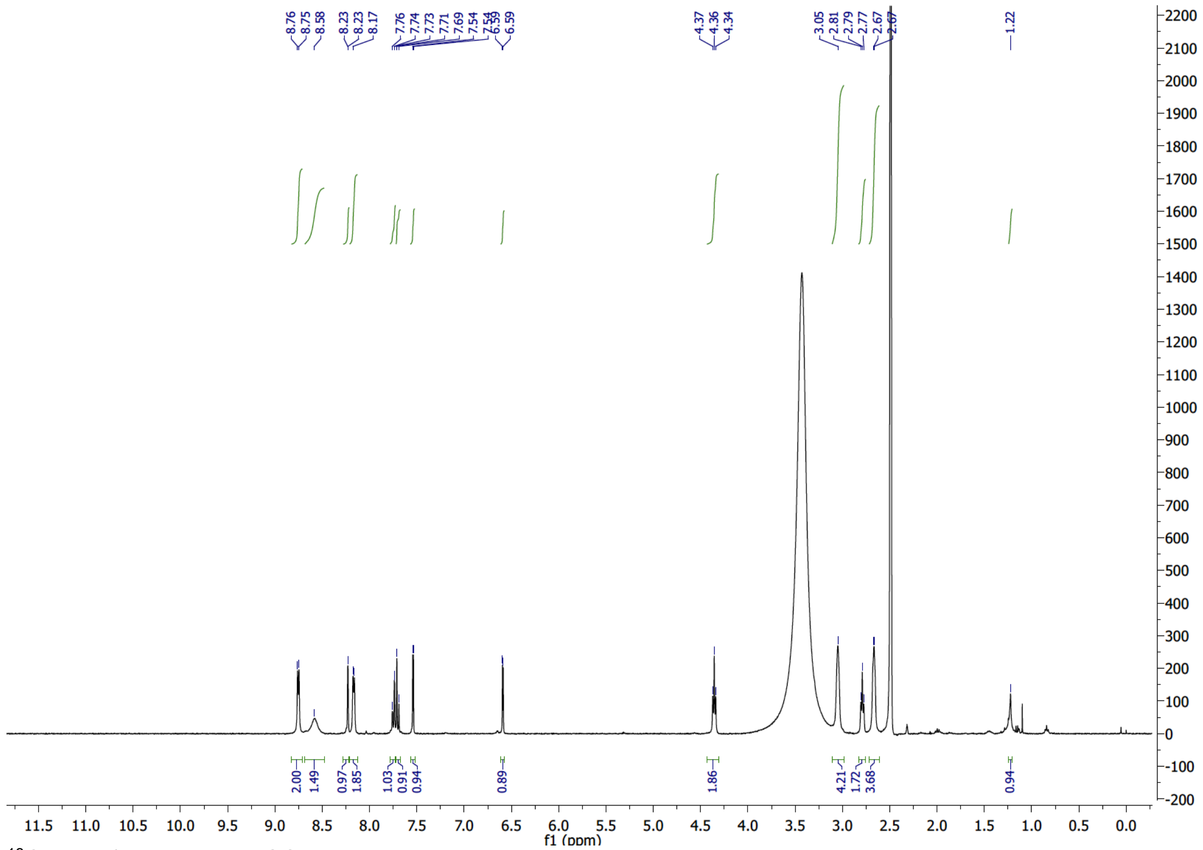

^{13}C NMR (126 MHz, DMSO-d_6

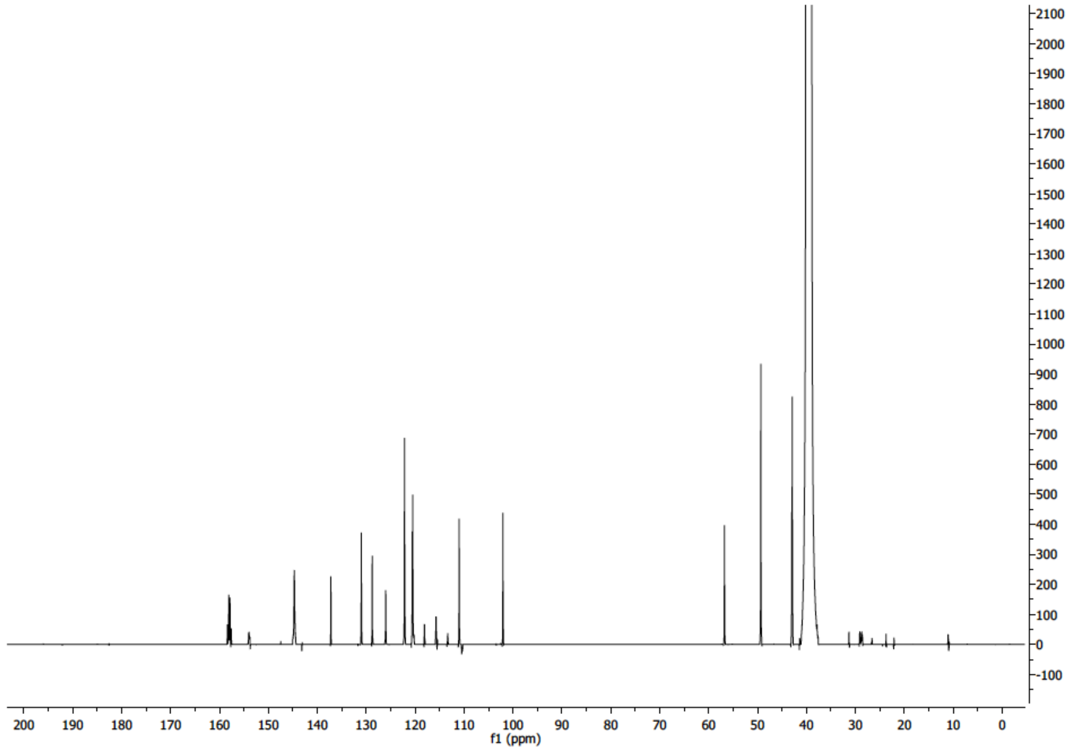

1-(2-(piperazin-1-yl)ethyl)-5-(pyridin-4-yl)-1H-indole-2-carboxamide (TFA Salt) (**15**)

^{1}H NMR (500 MHz, DMSO-d_6)

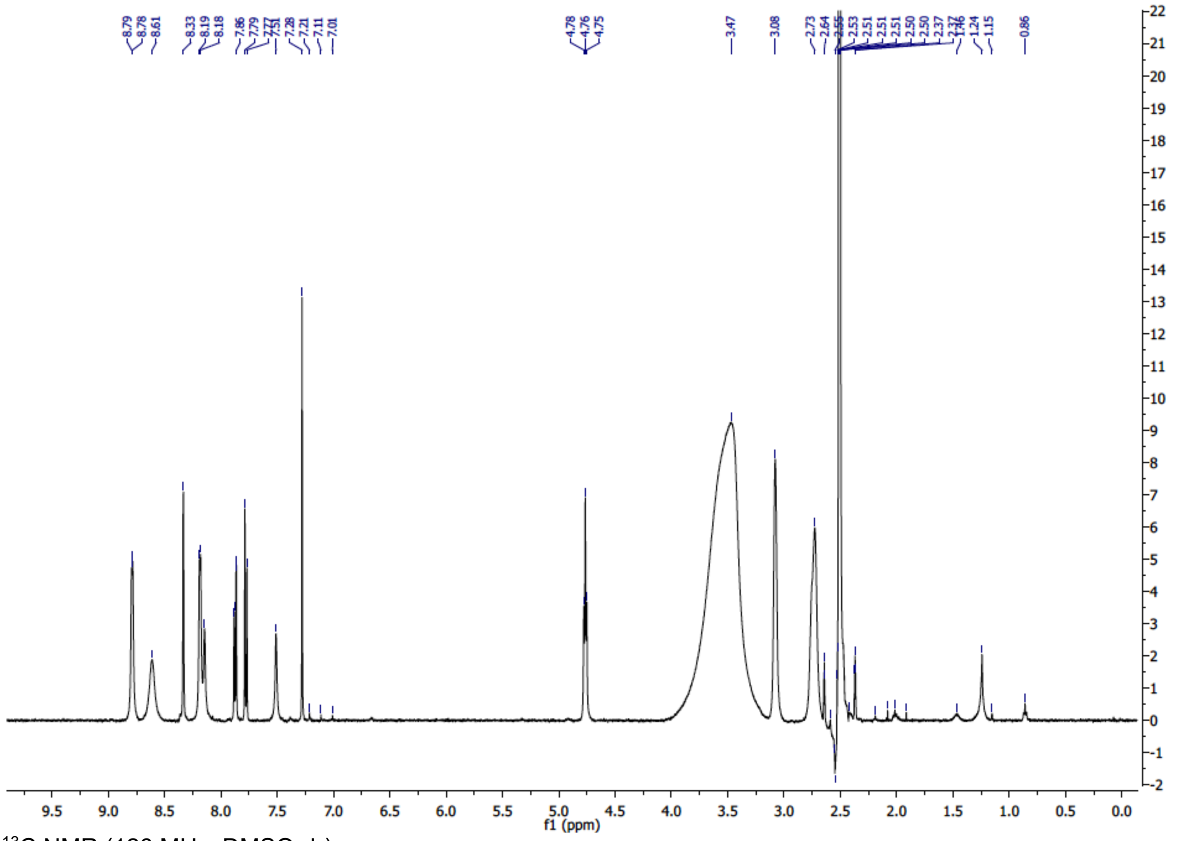

^{13}C NMR (126 MHz, DMSO-d_6)

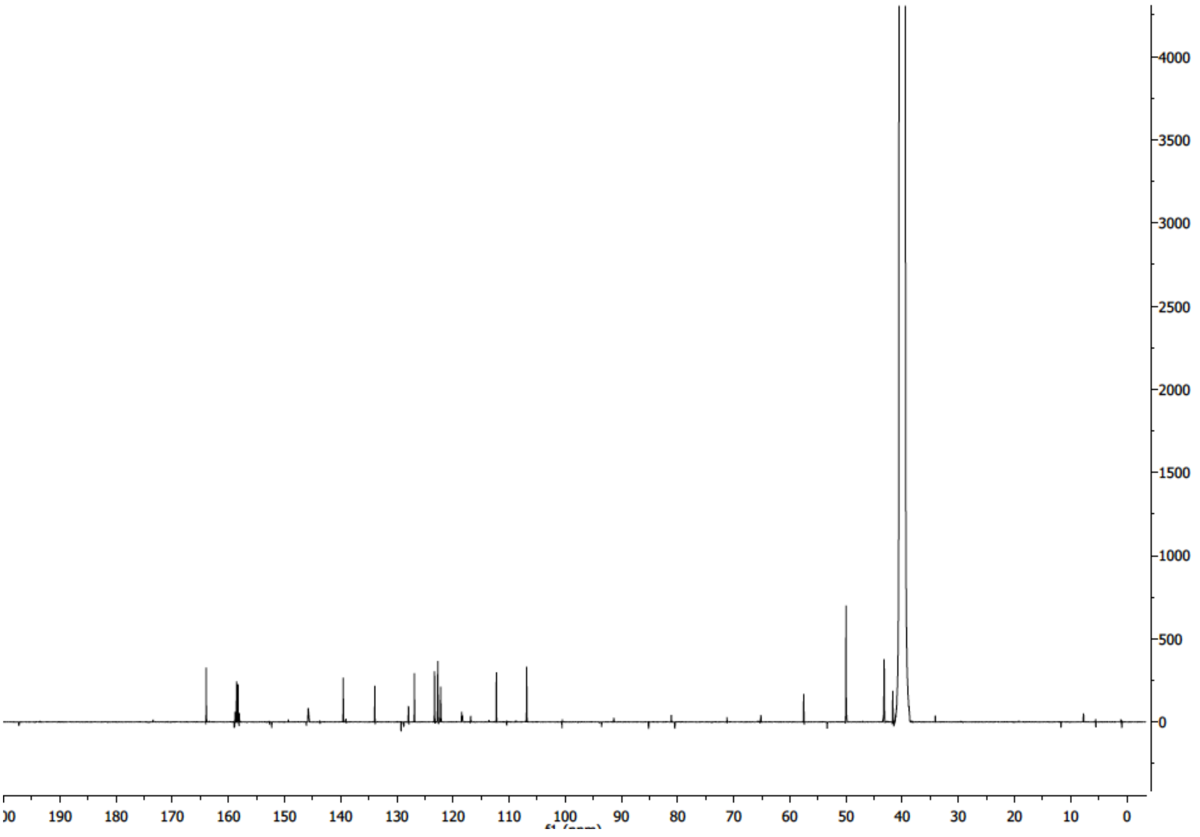

3-Methyl-5-(1-(2-(piperidin-4-yl)ethyl)-5-(pyridin-4-yl)-1H-indol-2-yl)-1,2,4-oxadiazole (TFA salt) (16)

^{1}H NMR (500 MHz, DMSO-d_6)

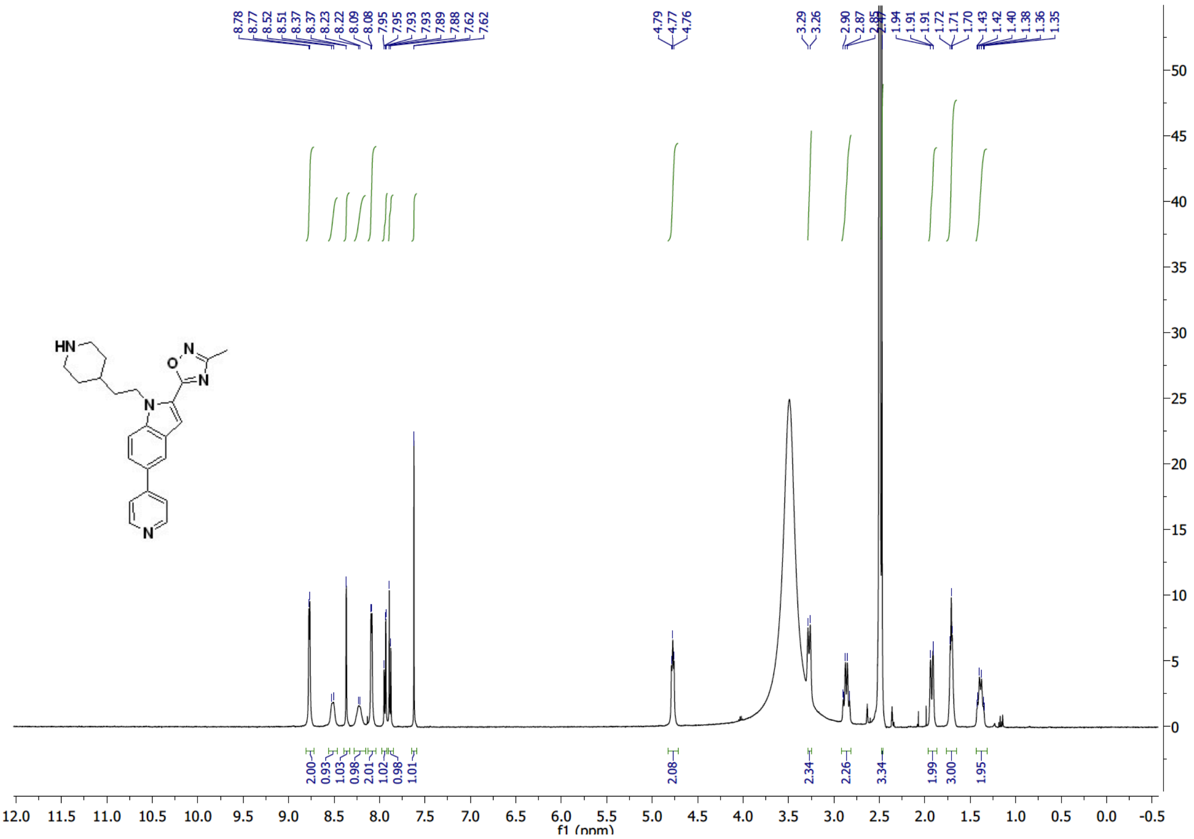

^{13}C NMR (126 MHz, DMSO-d$_6$)

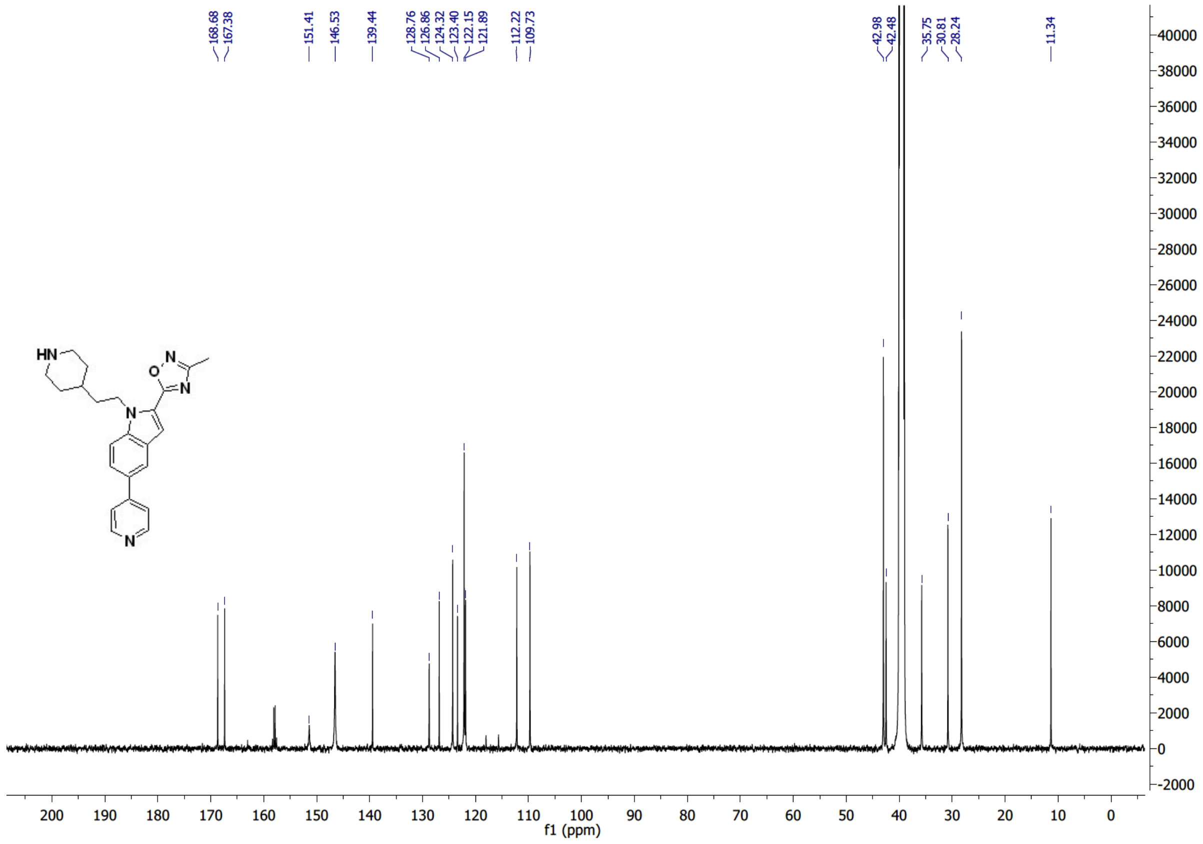

5-Methyl-2-(1-(2-(piperidin-4-yl)ethyl)-5-(pyridin-4-yl)-1H-indol-2-yl)oxazole (TFA salt) (17)

^{1}H NMR (400 MHz, DMSO-d$_6$)

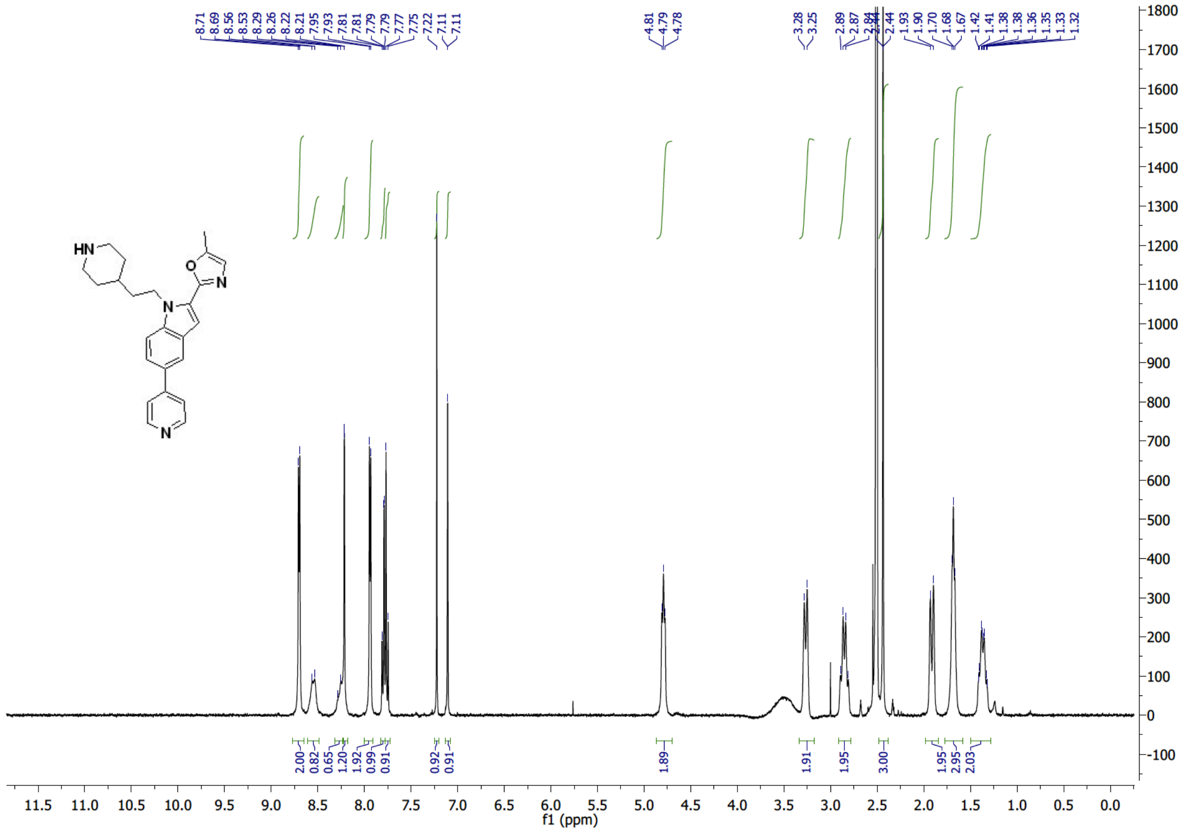

^{13}C NMR (126 MHz, DMSO-d_6)

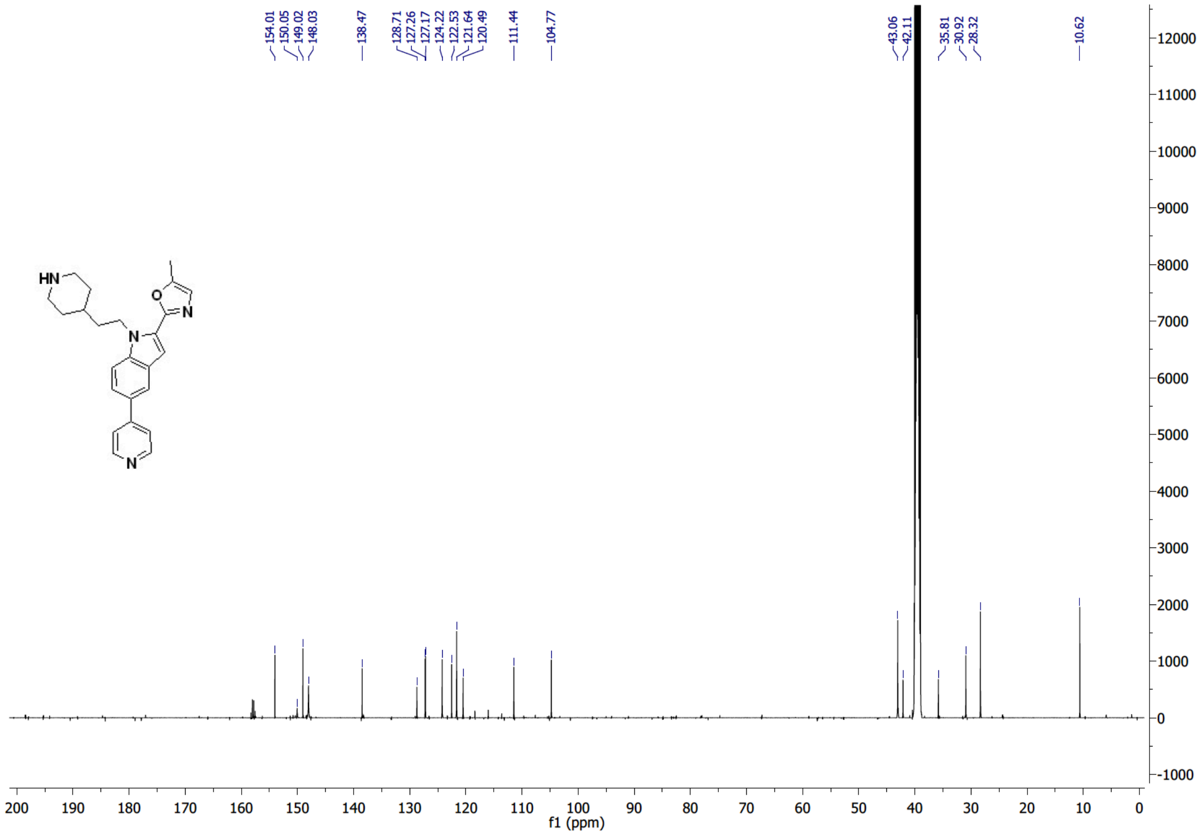

2-Methyl-5-(1-(2-(piperidin-4-yl)ethyl)-5-(pyridin-4-yl)-1H-indol-2-yl)-1,3,4-oxadiazole (TFA salt) (**18**)

^{1}H NMR (400 MHz, DMSO-d_6)

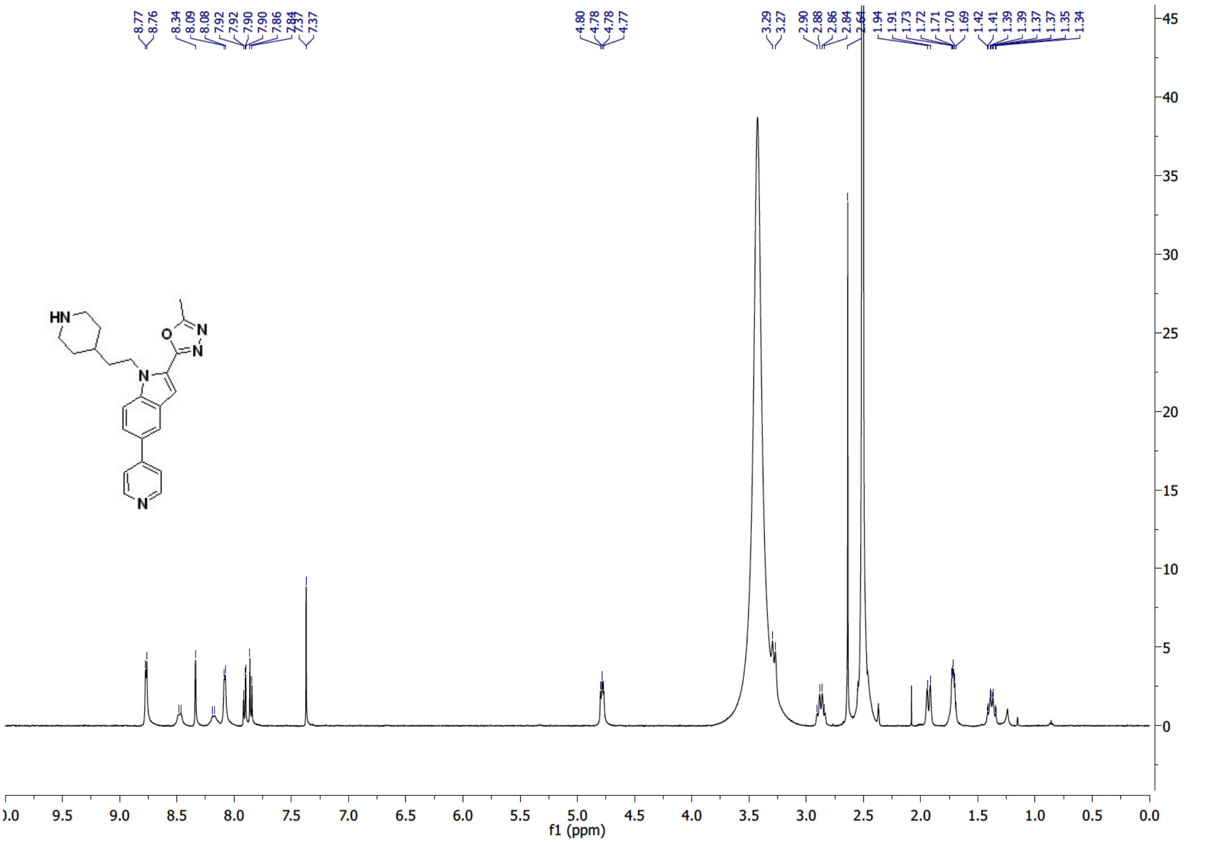

^{13}C NMR (126 MHz, DMSO-d_6)

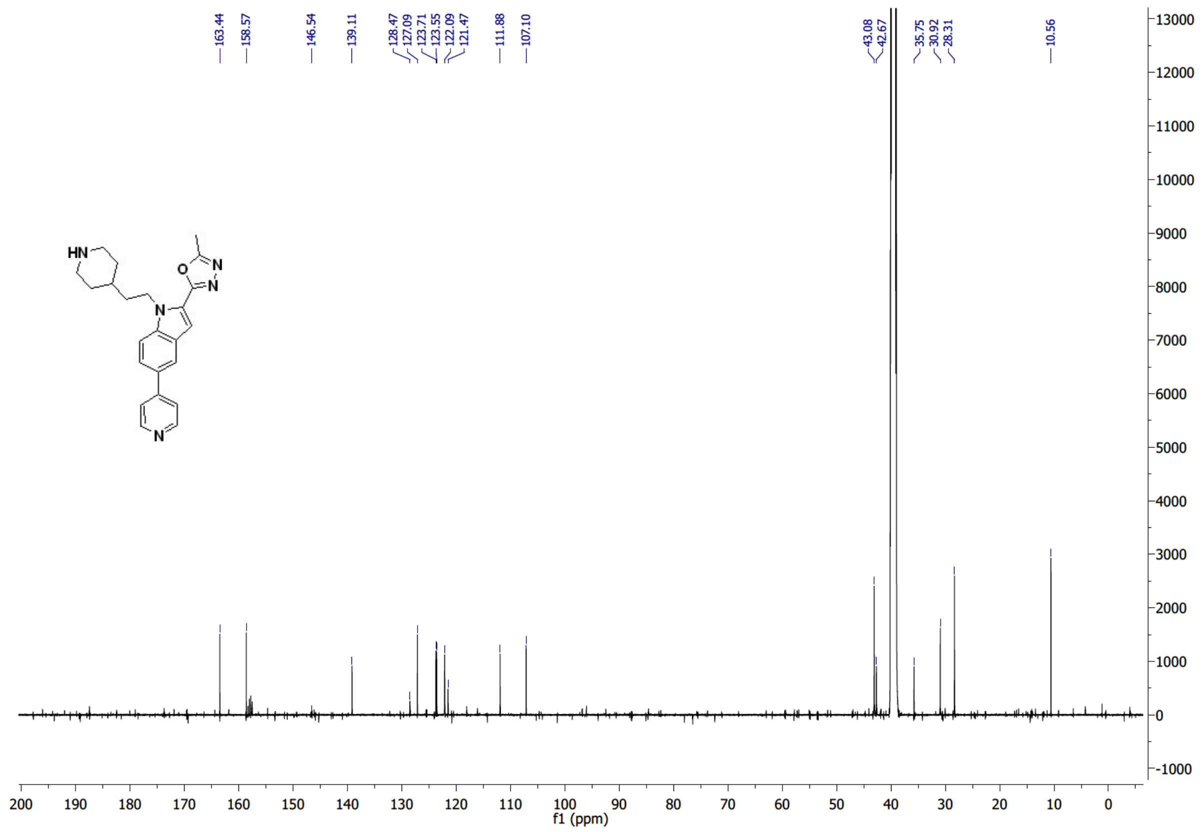

1-(1-(2-(piperidin-4-yl)ethyl)-5-(pyridin-4-yl)-1H-indol-2-yl)butan-1-one (TFA salt) (**19**)

^{1}H NMR (400 MHz, DMSO-d_6)

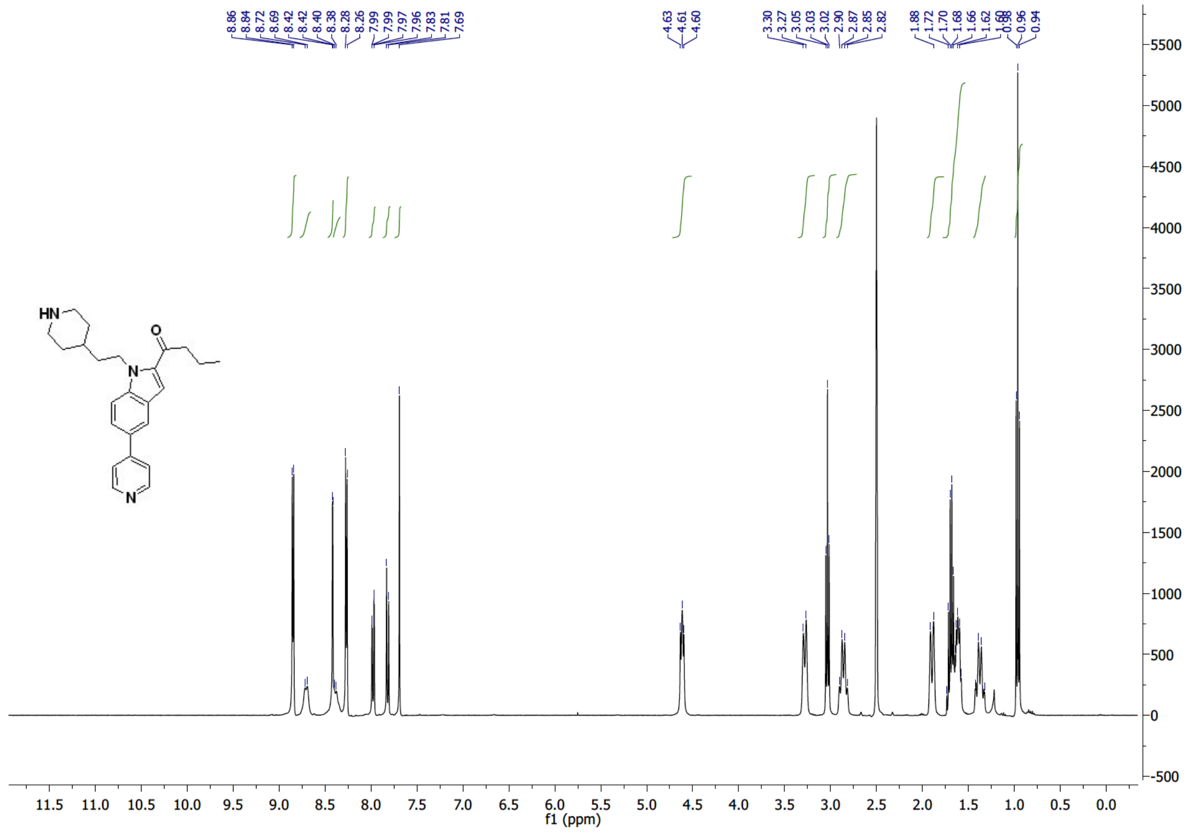

^{13}C NMR (126 MHz, DMSO-d6)

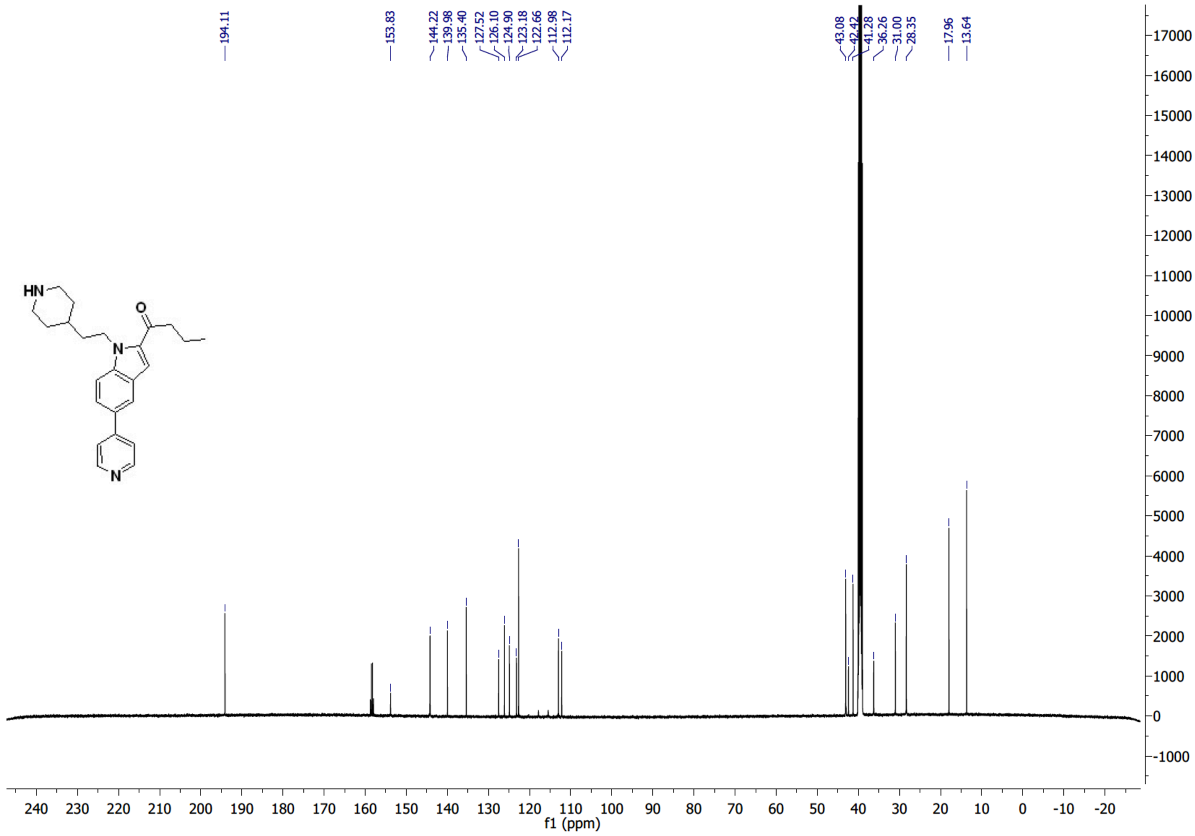

Figure S2: Selected LMCS chromatograms of screened compounds

Compound **4**

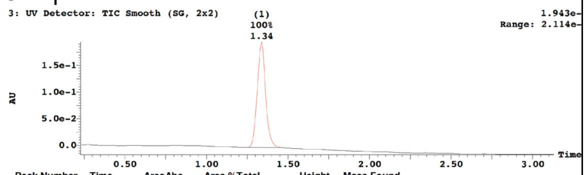

Peak Number	Time	AreaAbs	Area %Total	Height	Mass Found
1	1.34	11744	100.00	198241	

Compound **5**

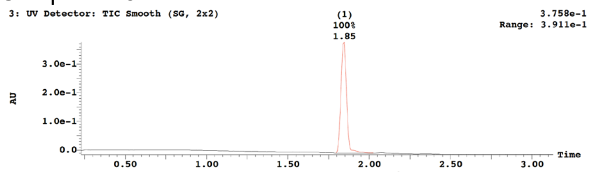

Peak Number	Time	AreaAbs	Area %Total	Height	Mass Found
1	1.85	15490	100.00	386081	

Compound **6**

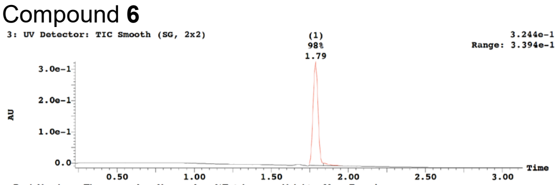

Peak Number	Time	AreaAbs	Area %Total	Height	Mass Found
1	1.79	12293	97.51	331761	
2	1.85	313	2.49	7049	

Compound **7**

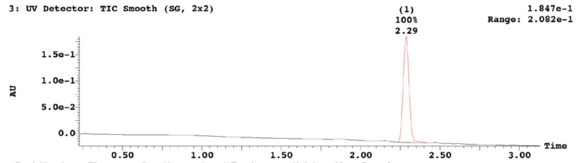

Peak Number	Time	AreaAbs	Area %Total	Height	Mass Found
1	2.29	8435	100.00	201696	

Compound **9**

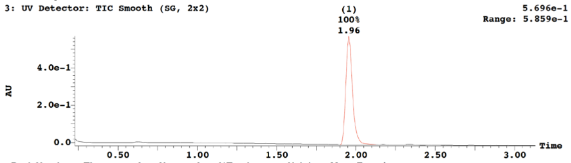

Peak Number	Time	AreaAbs	Area %Total	Height	Mass Found
1	1.96	28135	100.00	581367	

Compound **10**

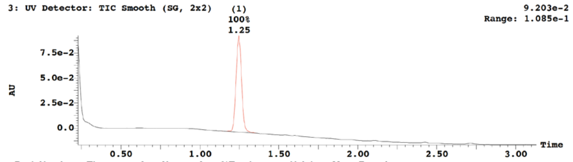

Peak Number	Time	AreaAbs	Area %Total	Height	Mass Found
1	1.25	3887	100.00	95955	

Compound **11**

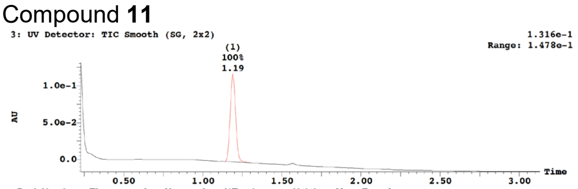

Peak Number	Time	AreaAbs	Area %Total	Height	Mass Found
1	1.19	4739	100.00	118966	

Compound **13**

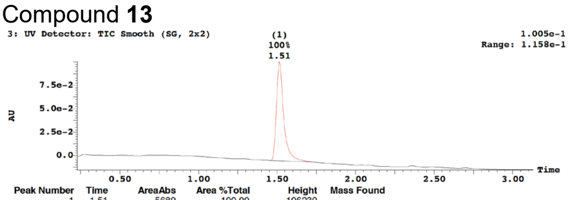

Peak Number	Time	AreaAbs	Area %Total	Height	Mass Found
1	1.51	5689	100.00	106239	

Compound **16**

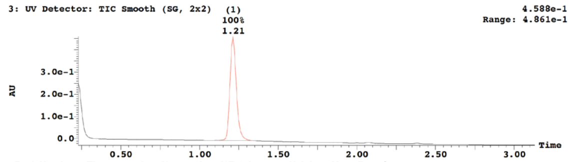

Peak Number	Time	AreaAbs	Area %Total	Height	Mass Found
1	1.21	21746	100.00	465500	

Compound **17**

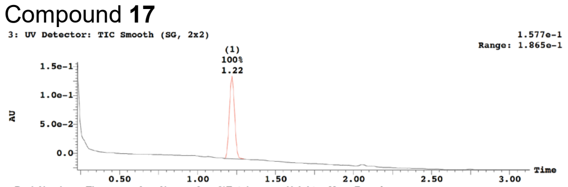

Peak Number	Time	AreaAbs	Area %Total	Height	Mass Found
1	1.22	5466	100.00	143208	

Compound **18**

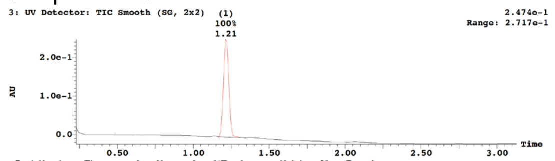

Peak Number	Time	AreaAbs	Area %Total	Height	Mass Found
1	1.21	10309	100.00	253535	

Compound **19**

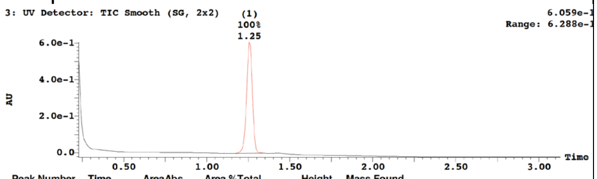

Peak Number	Time	AreaAbs	Area %Total	Height	Mass Found
1	1.25	25001	100.00	609235	
